# Sequence Diversity in Coding Regions of Candidate Genes in the Glycoalkaloid Biosynthetic Pathway of Wild Potato Species

**DOI:** 10.1534/g3.113.007146

**Published:** 2013-09-01

**Authors:** Norma C. Manrique-Carpintero, James G. Tokuhisa, Idit Ginzberg, Jason A. Holliday, Richard E. Veilleux

**Affiliations:** * Department of Horticulture, Virginia Polytechnic Institute and State University, Blacksburg, Virginia 24061; † Department of Vegetable Research, The Volcani Center, Agricultural Research Organization, Bet Dagan, Israel; ‡ Department of Forest Resources and Environmental Conservation, Virginia Polytechnic Institute and State University, Blacksburg, Virginia 24061

**Keywords:** nucleotide diversity, dN/dS ratio, *Solanum*, Infinium 8303 Potato Array

## Abstract

Natural variation in five candidate genes of the steroidal glycoalkaloid (SGA) metabolic pathway and whole-genome single nucleotide polymorphism (SNP) genotyping were studied in six wild [*Solanum chacoense* (*chc* 80-1), *S. commersonii*, *S. demissum*, *S. sparsipilum*, *S. spegazzinii*, *S. stoloniferum*] and cultivated *S. tuberosum* Group Phureja (*phu* DH) potato species with contrasting levels of SGAs. Amplicons were sequenced for five candidate genes: 3-hydroxy-3-methylglutaryl coenzyme A reductase 1 and 2 (*HMG1*, *HMG2*) and 2.3-squalene epoxidase (*SQE*) of primary metabolism, and solanidine galactosyltransferase (*SGT1*), and glucosyltransferase (*SGT2*) of secondary metabolism. SNPs (n = 337) producing 354 variations were detected within 3.7 kb of sequenced DNA. More polymorphisms were found in introns than exons and in genes of secondary compared to primary metabolism. Although no significant deviation from neutrality was found, dN/dS ratios < 1 and negative values of Tajima’s D test suggested purifying selection and genetic hitchhiking in the gene fragments. In addition, patterns of dN/dS ratios across the SGA pathway suggested constraint by natural selection. Comparison of nucleotide diversity estimates and dN/dS ratios showed stronger selective constraints for genes of primary rather than secondary metabolism. SNPs (n = 24) with an exclusive genotype for either *phu* DH (low SGA) or *chc* 80-1 (high SGA) were identified for *HMG2*, *SQE*, *SGT1* and *SGT2*. The SolCAP 8303 Illumina Potato SNP chip genotyping revealed eight informative SNPs on six pseudochromosomes, with homozygous and heterozygous genotypes that discriminated high, intermediate and low levels of SGA accumulation. These results can be used to evaluate SGA accumulation in segregating or association mapping populations.

Steroidal glycoalkaloids (SGAs) are secondary metabolites mainly produced in solanaceous species. These compounds function in potato species as a defense against pathogens and insects (Wink 2003; Friedman 2006; Nema *et al.* 2008). The SGA structure consists of a hydrophobic C_27_-steroidal alkaloid skeleton (aglycone) containing a nitrogen atom as a secondary or tertiary amine and a hydrophilic glycosidic moiety of three or four sugars attached to the C-3 hydroxyl position of the aglycone (Bushway *et al.* 1980; Milner *et al.* 2011). This chemical structure has cytotoxic properties, such as the inhibition of acetylcholinesterase activity and the disruption of cell membrane function (Orgell *et al.* 1958; Roddick *et al.* 1990; Keukens *et al.* 1995). Several types of SGAs have been reported in cultivated potato (*Solanum tuberosum* L. Group Tuberosum) and wild relative species, the most commonly accumulated are the triose glycosides of solanidine, α-chaconine and α-solanine (Shakya and Navarre 2008; Distl and Wink 2009). Some of the SGAs that have been identified as the most effective poisonous compounds against different potato pests are demissine, commersonine, dehydrocommersonine, and the leptines (Distl and Wink 2009). Several potato breeding programs have attempted to incorporate the resistance or tolerance to insects associated with these compounds into the cultivated potato by introgression (Sanford *et al.* 1996; Lorenzen *et al.* 2001; Sørensen *et al.* 2008; Thompson *et al.* 2008). However, tissue-specific accumulation of SGAs in leaves rather than overall production is required to breed potato resistant to its major pests to avoid toxicity in the tubers.

Wild potato species have an enormous genetic potential for potato breeding, both with regard to tuber quality and resistance to insects and pathogens. The tuber-bearing *Solanum* section *Petota*, with about 100 wild species and four cultivated species (Spooner 2009), is distributed from southwestern United States to Chile, Argentina, and Uruguay. Taxonomically they are a closely related group due to sexual compatibility among many species, introgression, interspecific hybridization, auto- and allopolyploidy, a mixture of sexual and asexual reproduction, possible recent species divergence, phenotypic plasticity, and morphologic similarity that make it difficult to distinguish species and series (Spooner and Salas 2006; Spooner 2009). The close relatedness within species of section *Petota* has allowed introgression of characteristics from multiple wild species in the cultivated potato. Fourteen wild species have been used to incorporate resistance to viral, fungal, and bacterial diseases, as well as to insect and nematode pests of potato (Spooner and Salas 2006). The presence of glycoalkaloids, dense hairs, and glandular trichomes are characteristics that have been identified in the wild potato species associated with resistance to major potato insect pests (Flanders *et al.* 1992). Breeding for resistance mediated by SGAs is complicated by polygenic inheritance, by the high correlation in SGA content between foliage and tubers, and because SGA accumulation is influenced by environmental factors and crop management activities (Tingey 1984). Previous studies seeking to identify molecular markers associated with SGA production demonstrated the complexity of the genetic control of SGA production (Yencho *et al.* 1998; Ronning *et al.* 1999; Hutvágner *et al.* 2001; Boluarte-Medina *et al.* 2002; Van Dam *et al.* 2003; Sagredo *et al.* 2006). Identification and cloning genes associated with synthesis and accumulation of SGAs present an alternative to either marker-assisted selection or genetic transformation. In the candidate gene approach, alleles of SGA-related biosynthetic genes within defined germplasm could be identified and used to determine the relationships between genetic and SGA variation.

The biosynthetic genes and the genetic factors that regulate the expression of SGAs are not fully understood. SGA metabolism diverges from the well-defined primary metabolism of the cytoplasmic mevalonic/isoprenoid pathway (Lichtenthaler *et al.* 1997; Liao *et al.* 2006; Okada 2011) (Figure 1). SGA synthesis commences with the formation of cholesterol; however, little is known about further metabolic steps involved in the conversion of cholesterol to SGAs.

**Figure 1:**
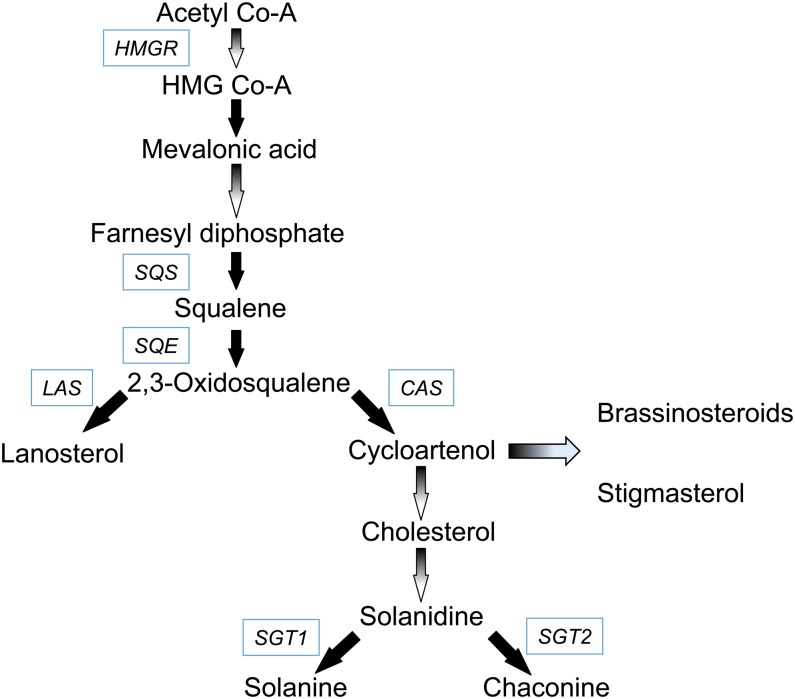
Potato steroidal glycoalkaloid biosynthetic pathway. Gradient line arrows indicate steps with multiple enzymatic reactions. Solid fill arrows represent specific reactions with the abbreviation of the gene catalyzing the reaction indicated in the adjacent box in italic. *HMGR*, 3-hydroxy-3-methylglutaryl coenzyme A reductase; *SQS*, squalene synthase; *SQE*, squalene epoxidase; *LAS*, lanosterol synthase; *CAS*, cycloartenol synthase; *SGT1*, solanidine galactosyltransferase; and *SGT2*, solanidine glucosyltransferase. Brassinosteroids and stigmasterol are the other products of sterol biosynthesis in addition to cholesterol.

In primary metabolism, 3-hydroxy-3-methylglutaryl coenzyme A reductase (HMGR) catalyzes the formation of mevalonic acid. *HMGR* is a family of genes in which some members are involved in biosynthesis of sterols and triterpenoids mainly for plant development as is the case of *HMG1*, whereas others, including *HMG2*, are involved in the production of defense compounds (Suzuki and Muranaka 2007). In potato, where three genes have been characterized in this family, *HMG1* has been associated with SGA accumulation after wounding, whereas *HMG2* and *HMG3* transcripts have been up-regulated after wounding and pathogen inoculation and associated with increased sesquiterpenoid production (Choi *et al.* 1992, 1994; Krits *et al.* 2007). Downstream in this primary pathway, squalene synthase (SQS) condenses two farnesyl diphosphate molecules to form squalene. Increased transcription of *SQS* has been associated with high SGA levels in potato species (Yoshioka *et al.* 1999; Krits *et al.* 2007; Ginzberg *et al.* 2009). Squalene epoxidase (SQE) mediates the epoxidation of squalene to 2.3-oxidosqualene. Inhibition of this enzyme decreased sterol levels in tobacco *Nicotiana tabacum* cv. Bright Yellow-2 suspension cells (Wentzinger *et al.* 2002). A family of seven *SQE* genes has been reported in Arabidopsis, which may imply a more regulated role of this enzyme in sterol biosynthesis (Suzuki and Muranaka 2007). Cycloartenol synthase converts 2.3-oxidosqualene into cycloartenol, which is used to produce cholesterol, campesterol, and sitosterol, the main sterols found in plants (Schaller 2004; Ginzberg *et al.* 2009).

A second segment of SGA biosynthesis is the conversion of cholesterol to SGAs, which is considered secondary metabolism, involving enzymes that are unique to liliaceous and solanaceous species that produce SGAs (Heftmann 1983). Reactions that convert cholesterol into solanidine have been proposed (Kaneko *et al.* 1977), but the enzymes involved have not been identified (Ginzberg *et al.* 2009). Solanidine is the precursor of α-chaconine and α-solanine, the most common SGAs in potato species (Tschesche and Hulpke 1967; Canonica *et al.* 1977; Heftmann 1983, cited by Arnqvist 2007). Glycosyltransferase enzymes catalyze the final reactions in the biosynthesis of SGAs, where different sugar moieties are added to the solanidine aglycone. Three genes have been identified in potato encoding: solanidine galactosyltransferase (*SGT1*), solanidine glucosyltransferase (*SGT2*), and rhamnosyltransferase (*SGT3*) (Moehs *et al.* 1997; McCue *et al.* 2005, 2007a,b). *SGT1* and *SGT2* initiate the glycosylation of solanidine to γ-solanine and γ-chaconine, respectively, and *SGT3* catalyzes the conversion to α-solanine and α-chaconine.

Genetic studies of wild and cultivated potato species have concluded that multiple genetic factors interact for the production of SGAs (Sanford and Sinden 1972; Sanford *et al.* 1996; Yencho *et al.* 1998; Sørensen *et al.* 2008). The objective of this work was to analyze the nucleotide diversity of coding region fragments of five candidate genes involved in biosynthesis of SGAs (*HMG1*, *HMG2*, *SQE*, *SGT1*, and *SGT2*) in six wild potato species with different levels of SGAs (*Solanum chacoense*, *S. commersonii* subsp. *commersonii*, *S. demissum*, *S. sparsipilum*, *S. spegazzinii*, *S. stoloniferum*) compared with the cultivated *S. tuberosum* Group Phureja, to identify patterns of variation and their possible association with the synthesis and accumulation of SGAs. We assessed levels of genetic variation and single-nucleotide polymorphisms (SNPs) in SGA candidate genes to determine their possible role in governing accumulation of SGAs. A whole-genome SNP genotyping analysis was performed to identify new genomic regions putatively associated with the accumulation of these compounds in potato species.

## Materials and Methods

### Plant material

Two accessions with contrasting SGA content (based on SGA data available in the National Research Project 6-NRSP-6 United States Potato Genebank) were selected for five wild potato species from the NRSP-6 collection (Table 1). These include *S. commersonii* subsp. *commersonii*, *S. demissum*, *S. sparsipilum*, *S. spegazzinii*, and *S. stoloniferum*. In addition, a single clone each of *S. chacoense* USDA 8380-1 (*chc* 80-1) and *S. tuberosum* Group Phureja DH OT-B × N-B P5/AR2 (*phu* DH) were used. Sinden *et al.* (1980) reported high levels of accumulation of SGAs in the leaves of *chc* 80-1, and the production of leptines associated with resistance to Colorado potato beetle (*Leptinotarsa decemlineata* Say). By contrast, *phu* DH with low levels of SGAs is a dihaploid derived from an intermonoploid somatic hybrid (Veilleux 1990; Johnson *et al.* 2001; Lightbourn and Veilleux 2007). Instead of the *chc* 80-1 clone, its monoploid line *chc* 80 1-4 was used in the SolCAP 8303 Illumina Infinium potato SNP chip analysis. Short names were assigned to each accession, the standard abbreviation for S*olanum* species based on Simmonds (1963) followed by a random number (Table 1).

**Table 1:** Selected accessions with contrasting levels of SGA

Accession No.	Species	Short Name*^a^*	Foliar SGA Level	Origin
PI 243503	*Solanum commersonii* subsp. *commersonii*	*cmm* (7)	Very high	Argentina
PI 320266	*Solanum commersonii* subsp. *commersonii*	*cmm* (26)	Low	Uruguay
PI 347760	*Solanum demissum*	*dms* (54)	Very low	Mexico
PI 186562	*Solanum demissum*	*dms* (78)	Very high	Mexico
PI 311000	*Solanum sparsipilum*	*spl* (16)	Very low	Peru
PI 473373	*Solanum sparsipilum*	*spl* (81)	High	Bolivia
PI 458334	*Solanum spegazzinii*	*spg* (55)	Very low	Argentina
PI 205394	*Solanum spegazzinii*	*spg* (74)	High	Argentina
PI 184773	*Solanum stoloniferum*	*sto* (40)	Very high	Mexico
PI 243458	*Solanum stoloniferum*	*sto* (61)	Very low	Mexico
	*Solanum phureja*	*phu* DH	Very low	
PI 458310	*Solanum chacoense*	*chc* 80-1	Very High	Argentina

Accession number, SGA level, and origin information from NRSP-6 U.S. Potato Genebank, the SGA level for the last two accessions by HPLC analysis in our laboratory. SGA, steroidal glycoalkaloid.

a Short name assigned to each accession based on standard abbreviations for potato species and in parenthesis a random number.

Five seeds per accession were treated with 1000 mg/L GA_3_ solution overnight, and then sown in cell packs with Fafard super fine germination mix (Farfard, Agawan, MA). A single seedling per accession was transferred to D-40 Deepots (Hummert International, Earth City, MO) with Premier Horticulture Pro-mix BX 15 days after sowing. Simultaneously, after 1 wk of acclimation to growth in soil, *in vitro* grown plants of *chc* 80-1 and *phu* DH clones were transferred to D-40 Deepots. The plants were grown under controlled environment (Conviron, Winnipeg) set to 60% relative humidity, 14-hr photoperiod, 250 µmol/m^2^/s light intensity and day/night temperatures of 20/16°. Plants were fertilized with MiracleGro All Purpose (Scotts Co, Marysville, OH) at the rate of 1 g/L every 15 d. Three biological replications were established per individual plant from each accession. At 15-d intervals serial apical cuttings were taken to generate the three temporal replications planted into D-40 Deepots from 1 month-old established plants. At 55 days after cutting, leaves 4−6 from the shoot apex were harvested for SGA extraction. Young leaves were collected for DNA extraction from the starter plants.

### DNA extraction and polymerase chain reaction (PCR) amplification

Genomic DNA was extracted from leaf tissue of 12 accessions by a modified cetyltrimethylammonium bromide protocol (Murray and Thompson 1980; Doyle and Doyle 1987; Stewart and Via 1993). Homologous nucleotide sequences for each candidate gene from various solanaceous species were obtained from the GenBank database between 2008 and 2010. These sequences were aligned to design primers in conserved coding regions (Table 2). For *HMG1* the sequence from the draft genome of DM 1-3 516 *S. tuberosum* Group Phureja potato was also used (The Potato Genome Sequencing Consortium 2011). A DNA sequence within scaffold PGSC0003DMS000003141 had the greatest similarity score in a BLAST search with LO1400, the HMG1 GenBank sequence of *S. tuberosum*. For *HMG1* (PGSC0003DMG400013663, 35185, and 46343 gene models), *SGT1* (PGSC0003DMG400011749), and *SGT2* (PGSC0003DMG400017508), the fragment was located exclusively in a single exonic region, whereas the PCR products for *HMG2* (PGSC0003DMG400003461) and *SQE* (PGSC0003DMG400004923) captured some intronic sequence. The amplified region of *HMG1* coded for a 292 amino acid (aa) sequence with a catalytic and tetramerization interface domains. For *HMG2* the fragment covered 134 aa with catalytic, NADP(H) binding, substrate binding, and tetramerization residues. The 134-aa segment of *SQE* spanned more than one domain of its gene family. A region of 277 aa with a glycosyltransferase multidomain was amplified for *SGT1*, and a segment encoding 189 aa with active site conserved domains and thymidine diphosphate-binding site residues was isolated for *SGT2*.

**Table 2:** GenBank accessions used for primer design and primer sequences used to amplify fragments of five candidate genes

Gene	Linkage Group	GenBank Accessions	Primer Sequence	Product Size, bp	*T*_m_°
*HMG1*	11	L01400, AF110383, U60452, L40938, U51985	f- CGACCTGTTAAGCCTCTATACAC	877	60
			r- GCCACCAGAGACAAAGATAGCCT		
*HMG2*	2	M63642, AF110383, AB041031, U51985-6, L01400, AF110383	f- TGGTGTCCAAAGGTGTACAAAATG	608	59
			r- ACAGAAATATGGAGGTCCTTGCC		
*SQE*	4	AY995182, BG123494, CU915722	f- TGGGGTTCGTTGCAGTTTTC	884	60
			r- CAGGGGATAAGAAAGACGTGTACTC		
*SGT1*	7	U82367, DQ218276, DQ218277, DQ266437, AK323113, AB182385	f- TCCCTTGGACAGTAGATATTGCTG	834	58
			r- TTCCCAATCCCCTAACCTCG		
*SGT2*	8	U82367, DQ218276, DQ218277, DQ266437, AK323113, AB182385	f- CCTGCGGATGAGAGGAATGC	567	64
			r- CACCAACGGCACCCCAGCG		

Primer direction: forward (f) and reverse (r). Melting temperature of primers = Tm. Linkage group correspond with the location of these gene sequences at potato genome.

The primers were designed using DNASTAR Lasergene 9 core suite software for sequence analysis and assembly. PCR was performed in 25 µL of 1× *Ex Taq* polymerase buffer, 0.2 mM of each dNTP, 0.24 µM of each primer, and 0.2 units of high-fidelity TaKaRa *Ex Taq* DNA polymerase (Takara Biotechnology, Shiga, Japan) and 100 ng of genomic DNA template. Standard cycling conditions were 5 min initial denaturation at 95° followed by 30 cycles of 0.5 min at 94°, 0.5 min annealing at the appropriate *T*_m_, and a 2-min extension time (Table 2). The reactions were finished by a 5-min incubation at 72°. Reconditioning PCR conditions were used for *HMG1* and *SGT1* to avoid recombinant PCR products (Judo *et al.* 1998; Thompson *et al.* 2002; Lenz and Becker 2008). The final PCR product was derived from two PCRs of 20 cycles each, where 2 µL of product from the first reaction were used as a template in the second reaction.

### Identification of allelic sequences

PCR products were gel-purified with QIAquick Gel Extraction Kit (QIAGEN, Hilden, Germany) and cloned into pJET1.2/blunt vector (CloneJET PCR Cloning Kit, Fermentas, Thermo Fisher Scientific Inc., Waltham, MA) using JM109 competent cells (Promega, Madison, WI). At least five colonies from each cloning reaction were sequenced with the use of both forward and reverse primers (Virginia Bioinformatics Institute Core Facility at Virginia Tech, Blacksburg, VA). Forward and reverse sequences (File S1) were aligned to identify consensus sequences. Sequencing errors were corrected by visual inspection of peak quality in chromatograms. The set of consensus sequences per accession was aligned to identify alleles. A minimum alignment of at least three identical consensus sequences defined an allelic sequence from the pool of sequences. In some cases, additional sequence errors were detected when consensus sequences following the same pattern were aligned. These were corrected, and the amended sequences were used for identification of allelic sequences. Sequence alignments and editing were done using the different applications included in the DNASTAR Lasergene 9 core suite software for sequence analysis and assembly and Sequence Scanner Software v1.0 from Applied Biosystems.

### Sequence polymorphism and diversity analysis

Sequence polymorphisms were analyzed using the DNA Sequence Polymorphism (DnaSP5) software version 5.0 (Librado and Rozas 2009) and Molecular Evolutionary Genetics Analysis (MEGA5) software version 5.0 (Tamura *et al.* 2011). The ratio of nonsynonymous (dN) and synonymous (dS) substitution rates was calculated per gene using CODEML software from Phylogenetic Analysis by Maximum Likelihood version 4.5 (PAML4.5) package of programs (Yang 2007). This ratio provides insights of selective pressures acting on protein-coding regions and allows identifying positive selection (dN/dS > 1) or purifying selection (dN/dS < 1). Likelihood analyses were calculated on six codon substitution site models implemented in CODEML. The difference between two models was estimated by likelihood ratio test statistics, twice the log likelihood difference between the two compared models (2Δl) compared against χ^2^ distribution. Multiple allelic sequences of each candidate gene were aligned by the CLUSTAL W method (Thompson *et al.* 1994) using MEGA5. Then a phylogenetic tree was constructed per gene according to maximum likelihood method in the analysis interface of MEGA5.

The alignments and phylogenetic trees were used for analyses in MEGA5, DnaSP5, and PAML4.5. Besides the allelic sequences identified per gene in the wild potato species selected for this study, available GenBank cDNA sequences of *S. tuberosum* and genomic sequences from the sequenced potato genome *S. tuberosum* Group Phureja DM 1-3 516 R44 were added to the analysis of each gene. Nucleotide diversity for the entire population per gene was calculated using Tajima-Nei’s π estimator (Tajima and Nei 1984) using MEGA5. π is the average number of nucleotide differences per site between two homologous sequences. MEGA5 calculates π as the average of the values for all pairwise comparisons. The deviation of nucleotide variation patterns from the neutral theory of molecular evolution (Kimura 1983), that assumes all mutations in a DNA region are selectively neutral, was tested by Tajima’s D statistical analysis using MEGA5. The Tajima’s D test compares whether π and θ values are significantly different (Tajima 1989). Exon delimitations were assigned in MEGA5 and DnaSP5 following GenBank cDNA sequences as a pattern. Numbers of synonymous and nonsynonymous substitutions were estimated using DnaSP5 conservative criteria. In some complex cases with sites segregating for several codons (highly variable regions), the synonymous and nonsynonymous substitutions were estimated manually.

### SNP chip analysis

A whole-genome SNP genotyping using the SolCAP 8303 Illumina Infinium potato SNP chip was performed on our 12 accessions alongside the *chc* 80 1-4 monoploid clone (Hamilton *et al.* 2011). The SNP genotyping facility at Michigan State University processed the genomic DNA samples on an Illumina iScan Reader utilizing the Infinium HD Assay Ultra (Illumina, Inc., San Diego, CA) and the SolCAP 8303 Illumina SNP Infinium Potato genotyping Array. The 8303 SNP data were filtered and used for analysis of variance (ANOVA) with the average of total SGA accumulation amounts per accession. SNPs that were monomorphic for all individuals, and SNPs with a no-call rate >25% (greater than three samples with missing genotypes) were eliminated from the initial data set. From 5392 analyzed SNPs, significant ones were selected based on R^2^ > 0.2, *P* < 0.05, minimum of two samples per SNP genotype, and minimum difference of 5 logarithm units between the means of the most significant different SNP genotypes, and SNPs with superscaffold and pseudomolecule information on the potato genome browser (http://solanaceae.plantbiology.msu.edu/cgi-bin/gbrowse/potato/). Significant SNPs were used for cluster analysis to identify informative SNPs (SNPs putatively associated with SGA accumulation). The physical position in the potato genome of informative SNPs was obtained from Felcher *et al.* (2012).

### SGA extraction and quantification

Leaf tissue harvested from each of three biological replications of the 12 accessions was freeze-dried for 72 hr in a lyophilizer (Labconco, Kansas City, MO), then ground and SGAs extracted using a modified procedure (Edwards and Cobb 1996). An initial extract, from 30 mg of leaf powder mixed by vortex with 1 mL of extraction buffer (5% v/v acetic acid, 0.02 M heptane sulfonic acid) was ultrasonicated for 2 sec (Digital sonifier cell disruptor, Branson Ultrasonic Corporation, NY, with 20% amplitude). The extract was set on a microtube thermal-mixer for 15 min at 0.51 × g, centrifuged 3−5 min at 16,110 × g, and a clear solution recovered after it was sieved in a 50-µm filter plate. The precipitate was used for a second cycle of extraction. SGAs were concentrated and purified by solid-phase extraction using Sep-Pak Classic C18 cartridge columns (Waters, Milford, MA). Five milliliters of methanol (MeOH) followed by 5 mL of extraction buffer were added to the columns to activate and equilibrate them. Then, the leaf extract was applied followed by a sequence of washes: 7.5 mL of water, 5 mL of 50 mM ammonium bicarbonate (NH_4_ HCO_3_), 5 mL of 50 mM NH_4_ HCO_3_:MeOH (1:1 v/v), and 7.5 mL of water. The final SGA extract was eluted with 1.2 mL of elution buffer (80% v/v MeOH, 0.5% v/v formate).

A high-performance liquid chromatography (Agilent HP 1200 Series, Santa Clara, CA) on a C-18 reverse-phase column (Agilent Eclipse XDB-C18, 5-µm pore size and 4.6 × 150 mm) procedure was used to separate the SGAs, and a photodiode array detector to quantified them. Elution of SGAs was attained by using a binary gradient system consisting of Solvent A (30% acetonitrile, 6 mM Tris-HCl, pH 8.0) and Solvent B (80% acetonitrile, 6 mM Tris-HCl, pH 7.6) at a flow rate of 0.3 mL/min at 25° column temperature. The gradient elution was: 0−0.5 min, 0% B; 0.5−8.5 min, 0–30% B; 8.5−12 min, 30–100% B; 12−16 min, 100% B; 16−16.5 min, 100–0% B; and 16.5−21 min, 0% B. Eluent was monitored at 202 nm. Purified standards solutions of α-solanine and α-chaconine (Sigma-Aldrich) were used to generate calibration curves. Peak absorbance area at A_202nm_ was used to quantify the different SGAs detected and separated in a frame of retention time between 5 and 15 minu. α-Solanine and α-chaconine standard solutions were used as reference peaks. Separation of the plant extracts produced peaks associated with SGA compounds specific of each wild species. The different peaks detected in each sample were added to calculate the total amount of SGAs per sample. A unique quantification unit of mAU of SGAs per mg of dry weight leaf tissue (mAU/mg DW) was used for further analysis.

### Statistical analysis

A completely randomized design was used for plants in the growth chamber and for SGA analysis. The amount of total SGAs was estimated in leaf tissue collected from each biological replication per accession. Analyses of variance were conducted for the total SGA response variable using as source either accessions or allelic states of polymorphic SNPs within candidate genes and the SNP array. The average of repetitions was used for statistical analysis of SNPs. SGA data were transformed with logarithm for all analyses. All statistical analyses were done using JMP 9 (SAS Institute Inc., Cary, NC).

### Protein modeling

The *SGT2* aa sequence of *S. tuberosum* (GenBank accession ABB29874) was used to model the structural effects of nonsynonymous and indel polymorphisms of *chc* 80-1 and *phu* DH allelic fragments. The amplified fragment spans 189 aa from positions 197 to 385 in the 482 aa sequence of *SGT2*. The original 189 aa fragment of *S. tuberosum* occurring in the position of our amplicon was substituted by either *chc* 80-1 or *phu* DH fragments and used for protein modeling. Both aa sequences were submitted to the Interactive Threading Assembly Refinement Algorithm I-TASSER server http://zhanglab.ccmb.med.umich.edu/I-TASSER/ (Zhang 2008). The output pdb structure file was manipulated and visualized using PyMOL Molecular Graphic System, Version 1.5.0.4 Schrödinger, LLC.

## Results

### Allelic sequences and sequence polymorphisms

Sequences of genomic DNA fragments corresponding to conserved coding regions of five candidate genes were obtained by PCR amplification, cloning, sequencing, and sequence analysis. One or two allelic sequences per gene were detected per individual since the accessions derived from diploid heterozygous species (Table 3). In some cases a single allelic sequence was shared by accessions from the same species or different species. The total number of unique allelic sequences identified was 66, with a range of 9−17 unique sequences per gene. Sequence polymorphism analysis among the allelic sequences per gene detected a total of 337 variable sites (Table 4). The SNP sites produced 354 SNP mutations, of which 35% were nonsynonymous, and 65% were silent (synonymous or in noncoding regions). The frequency of transitions *vs.* transversions was greater for *HMG1* (3.8) and similar for all other genes (2.0−2.4). Genes involved in primary metabolism, *HMG1*, *HMG2* and *SQE*, had fewer SNPs in exon regions, 0.03, 0.05, and 0.05 SNPs/bp respectively, than those involved in secondary metabolism, *SGT1* and *SGT2*, with 0.13 and 0.10 SNPs/bp, respectively. However, *HMG2* and *SQE* fragments spanning regions with one and two introns that had the greatest SNP rates, 0.26 and 0.13 SNPs/bp, for each gene. Indels with 1−3 codon deletions were found in *HMG1*, *SGT1*, and *SGT2*, and various sized indels were identified in introns of *HMG2* and *SQE*. Codons with 2-3 SNP sites and several variants in each were detected in *SGT1* and *SGT2*; such variable codons would be expected to yield proteins with highly variable amino acids in the position.

**Table 3:** Number of allelic sequences identified in six wild and one cultivated potato species for five candidate genes within the glycoalkaloid biosynthetic pathway

Gene	Total Unique Allelic Sequences per Locus	*cmm*	*dms*	*spg*	*spl*	*sto*	*chc*	*phu*
*HMG1*	9	2	2	2	2*^a^*	2*^a^*	0	0
*HMG2*	12	1	2*^a^*	3*^a^*	2	2*^a^*	2	2
*SQE*	16	3	2	2	3	3	2	1
*SGT1*	17	1	4*^a^*	4	3	2*^a^*	2	2
*SGT2*	12	1	1*^a^*	4	3	2*^a^*	1	1
Total	66	8	11	15	15	11	9	8

a These wild species share one identical allelic sequence. Potato species listed using standard abbreviations for potato species.

**Table 4:** Summary of polymorphic sites and SNPs discovered in five candidate genes in the glycoalkaloid biosynthetic pathway

Gene	N	Total sites, bp*^a^*	Total SNP sites, S	Total No. Variations	SNPs in Noncoding Regions	Synonymous SNPs	Nonsynonymous SNPs	Transitions/ Transversion	Indels (Location)
*HMG1*	22	876	22	22	0	12	10	3.8	1 (Exon)
*HMG2*	28	571	65	65	44	14	7	2.4	3 (Intron)
*SQE*	26	887	85	89	66	13	10	2.0	10 (Intron)
*SGT1*	28	834	106	113	0	49	64	2.3	1 (Exon)
*SGT2*	27	567	59	65	0	31	34	2.0	2 (Exon)
Average	26.2	754.2	67.4	70.8	22	23.8	25	2.5	3.4
Total	131	3,771	337	354	110	119	125		17

SNP, single-nucleotide polymorphism; N, total number of analyzed sequences.

a Excluding sites with gaps on the total alignment.

### Sequence diversity

Codon and nucleotide diversity were estimated for the pool of sequences of each gene (Table 5). In the codon based analysis, the dN/dS ratios were less than one in all gene fragments. The likelihood ratio tests between unconstrained (M0) and constrained (M3) analysis were not significant in all cases; thus accepting the M0 null hypothesis of no evidence of selective constraints or adaptive evolution acting on those gene sequences. Tajima’s test of neutrality was computed at the nucleotide level, where we found that all gene fragments had non-significant D-statistics values. This analysis confirms no evidence for natural selection at these genetic regions. A significant relationship between pathway position and the estimates of divergence (dN, dS and dN/dS ratios) was found (n = 5, *r*^2^ = 0.96, *P* < 0.003*; n = 5, *r*^2^ = 0.85, *P* < 0.026; and n = 5, *r*^2^ = 0.77, *P* < 0.049*), with smaller ratios (0.29, 0.15, and 0.24) in the genes of primary metabolism (*HMG1*, *HMG2*, and *SQE*) than in those (0.42 and 0.39) of secondary metabolism (*SGT1* and *SGT2*) respectively. The nucleotide diversity estimations followed the same pattern for exon regions, and increased greatly for *HMG2* and *SQE* when introns were taken into account for total estimations. Positions of the candidate genes in the metabolic pathway (1 = *HMG1* and *HMG2*, 2= *SQE*, and 4= *SGT1* and *SGT2*), for regression analysis, were assigned based on the distance (in the number of reactions) between these genes. At least seven reactions (1 unit) could occur between *HMG1*/*HMG2* and *SQE* in the putative pathway of sterol biosynthesis proposed by Suzuki and Muranaka (2007). In the same way, there are around 13 reactions (2 units) from *SQE* to *SGT1*/*SGT1* using as reference the putative metabolic pathway of cholesterol suggested by Arnqvist (2007) and the three minimal reactions from cholesterol to solanidine aglycone proposed by (Kaneko *et al.* 1977).

**Table 5:** Estimates of codon and nucleotide diversity of sequences of segments in coding regions for five candidate genes in wild potato species

Gene	N	dN	dS	dN/dS	(2Δl) M0 *vs.* M3 (df = 4)	π Exons	π Total	D-statistics
*HMG1*	22	0.02	0.06	0.29	−8.14 NS	0.005	0.005	−1.0329 NS
*HMG2*	28	0.02	0.15	0.15	0.00 NS	0.015	0.032	−0.0561 NS
*SQE*	26	0.03	0.14	0.24	0.00 NS	0.013	0.022	−0.8299 NS
*SGT1*	28	0.12	0.28	0.42	−22.91 NS	0.020	0.020	−1.5507 NS
*SGT2*	27	0.10	0.27	0.39	−18.90 NS	0.029	0.029	0.2592 NS

N, Total number of analyzed sequences; dN, nonsynonymous; dS, synonymous; π, nucleotide diversity; NS, not significant.

### SGA accumulation and association with SNPs at candidate genes

Total SGA levels in leaf tissue of the 12 accessions were determined. Between 0 and 4 different SGA compounds were quantified per sample and added to calculate total amount of SGA per sample in mAU/mg DW. ANOVA analysis of total SGAs indicated a statistically significant difference in the SGA accumulation level among accessions (*P* = 0.0023 for nonparametric test and *P* < 0.0001 in normal ANOVA). For the most part, the seedlings selected randomly from paired accessions within each species expected to be low or high for SGAs based on the data available from NRSP-6 differed dramatically from each other with regard to total SGAs (Table 6). Only the two *dms* accessions did not differ significantly. The high selections ranged from 5966 to 55.611 mAU/mg DW with *dms* 78 significantly different from the other five. The low selections ranged from 0 to 5857 mAU/mg DW with *phu* DH significantly different from the other five. There was some overlap between the lowest three (*dms* 78, *cmm* 26, and *sto* 40) of the high selections and the highest three (*cmm* 7, *dms* 54, and *spl* 16) of the low selections (Table 6). Regardless of the selection within species, the overall distribution of SGA levels created three groups: low (accessions grouped in e and f mean separation categories), intermediate (in d), and high (classified as a and b).

**Table 6:** Total SGA accumulation in leaf tissue of 12 accessions determined by HPLC

Low Selection	N	Mean (mAU/mg DW)	SD	High Selection	n	Mean (mAU/mg DW)	SD	Difference
*cmm* 7	1	2619 de		*cmm* 26	1	25,302 a,b,c		22,683
*dms* 54	3	4013 d	1689	*dms* 78	3	5,966 d	1,689	1,953
*spg* 55	3	1360 e	2230	*spg* 74	3	19,892 a,b	2,807	18,532
*spl* 16	3	5857 cd	792	*spl* 81	3	42,095 a,b	26,037	36,238
*sto* 61	3	1290 e	283	*sto* 40	3	16,966 b,c	5,964	15,676
*phu* DH	3	0 f	0	*chc* 80-1	3	55,611 a	4,937	

The accessions were named using the species abbreviation and a random number assigned in this study. The amount of accumulation of SGAs is in milliabsorbance units (mAU) of compound per mg of dried weight leaf tissue (DW). Means followed by the same letter are not significantly different at 0.01 α level using Student’s *t* mean separation analysis. SGA, steroidal glycoalkaloid; HPLC, high-performance liquid chromatography; n, number of biological repetition per accession.

**Figure 2:**
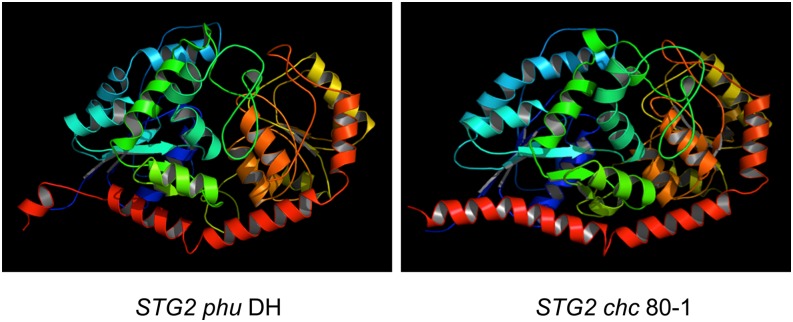
Predicted protein structure modifications resulting from amino acid changes and indel polymorphisms in *phu* DH and *chc* 80-1 allelic sequences of *SGT2*. The three aa indel in *phu* DH in the red colloidal is the most evident modifications beside the other four aa changes that differentiate both sequences.

To determine potential associations between informative SNPs and SGA accumulation in the potato accessions, we conducted ANOVAs on the logarithm of averages of total SGA levels estimated per accession using allelic variants for SNPs identified in exons of candidate gene fragments as the source of variation (Table 7). Then haplotype clusters were built per SNP genotype, their accessions and total SGA levels, to identify clusters holding mainly low or high SGA accessions within a single SNP genotype. Since the significant SNPs identified by ANOVA were the most likely to be informative, we analyzed them first and used their pattern to identify other informative SNPs. For *HMG1*, 19 polymorphic SNPs were in our potato species, none of which was statistically significant for association with SGA accumulation, nor was there a SNP genotype cluster associated with high or low SGA accessions. From the 20 polymorphic SNPs in *HMG2* a set of three nonsynonymous SNPs could explain different levels of SGA accumulation. HMG2_snp_202 was an informative SNP identified by ANOVA (*P* < 0.001*), which separated *phu* DH, which did not produce SGAs from the other individuals. The different alleles of HMG2_snp_202 would be expected to code for either serine, a polar amino acid, or alanine, a nonpolar amino acid. HMG2_snp_128 and 199 separated the highest SGA producer, *chc* 80-1, from the other samples, with codons specifying either arginine/lysine (both polar) or alanine/serine, with different polarity. One SNP (SQE_snp_220) of 22 that were polymorphic in *SQE* was potentially associated with SGA accumulation (*P* < 0.001*). This SNP is nonsynonymous, specifying a change from lysine to glutamine, both polar amino acids, and separated *phu* DH from the other samples. *SGT1* had 106 polymorphic SNPs and for 11 the SGA accumulation levels showed significant differences among SNP genotypes with *P* < 0.006. Seven were nonsynonymous, specifying amino acids with similar polarity, and four were synonymous. The 11 SNPs (SGT1_snp_171, 210, 249, 250, 255, 408, 415, 435, 612, 666, and 714) were mainly heterozygous in *phu* DH and homozygous for all other individuals, with the exception of two that were also heterozygous in 1-2 other individuals. Three SNPs (SGT1_snp_210, 256, and 549) that were heterozygous for the greatest SGA producer, *chc* 80-1, separated it from all other individuals. SNP SGT1_snp_210 was detected by ANOVA, whereas the other two were found during the cluster analysis. All of them were nonsynonymous, specifying amino acids changes of similar polarity, lysine by asparagine, aspartic acid by glutamic acid and different polarity, and glycine by arginine (SGT1_snp_256). In *SGT2* there were 53 polymorphic SNPs, and seven were putatively associated with SGA accumulation. We observed only two homozygous genotypes for six of these SNPs in our germplasm panel, whereas there were four possible different homozygotes for SGT2_snp_264. Four of these SNPs were nonsynonymous and three synonymous; two of the nonsynonymous changed from polar to non-polar and the other two kept same the polarity. Four SNPs (SGT2_snp_126, 264, 396 and 404) with an exclusive genotype for *phu* DH separated it from other accessions. These SNPs were detected by ANOVA, two were synonymous and two nonsynonymous specifying changes of glutamine by histidine and serine by leucine. Four SNPs (SGT2_snp_10, 11, 76 and 404) exhibited a specific genotype for *chc* 80-1. Two produced amino acid changes in *chc* 80-1 from glutamic acid to tryptophan and valine to isoleucine, and two were synonymous. A comparison of protein models of *SGT2* using either the *chc* or *phu* amplicons to specify aa from positions 197-385 and the *S. tuberosum* aa profile to complete the 482 aa protein yielded models with different secondary structure (Figure 2). In general the SNPs at candidate genes did not cluster accessions with similar levels of SGA accumulation, but were able to discriminate no synthesis in *phu* DH and the greatest accumulator of SGAs, *chc* 80-1. Some of these informative SNPs can be used as tag SNPs to screen segregating populations.

**Table 7:** Informative SNPs found within exons of candidate genes putatively associated with SGA accumulation in leaf tissue of potato species

	SNP Genotypes				Number of Samples per Genotype				Mean per Genotype (Logarithmic Units)				
SNP_ID	G1	G2	G3	G4	*n*1	*n*2	*n*3	*n*4	Mean 1	Mean 2	Mean 3	Mean 4	Type of SNP
HMG2_snp_128	GG	AG			11	1			8	10.9			Nonsyn (R/K)
HMG2_snp_199	GG	TT			11	1			8	10.9			Nonsyn (A/S)
HMG2_snp_202	GG	GT			11	1			9	0			Nonsyn (S/A)
SQE_snp_220	AA	CC			11	1			9	0			Nonsyn (K/Q)
SGT1_snp_171	AA	GA			11	1			9	0			Nonsyn (I/M)
SGT1_snp_210	AG	GG	GT		1	10	1		0	8.8	10.9		Nonsyn (K/N/R)
SGT1_snp_249	AA	TA			11	1			9	0			Nonsyn (E/K/D)
SGT1_snp_250	AC	CC			1	11			0	9			Nonsyn (Q/K)
SGT1_snp_255	AT	TT			1	11			0	9			Nonsyn (V/I)
SGT1_snp_256	AG	CC	GC	GG	1	1	3	7	10.9	7.2	5.6	9.2	Nonsyn (A/G/R)
SGT1_snp_408	AA	GA			11	1			9	0			Syn
SGT1_snp_415	GT	TA	TT		1	2	9		0	8.5	9.2		Nonsyn (S/T/A)
SGT1_snp_535	GG	TG	TT		10	1	1		9.2	0	7.2		Nonsyn (A/S/D)
SGT1_snp_549	TA	TG	TT		1	1	10		10.9	7.2	8.1		Nonsyn (D/E/N)
SGT1_snp_612	AA	AC			11	1			9	0			Syn
SGT1_snp_666	CC	TT			11	1			9	0			Syn
SGT1_snp_714	TT	AA			11	1			9	0			Syn
SGT2_spn_10	GG	TT			11	1			8	10.9			Syn
SGT2_spn_11	AA	GG			11	1			8	10.9			Nonsyn (E/W)
SGT2_spn_76	AA	GG			1	11			10.9	8			Nonsyn (V/I)
SGT2_spn_126	AA	GG			1	11			0	9			Syn
SGT2_spn_264	AA	CC	GG	TT	2	1	2	7	9	0	9.1	9	Nonsyn (Q/H)
SGT2_spn_396	AA	CC			11	1			9	0			Nonsyn (S/L)
SGT2_spn_404	–	CC	TT		1	10	1		0	8.8	10.9		Syn

SNP ID, candidate gene and bp position in the sequenced fragment. SNP genotypes = G1−G4, number of samples per SNP genotype = *n*1−*n*4, and mean per SNP genotype in logarithmic units = Mean 1−4. Syn, synonymous; Nonsyn, nonsynonymous. In parentheses, the amino acid changes are shown in a standard amino acid abbreviation. SNP, single-nucleotide polymorphism.

### SNP chip analysis

**Figure 3:**
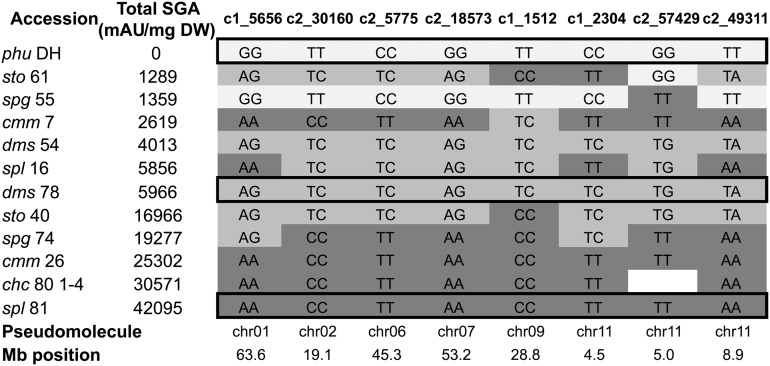
Haplotypes of informative SNPs identified in a whole-genome analysis of wild potato species with different levels of SGA accumulation. Accessions are listed from low to high levels of accumulation. The bottom lines show the pseudochromosome and physical position of informative SNPs in the published potato genome sequence. The three boxes indicate putatively low, intermediate, and high SGA accumulation haplotypes.

**Figure 4:**
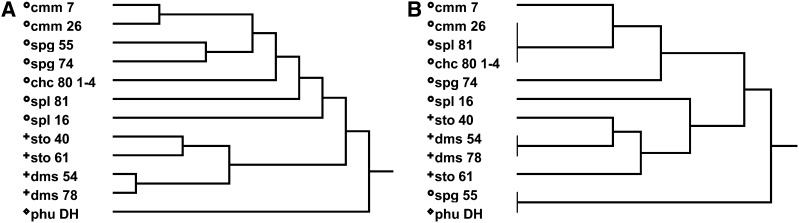
Comparison of hierarchical clusters based on 3841 polymorphic SNPs (A) and eight SGA informative SNPs (B) from whole genome analysis of germplasm panel. A neighbor-joining phylogenetic tree built in TASSEL classified species and accessions with similar patterns as A. Marks on the left of species name corresponded to defined clusters. Most of the accessions with high SGA levels cluster on big dots, intermediate levels on small dots, and low on diamonds. However, *cmm* 7 and *sto* 61 with low SGA levels were not in the right cluster.

A whole genome SNP chip analysis was done on the twelve potato accessions to identify genomic regions putatively associated with SGA accumulation (Table S1). In this analysis, a monoploid line derived from *chc* 80 1−4 was used instead of *chc* 80−1 itself, and the ANOVA was used to identify the most likely SNPs associated with SGA accumulation. The stringency was increased to identify significant SNPs that clustered at least two accessions with low or high SGA levels, with mean difference between SNP clusters equal to or greater than 5 logarithm units (Table 8). Then the allelic structure of significant SNPs was analyzed by accessions to identify informative SNPs. Thirty-four significant SNPs located on 10 pseudochromosomes were initially associated with total SGA accumulation (Table 8). Cluster analysis identified eight informative SNPs on six pseudochromosomes with homozygous and heterozygous genotypes that discriminated high, intermediate and low levels of SGA accumulation (Figure 3). Two accessions, *phu* DH and *spg* 55, were mostly representative in the cluster of low SGA levels. Of four SNPs located on pseudochromosome 1, one (solcap_snp_c1_5656) at 63.6 Mb mainly clustered in two SNP genotypes (AA and AG) six accessions with the greatest accumulation of SGAs. Five SNPs were found on pseudochromosome 2, of which solcap_snp_c2_30160 at 19.1 Mb grouped in two SNP genotypes (CC and TC) eight accessions with the greatest levels of accumulation, with a gradual decrease from the four with CC to the four with TC. Pseudochromosomes 3, 4, and 5 with two, four and one SNPs did not group the accessions into any logical arrangement. Of five SNPs on pseudochromosome 6, solcap_snp_5775 at 45.3 Mb was informative in grouping eight accessions with the greatest levels of SGA accumulation in homozygous (CC) and heterozygous (TC) genotypes similar to the distribution by solcap_snp_c2_30160 on pseudochromosome 2. Solcap_snp_c2_18573 at 53.2 Mb of five SNPs on pseudochromosome 7 again grouped the 8 accessions with the greatest levels of SGAs under similar patterns. Solcap_snp_c1_1512 one of the two SNPs on pseudochromosome 9, at 28.8 Mb clustered nine accessions; the five with the greatest levels of SGAs had the CC allele whereas the four with lower levels had the TC genotype. The SNP on pseudochromosome 10 did not have any defined cluster associated with high or low SGA accessions. Finally, three informative SNPs were identified out of five on pseudochromosome 11 (solcap_snp_c1_2304, solcap_snp_c2_57429 and solcap_snp_c2_49311) between 4.5 and 8.9 Mb. These SNPs have at least three accessions with the greatest SGA level, three in intermediate levels and two with the lowest levels grouped in different SNP genotypes. The SNP on pseudochromosome 12 was not informative. The group of eight informative SNPs defined putative haplotypes for low, intermediate and high SGA accumulation (Figure 3). Comparison of hierarchical clustering trees, built in the statistical software JMP using 3841 SNPs with nonmissing data from the potato array and another with the eight informative SNPs, showed that the twelve accessions were grouped by species except for *spl* taxa in the first tree in contrast with the second where they were grouped by SGA levels (Figure 4). The neighbor-joining phylogenetic tree constructed in TASSEL 3.0 (Bradbury *et al.* 2007) (data not shown) using the 3841 SNPs followed the kinship between samples from the same species found in the analysis in JMP. *Phu* DH the only cultivated species in the germplasm panel was separated from all other samples. Three general clusters were generated based on informative SNPs that grouped by SGA levels for most of the accessions. Two accessions (*cmm* 7 and *sto* 61) with low levels of SGA were located in high and intermediate SGA cluster.

**Table 8:** Significant SNPs associated with SGA accumulation in a whole genome SNP chip analysis of wild and cultivated potato species

SNP_ID	*P*-value	R^2^	G1	G2	G3	*n*1	*n*2	*n*3	Mean 1	Mean 2	Mean 3	Mean Difference	Pseudomolecule	Mb Position
solcap_snp_c2_45058	0.023	0.568	AA	AC	CC	3	7	2	10.3	8.6	4.3	6	chr01	4.7
solcap_snp_c2_35520	0.023	0.568	CC	TC	TT	3	7	2	10.3	8.6	4.3	6	chr01	53.6
solcap_snp_c1_5656*^a^*	0.018	0.592	AA	AG	GG	5	5	2	9.5	8.8	4.0	6	chr01	63.6
solcap_snp_c2_19956	0.028	0.433	TA	TT		9	2		9.2	4.7		5	chr01	65.7
solcap_snp_c2_30160*^a^*	0.012	0.623	CC	TC	TT	5	5	2	9.8	8.5	4.0	6	chr02	19.1
solcap_snp_c2_41963	0.020	0.579	AA	AG	GG	6	4	2	9.3	8.9	4.0	5	chr02	26.2
solcap_snp_c2_17954	0.011	0.489	AA	GG		10	2		9.1	4.3		5	chr02	34.8
solcap_snp_c2_35212	0.005	0.595	CC	CG		9	2		9.3	4.0		5	chr02	36.3
solcap_snp_c1_2587	0.021	0.579	CC	TC	TT	6	4	2	9.3	8.9	4.0	5	chr02	40.6
solcap_snp_c2_20259	0.021	0.578	AA	AG	GG	3	7	2	9.4	9.0	4.0	5	chr03	20.9
solcap_snp_c2_29678	0.004	0.580	AG	GG		2	10		3.9	9.1		5	chr03	23.4
solcap_snp_c2_55709	0.004	0.573	CC	TC		10	2		9.1	4.0		5	chr04	19.4
solcap_snp_c2_45040	0.004	0.573	AG	GG		2	10		4.0	9.1		5	chr04	47.6
solcap_snp_c2_32550	0.009	0.593	AG	GG		2	8		3.9	9.2		5	chr04	54.4
solcap_snp_c2_34866	0.021	0.575	CC	TC	TT	2	3	7	4.0	9.0	9.2	5	chr04	60.1
solcap_snp_c2_11829	0.004	0.580	CC	TC		10	2		9.1	3.9		5	chr05	3.9
solcap_snp_c2_3108	0.021	0.575	GG	TG	TT	2	4	6	4.0	9.0	9.3	5	chr06	0.8
solcap_snp_c1_15371	0.011	0.489	CC	TT		10	2		9.1	4.3		5	chr06	41.8
solcap_snp_c2_5774	0.014	0.611	CC	TC	TT	6	4	2	9.6	8.5	4.0	6	chr06	45.3
solcap_snp_c2_5775*^a^*	0.012	0.623	CC	TC	TT	2	5	5	4.0	8.5	9.8	6	chr06	45.3
solcap_snp_c2_29216	0.018	0.588	AA	AG	GG	2	2	8	4.0	8.5	9.3	5	chr06	50.4
solcap_snp_c2_36831	0.018	0.480	AA	GG		9	2		9.0	4.3		5	chr07	2.6
solcap_snp_c2_26003	0.011	0.489	AT	TT		2	10		4.3	9.1		5	chr07	46.8
solcap_snp_c2_12405	0.004	0.573	AG	GG		2	10		4.0	9.1		5	chr07	49.3
solcap_snp_c2_28855	0.004	0.573	AA	AG		2	10		4.0	9.1		5	chr07	52.3
solcap_snp_c2_18573*^a^*	0.012	0.623	AA	AG	GG	5	5	2	9.8	8.5	4.0	6	chr07	53.2
solcap_snp_c1_4228	0.004	0.573	AG	GG		2	10		4.0	9.1		5	chr09	1.6
solcap_snp_c1_1512*^a^*	0.013	0.622	CC	TC	TT	6	4	2	9.6	8.4	4.0	6	chr09	28.8
solcap_snp_c2_44210	0.028	0.433	AG	GG		2	9		4.7	9.2		5	chr10	3.8
solcap_snp_c2_13350	0.042	0.505	CC	TC	TT	4	6	2	9.5	8.8	4.3	5	chr11	2.0
solcap_snp_c1_2304*^a^*	0.022	0.573	CC	TC	TT	2	4	6	4.0	9.2	9.1	5	chr11	4.5
solcap_snp_c2_57429*^a^*	0.032	0.577	GG	TG	TT	2	4	5	3.9	8.9	9.1	5	chr11	5.0
solcap_snp_c2_49311*^a^*	0.014	0.611	AA	TA	TT	6	4	2	9.6	8.5	4.0	6	chr11	8.9
solcap_snp_c2_32982	0.041	0.528	CC	GG		6	2		9.6	4.5		5	chr11	9.2

SNP ID, SolCAP 8303 Illumina Infinium potato SNP chip. SNP genotypes = G1−G3, number of samples per SNP genotype = *n*1−*n*3, and mean per SNP genotype in logarithmic units = Mean 1−3. Pseudomolecule and Mb position based on published potato genome sequence. SGA, steroidal glycoalkaloid; SNP, single-nucleotide polymorphism.

a Informative SNPs.

## Discussion

### Sequence polymorphisms and diversity

Each of the 12 potato accessions that was tested in the present project had one or two different genomic sequences within the amplified fragments of conserved coding regions of five candidate genes related to the SGA biosynthetic pathway. Analysis of polymorphisms in a total length of 3.7 Kb from segments of five candidate genes found 354 SNP variations. Nucleotide diversity and dN/dS ratio estimations showed that rates of alteration varied from lesser to greater between exons and introns and between genes of primary and secondary metabolism. Even though no significant deviations from the neutral expectation were detected by either analysis, the negative values of Tajima’s D test and dN/dS ratios smaller than 1 suggested a tendency toward purifying selection with less stringency in the genes of secondary metabolism. Small sample size, low divergence among lineages and strength of positive selection affect the power of this kind of analysis. The large and negative Tajima’s D test in two genes (*HMG1* and *SGT1*) indicated an excess of rare nucleotide polymorphisms with low frequency compared with the expectation under neutral theory which could be explained by effect of genetic hitchhiking selection (Braverman *et al.* 1995).

Nucleotide diversity analyses of genes associated with traits of interest in plants have reported different evolutionary constraints related to gene function and gene segments when comparing between and within gene sequences. Giordani *et al.* (2011) analyzed eight putative drought response genes in sunflower and found greater variability in the intron regions for one gene and less variability in a pool of genes coding for regulatory proteins than in those coding enzymes involved in cell metabolism. They compared different nucleotide diversity studies of plant genes to support the theory that sequence variability increased from upstream to downstream stress response genes, *e.g.*, in Arabidopsis defense response genes involved in different signaling pathways displayed lower nonsynonymous levels of nucleotide diversity than actual R genes (Bakker *et al.* 2008). Transient balancing selection seemed to act on resistance genes to maintain high levels of protein variation in intermediate periods of time (Bakker *et al.* 2006).

Evolutionary variation of genes involved in plant metabolic pathways also has been reported. Research of synonymous and nonsynonymous genetic distances of structural genes in the anthocyanin pathway for species representing different divergent times (monocots and dicots) as well as within the genus *Ipomoea* showed that downstream genes exhibited statistically significant greater divergence rates than upstream genes (Rausher *et al.* 1999; Lu and Rausher 2003). Similar patterns of variation were found in four genes of the carotenoid biosynthetic pathway for six species (Livingstone and Anderson 2009). Significant positive rank correlation was also found between positions in the pathway and nonsynonymous substitution rates. Because upstream enzymes are intermediary of multiple end products, even slightly deleterious amino acid changes in these enzymes could have major deleterious fitness consequences. Greater constraint in upstream genes may also be explained because they are associated with pathway branches and may control pathway flux (Crabtree and Newsholme 1987). In fact, a protein interaction network analysis showed that proteins with more interactions tend to evolve more slowly, because a greater portion of the protein is directly involved in its function, and connectivity is positively associated with pleiotropic effects on cellular function (Fraser *et al.* 2002; Promislow 2004; Hahn and Kern 2005; Rausher *et al.* 2008). The nucleotide and nonsynonymous variation found in SGA biosynthetic genes could be related not only with stronger purifying selection in upstream genes (*HMG1*, *HMG2*, and *SQE*) that provide precursors for various groups of end products (triterpenes, sterols, brassinosteroids, SGAs), but also with greater protein variation of modifying enzymes in the secondary metabolism, such as SGT1 and SGT2, that direct the synthesis of unusual SGAs since the glycoside chain structure adds different toxicity properties to the final compound (Rayburn *et al.* 1994).

Regarding greater diversity found in introns of *HMG2* and *SQE* compared with their exons, contrasting effects of selection within a gene could explain differential levels of nucleotide diversity on introns. High levels of sequence conservation are expected if regulatory elements are present in the introns (Hare and Palumbi 2003). Enhancer and repressor regulatory elements have been found on introns influencing gene expression (Le Hir *et al.* 2003). In maize domestication was associated with strong selection of non-transcribed gene regions carrying a regulatory element in *teosinte branched1* gene (Wang *et al.* 1999). In *HMG2* and *SQE* the greater sequence variation in introns was caused not only by the number of SNPs but also greater frequency of indels as it was found for an apoplastic invertase inhibitor gene in potato (Datir *et al.* 2012). The lack of regulatory elements in the introns and major constrains on exon region due to pleiotropic effect of these primary metabolism genes could explain greater polymorphism on introns.

### Informative SNPs

Analysis of allelic variation of SNPs in candidate genes revealed 24 SNPs putatively associated with accumulation of SGAs. These SNPs mainly discriminated among (1) absence of SGAs in *phu* DH, (2) and the greatest accumulation of SGAs in *chc* 80-1. The whole genome analysis detected eight informative SNPs on six pseudochromosomes (1, 2, 6, 7, 9, and 11) with homozygous and heterozygous genotypes that discriminated high, intermediate, and low levels of SGA accumulation. Hierarchical cluster analysis showed that the selected informative SNPs did not follow the phylogenetic pattern found when total polymorphic SNPs were used. In general, three haplotypes could describe each accumulation level. These haplotypes cluster most of the accessions except for *cmm* 7 and *sto* 61 that did not have the allelic structure found for low level of SGAs. Sequence and analysis of polymorphisms in candidate genes is a strategy to find variation associated with phenotypes of interest. Then informative SNPs should be tested in a segregating or association mapping population to elucidate the genetic control of allelic variation in the trait of interest. Draffehn *et al.* (2010) cloned and sequenced five sugar invertase candidate genes involved in cold-induced sweetening of potato tubers from six heterozygous genotypes of potato. The variation was used to screen an association-mapping population of 219 individuals. Allelic sequences associated with chip quality and tuber starch were successfully identified. The allelic polymorphisms of our candidate genes were used to screen a segregating F_2_ population of *phu* DH × *chc* 80-1 (unpublished data). Allelic sequences of *chc* 80-1 for *HMG2* and *SGT2* were significantly associated with greater levels of SGA accumulation. In addition to the informative SNPs detected within the candidate gene *HMG2* for our germplasm panel, the SNP chip analysis detected one locus nearby this gene at 19.1 Mb on pseudochromosome 2. This chromosome has been associated with a QTL for synthesis of SGAs (Sagredo *et al.* 2006). The analysis of the segregating population also identified a locus at 61 Mb on pseudochromosome 1 that corresponded with a previously mapped QTL associated with SGA synthesis and accumulation (Sørensen *et al.* 2008). In the SNP chip cluster analysis of our germplasm panel, an informative SNP was detected at 63.6 Mb on this pseudochromosome. Together, these analyses highlight that major genes acting on synthesis and accumulation of SGA are located on pseudochromosome 1 and 2, and the potential of using this informative SNPs. Future studies will clarify the significance of the discovered informative SNP as a marker associated with SGA synthesis and accumulation.

Association of traits with allelic variation of SNPs in candidate genes could also lead to identification of sequence polymorphisms directly involved in the phenotypic variation or functional markers (Andersen and Lübberstedt 2003). The fragments analyzed in the candidate genes of this study spanned different protein domains, and the amino acid changes encoded by nonsynonymous SNPs could be directly involved in explaining phenotypes. We made a brief analysis about possible implications of amino acid changes of informative SNP in candidate genes. For *HMG2*, *SQE*, *SGT1*, and *SGT2* we identified amino acids specific to *phu* DH and *chc* 80-1. In *HMG2* the amino acid changes were near a conserved tetramerization sequence. Alignment of multiple *HMG* homologs sequences showed that there were some variable base-pairs within an otherwise highly conserved region. Further studies will determine if regulatory sequences in the intron or nonsequenced gene and its contiguous regions could influence phenotype more than the amino acid changes studied here. The SNP of *SQE* was within the squalene monooxygenase conserved domain where multiple polar amino acids were found for homologous sequences. For *SGT1*, the nine amino acid changes located within the multidomain region of the glycosyltransferase family could play a role in the catalytic activity of this protein. However, in the F_2_ population analysis there was no association of this gene and SGA synthesis or accumulation. Both *phu* DH and *chc* 80-1 had one of two sequences where amino acid changes were concentrated, so the second allelic sequences could also neutralize part of amino acid changes effects. For *SGT2* the seven amino acid changes including a deletion found within the active site of this protein could influence its activity or specificity. In fact, differences in the secondary structure of the *S. tuberosum* SGT2 aa sequence were found when the original segment was substituted by *chc* 80-1 and *phu* DH allelic fragments (Figure 2). Overall, we amplified fragments in coding regions of these candidate genes, where polymorphisms associated with SGA accumulation not only could alter the protein function but also could be linked to meaningful polymorphism in unsequenced segments. Supporting Information, File S1 and Table S1.

## Supplementary Material

Supporting Information
