# Supplementary material for: Sequence Diversity in Coding Regions of Candidate Genes in the Glycoalkaloid Biosynthetic Pathway of Wild Potato Species
Source: G3 (Bethesda). 2013 Sep 1;3(9):1467–79. doi: 10.1534/g3.113.007146 (PMC3755908; doi:10.1534/g3.113.007146)
Supplement: Supporting Information [file supp_3_9_1467__index.html]

Sequence Diversity in Coding Regions of Candidate Genes in the Glycoalkaloid Biosynthetic Pathway of Wild Potato Species — Supporting Information 

# Sequence Diversity in Coding Regions of Candidate Genes in the Glycoalkaloid Biosynthetic Pathway of Wild Potato Species

## Supporting Information for Manrique-Carpintero *et al.*, 2013

**Files in this Data Supplement:**

- Supporting Information - File S1 and Table S1 (PDF, 473 KB)
- File S1 - Allelic sequences identified in six wild and one cultivated potato species for five candidate genes within the glycoalkaloid biosynthetic pathway (PDF, 365 KB)
- Table S1 - Raw data from whole genome SNP genotyping with SolCAP 8303 Illumina Infinium potato SNP chip for 12 accessions (.xlsx, 534 KB)
