# Supplementary material for: Sequence Diversity in Coding Regions of Candidate Genes in the Glycoalkaloid Biosynthetic Pathway of Wild Potato Species
Source: G3 (Bethesda). 2013 Sep 1;3(9):1467–79. doi: 10.1534/g3.113.007146 (PMC3755908; doi:10.1534/g3.113.007146)
Supplement: Supporting Information [file supp_g3.113.007146_007146SI.pdf]

G3·Genes | Genomes | Genetics

Sequence Diversity in Coding Regions of Candidate Genes in the Glycoalkaloid Biosynthetic Pathway of Wild Potato Species

Manrique-Carpintero NC[*], Tokuhisa JG[*], Ginzberg I[§], Holliday JA[†] and Veilleux RE[*]

Author Institutional Affiliations

[*]Department of Horticulture, Virginia Polytechnic Institute and State University, Blacksburg, VA 24061, USA

[§]Department of Vegetable Research, The Volcani Center, Agricultural Research Organization, Bet Dagan, Israel

[†]Department of Forest Resources and Environmental Conservation, Virginia Polytechnic Institute and State University, Blacksburg, VA 24061, USA

DOI: 10.1534/g3.113.007146

Supporting Data 1 Allelic sequences identified in six wild and one cultivated potato species for five candidate genes within the glycoalkaloid biosynthetic pathway

1.1 3-Hydroxy-3-methylglutaryl coenzyme A reductase 1 (*HMG1*) sequences
22
HMG1_cmm7_seq1
CGACCTGTTAAGCCTCTATACACATCTAAAGATGCTTCCGCCGGCGAACCTCTGAAACAACAAGAAGTTTCTTCTCCTAAAGCATCTGATGCGC
TTCCACTCCCATTGTACCTAACCAATGGGTTGTTTTTCACCATGTTTTTCTCTGTTATGTATTTTCTTCTCGTAAGGTGGCGTGAGAAGATCCGTA
ATTCTATTCCTCTTCATGTGGTTACCCTTTCTGAATTGTTAGCTATGGTGTCATTGATTGCTTCCGTTATATATCTTTTGGGTTTCTTTGGGATTG
GGTTTGTTCAGTCGTTTGTGTCCAGGTCGAATAGTGATTCATGGGATATTGAGGATGAGAATGCTGAGCAGCTTATTATTGAGGAAGATAGC
CGCCGTGGACCATGTGCTGCTGCCACTACTCTTGGCTGCGTTGTGCCTCCACCACCTGTTCGAAAAATTGCCCCAATGGTTCCACAGCAACCTG
CTAAGGTAGCTTTGTCCCAAACGGAGAAGCCTGCGCCAATAATTATGCCAGCATTATCGGAAGATGACGAGGAGATTATACAATCTGTTGTTC
AGGGTAAAACACCATCATATTCGTTGGAATCAAAGCTTGGTGATTGTATGAGAGCTGCTTCGATTCGAAAAGAGGCGTTACAGAGGATTACA
GGGAAGTCATTAGAAGGGCTCCCATTGGAGGGATTTGACTATGAGTCTATTCTTGGACAGTGCTGTGAGATGCCTGTAGGATATGTGCAAAT
ACCGGTGGGTATTGCTGGGCCTTTGTTGCTTGATGGGAGAGAGTACTCAGTGCCAATGGCAACTACAGAAGGATGTTTAGTGGCTAGCACCA
ACAGGGGTTGCAAGGCTATCTTTGTCTCTGGTGGC
HMG1_cmm7_seq1(2)
CGACCTGTTAAGCCTCTATACACATCTAAAGATGCTTCCGCCGGCGAACCTCTGAAACAACAAGAAGTTTCTTCTCCTAAAGCATCTGATGCGC
TTCCACTCCCATTGTACCTAACCAATGGGTTGTTTTTCACCATGTTTTTCTCTGTTATGTATTTTCTTCTCGTAAGGTGGCGTGAGAAGATCCGTA
ATTCTATTCCTCTTCATGTGGTTACCCTTTCTGAATTGTTAGCTATGGTGTCATTGATTGCTTCCGTTATATATCTTTTGGGTTTCTTTGGGATTG
GGTTTGTTCAGTCGTTTGTGTCCAGGTCGAATAGTGATTCATGGGATATTGAGGATGAGAATGCTGAGCAGCTTATTATTGAGGAAGATAGC
CGCCGTGGACCATGTGCTGCTGCCACTACTCTTGGCTGCGTTGTGCCTCCACCACCTGTTCGAAAAATTGCCCCAATGGTTCCACAGCAACCTG
CTAAGGTAGCTTTGTCCCAAACGGAGAAGCCTGCGCCAATAATTATGCCAGCATTATCGGAAGATGACGAGGAGATTATACAATCTGTTGTTC
AGGGTAAAACACCATCATATTCGTTGGAATCAAAGCTTGGTGATTGTATGAGAGCTGCTTCGATTCGAAAAGAGGCGTTACAGAGGATTACA
GGGAAGTCATTAGAAGGGCTCCCATTGGAGGGATTTGACTATGAGTCTATTCTTGGACAGTGCTGTGAGATGCCTGTAGGATATGTGCAAAT
ACCGGTGGGTATTGCTGGGCCTTTGTTGCTTGATGGGAGAGAGTACTCAGTGCCAATGGCAACTACAGAAGGATGTTTAGTGGCTAGCACCA
ACAGGGGTTGCAAGGCTATCTTTGTCTCTGGTGGC
HMG1_cmm26_seq2
CGACCTGTTAAGCCTCTATACACATCTAAAGATGCTTCCGCCGGCGAACCTCTGAAACAACAAGAAGTTTCTTCTCCTAAAGCATCTGATGCGC
TTCCACTCCCATTGTACCTAACCAATGGGTTGTTTTTCACCATGTTTTTCTCTGTTATGTATTTTCTTCTCGTAAGGTGGCGTGAGAAGATCCGTA
ATTCTATTCCTCTTCATGTGGTTACCCTTTCTGAATTGTTAGCTATGGTGTCATTGATTGCTTCCGTTATATATCTTTTGGGTTTCTTTGGGATTG
GGTTTGTTCAGTCGTTTGTGTCCAGGTCGAATAGTGATTCATGGGATATTGAGGATGAGAATGCTGAGCAGCTTATTATTGAGGAAGATAGC
CGCCGTGGACCATGTGCTGCTGCCACTACTCTTGGCTGCGTTGTGCCTCCACCACCTGTTCGAAAAATTGCCCCAATGGTTCCACAGCAACCTG
CTAAGGTAGCTTTGTCCCAAACGGAGAAGCCTGCGCCAATAATTATGCCAGCATTATCGGAAGATGACGAGGAGATTATACAATCTGTTGTTC
AGGGTAAAACACCATCATATTCGTTGGAATCAAAGCTTGGTGATTGTATGAGAGCTGCTTCGATTCGAAAAGAGGCGTTACAGAGGATTACA
GGGAAGTCATTGGAAGGGCTCCCATTGGAGGGATTTGACTATGAGTCTATTCTTGGACAGTGCTGTGAGATGCCTGTAGGATATGTGCAAAT
ACCGGTGGGTATTGCTGGGCCTTTGCTGCTTGATGGGAGAGAGTACTCAGTGCCAATGGCAACTACAGAAGGATGTTTAGTGGCTAGCACCA
ACAGGGGTTGCAAGGCTATCTTTGTCTCTGGTGGC
HMG1_cmm26_seq2(2)
CGACCTGTTAAGCCTCTATACACATCTAAAGATGCTTCCGCCGGCGAACCTCTGAAACAACAAGAAGTTTCTTCTCCTAAAGCATCTGATGCGC
TTCCACTCCCATTGTACCTAACCAATGGGTTGTTTTTCACCATGTTTTTCTCTGTTATGTATTTTCTTCTCGTAAGGTGGCGTGAGAAGATCCGTA
ATTCTATTCCTCTTCATGTGGTTACCCTTTCTGAATTGTTAGCTATGGTGTCATTGATTGCTTCCGTTATATATCTTTTGGGTTTCTTTGGGATTG
GGTTTGTTCAGTCGTTTGTGTCCAGGTCGAATAGTGATTCATGGGATATTGAGGATGAGAATGCTGAGCAGCTTATTATTGAGGAAGATAGC
CGCCGTGGACCATGTGCTGCTGCCACTACTCTTGGCTGCGTTGTGCCTCCACCACCTGTTCGAAAAATTGCCCCAATGGTTCCACAGCAACCTG
CTAAGGTAGCTTTGTCCCAAACGGAGAAGCCTGCGCCAATAATTATGCCAGCATTATCGGAAGATGACGAGGAGATTATACAATCTGTTGTTC
AGGGTAAAACACCATCATATTCGTTGGAATCAAAGCTTGGTGATTGTATGAGAGCTGCTTCGATTCGAAAAGAGGCGTTACAGAGGATTACA
GGGAAGTCATTGGAAGGGCTCCCATTGGAGGGATTTGACTATGAGTCTATTCTTGGACAGTGCTGTGAGATGCCTGTAGGATATGTGCAAAT
ACCGGTGGGTATTGCTGGGCCTTTGCTGCTTGATGGGAGAGAGTACTCAGTGCCAATGGCAACTACAGAAGGATGTTTAGTGGCTAGCACCA
ACAGGGGTTGCAAGGCTATCTTTGTCTCTGGTGGC
HMG1_dms54_seq1
CGACCTGTTAAGCCTCTATACACATCTAAAGATGCTTCCGCCGGCGAACCTCTGAAGCAACAAGAAGTTTCTTCTCCTAAAGCATCTGATGCGC
TTCCACTCCCATTGTACCTAACCAATGGGTTGTTTTTCACCATGTTTTTCTCTGTTATGTATTTTCTTCTCGTAAGGTGGCGTGAGAAGATCCGTA
ATTCTATTCCTCTTCATGTGGTTACCCTTTCTGAATTGTTAGCTATGGTGTCATTGATTGCTTCCGTTATATATCTTTTGGGTTTCTTTGGGATTG
GGTTTGTTCAGTCGTTTGTGTCCAGGTCGAATAGTGATTCATGGGATATTGAGGATGAGAATGCTGAGCAGCTTATTATTGAGGAAGATAGC
CGCCGTGGACCATGTGCTGCTGCCACTACTCTTGGCTGCGTTGTGCCTCCACCACCTGTTCGAAAAATTGCCCCAATGGTTCCACAGCAACCTG
CTAAGGTAGCTTTGTCCCAAACGGAGAAGCCTGCGCCAATAATTATGCCAGCATTATCGGAAGATGACGAGGAGATTATACAATCTGTTGTTC
AGGGTAAAACACCATCATATTCGTTGGAATCAAAGCTTGGTGATTGTATGAGAGCTGCTTCGATTCGAAAAGAGGCGTTACAGAGGATTACA
GGGAAGTCATTGGAAGGGCTCCCATTGGAGGGATTTGACTATGAGTCTATTCTTGGACAGTGCTGTGAGATGCCTGTAGGATATGTGCAAAT

ACCGGTGGGTATTGCTGGGCCTTTGTTGCTTGATGGGAGAGAGTACTCAGTGCCAATGGCAACTACAGAAGGATGTTTAGTGGCTAGCACCA
ACAGGGGTTGCAAGGCTATCTTTGTCTCTGGTGGC
HMG1_dms54_seq2
CGACCTGTTAAGCCTCTATACACATCTAAAGATGCTTCCGCCGGCGAACCTCTGAAACAACAAGAAGTTTCTTCTCCTAAAGCATCTGATGCGC
TTCCACTCCCATTGTACCTAACCAATGGGCTGTTTTTCACCATGTTTTTCTCTGTTATGTATTTTCTTCTCGTAAGGTGGCGTGAGAAGATCCGT
AATTCTATTCCTCTTCATGTGGTTACCCTTTCTGAATTGTTAGCTATGGTGTCATTGATTGCTTCCGTTATATATCTTTTGGGTTTCTTTGGGATT
GGGTTTGTTCAGTCGTTTGTGTCCAGGTCGAATAGTGATTCATGGGATATTGAGGATGAGAATGCTGAGCAGCTTATTATTGAGGAAGATAG
CCGCCGTGGACCATGTGCTGCTGCCACTACTCTTGGCTGCGTTGTGCCTCCACCACCTGTTCGAAAAATTGCCCCAATGGTTCCACTGCAACCT
GCTAAGGTAGCTTTGTCCCAAACGGAGAAGCCTGCGCCAATAATTATGCCAGCATTATCGGAAGATGACGAGGAGATTATACAATCTGTTGT
TCAGGGTAAAACACCATCATATTCGTTGGAATCAAAGCTTGGTGATTGTATGAGAGCTGCTTCGATTCGAAAAGAGGCGTTACAGAGGATTA
CAGGGAAGTCATTGGAAGGGCTCCCATTGGAGGGATTTGACTATGAGTCTATTCTTGGACAGTGCTGTGAGATGCCTGTAGGATATGTGCAA
ATACCGGTGGGTATTGCTGGGCCTTTGTTGCTTGATGGGAGAGAGTACTCAGTGCCAATGGCAACTACAGAAGGATGTTTAGTGGCTAGCAC
CAACAGGGGTTGCAAGGCTATCTTTGTCTCTGGTGGC
HMG1_dms78_seq1
CGACCTGTTAAGCCTCTATACACATCTAAAGATGCTTCCGCCGGCGAACCTCTGAAGCAACAAGAAGTTTCTTCTCCTAAAGCATCTGATGCGC
TTCCACTCCCATTGTACCTAACCAATGGGTTGTTTTTCACCATGTTTTTCTCTGTTATGTATTTTCTTCTCGTAAGGTGGCGTGAGAAGATCCGTA
ATTCTATTCCTCTTCATGTGGTTACCCTTTCTGAATTGTTAGCTATGGTGTCATTGATTGCTTCCGTTATATATCTTTTGGGTTTCTTTGGGATTG
GGTTTGTTCAGTCGTTTGTGTCCAGGTCGAATAGTGATTCATGGGATATTGAGGATGAGAATGCTGAGCAGCTTATTATTGAGGAAGATAGC
CGCCGTGGACCATGTGCTGCTGCCACTACTCTTGGCTGCGTTGTGCCTCCACCACCTGTTCGAAAAATTGCCCCAATGGTTCCACAGCAACCTG
CTAAGGTAGCTTTGTCCCAAACGGAGAAGCCTGCGCCAATAATTATGCCAGCATTATCGGAAGATGACGAGGAGATTATACAATCTGTTGTTC
AGGGTAAAACACCATCATATTCGTTGGAATCAAAGCTTGGTGATTGTATGAGAGCTGCTTCGATTCGAAAAGAGGCGTTACAGAGGATTACA
GGGAAGTCATTGGAAGGGCTCCCATTGGAGGGATTTGACTATGAGTCTATTCTTGGACAGTGCTGTGAGATGCCTGTAGGATATGTGCAAAT
ACCGGTGGGTATTGCTGGGCCTTTGTTGCTTGATGGGAGAGAGTACTCAGTGCCAATGGCAACTACAGAAGGATGTTTAGTGGCTAGCACCA
ACAGGGGTTGCAAGGCTATCTTTGTCTCTGGTGGC
HMG1_dms78_seq2
CGACCTGTTAAGCCTCTATACACATCTAAAGATGCTTCCGCCGGCGAACCTCTGAAACAACAAGAAGTTTCTTCTCCTAAAGCATCTGATGCGC
TTCCACTCCCATTGTACCTAACCAATGGGCTGTTTTTCACCATGTTTTTCTCTGTTATGTATTTTCTTCTCGTAAGGTGGCGTGAGAAGATCCGT
AATTCTATTCCTCTTCATGTGGTTACCCTTTCTGAATTGTTAGCTATGGTGTCATTGATTGCTTCCGTTATATATCTTTTGGGTTTCTTTGGGATT
GGGTTTGTTCAGTCGTTTGTGTCCAGGTCGAATAGTGATTCATGGGATATTGAGGATGAGAATGCTGAGCAGCTTATTATTGAGGAAGATAG
CCGCCGTGGACCATGTGCTGCTGCCACTACTCTTGGCTGCGTTGTGCCTCCACCACCTGTTCGAAAAATTGCCCCAATGGTTCCACTGCAACCT
GCTAAGGTAGCTTTGTCCCAAACGGAGAAGCCTGCGCCAATAATTATGCCAGCATTATCGGAAGATGACGAGGAGATTATACAATCTGTTGT
TCAGGGTAAAACACCATCATATTCGTTGGAATCAAAGCTTGGTGATTGTATGAGAGCTGCTTCGATTCGAAAAGAGGCGTTACAGAGGATTA
CAGGGAAGTCATTGGAAGGGCTCCCATTGGAGGGATTTGACTATGAGTCTATTCTTGGACAGTGCTGTGAGATGCCTGTAGGATATGTGCAA
ATACCGGTGGGTATTGCTGGGCCTTTGTTGCTTGATGGGAGAGAGTACTCAGTGCCAATGGCAACTACAGAAGGATGTTTAGTGGCTAGCAC
CAACAGGGGTTGCAAGGCTATCTTTGTCTCTGGTGGC
HMG1_spg55_seq1
CGACCTGTTAAGCCTCTATACACATCTAAAGATGCTTCCGCCGGCGAACCTCTGAAACAACAAGAAGTTTCTTCTCCTAAAGCATCTGATGCGC
TTCCACTCCCATTGTACCTAACCAATGGGTTGTTTTTCACCATGTTTTTCTCTGTTATGTATTTTCTTCTCGTAAGGTGGCGTGAGAAGATCCGTA
ATTCTATTCCTCTTCATGTGGTTACCCTTTCTGAATTGTTAGCTATGGTGTCATTGATTGCTTCCGTTATATATCTTTTGGGTTTCTTTGGGATTG
GGTTTGTTCAGTCGTTTGTGTCCAGGTCGAATAGTGATTCATGGGATATTGAGGATGAGAATGCTGAGCAGCTTATTATTGAGGAAGATAGC
CGCCGTGGACCATGTGCTGCTGCCACTACTCTTGGCTGCGTTGTGCCTCCACCACCTGTTCGAAAAATTGCCCCAATGGTTCCACAGCAACCTG
CTAAGGTAGCTTTGTCCCAAACGGAGAAGCCTGCGCCAATAATTATGCCAGCATTATCGGAAGATGACGAGGAGATTATACAATCTGTTGTTC
AGGGTAAAACACCATCATATTCGTTGGAATCAAAGCTTGGTGATTGTATGAGAGCTGCTTCGATTCGAAAAGAGGCGTTACAGAGGATTACA
GGGAAGTCATTGGAAGGGCTCCCATTGGAGGGATTTGACTATGAGTCTATTCTTGGACAGTGCTGTGAGATGCCTGTAGGATATGTGCAAAT
ACCGGTGGGTATTGCTGGGCCTTTGTTGCTTGATGGGAGAGAGTACTCAGTGCCAATGGCAACTACAGAAGGATGTTTAGTGGCTAGCACCA
ACAGGGGTTGCAAGGCTATCTTTGTCTCTGGTGGC
HMG1_spg55_seq2
CGACCTGTTAAGCCTCTATACACATCTAAAGATGCTTTCGCCGGCGAACCTCTGAAACAAGAAGTTTTTTCTCCTAAAGCATCTGATGCGCTTC
CACTCCCATTGTACCTAACCACTGGGTTGTTTTTCACCATGTTTTTCTCTGTTATGTATTTTCTTCTCGTAAGGTGGCGTGAGAAGATCCGTAATT
CTATTCCTCTTCATGTGGTTACCCTTTCTGAATTGTTAGCTATGGTGTCATTGATTGCTTCCGTTATATATCTTTTGGGTTTCTTTGGGATTGGGT
TTGTTCAGTCATTTGTGTCCAGGTCGAATAGTGATTCATGGGATATTGAGGATGAGAATGCTGAGCAGCTTATTATTGAGGAAGATAGCCGTC
GCGGACCATGTGCTGCTGCCACTACTCTTGGCTGCGTTGTGCCTCCACCACCTGTTCGAAAAATTGCCCCAATGGTTCCACAGCAACCTGCTAA
GGTAGCTTTGTCCCAAATGGAGAAGCCTGCGCCAATAATTATGCCCGCATTATCGGAAGATGACGAGGAGATTATACAATCTGTTGTTCAGG
GTAAAACACCATCATATTCGTTGGAATCAAAGCTTGGTGATTGTATGAGAGCTGCTTCGATTCAAAAAGAGGCGTTACAGAGGATTACAGGG
AAGTCATTGGAAGGGCTCCCATTGGAGGGATTTGACTATGAGTCTATTCTTGGACAGTGCTGTGAGATGCCTGTAGGATATGTGCAAATACC
GGTGGGTATTGTTGGGCCTTTGTTGCTTGATGGGAGAGAGTACTCAGTGCCAATGGCAACTACAGAAGGATGCTTAGTGGCTAGCACCAACA
GGGGTTGCAAGGCTATCTTTGTCTCTGGTGGC
HMG1_spg74_seq1

CGACCTGTTAAGCCTCTATACACATCTAAAGATGCTTCCGCCGGCGAACCTCTGAAACAACAAGAAGTTTCTTCTCCTAAAGCATCTGATGCGC
TTCCACTCCCATTGTACCTAACCAATGGGTTGTTTTTCACCATGTTTTTCTCTGTTATGTATTTTCTTCTCGTAAGGTGGCGTGAGAAGATCCGTA
ATTCTATTCCTCTTCATGTGGTTACCCTTTCTGAATTGTTAGCTATGGTGTCATTGATTGCTTCCGTTATATATCTTTTGGGTTTCTTTGGGATTG
GGTTTGTTCAGTCGTTTGTGTCCAGGTCGAATAGTGATTCATGGGATATTGAGGATGAGAATGCTGAGCAGCTTATTATTGAGGAAGATAGC
CGCCGTGGACCATGTGCTGCTGCCACTACTCTTGGCTGCGTTGTGCCTCCACCACCTGTTCGAAAAATTGCCCCAATGGTTCCACAGCAACCTG
CTAAGGTAGCTTTGTCCCAAACGGAGAAGCCTGCGCCAATAATTATGCCAGCATTATCGGAAGATGACGAGGAGATTATACAATCTGTTGTTC
AGGGTAAAACACCATCATATTCGTTGGAATCAAAGCTTGGTGATTGTATGAGAGCTGCTTCGATTCGAAAAGAGGCGTTACAGAGGATTACA
GGGAAGTCATTGGAAGGGCTCCCATTGGAGGGATTTGACTATGAGTCTATTCTTGGACAGTGCTGTGAGATGCCTGTAGGATATGTGCAAAT
ACCGGTGGGTATTGCTGGGCCTTTGTTGCTTGATGGGAGAGAGTACTCAGTGCCAATGGCAACTACAGAAGGATGTTTAGTGGCTAGCACCA
ACAGGGGTTGCAAGGCTATCTTTGTCTCTGGTGGC
HMG1_spg74_seq2
CGACCTGTTAAGCCTCTATACACATCTAAAGATGCTTTCGCCGGCGAACCTCTGAAACAAGAAGTTTTTCTCCTAAAGCATCTGATGCGCTTC
CACTCCCATTGTACCTAACCACTGGGTTGTTTTTCACCATGTTTTTCTCTGTTATGTATTTTCTTCTCGTAAGGTGGCGTGAGAAGATCCGTAATT
CTATTCCTCTTCATGTGGTTACCCTTTCTGAATTGTTAGCTATGGTGTCATTGATTGCTTCCGTTATATATCTTTTGGGTTTCTTTGGGATTGGGT
TTGTTCAGTCATTTGTGTCCAGGTCGAATAGTGATTCATGGGATATTGAGGATGAGAATGCTGAGCAGCTTATTATTGAGGAAGATAGCCGTC
GCGGACCATGTGCTGCTGCCACTACTCTTGGCTGCGTTGTGCCTCCACCACCTGTTCGAAAAATTGCCCCAATGGTTCCACAGCAACCTGCTAA
GGTAGCTTTGTCCCAAATGGAGAAGCCTGCGCCAATAATTATGCCCGCATTATCGGAAGATGACGAGGAGATTATACAATCTGTTGTTCAGG
GTAAAACACCATCATATTCGTTGGAATCAAAGCTTGGTGATTGTATGAGAGCTGCTTCGATTCAAAAAGAGGCGTTACAGAGGATTACAGGG
AAGTCATTGGAAGGGCTCCCATTGGAGGGATTTGACTATGAGTCTATTCTTGGACAGTGCTGTGAGATGCCTGTAGGATATGTGCAAATACC
GGTGGGTATTGTTGGGCCTTTGTTGCTTGATGGGAGAGAGTACTCAGTGCCAATGGCAACTACAGAAGGATGCTTAGTGGCTAGCACCAACA
GGGGTTGCAAGGCTATCTTTGTCTCTGGTGGC
HMG1_spl16_seq2
CGACCTGTTAAGCCTCTATACACATCTAAAGATGCTTCCGCCGGCGAACCTCTGAAACAACAAGAAGTTTCTTCTCCTAAAGCATCTGATGCGC
TTCCACTCCCATTGTACCTAACCAATGGGTTGTTTTTCACCATGTTTTTCTCTGTTATGTATTTTCTTCTCGTAAGGTGGCGTGAGAAGATCCGTA
ATTCTATTCCTCTTCATGTGGTTACCCTTTCTGAATTGTTAGCTATGGTGTCATTGATTGCTTCCGTTATATATCTTTTGGGTTTCTTTGGGATTG
GGTTTGTTCAGTCGTTTGTGTCCAGGTCGAATAGTGATTCATGGGATATTGAGGATGAGAATGCTGAGCAGCTTATTATTGAGGAAGATAGC
CGCCGTGGACCATGTGCTGCTGCCACTACTCTTGGCTGCGTTGTGCCTCCACCACCTGTTCGAAAAATTGCCCCAATGGTTCCACAGCAACCTG
CTAAGGTAGCTTTGTCCCAAACGGAGAAGCCTGCGCCAATAATTATGCCAGCATTATCGGAAGATGACGAGGAGATTATACAATCTGTTGTTC
AGGGTAAAACACCATCATATTCGTTGGAATCAAAGCTTGGTGATTGTATGAGAGCTGCTTCGATTCGAAAAGAAGCGTTACAGAGGATTACA
GGGAAGTCATTGGAAGGGCTCCCATTGGAGGGATTTGACTATGAGTCTATTCTTGGACAGTGCTGTGAGATGCCTGTAGGATATGTGCAAAT
ACCGGTGGGTATTGCTGGGCCTTTGTTGCTTGATGGGAGAGAGTACTCAGTGCCAATGGCAACTACAGAAGGATGTTTAGTGGCTAGCACCA
ACAGGGGTTGCAAGGCTATCTTTGTCTCTGGTGGC
HMG1_spl81_seq3
CGACCTGTTAAGCCTCTATACACATCTAAAGATGCTTCCGCCGGCGAACCTCTGAAACAACAAGAAGTTTCTTCTCCTAAAGCATCTGATGCGC
TTCCACTCCCATTGTACCTAACCAATGGGCTGTTTTTCACCATGTTTTTCTCTGTTATGTATTTTCTTCTCGTAAGGTGGCGTGAGAAGATCCGT
AATTCTATTCCTCTTCATGTGGTTACCCTTTCTGAATTGTTAGCTATGGTGTCATTGATTGCTTCCGTTATATATCTTTTGGGTTTCTTTGGGATT
GGGTTTGTTCAGTCGTTTGTGTCCAGGTCGAATAGTGATTCATGGGATATTGAGGATGAGAATGCTGAGCAGCTTATTATTGAGGAAGATAG
CCGCCGTGGACCATGTGCTGCTGCCACTACTCTTGGCTGCGTTGTGCCTCCACCACCTGTTCGAAAAATTGCCCCAATGGTTCCACAGCAACCT
GCTAAGGTAGCTTTGTCCCAAACGGAGAAGCCTGCGCCAATAATTATGCCAGCATTATCGGAAGATGACGAGGAGATTATACAATCTGTTGT
TCAGGGTAAAACACCATCATATTCGTTGGAATCAAAGCTTGGTGATTGTATGAGAGCTGCTTCGATTCGAAAAGAGGCGTTACAGAGGATTA
CAGGGAAGTCATTGGAAGGGCTCCCATTGGAGGGATTTGACTATGAGTCTATTCTTGGACAGTGCTGTGAGATGCCTGTAGGATATGTGCAA
ATACCGGTGGGTATTGCTGGGCCTTTGTTGCTTGATGGGAGAGAGTACTCAGTGCCAATGGCAACTACAGAAGGATGTTTAGTGGCTAGCAC
CAACAGGGGTTGCAAGGCTATCTTTGTCTCTGGTGGC
HMG1_sto40_seq1
CGACCTGTTAAGCCTCTATACACATCTAAAGATGCTTCCGCCGGCGAACCTCTGAAACAACAAGAAGTTTCTTCTCCTAAAGCATCTGATGCGC
TTCCACTCCCATTGTACCTAACCAATGGGTTGTTTTTCACCATGTTTTTCTCTGTTATGTATTTTCTTCTCGTAAGGTGGCGTGAGAAGATCCGTA
ATTCTATTCCTCTTCATGTGGTTACCCTTTCTGAATTGTTAGCTATGGTGTCATTGATTGCTTCCGTTATATATCTTTTGGGTTTCTTTGGGATTG
GGTTTGTTCAGTCGTTTGTGTCCAGGTCGAATAGTGATTCATGGGATATTGAGGATGAGAATGCTGAGCAGCTTATTATTGAGGAAGATAGC
CGTCGTGGACCATGTGCTGCTGCCACTACTCTTGGCTGCGTTGTGCCTCCACCACCTGTTCGAAAAATTGCCCCAATGGTTCCACAGCAACCTG
CTAAGGTAGCTTTGTCCCAAACGGAGAAACCTGCGCCAATAATTATGCCAGCATTATCGGAAGATGACGAGGAGATTATACAATCTGTTGTTC
AGGGTAAAACACCATCATATTCGTTGGAATCAAAGCTTGGTGATTGTATGAGAGCTGCTTCGATTCGAAAAGAGGCGTTACAGAGGATTACA
GGGAAGTCATTGGAAGGGCTCCCATTGGAGGGATTTGACTATGAGTCTATTCTTGGACAGTGTTGTGAGATGCCTGTAGGATATGTGCAAAT
ACCGGTGGGTATTGCTGGGCCTTTGTTGCTTGATGGGAGAGAGTACTCAGTGCCAATGGCAACTACAGAAGGATGTTTAGTGGCTAGCACCA
ACAGGGGTTGCAAGGCTATCTTTGTCTCTGGTGGC
HMG1_sto40_seq2
CGACCTGTTAAGCCTCTATACACATCTAAAGATGCTTCCGCCGGCGAACCTCTGAAACAACAAGAAGTTTCTTCTCCTAAAGCATCTGATGCGC
TTCCACTCCCATTGTACCTAACCAATGGGTTGTTTTTCACCATGTTTTTCTCTGTTATGTATTTTCTTCTCGTAAGGTGGCGTGAGAAGATCCGTA
ATTCTATTCCTCTTCATGTGGTTACCCTTTCTGAATTGTTAGCTATGGTGTCATTGATTGCTTCCGTTATATATCTTTTGGGTTTCTTTGGGATTG

GGTTTGTTCAGTCGTTTGTGTCCAGGTCGAATAGTGATTCATGGGATATTGAGGATGAGAATGCTGAGCAGCTTATTATTGAGGAAGATAGC
CGCCGTGGACCATGTGCTGCTGCCACTACTCTTGGCTGCGTTGTGCCTCCACCACCTGTTCGAAAAATTGCCCCAATGGTTCCACAGCAACCTG
CTAAGGTAGCTTTGTCCCAAACGGAGAAGCCTGCGCCAATAATTATGCCAGCATTATCGGAAGATGACGAGGAGATTATACAATCTGTTGTTC
AGGGTAAAACACCATCATATTCGTTGGAATCAAAGCTTGGTGATTGTATGAGAGCTGCTTCGATTCGAAAAGAAGCGTTACAGAGGATTACA
GGGAAGTCATTGGAAGGGCTCCCATTGGAGGGATTTGACTATGAGTCTATTCTTGGACAGTGCTGTGAGATGCCTGTAGGATATGTGCAAAT
ACCGGTGGGTATTGCTGGGCCTTTGTTGCTTGATGGGAGAGAGTACTCAGTGCCAATGGCAACTACAGAAGGATGTTTAGTGGCTAGCACCA
ACAGGGGTTGCAAGGCTATCTTTGTCTCTGGTGGC

HMG1_sto61_seq1

CGACCTGTTAAGCCTCTATACACATCTAAAGATGCTTCCGCCGGCGAACCTCTGAAACAACAAGAAGTTTCTTCTCCTAAAGCATCTGATGCGC
TTCCACTCCCATTGTACCTAACCAATGGGTTGTTTTTCACCATGTTTTTCTCTGTTATGTATTTTCTTCTCGTAAGGTGGCGTGAGAAGATCCGTA
ATTCTATTCCTCTTCATGTGGTTACCCTTTCTGAATTGTTAGCTATGGTGTCATTGATTGCTTCCGTTATATATCTTTTGGGTTTCTTTGGGATTG
GGTTTGTTCAGTCGTTTGTGTCCAGGTCGAATAGTGATTCATGGGATATTGAGGATGAGAATGCTGAGCAGCTTATTATTGAGGAAGATAGC
CGTCGTGGACCATGTGCTGCTGCCACTACTCTTGGCTGCGTTGTGCCTCCACCACCTGTTCGAAAAATTGCCCCAATGGTTCCACAGCAACCTG
CTAAGGTAGCTTTGTCCCAAACGGAGAAACCTGCGCCAATAATTATGCCAGCATTATCGGAAGATGACGAGGAGATTATACAATCTGTTGTTC
AGGGTAAAACACCATCATATTCGTTGGAATCAAAGCTTGGTGATTGTATGAGAGCTGCTTCGATTCGAAAAGAGGCGTTACAGAGGATTACA
GGGAAGTCATTGGAAGGGCTCCCATTGGAGGGATTTGACTATGAGTCTATTCTTGGACAGTGTTGTGAGATGCCTGTAGGATATGTGCAAAT
ACCGGTGGGTATTGCTGGGCCTTTGTTGCTTGATGGGAGAGAGTACTCAGTGCCAATGGCAACTACAGAAGGATGTTTAGTGGCTAGCACCA
ACAGGGGTTGCAAGGCTATCTTTGTCTCTGGTGGC

HMG1_sto61_seq2

CGACCTGTTAAGCCTCTATACACATCTAAAGATGCTTCCGCCGGCGAACCTCTGAAACAACAAGAAGTTTCTTCTCCTAAAGCATCTGATGCGC
TTCCACTCCCATTGTACCTAACCAATGGGTTGTTTTTCACCATGTTTTTCTCTGTTATGTATTTTCTTCTCGTAAGGTGGCGTGAGAAGATCCGTA
ATTCTATTCCTCTTCATGTGGTTACCCTTTCTGAATTGTTAGCTATGGTGTCATTGATTGCTTCCGTTATATATCTTTTGGGTTTCTTTGGGATTG
GGTTTGTTCAGTCGTTTGTGTCCAGGTCGAATAGTGATTCATGGGATATTGAGGATGAGAATGCTGAGCAGCTTATTATTGAGGAAGATAGC
CGCCGTGGACCATGTGCTGCTGCCACTACTCTTGGCTGCGTTGTGCCTCCACCACCTGTTCGAAAAATTGCCCCAATGGTTCCACAGCAACCTG
CTAAGGTAGCTTTGTCCCAAACGGAGAAGCCTGCGCCAATAATTATGCCAGCATTATCGGAAGATGACGAGGAGATTATACAATCTGTTGTTC
AGGGTAAAACACCATCATATTCGTTGGAATCAAAGCTTGGTGATTGTATGAGAGCTGCTTCGATTCGAAAAGAAGCGTTACAGAGGATTACA
GGGAAGTCATTGGAAGGGCTCCCATTGGAGGGATTTGACTATGAGTCTATTCTTGGACAGTGCTGTGAGATGCCTGTAGGATATGTGCAAAT
ACCGGTGGGTATTGCTGGGCCTTTGTTGCTTGATGGGAGAGAGTACTCAGTGCCAATGGCAACTACAGAAGGATGTTTAGTGGCTAGCACCA
ACAGGGGTTGCAAGGCTATCTTTGTCTCTGGTGGC

HMG1_tbr_seq1

CGACCTGTTAAGCCTCTATACACATCTAAAGATGCTTCCGCCGGCGAACCTCTGAAACAACAAGAAGTTTCTTCTCCTAAAGCATCTGATGCGC
TTCCACTCCCATTGTACCTAACCAATGGGTTGTTTTTCACCATGTTTTTCTCTGTTATGTATTTTCTTCTCGTAAGGTGGCGTGAGAAGATCCGTA
ATTCTATTCCTCTTCATGTGGTTACCCTTTCTGAATTGTTAGCTATGGTGTCATTGATTGCTTCCGTTATATATCTTTTGGGTTTCTTTGGGATTG
GGTTTGTTCAGTCGTTTGTGTCCAGGTCGAATAGTGATTCATGGGATATTGAGGATGAGAATGCTGAGCAGCTTATTATTGAGGAAGATAGC
CGCCGTGGACCATGTGCTGCTGCCACTACTCTTGGCTGCGTTGTGCCTCCACCACCTGTTCGAAAAATTGCCCCAATGGTTCCACAGCAACCTG
CTAAGGTAGCTTTGTCCCAAACGGAGAAGCCTTCGCCAATAATTATGCCAGCATTATCGGAAGATGACGAGGAGATTATACAATCTGTTGTTC
AGGGTAAAACACCATCATATTCGTTGGAATCAAAGCTTGGTGATTGTATGAGAGCTGCTTCGATTCGAAAAGAGGCGTTACAGAGGATTACA
GGGAAGTCATTGGAAGGGCTCCCATTGGAGGGATTTGACTATTCGTCTATTCTTGGACAGTGCTGTGAGATGCCTGTAGGATATGTGCAAAT
ACCGGTGGGTATTGCTGGGCCTTTGTTGCTTGATGGGAGAGAGTACTCAGTGCCAATGGCAACTACAGAAGGATGTTTAGTGGCTAGCACCA
ACAGGGGTTGCAAGGCTATCTTTGTCTCTGGTGGC

HMG1_tbr_seq1(2)

CGACCTGTTAAGCCTCTATACACATCTAAAGATGCTTCCGCCGGCGAACCTCTGAAACAACAAGAAGTTTCTTCTCCTAAAGCATCTGATGCGC
TTCCACTCCCATTGTACCTAACCAATGGGTTGTTTTTCACCATGTTTTTCTCTGTTATGTATTTTCTTCTCGTAAGGTGGCGTGAGAAGATCCGTA
ATTCTATTCCTCTTCATGTGGTTACCCTTTCTGAATTGTTAGCTATGGTGTCATTGATTGCTTCCGTTATATATCTTTTGGGTTTCTTTGGGATTG
GGTTTGTTCAGTCGTTTGTGTCCAGGTCGAATAGTGATTCATGGGATATTGAGGATGAGAATGCTGAGCAGCTTATTATTGAGGAAGATAGC
CGCCGTGGACCATGTGCTGCTGCCACTACTCTTGGCTGCGTTGTGCCTCCACCACCTGTTCGAAAAATTGCCCCAATGGTTCCACAGCAACCTG
CTAAGGTAGCTTTGTCCCAAACGGAGAAGCCTTCGCCAATAATTATGCCAGCATTATCGGAAGATGACGAGGAGATTATACAATCTGTTGTTC
AGGGTAAAACACCATCATATTCGTTGGAATCAAAGCTTGGTGATTGTATGAGAGCTGCTTCGATTCGAAAAGAGGCGTTACAGAGGATTACA
GGGAAGTCATTGGAAGGGCTCCCATTGGAGGGATTTGACTATTCGTCTATTCTTGGACAGTGCTGTGAGATGCCTGTAGGATATGTGCAAAT
ACCGGTGGGTATTGCTGGGCCTTTGTTGCTTGATGGGAGAGAGTACTCAGTGCCAATGGCAACTACAGAAGGATGTTTAGTGGCTAGCACCA
ACAGGGGTTGCAAGGCTATCTTTGTCTCTGGTGGC

HMG1_DM_seq3

CGACCTGTTAAGCCTCTATACACATCTAAAGATGCTTCCGCCGGCGAACCTCTGAAACAAGAAGTTTCTTCTCCTAAAGCATCTGATGCGCTTC
CACTCCCATTGTACCTAACCACTGGGTTGTTTTTCACCATGTTTTTCTCTGTTATGTATTTTCTTCTCGTAAGGTGGCGTGAGAAGATCCGTAATT
CTATTCCTCTTCATGTGGTTACCCTTTCTGAATTGTTAGCTATGGTGTCATTGATTGCTTCCGTTATATATCTTTTGGGTTTCTTTGGGATTGGGT
TTGTTCAGTCGTTTGTGTCCAGGTCGAATAGTGATTCATGGGATATTGAGGATGAGAATGCTGAGCAGCTTATTATTGAGGAAGATAGCCGC
CGTGGACCATGTGCTGCTGCCACTACTCTTGGCTGCGTTGTGCCTCCACCACCTGTTCGAAAAATTGCCCCAATGGTTCCACAGCAACCTGCTA
AGGTAGCTTTGTCCCAAACGGAGAAGCCTTCGCCAATAATTATGCCAGCATTATCGGAAGATGACGAGGAGATTATACAATCTGTTGTTCAG

GGTAAAACACCATCATATTCGTTGGAATCAAAGCTTGGTGATTGTATGAGAGCTGCTTCGATTCGAAAAGAGGCGTTACAGAGGATTACAGG
GAAGTCATTGGAAGGGCTCCCATTGGAGGGATTTGACTATGAGTCTATTCTTGGACAGTGCTGTGAGATGCCTGTAGGATATGTGCAAATAC
CGGTGGGTATTGCTGGGCCTTTGTTGCTTGATGGGAGAGAGTACTCAGTGCCAATGGCAACTACAGAAGGATGTTTAGTGGCTAGCACCAAC
AGGGGTTGCAAGGCTATCTTTGTCTCTGGTGGC

HMG1_DM_seq3(2)

CGACCTGTTAAGCCTCTATACACATCTAAAGATGCTTCCGCCGGCGAACCTCTGAAACAAGAAGTTTCTTCTCCTAAAGCATCTGATGCGCTTC
CACTCCCATTGTACCTAACCACTGGGTTGTTTTTCACCATGTTTTTCTCTGTTATGTATTTTCTTCTCGTAAGGTGGCGTGAGAAGATCCGTAATT
CTATTCCTCTTCATGTGGTTACCCTTTCTGAATTGTTAGCTATGGTGTCATTGATTGCTTCCGTTATATATCTTTTGGGTTTCTTTGGGATTGGGT
TTGTTCAGTCGTTTGTGTCCAGGTCGAATAGTGATTCATGGGATATTGAGGATGAGAATGCTGAGCAGCTTATTATTGAGGAAGATAGCCGC
CGTGGACCATGTGCTGCTGCCACTACTCTTGGCTGCGTTGTGCCTCCACCACCTGTTCGAAAAATTGCCCCAATGGTTCCACAGCAACCTGCTA
AGGTAGCTTTGTCCCAAACGGAGAAGCCTTCGCCAATAATTATGCCAGCATTATCGGAAGATGACGAGGAGATTATACAATCTGTTGTTCAG
GGTAAAACACCATCATATTCGTTGGAATCAAAGCTTGGTGATTGTATGAGAGCTGCTTCGATTCGAAAAGAGGCGTTACAGAGGATTACAGG
GAAGTCATTGGAAGGGCTCCCATTGGAGGGATTTGACTATGAGTCTATTCTTGGACAGTGCTGTGAGATGCCTGTAGGATATGTGCAAATAC
CGGTGGGTATTGCTGGGCCTTTGTTGCTTGATGGGAGAGAGTACTCAGTGCCAATGGCAACTACAGAAGGATGTTTAGTGGCTAGCACCAAC
AGGGGTTGCAAGGCTATCTTTGTCTCTGGTGGC

1.2 3-Hydroxy-3-methylglutaryl coenzyme A reductase 2 (*HMG2*) sequences with intron
28
HMG2_cmm-7_seq1
TGGTGTCCAAAGGTGTACAAAATGTTCTTGATTACCTTCAGAATGAATATCCTGACATGGATGTCATCGGCATATCTGGTATTCCACCTACCCT
TTAGTTTTTCCTTAACTTCTCTGCTATGCATGGTGATCTGGTTCTGCTTTTTGTTCCCCCTAAAATAGAGATGTTTGTCTAATTAAAGTTATCAAT
AACTGTGACAGGGAACTTTTGCTCGGACAAGAAGCCATCAGCAGTTAATTGGATCGAGGGGAGAGGAAAGTCTGTAGTTTGTGAGGCAATT
ATTACAGAAGAGGTGGTGAAGAAAGTTCTAAAAACTGAGGTTGCTGCTCTTGTGGAGCTGAACATGCTTAAAAATCTTACTGGCTCTGCCAT
GGCTGGTGCCCTTGGTGGTTTCAATGCTCATGCCAGCAATATCGTCTCAGCTGTGTTTATTGCCACTGGTCAGGATCCAGCTCAGAACATAGA
GAGCTCGCACTGCATCACTATGATGGAGGCTGTAAATGATGGCAAGGACCTCCATATTTCTGT
HMG2_cmm-7_seq1-2
TGGTGTCCAAAGGTGTACAAAATGTTCTTGATTACCTTCAGAATGAATATCCTGACATGGATGTCATCGGCATATCTGGTATTCCACCTACCCT
TTAGTTTTTCCTTAACTTCTCTGCTATGCATGGTGATCTGGTTCTGCTTTTTGTTCCCCCTAAAATAGAGATGTTTGTCTAATTAAAGTTATCAAT
AACTGTGACAGGGAACTTTTGCTCGGACAAGAAGCCATCAGCAGTTAATTGGATCGAGGGGAGAGGAAAGTCTGTAGTTTGTGAGGCAATT
ATTACAGAAGAGGTGGTGAAGAAAGTTCTAAAAACTGAGGTTGCTGCTCTTGTGGAGCTGAACATGCTTAAAAATCTTACTGGCTCTGCCAT
GGCTGGTGCCCTTGGTGGTTTCAATGCTCATGCCAGCAATATCGTCTCAGCTGTGTTTATTGCCACTGGTCAGGATCCAGCTCAGAACATAGA
GAGCTCGCACTGCATCACTATGATGGAGGCTGTAAATGATGGCAAGGACCTCCATATTTCTGT
HMG2_cmm26_seq1
TGGTGTCCAAAGGTGTACAAAATGTTCTTGATTACCTTCAGAATGAATATCCTGACATGGATGTCATCGGCATATCTGGTATTCCACCTACCCT
TTAGTTTTTCCTTAACTTCTCTGCTATGCATGGTGATCTGGTTCTGCTTTTTGTTCCCCCTAAAATAGAGATGTTTGTCTAATTAAAGTTATCAAT
AACTGTGACAGGGAACTTTTGCTCGGACAAGAAGCCATCAGCAGTTAATTGGATCGAGGGGAGAGGAAAGTCTGTAGTTTGTGAGGCAATT
ATTACAGAAGAGGTGGTGAAGAAAGTTCTAAAAACTGAGGTTGCTGCTCTTGTGGAGCTGAACATGCTTAAAAATCTTACTGGCTCTGCCAT
GGCTGGTGCCCTTGGTGGTTTCAATGCTCATGCCAGCAATATCGTCTCAGCTGTGTTTATTGCCACTGGTCAGGATCCAGCTCAGAACATAGA
GAGCTCGCACTGCATCACTATGATGGAGGCTGTAAATGATGGCAAGGACCTCCATATTTCTGT
HMG2_cmm26_seq1-2
TGGTGTCCAAAGGTGTACAAAATGTTCTTGATTACCTTCAGAATGAATATCCTGACATGGATGTCATCGGCATATCTGGTATTCCACCTACCCT
TTAGTTTTTCCTTAACTTCTCTGCTATGCATGGTGATCTGGTTCTGCTTTTTGTTCCCCCTAAAATAGAGATGTTTGTCTAATTAAAGTTATCAAT
AACTGTGACAGGGAACTTTTGCTCGGACAAGAAGCCATCAGCAGTTAATTGGATCGAGGGGAGAGGAAAGTCTGTAGTTTGTGAGGCAATT
ATTACAGAAGAGGTGGTGAAGAAAGTTCTAAAAACTGAGGTTGCTGCTCTTGTGGAGCTGAACATGCTTAAAAATCTTACTGGCTCTGCCAT
GGCTGGTGCCCTTGGTGGTTTCAATGCTCATGCCAGCAATATCGTCTCAGCTGTGTTTATTGCCACTGGTCAGGATCCAGCTCAGAACATAGA
GAGCTCGCACTGCATCACTATGATGGAGGCTGTAAATGATGGCAAGGACCTCCATATTTCTGT
HMG2_dms54_seqR
TGGTGTCCAAAGGTGTACAAAATGTTCTTGATTACCTTCAGAATGAATATCCCGACATGGATGTCATCGGCATATCTGGTATTCCATCTACCTT
TATAGTAATTTGTTTCAAATAGGAGTAGCAAAATTTTTCAAAAAAATAATTTTCATAACGAAAAATATGGTGATCTGGTTCTGCTTTTTGTTCCC
CCTAAAATAGAGATGTTTGTCTAATTAAAGTTATCAATAACTGTGACAGGGAACTTTTGCTCGGACAAGAAGCCATCAGCAGTTAATTGGATC
GAGGGGAGAGGAAAGTCTGTAGTTTGTGAGGCAATTATCACAGAAGAGGTGGTGAAGAAAGTTCTGAAAACTGAAGTTGCTGCTCTTGTGG
AGCTGAACATGCTTAAAAATCTTACTGGCTCTGCCATGGCTGGTGCTCTTGGTGGTTTCAATGCCCATGCCAGCAATATCGTCTCTGCTGTGTT
TATAGCCACTGGTCAGGATCCAGCCCAGAACATAGAGAGCTCGCACTGCATCACTATGATGGAGGCTGTAAATGATGGCAAGGACCTCCATA
TTTCTGT
HMG2_dms54_seq2
TGGTGTCCAAAGGTGTACAAAATGTTCTTGATTACCTTCAGAATGAATATCCTGACATGGATGTCATCGGCATATCTGGTATTCCACCTACCCT
TTAGTTTTTCCTTAACTTCTCTGCTATGCATGGTGATCTGGTTCTGCTTTAATTTTTGTTCCCCCTAAAATAGAGATGTTTGTCTAATTAAAGTTA
TCAATAACTGTGACAGGGAACTTTTGCTCGGACAAGAAGCCATCAGCAGTTAATTGGATCGAGGGGAGAGGAAAGTCTGTAGTTTGTGAGG
CAATTATCACAGAAGAGGTGGTGAAGAAAGTTCTGAAAACTGAAGTTGCTGCTCTTGTGGAGCTGAACATGCTTAAAAATCTTACTGGCTCTG
CCATGGCTGGTGCTCTTGGTGGTTTCAATGCCCATGCCAGCAATATCGTCTCAGCTGTGTTTATAGCCACTGGTCAGGATCCAGCTCAGAACAT
AGAGAGCTCGCACTGCATCACTATGATGGAGGCTGTAAATGATGGCAAGGACCTCCATATTTCTGT
HMG2_dms78_seqR
TGGTGTCCAAAGGTGTACAAAATGTTCTTGATTACCTTCAGAATGAATATCCCGACATGGATGTCATCGGCATATCTGGTATTCCATCTACCTT
TATAGTAATTTGTTTCAAATAGGAGTAGCAAAATTTTTCAAAAAAATAATTTTCATAACGAAAAATATGGTGATCTGGTTCTGCTTTTTGTTCCC
CCTAAAATAGAGATGTTTGTCTAATTAAAGTTATCAATAACTGTGACAGGGAACTTTTGCTCGGACAAGAAGCCATCAGCAGTTAATTGGATC
GAGGGGAGAGGAAAGTCTGTAGTTTGTGAGGCAATTATCACAGAAGAGGTGGTGAAGAAAGTTCTGAAAACTGAAGTTGCTGCTCTTGTGG
AGCTGAACATGCTTAAAAATCTTACTGGCTCTGCCATGGCTGGTGCTCTTGGTGGTTTCAATGCCCATGCCAGCAATATCGTCTCTGCTGTGTT
TATAGCCACTGGTCAGGATCCAGCCCAGAACATAGAGAGCTCGCACTGCATCACTATGATGGAGGCTGTAAATGATGGCAAGGACCTCCATA
TTTCTGT
HMG2_dms78_seq2
TGGTGTCCAAAGGTGTACAAAATGTTCTTGATTACCTTCAGAATGAATATCCTGACATGGATGTCATCGGCATATCTGGTATTCCACCTACCCT
TTAGTTTTTCCTTAACTTCTCTGCTATGCATGGTGATCTGGTTCTGCTTTAATTTTTGTTCCCCCTAAAATAGAGATGTTTGTCTAATTAAAGTTA
TCAATAACTGTGACAGGGAACTTTTGCTCGGACAAGAAGCCATCAGCAGTTAATTGGATCGAGGGGAGAGGAAAGTCTGTAGTTTGTGAGG
CAATTATCACAGAAGAGGTGGTGAAGAAAGTTCTGAAAACTGAAGTTGCTGCTCTTGTGGAGCTGAACATGCTTAAAAATCTTACTGGCTCTG

CCATGGCTGGTGCTCTTGGTGGTTTCAATGCCCATGCCAGCAATATCGTCTCAGCTGTGTTTATAGCCACTGGTCAGGATCCAGCTCAGAACAT
AGAGAGCTCGCACTGCATCACTATGATGGAGGCTGTAAATGATGGCAAGGACCTCCATATTTCTGT
HMG2_spg55_seq1
TGGTGTCCAAAGGTGTACAAAATGTTCTTGATTACCTTCAGAATGAATATCCCGACATGGATGTCATCGGCATATCTGGTATTCCATCTACTAC
CAATAATTTTCATAACGAAAAACATGGTGATCTGTTTGTCTAATTAAAGTTATCTATAACTGTGACAGGGAACTTTTGCTCGGACAAGAAGCCA
GCAGCAGTTAATTGGATTGAGGGGAGAGGAAAGTCTGTAGTTTGTGAGGCAATCATCACAGAAGAGGTGGTGAAGAAAGTTTTGAAAACTG
AGGTTGCTGCTCTTGTGGAGCTGAACATGCTTAAAAATCTTACTGGCTCTGCCATGGCTGGTGCCCTTGGTGGTTTCAATGCCCACGCCAGCA
ATATCGTCTCAGCTGTGTTTATAGCCACTGGTCAGGATCCAGCTCAGAACATAGAGAGCTCGCACTGCATCACTATGATGGAGGCTGTAAATG
ATGGCAAGGACCTCCATATTTCTGT
HMG2_spg55_seq1-2
TGGTGTCCAAAGGTGTACAAAATGTTCTTGATTACCTTCAGAATGAATATCCCGACATGGATGTCATCGGCATATCTGGTATTCCATCTACTAC
CAATAATTTTCATAACGAAAAACATGGTGATCTGTTTGTCTAATTAAAGTTATCTATAACTGTGACAGGGAACTTTTGCTCGGACAAGAAGCCA
GCAGCAGTTAATTGGATTGAGGGGAGAGGAAAGTCTGTAGTTTGTGAGGCAATCATCACAGAAGAGGTGGTGAAGAAAGTTTTGAAAACTG
AGGTTGCTGCTCTTGTGGAGCTGAACATGCTTAAAAATCTTACTGGCTCTGCCATGGCTGGTGCCCTTGGTGGTTTCAATGCCCACGCCAGCA
ATATCGTCTCAGCTGTGTTTATAGCCACTGGTCAGGATCCAGCTCAGAACATAGAGAGCTCGCACTGCATCACTATGATGGAGGCTGTAAATG
ATGGCAAGGACCTCCATATTTCTGT
HMG2_spg74_seqR
TGGTGTCCAAAGGTGTACAAAATGTTCTTGATTACCTTCAGAATGAATATCCCGACATGGATGTCATCGGCATATCTGGTATTCCATCTACCTT
TATAGTAATTTGTTTCAAATAGGAGTAGCAAAATTTTTCAAAAAAATAATTTTCATAACGAAAAATATGGTGATCTGGTTCTGCTTTTTGTTCCC
CCTAAAATAGAGATGTTTGTCTAATTAAAGTTATCAATAACTGTGACAGGGAACTTTTGCTCGGACAAGAAGCCATCAGCAGTTAATTGGATC
GAGGGGAGAGGAAAGTCTGTAGTTTGTGAGGCAATTATCACAGAAGAGGTGGTGAAGAAAGTTCTGAAAACTGAAGTTGCTGCTCTTGTGG
AGCTGAACATGCTTAAAAATCTTACTGGCTCTGCCATGGCTGGTGCTCTTGGTGGTTTCAATGCCCATGCCAGCAATATCGTCTCTGCTGTGTT
TATAGCCACTGGTCAGGATCCAGCCCAGAACATAGAGAGCTCGCACTGCATCACTATGATGGAGGCTGTAAATGATGGCAAGGACCTCCATA
TTTCTGT
HMG2_spg74_seq2
TGGTGTCCAAAGGTGTACAAAATGTTCTTGATTACCTTCAGAATGAATATCCCGACATGGATGTCATCGGCATATCTGGTATTCCATCTACTAG
CAAAAATTTTCATAACGAAAAACATGGTGATCTGTTTGTCTAATTAAAGTTATCTATAACTGTGACAGGGAACTTTTGCTCGGACAAGAAGCC
AGCAGCAGTTAATTGGATTGAGGGGAGAGGAAAGTCTGTAGTTTGTGAGGCAATCATCACAGAAGAGGTGGTGAAGAAAGTTTTGAAAACT
GAGGTTGCTGCTCTTGTGGAGCTGAACATGCTTAAAAATCTTACTGGCTCTGCCATGGCTGGTGCCCTTGGTGGTTTCAATGCCCACGCCAGC
AATATCGTCTCAGCTGTGTTTATAGCCACTGGTCAGGATCCAGCTCAGAACATAGAGAGCTCGCACTGCATCACTATGATGGAGGCTGTAAAT
GATGGCAAGGACCTCCATATTTCTGT
HMG2_spl16_seq1
TGGTGTCCAAAGGTGTACAAAATGTTCTTGATTACCTTCAGAATGAATATCCCGACATGGATGTCATCGGCATATCTGGTATATCATCTACCTT
TATAGTAATTTGTTTCAAATAGGAGTACCAAAAATAATTTTCATAACGAAAAATATGGTGATCTGGTTCTGCTTTAATTTTTGTTCCCCCTAAAA
TAGAGATGTTTGTCTAATTAAAGTTATCAATAACTGTGACAGGGAACTTTTGCTCGGACAAGAAGCCATCAGCAGTTAATTGGATCGAGGGG
AGAGGAAAGTCTGTAGTTTGTGAGGCAATTATCACAGAAGAGGTGGTGAAGAAAGTTCTGAAAACTGAAGTTGCTGCTCTTGTGGAGCTGA
ACATGCTTAAAAATCTTACTGGCTCTGCCATGGCTGGTGCTCTTGGTGGTTTCAATGCCCATGCCAGCAATATCGTCTCAGCTGTGTTTATAGC
CACTGGTCAGGATCCAGCTCAGAACATAGAGAGCTCGCACTGCATCACTATGATGGAGGCTGTAAATGATGGCAAGGACCTCCATATTTCTG
T
HMG2_spl16_seq1-2
TGGTGTCCAAAGGTGTACAAAATGTTCTTGATTACCTTCAGAATGAATATCCCGACATGGATGTCATCGGCATATCTGGTATATCATCTACCTT
TATAGTAATTTGTTTCAAATAGGAGTACCAAAAATAATTTTCATAACGAAAAATATGGTGATCTGGTTCTGCTTTAATTTTTGTTCCCCCTAAAA
TAGAGATGTTTGTCTAATTAAAGTTATCAATAACTGTGACAGGGAACTTTTGCTCGGACAAGAAGCCATCAGCAGTTAATTGGATCGAGGGG
AGAGGAAAGTCTGTAGTTTGTGAGGCAATTATCACAGAAGAGGTGGTGAAGAAAGTTCTGAAAACTGAAGTTGCTGCTCTTGTGGAGCTGA
ACATGCTTAAAAATCTTACTGGCTCTGCCATGGCTGGTGCTCTTGGTGGTTTCAATGCCCATGCCAGCAATATCGTCTCAGCTGTGTTTATAGC
CACTGGTCAGGATCCAGCTCAGAACATAGAGAGCTCGCACTGCATCACTATGATGGAGGCTGTAAATGATGGCAAGGACCTCCATATTTCTG
T
HMG2_spl81_seq2
TGGTGTCCAAAGGTGTACAAAATGTTCTTGATTACCTTCAGAATGAATATCCCGACATGGATGTCATCGGCATATCTGGTATTCCATCTACCTT
TATAGTAATTTGTTTCAAATAGGAGTACCAAAATTTTCAAAAAAATAATTTTCCTAACGAAAAATATGGTGATCTGGTTCTGCTTTTTGTTTCCC
CTAAAATAGAGATGTTTGTCTAATTAAAGTTATCAATAACTGTGACAGGGAACTTTTGCTCGGACAAGAAGCCATCAGCAGTTAATTGGATCG
AGGGGAGAGGAAAGTCTGTAGTTTGTGAGGCAATTATCACAGAAGAGGTGGTGAAGAAAGTTCTGAAAACTGAAGTTGCTGCTCTTGTGGA
GCTGAACATGCTTAAAAATCTTACTGGCTCTGCCATGGCTGGTGCTCTTGGTGGTTTCAATGCCCATGCCAGCAATATCGTCTCTGCTGTGTTT
ATAGCCACTGGTCAGGATCCAGCCCAGAACATAGAGAGCTCGCACTGCATCACTATGATGGAGGCTGTAAATGATGGCAAGGACCTCCATAT
TTCTGT
HMG2_spl81_seq2-2
TGGTGTCCAAAGGTGTACAAAATGTTCTTGATTACCTTCAGAATGAATATCCCGACATGGATGTCATCGGCATATCTGGTATTCCATCTACCTT
TATAGTAATTTGTTTCAAATAGGAGTACCAAAATTTTCAAAAAAATAATTTTCCTAACGAAAAATATGGTGATCTGGTTCTGCTTTTTGTTTCCC

CTAAAATAGAGATGTTTGTCTAATTAAAGTTATCAATAACTGTGACAGGGAACTTTTGCTCGGACAAGAAGCCATCAGCAGTTAATTGGATCG
AGGGGAGAGGAAAGTCTGTAGTTTGTGAGGCAATTATCACAGAAGAGGTGGTGAAGAAAGTTCTGAAAACTGAAGTTGCTGCTCTTGTGGA
GCTGAACATGCTTAAAAATCTTACTGGCTCTGCCATGGCTGGTGCTCTTGGTGGTTTCAATGCCCATGCCAGCAATATCGTCTCTGCTGTGTTT
ATAGCCACTGGTCAGGATCCAGCCCAGAACATAGAGAGCTCGCACTGCATCACTATGATGGAGGCTGTAAATGATGGCAAGGACCTCCATAT
TTCTGT
HMG2_sto40_seqR
TGGTGTCCAAAGGTGTACAAAATGTTCTTGATTACCTTCAGAATGAATATCCCGACATGGATGTCATCGGCATATCTGGTATTCCATCTACCTT
TATAGTAATTTGTTTCAAATAGGAGTAGCAAAATTTTTCAAAAAAATAATTTTCATAACGAAAAATATGGTGATCTGGTTCTGCTTTTTGTTCCC
CCTAAAATAGAGATGTTTGTCTAATTAAAGTTATCAATAACTGTGACAGGGAACTTTTGCTCGGACAAGAAGCCATCAGCAGTTAATTGGATC
GAGGGGAGAGGAAAGTCTGTAGTTTGTGAGGCAATTATCACAGAAGAGGTGGTGAAGAAAGTTCTGAAAACTGAAGTTGCTGCTCTTGTGG
AGCTGAACATGCTTAAAAATCTTACTGGCTCTGCCATGGCTGGTGCTCTTGGTGGTTTCAATGCCCATGCCAGCAATATCGTCTCTGCTGTGTT
TATAGCCACTGGTCAGGATCCAGCCCAGAACATAGAGAGCTCGCACTGCATCACTATGATGGAGGCTGTAAATGATGGCAAGGACCTCCATA
TTTCTGT
HMG2_sto40_seqR-2
TGGTGTCCAAAGGTGTACAAAATGTTCTTGATTACCTTCAGAATGAATATCCCGACATGGATGTCATCGGCATATCTGGTATTCCATCTACCTT
TATAGTAATTTGTTTCAAATAGGAGTAGCAAAATTTTTCAAAAAAATAATTTTCATAACGAAAAATATGGTGATCTGGTTCTGCTTTTTGTTCCC
CCTAAAATAGAGATGTTTGTCTAATTAAAGTTATCAATAACTGTGACAGGGAACTTTTGCTCGGACAAGAAGCCATCAGCAGTTAATTGGATC
GAGGGGAGAGGAAAGTCTGTAGTTTGTGAGGCAATTATCACAGAAGAGGTGGTGAAGAAAGTTCTGAAAACTGAAGTTGCTGCTCTTGTGG
AGCTGAACATGCTTAAAAATCTTACTGGCTCTGCCATGGCTGGTGCTCTTGGTGGTTTCAATGCCCATGCCAGCAATATCGTCTCTGCTGTGTT
TATAGCCACTGGTCAGGATCCAGCCCAGAACATAGAGAGCTCGCACTGCATCACTATGATGGAGGCTGTAAATGATGGCAAGGACCTCCATA
TTTCTGT
HMG2_sto61_seqR
TGGTGTCCAAAGGTGTACAAAATGTTCTTGATTACCTTCAGAATGAATATCCCGACATGGATGTCATCGGCATATCTGGTATTCCATCTACCTT
TATAGTAATTTGTTTCAAATAGGAGTAGCAAAATTTTTCAAAAAAATAATTTTCATAACGAAAAATATGGTGATCTGGTTCTGCTTTTTGTTCCC
CCTAAAATAGAGATGTTTGTCTAATTAAAGTTATCAATAACTGTGACAGGGAACTTTTGCTCGGACAAGAAGCCATCAGCAGTTAATTGGATC
GAGGGGAGAGGAAAGTCTGTAGTTTGTGAGGCAATTATCACAGAAGAGGTGGTGAAGAAAGTTCTGAAAACTGAAGTTGCTGCTCTTGTGG
AGCTGAACATGCTTAAAAATCTTACTGGCTCTGCCATGGCTGGTGCTCTTGGTGGTTTCAATGCCCATGCCAGCAATATCGTCTCTGCTGTGTT
TATAGCCACTGGTCAGGATCCAGCCCAGAACATAGAGAGCTCGCACTGCATCACTATGATGGAGGCTGTAAATGATGGCAAGGACCTCCATA
TTTCTGT
HMG2_sto61_seq2
TGGTGTCCAAAGGTGTACAAAATGTTCTTGATTACCTTCAGAATGAATATCCCGACATGGATGTCATCGGCATATCTGGTATTCCATCTACCTT
TATAGTAATTTGTTTCAAATAGGAGTACCAAAATTTTTCAAAAAAATAATTTTCATAACGAAAAATATGGTGATCTGGTTCTGCTTTTTGTTCCC
CCTAAAATAGAGATGTTTGTCTAATTAAAGTTATCAATAACTGTGACAGGGAACTTTTGCTCGGACAAGAAGCCAGCAGCAGTTAATTGGATC
GAGGGGAGAGGAAAGTCTGTAGTTTGTGAGGCAACTATCACAGAAGATGTGGTGAAGAAAGTTCTGAAAACTGAAGTTGCTGCTCTTGTGG
AGCTGAACATGCTTAAAAATCTTACTGGCTCTGCCATGGCTGGTGCCCTTGGTGGTTTCAATGCCCACGCCAGCAATATCGTCTCAGCTTTGTT
TATAGCCACTGGTCAGGATCCAGCTCAGAACATAGAGAGCTCGCACTGCATCACTATGATGGAGGCTGTAAATGATGGCAAGGACCTCCATA
TTTCTGT
HMG2_chc80-1_seq1
TGGTGTCCAAAGGTGTACAAAATGTTCTTGATTACCTTCAGAATGAATATCCCGACATGGATGTCATCGGCATATCTGGTATTCCATCTACTAG
CAAAAATTTTCATAACGAAAAACATGGTGATCTGTTTGTCTAATTAAAGTTATCTATAACTGTGACAGGGAACTTTTGCTCGGACAAGAAGCC
AGCAGCAGTTAATTGGATTGAGGGGAAAGGAAAGTCTGTAGTTTGTGAGGCAATCATCACAGAAGAGGTGGTGAAGAAAGTTTTGAAAACT
GAGGTTTCTGCTCTTGTGGAGCTGAACATGCTTAAAAATCTTACTGGCTCTGCCATGGCTGGTGCCCTTGGTGGTTTCAATGCCCACGCCAGC
AATATCGTCTCAGCTGTGTTTATAGCCACTGGTCAGGATCCAGCTCAGAACATAGAGAGCTCGCACTGCATCACTATGATGGAGGCTGTAAAT
GATGGCAAGGACCTCCATATTTCTGT
HMG2_chc80-1_seq2
TGGTGTCCAAAGGTGTACAAAATGTTCTTGATTACCTTCAGAATGAATATCCCGACATGGATGTCATCGGCATATCTGGTATTCCATCTACTAG
CAAAAATTTTCATAACGAAAAACATGGTGATCTGTTTGTCTAATTAAAGTTATCTATAACTGTGACAGGGAACTTTTGCTCGGACAAGAAGCC
AGCAGCAGTTAATTGGATTGAGGGGAGAGGAAAGTCTGTAGTTTGTGAGGCAATCATCACAGAAGAGGTGGTGAAGAAAGTTTTGAAAACT
GAGGTTTCTGCTCTTGTGGAGCTGAACATGCTTAAAAATCTTACTGGCTCTGCCATGGCTGGTGCCCTTGGTGGTTTCAATGCCCACGCCAGC
AATATCGTCTCAGCTGTGTTTATAGCCACTGGTCAGGATCCAGCTCAGAACATAGAGAGCTCGCACTGCATCACTATGATGGAGGCTGTAAAT
GATGGCAAGGACCTCCATATTTCTGT
HMG2_phuDH_seq1
TGGTGTCCAAAGGTGTACAAAATGTTCTTGATTACCTTCAGAATGAATATCCCGACATGGATGTCATCGGCATATCTGGTATTCCATCTACTAG
CAAAAATTTTCATAACGAAAAACATGGTGATCTGTTTGTCTAATTAAAGTTATCTATAACTGTGACAGGGAACTTTTGCTCGGACAAGAAGCC
AGCAGCAGTTAATTGGATTGAGGGGAGAGGAAAGTCTGTAGTTTGTGAGGCAATCATCACAGAAGAGGTGGTGAAGAAAGTTTTGAAAACT
GAGGTTGCTGCTCTTGTGGAGCTGAACATGCTTAAAAATCTTACTGGCTCTGCCATGGCTGGTGCCCTTGGTGGTTTCAATGCCCACGCCAGC
AATATCGTCTCAGCTGTGTTTATAGCCACTGGTCAGGATCCAGCTCAGAACATAGAGAGCTCGCACTGCATCACTATGATGGAGGCTGTAAAT
GATGGCAAGGACCTCCATATTTCTGT

HMG2_phuDH_seq2
TGGTGTCCAAAGGTGTACAAAATGTTCTTGATTACCTTCAGAATGAATATCCTGACATGGATGTCATCGGCATATCTGGTATTCCACCTACCCT
TTAGTTTTTCCTTAACTTCTCTGCTATGCATGGTGATCTGGTTCTGCTTTAATTTTTGTTCCCCCTAAAATAGAGATGTTTGTCTAATTAAAGTTA
TCAATAACTGTGACAGGGAACTTTTGCTCGGACAAGAAGCCATCAGCAGTTAATTGGATCGAGGGGAGAGGAAAGTCTGTAGTTTGTGAGG
CAATTATCACAGAAGAGGTGGTGAAGAAAGTTCTGAAAACTGAAGTTGCTTCTCTTGTGGAGCTGAACATGCTTAAAAATCTTACTGGCTCTG
CCATGGCTGGTGCTCTTGGTGGTTTCAATGCCCATGCCAGCAATATCGTCTCAGCTGTGTTTATAGCCACTGGTCAGGATCCAGCTCAGAACAT
AGAGAGCTCGCACTGCATCACTATGATGGAGGCTGTAAATGATGGCAAGGACCTCCATATTTCTGT
HMG2_phuDM_seq2
TGGTGTCCAAAGGTGTACAAAATGTTCTTGATTACCTTCAGAATGAATATCCTGACATGGATGTCATCGGCATATCTGGTATTCCACCTACCCT
TTAGTTTTTCCTTAACTTCTCTGCTATGCATGGTGATCTGGTTCTGCTTTAATTTTTGTTCCCCCTAAAATAGAGATGTTTGTCTAATTAAAGTTA
TCAATAACTGTGACAGGGAACTTTTGCTCGGACAAGAAGCCATCAGCAGTTAATTGGATCGAGGGGAGAGGAAAGTCTGTAGTTTGTGAGG
CAATTATCACAGAAGAGGTGGTGAAGAAAGTTCTGAAAACTGAAGTTGCTTCTCTTGTGGAGCTGAACATGCTTAAAAATCTTACTGGCTCTG
CCATGGCTGGTGCTCTTGGTGGTTTCAATGCCCATGCCAGCAATATCGTCTCAGCTGTGTTTATAGCCACTGGTCAGGATCCAGCTCAGAACAT
AGAGAGCTCGCACTGCATCACTATGATGGAGGCTGTAAATGATGGCAAGGACCTCCATATTTCTGT
HMG2_phuDM_seq2-2
TGGTGTCCAAAGGTGTACAAAATGTTCTTGATTACCTTCAGAATGAATATCCTGACATGGATGTCATCGGCATATCTGGTATTCCACCTACCCT
TTAGTTTTTCCTTAACTTCTCTGCTATGCATGGTGATCTGGTTCTGCTTTAATTTTTGTTCCCCCTAAAATAGAGATGTTTGTCTAATTAAAGTTA
TCAATAACTGTGACAGGGAACTTTTGCTCGGACAAGAAGCCATCAGCAGTTAATTGGATCGAGGGGAGAGGAAAGTCTGTAGTTTGTGAGG
CAATTATCACAGAAGAGGTGGTGAAGAAAGTTCTGAAAACTGAAGTTGCTTCTCTTGTGGAGCTGAACATGCTTAAAAATCTTACTGGCTCTG
CCATGGCTGGTGCTCTTGGTGGTTTCAATGCCCATGCCAGCAATATCGTCTCAGCTGTGTTTATAGCCACTGGTCAGGATCCAGCTCAGAACAT
AGAGAGCTCGCACTGCATCACTATGATGGAGGCTGTAAATGATGGCAAGGACCTCCATATTTCTGT
HMG2_tbr_seq1
TGGTGTCCAAAGGTGTACAAAATGTTCTTGATTACCTTCAGAATGAATATCCCGACATGGATGTCATCGGCATATCTGGGAACTTTTGCTCGG
ACAAGAAGCCAGCAGCAGTTAATTGGATCGAGGGGAGAGGAAAGTCTGTAGTTTGTGAGGCAATTATCACAGAAGAGGTGGTGAAGAAAG
TTCTGAAAACTGAAGTTGCTGCTCTTGTGGAGCTGAACATGCTTAAAAATCTTACTGGCTCTGCCATGGCTGGTGCCCTTGGTGGTTTCAATGC
CCATGCCAGCAATATCGTCTCAGCTGTGTTTATAGCCACTGGTCAGGATCCAGCTCAGAACATAGAGAGCTCGCACTGCATCACTATGATGGA
GGCTGTAAATGATGGCAAGGACCTCCATATTTCTGT
HMG2_tbr_seq1-2
TGGTGTCCAAAGGTGTACAAAATGTTCTTGATTACCTTCAGAATGAATATCCCGACATGGATGTCATCGGCATATCTGGGAACTTTTGCTCGG
ACAAGAAGCCAGCAGCAGTTAATTGGATCGAGGGGAGAGGAAAGTCTGTAGTTTGTGAGGCAATTATCACAGAAGAGGTGGTGAAGAAAG
TTCTGAAAACTGAAGTTGCTGCTCTTGTGGAGCTGAACATGCTTAAAAATCTTACTGGCTCTGCCATGGCTGGTGCCCTTGGTGGTTTCAATGC
CCATGCCAGCAATATCGTCTCAGCTGTGTTTATAGCCACTGGTCAGGATCCAGCTCAGAACATAGAGAGCTCGCACTGCATCACTATGATGGA
GGCTGTAAATGATGGCAAGGACCTCCATATTTCTGT

1.3 2,3-squalene epoxidase (SQE) sequences with introns
26
SQE_cmm7_seq1
TTGGGGTTCGTTGCAGTTTTCTGTTTTTTCATTCAGAAAAATGGTGGCCGCAGCAAAAGTAAAATTGATGATTCGGCAACTAGCACCACTACA
ACTACCGTGTACAGTGGAGAGTCCAGAACAAAGGATGGAAACGACGACGTTGATATCATCATCGTCGGTGCCGGAGTTGCCGGTGCTGCTCT
TGCTCACACTCTTGGCAAGGTATTGTTTCTTTCTTTGTAATCCGTTTTTTAAAAGTTACTATTCTACTTTGGTCCTTCAACTATTTTTTATGTACAT
ATGTGAGCAAACCGTAATTGTGTAGTGAGAAGTAATTAGATTGATTGATTTACGTATCTGAAGGCATAGTTGAGTGGTTATTCATAAAAAACT
TGAACTTGATTGTTAATTTGATGAAATCAGAGAAATGAAAGATGCAATGATGATGAAAGCGTTTGGTGTGACTGCAAATTTTTTTGTTGGAGT
TCTTTGTTGACAATTCTCTATGAAAAAAAAATTCTTTTTTGTGAGATTTATGATTATACATTTGGGACTACAGGAAGGGCGTCGTGTAAAAGTAA
TTGAAAGAGATTTGACAGAGCCTGATCGAATTGTTGGAGAACTCCTACAACCGGGTGGTTACCTCAAATTGCAGGAGTTGGGATTGGAAGGT
AAAGTTCCTAAGTGATGTGATTTGTGTCAATTCTGATTAATTTACTCTCTTGAAGAGAAAAAAAAGGAAGTTAGTCTTACACTTTTAGCTTCTC
GTTTGTCTCTTTTTCGGCATTTCTCATATTTGTATTTTGGTCTACTGCAGATTGTGTGGAGAAAATTGATGCTCAACGAGTGTTTGGGTATGCCC
TTTTCAAGGATGGAAAGAGTACACGTCTTTCTTATCCCCTGG
>SQE_cmm7_seq2
TTGGGGTTCGTTGCAGTTTTCTGTTTTTTCATTCAGAAAAATGGTGGCCGCAGCAAAAGTAAAATTGATGATTCGGCAACTAGCACCACTACA
ACTACCGTGTACAGTGGAGAGTCCAGAACAAAGGATGGAAACGACGACGTTGATATCATCATCGTCGGTGCCGGAGTTGCCGGTGCTGCTCT
TGCTCACACTCTTGGCAAGGTATTGTTTCTTTCTTTGTAATCCGTTTTTTAAAAGTTACTATTCTACTTTGGTCCTTCAACTATTTTTTATGTACAT
ATGTGAGCAAACCGTAATTGTGTAGTGAGAAGTAATTAGATTGATTGATTTACGTATCTGAAGGCATAGTTGAGTGGTTATTCATAAAAAACT
TGAACTTGATTGTTAATTTGATGAAATCAGAGAAATGAAAGATGCATTGATGATGAAAGCGTTTGGTGTGACTGCAATTTTTTTTATTGGAGT
TCTTTGTTGACAATTCGCTATGAAAAAAAATTCTTTTTCGTGAGATTTATGATTATACATTTGGGACTACAGGAAGGGCGTCGTGTAAAAGTAAT
TGAAAGAGATTTGACAGAGCCTGATCGAATTGTTGGAGAACTCCTACAACCGGGTGGTTACCTCAAATTGCAGGAGTTGGGATTGGAAGGTA
AAGTTCCTAAGTGATGTGATTTGTGTCAATTCTGATTAATTTACTCTCTTGAAGAGCAAAAAAAGGAAGTTAGTCTTACACTTTTAGCTTCTCGT
TTGTCTCTTTTTCGGCATTTCTCATATTTGTATTTTGGTCTACTGCAGATTGTGTGGAGAAAATTGATGCTCAACGAGTGTTTGGGTATGCCCTT
TTCAAGGATGGAAAGAGTACACGTCTTTCTTATCCCCTGG
SQE_cmm26_seq2
TTGGGGTTCGTTGCAGTTTTCTGTTTTTTCATTCAGAAAAATGGTGGCCGCAGCAAAAGTAAAATTGATGATTCGGCAACTAGCACCACTACA
ACTACCGTGTACAGTGGAGAGTCCAGAACAAAGGATGGAAACGACGACGTTGATATCATCATCGTCGGTGCCGGAGTTGCCGGTGCTGCTCT
TGCTCACACTCTTGGCAAGGTATTGTTTCTTTCTTTGTAATCCGTTTTTTAAAAGTTACTATTCTACTTTGGTCCTTCAACTATTTTTTATGTACAT
ATGTGAGCAAACCGTAATTGTGTAGTGAGAAGTAATTAGATTGATTGATTTACGTATCTGAAGGCATAGTTGAGTGGTTATTCATAAAAAACT
TGAACTTGATTGTTAATTTGATGAAATCAGAGAAATGAAAGATGCATTGATGATGAAAGCGTTTGGTGTGACTGCAATTTTTTTTATTGGAGT
TCTTTGTTGACAATTCGCTATGAAAAAAAATTCTTTTTCGTGAGATTTATGATTATACATTTGGGACTACAGGAAGGGCGTCGTGTAAAAGTAAT
TGAAAGAGATTTGACAGAGCCTGATCGAATTGTTGGAGAACTCCTACAACCGGGTGGTTACCTCAAATTGCAGGAGTTGGGATTGGAAGGTA
AAGTTCCTAAGTGATGTGATTTGTGTCAATTCTGATTAATTTACTCTCTTGAAGAGCAAAAAAAGGAAGTTAGTCTTACACTTTTAGCTTCTCGT
TTGTCTCTTTTTCGGCATTTCTCATATTTGTATTTTGGTCTACTGCAGATTGTGTGGAGAAAATTGATGCTCAACGAGTGTTTGGGTATGCCCTT
TTCAAGGATGGAAAGAGTACACGTCTTTCTTATCCCCTGG
SQE_cmm26_seq3
TTGGGGTTCGTTGCAGTTTTCTGTTTTTTCATTCAGAAAAATGGTGGCCGCAGCAAAAGTAAAATTGATGACTCGGCAACTAGCACCACTACA
ACTACCGTGTACAGTGGAGAGTCCAGAACAAAGGATGGAAACGACGACGTTGATATCATCATCGTCGGTGCCGGAGTTGCCGGTGCTGCTCT
TGCTCACACTCTTGGCAAGGTATTGTTTCTTTCTTTGTAATCCGTTTTTTAAAAGTTACTATTCTACTTTGGTCCTTCAACTATTTTTTATGTACAT
ATGTGAGCAAACCGTAATTGTGTAGTGAGAAGTAATTAGATTGATTGATTTACGTATCTGAAGGCATAGTTGAGTGGTTATTCATAAAAAACT
TGAACTTGATTGTTAATTTGATGAAATCAGAGAAATGAAAGATGCATTGATGATGAAAGCGTTTGGTGTGACTGCAAATTTTTTTATTGGAGT
TCTTTGTTGACAATTCGCTATGAAAAAAAATTCTTTTTCGTGAGATTTAAGATTATACATTTGGGACTACAGGAAGGGCGTCGTGTAAAAGTAAT
TGAAAGAGATTTGACAGAGCCTGATCGAATTGTTGGAGAACTCCTACAACCGGGTGGTTACCTCAAATTGCAGGAGTTGGGATTGGAAGGTA
AAGTTCCTAAGTGATGTGATTTGTGTCAATTCTGATTAATTTACTCTCTTGAAGAGAAAAAAAGGGAAGTTAGTCTTACACTTTTAGCTTCTCG
TTTGTCTCTTTTTCGGCATTTCTCATATTTGTATTTTGGTCTACTGCAGATTGTGTGGAGAAAATTGATGCTCAACGAGTGTTTGGGTATGCCCT
TTTCAAGGATGGAAAGAGTACACGTCTTTCTTATCCCCTGG
SQE_dms54_seq1
TTGGGGTTCGTTGCAGTTTTCTGTTTTTTCATTCAGAGAAATGGTGGCTGCAGCAAAAGTAAAATTGATGACTCAGCAACTAGCACCACTACA
ACTACGGTGTACAGTGGAAAGTCCCGATCAAAGGATGGAAACGACGACGTTGATATCATCATCGTTGGTGCCGGAGTTGCCGGTGCTGCTCT
TGCTCACACTCTTGGCAAGGTATTGTTTCTTTCTTTGTAATCCTTTTTTTTAAAGTTATTGTTCTACTTTGATCCTTTAACTATTTTTATGTACATAT
GTGAGCAAACCGTAATTGTGAAGTGAGAAGTAATTAGACTGATTGATTTACGTATCTGAAGACATAGTTGATGTGGTTATTCATAAAAGACTT
GAACTTGATTGTTAATTTGATGAAACCAGAGAAATGAAAGATGCATTGATGATGAAAGCATTTGGTGTGACTGCAAATTTTTTTATTGGAGTT
CTTTGTTGACAATTCTCTATGAAAAAAAAATTCTTTTTTGTGAGATTTATGATTATACATTTGGGACTACAGGAAGGGCGTCGTGTAAAAGTAAT
TGAAAGAGATTTGACAGAGCCTGATCGAATTGTTGGAGAACTCCTACAACCGGGTGGTTACCTCAAATTGCAGGAGTTGGGATTGGAAGGTA
AAGTTCCTAAGTGATGTGATTTGTGTCAATTCTGGTTAATTTACTCTCTTGAAGAGAAAAAAAAGGAAGTTAGTCTTACACTTTTAGCTTCTCG
TCTGTCTCTTTTTTGGCATTTCTCATATTTGTATTTTGGTCTACTGCAGATTGTGTGGAGAAAATTGATGCTCAACGAGTGTTTGGGTATGCCCT
TTTCAAGGATGGAAAGAGTACACGTCTTTCTTATCCCCTGG
SQE_dms54_seq2

TTGGGGTTCGTTGCAGTTTTCTGTTTTTTCATTCAGAGAAATGGTGGCTGCAGCAAAAGTAAAATTGATGACTCAGCAACTAGCACCACTACA
ACTACGGTGTACAGTGGAAAGTCCCGATCAAAGGATGGAAACGACGACGTTGATATCATCATCGTCGGTGCCGGAGTTGCCGGTGCTGCTCT
TGCTCACACTCTTGGCAAGGTATTGTTTCTTTCTTTGTAATCCGTTTTTTTAAAGTTATATTCTACTTGGTCCTTCAACTATTTTTTATGTACATAT
GTGAGCAAACCGTAATTGTGTAGTGAGAAGTAATTAGATTGATTGATTTACGTATCTGAAGGCATAGTTGATGTGGTTATTCATAAAAAACTT
GAACTTGATTGTTAATTTGATGAAATCAGAGAAATGAAAGATCCATTGATGATGAAAGCGTTTGGTGTGACTGCAAATAATTTTATTGGAGTT
CTTTGTTGACAATTCTCTATAAAAAAAAATTATTTTTTTGTGAGATTTATGATTATACATTTGGGACTACAGGAAGGGCGTCGTGTAAAAGTAAT
TGAAAGAGATTTGACAGAGCCTGATCGAATTGTTGGAGAACTCCTACAACCGGGTGGTTACCTCAAATTGCAGGAGTTGGGATTGGAAGGTA
AAGTTCCTAAGTGATGTGATTTGTGTCAATTCTGGTTAATTTACTCTCTTGAAGAGAAAAAAAAGGAAGTTAGTCTTACGCTTTTAGCTTCTCG
TCTGTCTCTTTTTCGGCATTTCTCATATTTGTATTTTGGTCTACTGCAGATTGTGTGGAGAAAATTGATGCTCAACGAGTGTTTGGGTATGCCCT
TTTCAAGGATGGAAAGAGTACACGTCTTTCTTATCCCCTGG
SQE_dms78_seq1
TTGGGGTTCGTTGCAGTTTTCTGTTTTTTCATTCAGAGAAATGGTGGCTGCAGCAAAAGTAAAATTGATGACTCAGCAACTAGCACCACTACA
ACTACGGTGTACAGTGGAAAGTCCCGATCAAAGGATGGAAACGACGACGTTGATATCATCATCGTTGGTGCCGGAGTTGCCGGTGCTGCTCT
TGCTCACACTCTTGGCAAGGTATTGTTTCTTTCTTTGTAATCCTTTTTTTTAAAGTTATTGTTCTACTTTGATCCTTTAACTATTTTTATGTACATAT
GTGAGCAAACCGTAATTGTGAAGTGAGAAGTAATTAGACTGATTGATTTACGTATCTGAAGACATAGTTGATGTGGTTATTCATAAAAGACTT
GAACTTGATTGTTAATTTGATGAAACCAGAGAAATGAAAGATGCATTGATGATGAAAGCATTTGGTGTGACTGCAAATTTTTTTATTGGAGTT
CTTTGTTGACAATTCTCTATGAAAAAAAAATTCTTTTTTGTGAGATTTATGATTATACATTTGGGACTACAGGAAGGGCGTCGTGTAAAAGTAAT
TGAAAGAGATTTGACAGAGCCTGATCGAATTGTTGGAGAACTCCTACAACCGGGTGGTTACCTCAAATTGCAGGAGTTGGGATTGGAAGGTA
AAGTTCCTAAGTGATGTGATTTGTGTCAATTCTGGTTAATTTACTCTCTTGAAGAGAAAAAAAAGGAAGTTAGTCTTACACTTTTAGCTTCTCG
TCTGTCTCTTTTTTGGCATTTCTCATATTTGTATTTTGGTCTACTGCAGATTGTGTGGAGAAAATTGATGCTCAACGAGTGTTTGGGTATGCCCT
TTTCAAGGATGGAAAGAGTACACGTCTTTCTTATCCCCTGG
SQE_dms78_seq2
TTGGGGTTCGTTGCAGTTTTCTGTTTTTTCATTCAGAGAAATGGTGGCTGCAGCAAAAGTAAAATTGATGACTCAGCAACTAGCACCACTACA
ACTACGGTGTACAGTGGAAAGTCCCGATCAAAGGATGGAAACGACGACGTTGATATCATCATCGTCGGTGCCGGAGTTGCCGGTGCTGCTCT
TGCTCACACTCTTGGCAAGGTATTGTTTCTTTCTTTGTAATCCGTTTTTTTAAAGTTATATTCTACTTGGTCCTTCAACTATTTTTTATGTACATAT
GTGAGCAAACCGTAATTGTGTAGTGAGAAGTAATTAGATTGATTGATTTACGTATCTGAAGGCATAGTTGATGTGGTTATTCATAAAAAACTT
GAACTTGATTGTTAATTTGATGAAATCAGAGAAATGAAAGATCCATTGATGATGAAAGCGTTTGGTGTGACTGCAAATAATTTTATTGGAGTT
CTTTGTTGACAATTCTCTATAAAAAAAAATTATTTTTTTGTGAGATTTATGATTATACATTTGGGACTACAGGAAGGGCGTCGTGTAAAAGTAAT
TGAAAGAGATTTGACAGAGCCTGATCGAATTGTTGGAGAACTCCTACAACCGGGTGGTTACCTCAAATTGCAGGAGTTGGGATTGGAAGGTA
AAGTTCCTAAGTGATGTGATTTGTGTCAATTCTGGTTAATTTACTCTCTTGAAGAGAAAAAAAAGGAAGTTAGTCTTACGCTTTTAGCTTCTCG
TCTGTCTCTTTTTCGGCATTTCTCATATTTGTATTTTGGTCTACTGCAGATTGTGTGGAGAAAATTGATGCTCAACGAGTGTTTGGGTATGCCCT
TTTCAAGGATGGAAAGAGTACACGTCTTTCTTATCCCCTGG
SQE_spg55_seq1
TTGGGGTTCGTTGCAGTTTTCTGTTTTTTCATTCAGAGAAATGGTGGCTGCAGCAAAAGTAAAATTGATGATTCAGCAACTAGCACCACTACA
ACTACGGTGTACAGTGGAAAGTCCCGATCAAAGGATGGAAACGACGACGTTGATATCATCATCGTCGGTGCCGGAGTTGCCGGTGCTGCTCT
TGCTCACACTCTTGGCAAGGTATTGTTTCTTTCTTTGTAATCCTTTTTTTTAAAGTTATTGTTCTACTTTGGTCCTTTAACTATTTTTATGTACATAT
GTGAGCAAACCGTAATTGTGTAGTGAGAAGTAATTAGACTGATTGATTTACGTATCTGAAGACATAGTTGATGTGGTTATTCATAAAAAACTT
GAACTTGATTGTTAATTTGATGAATCAGAGAAATGAAAGATGCATTGATGATGAAAGCGTTTGGTGTGACTGCAATAGTTTTATTGGAGTTCT
TTGTTGACAATTCTCTATAAAAAAAAATTATTTTTTTGTGAGATTTATGATTATACATTTGGGACTACAGGAAGGGCGTCGTGTAAAAGTAATTG
AAAGAGATTTGACAGAGCCTGATCGAATTGTTGGAGAACTCCTACAACCGGGTGGTTACCTCAAATTGCAGGAGTTGGGATTGGAAGGTAAA
GTTCCTAAATGATGTGATTTGTGTCAATTCTGGTTAATTTACTGTCTTGAAGAGAAAAAAAGGAAAGTCTTACATTTTTAGCTTCTCGTCTGTCT
CTTTTTCGGCATTTCTCATATTTGTATTTTGGTCTACTGCAGATTGTGTGGAGAAAATTGATGCTCAACGAGTGTTTGGGTATGCCCTTTTCAAG
GATGGAAAGAGTACACGTCTTTCTTATCCCCTGG
SQE_spg55_seq1-2
TTGGGGTTCGTTGCAGTTTTCTGTTTTTTCATTCAGAGAAATGGTGGCTGCAGCAAAAGTAAAATTGATGATTCAGCAACTAGCACCACTACA
ACTACGGTGTACAGTGGAAAGTCCCGATCAAAGGATGGAAACGACGACGTTGATATCATCATCGTCGGTGCCGGAGTTGCCGGTGCTGCTCT
TGCTCACACTCTTGGCAAGGTATTGTTTCTTTCTTTGTAATCCTTTTTTTTAAAGTTATTGTTCTACTTTGGTCCTTTAACTATTTTTATGTACATAT
GTGAGCAAACCGTAATTGTGTAGTGAGAAGTAATTAGACTGATTGATTTACGTATCTGAAGACATAGTTGATGTGGTTATTCATAAAAAACTT
GAACTTGATTGTTAATTTGATGAATCAGAGAAATGAAAGATGCATTGATGATGAAAGCGTTTGGTGTGACTGCAATAGTTTTATTGGAGTTCT
TTGTTGACAATTCTCTATAAAAAAAAATTATTTTTTTGTGAGATTTATGATTATACATTTGGGACTACAGGAAGGGCGTCGTGTAAAAGTAATTG
AAAGAGATTTGACAGAGCCTGATCGAATTGTTGGAGAACTCCTACAACCGGGTGGTTACCTCAAATTGCAGGAGTTGGGATTGGAAGGTAAA
GTTCCTAAATGATGTGATTTGTGTCAATTCTGGTTAATTTACTGTCTTGAAGAGAAAAAAAGGAAAGTCTTACATTTTTAGCTTCTCGTCTGTCT
CTTTTTCGGCATTTCTCATATTTGTATTTTGGTCTACTGCAGATTGTGTGGAGAAAATTGATGCTCAACGAGTGTTTGGGTATGCCCTTTTCAAG
GATGGAAAGAGTACACGTCTTTCTTATCCCCTGG
SQE_spg74_seq2
TTGGGGTTCGTTGCAGTTTTCTGTTTTTTCATTCAGAGAAATGGTGGCTGCAGCAAAAGTAAAATTGATGATTCAGCAACTAGCACCACTACA
ACTACGGTGTACAGTGGAAAGTCCCGATCAAAGGATGGAAACGACGACGTTGATATCATCATCGTCGGTGCCGGAGTTGCCGGTGCTGCTCT
TGCTCACACTCTTGGCAAGGTATTGTTTCTTTCTTTGTAATCCTTTTTTTTAAAGTTATTGTTCTACTTTGGTCCTTTAACTATTTTTATGTACATAT

GTGAGCAAACCGTAATTGTGTAGTGAGAAGTAATTAGACTGATTGATTTACGTATCTGAAGACATAGTTGATGTGGTTATTCATAAAAAACTT
GAACTTGATTGTTAATTTGATGAAACCAGAGAAATGAAAGATGCATTGATGATGAAAGCTTTTGGTGTGACTGCAATTTTTTTTATTGGAGTTC
TTTGTTGACAATTCTCTATGAAAAAAAATCTTTTTTGTGAGATTTATGATTATACATTTGGGACTACAGGAAGGGCGTCGTGTAAAAGTAATTG
AAAGAGATTTGACAGAGCCTGATCGAATTGTTGGAGAACTCCTACAACCGGGTGGTTACCTCAAATTGCAGGAGTTGGGATTGGAAGGTAAA
GTTCCTAAGTGATGTGATTTGTGTCAATTCTGGTTAATTTACTCTCTTGAAGAGAAAAAAAAGGAAGTTAGTCTTACATTTTTAGCTTCTCGTCT
GTCTCTTTTTCGGCATTTCTCATATTTGTATTTTGGTCTACTGCAGATTGTGTGGAGAAAATTGATGCTCAACGAGTGTTTGGGTATGCCCTTTT
CAAGGATGGAAAGAGTACACGTCTTTCTTATCCCCTGG
SQE_spg74_seq2-2
TTGGGGTTCGTTGCAGTTTTCTGTTTTTTCATTCAGAGAAATGGTGGCTGCAGCAAAAGTAAAATTGATGATTCAGCAACTAGCACCACTACA
ACTACGGTGTACAGTGGAAAGTCCCGATCAAAGGATGGAAACGACGACGTTGATATCATCATCGTCGGTGCCGGAGTTGCCGGTGCTGCTCT
TGCTCACACTCTTGGCAAGGTATTGTTTCTTTCTTTGTAATCCTTTTTTTTAAAGTTATTGTTCTACTTTGGTCCTTTAACTATTTTTATGTACATAT
GTGAGCAAACCGTAATTGTGTAGTGAGAAGTAATTAGACTGATTGATTTACGTATCTGAAGACATAGTTGATGTGGTTATTCATAAAAAACTT
GAACTTGATTGTTAATTTGATGAAACCAGAGAAATGAAAGATGCATTGATGATGAAAGCTTTTGGTGTGACTGCAATTTTTTTTATTGGAGTTC
TTTGTTGACAATTCTCTATGAAAAAAAATCTTTTTTGTGAGATTTATGATTATACATTTGGGACTACAGGAAGGGCGTCGTGTAAAAGTAATTG
AAAGAGATTTGACAGAGCCTGATCGAATTGTTGGAGAACTCCTACAACCGGGTGGTTACCTCAAATTGCAGGAGTTGGGATTGGAAGGTAAA
GTTCCTAAGTGATGTGATTTGTGTCAATTCTGGTTAATTTACTCTCTTGAAGAGAAAAAAAAGGAAGTTAGTCTTACATTTTTAGCTTCTCGTCT
GTCTCTTTTTCGGCATTTCTCATATTTGTATTTTGGTCTACTGCAGATTGTGTGGAGAAAATTGATGCTCAACGAGTGTTTGGGTATGCCCTTTT
CAAGGATGGAAAGAGTACACGTCTTTCTTATCCCCTGG
SQE_spl16_seq1
TTGGGGTTCGTTGCAGTTTTCTGTTTTTTCATTCAGAGAAATGGTGGCTGCAGCAAAAGTAAAATTGATGATTCAGCAACTAGCACCACTACA
ACTACGGTGTACAGTGGAAAGTCCCGATCAAAGGATGGAAACGACGACGTTGATATCATCATCGTCGGAGCCGGAGTTGCCGGTGCTGCTCT
TGCTCACACTCTTGGCAAGGTATTGTTTCTTTCTTTGTAATCCTTTTTTTTAAAGTTATTGTTCTACCTTGGTCCTTTAACTATTTTTATGTACATAT
GTGAGAAAACCGTAATTGTGTAGTGAGAAGTAATTAGACTGATTGATTTACGTATCTGAAGACATAGTTGATGTGGTTATTCATAAAAAACTT
GAACTTGATTGTTAATTTGATGAAACCAGAGAAATGAAAGATGCATTGATGATGAAAGCGTTTGGTGTGACTGCAATTTTTTTTATTGGAGTT
CTTTGTTGACAATTCTCTATGGAAAAAATTCTTTTTTGTGAGATTTATGATTATACATTTGGGACTACAGGAAGGGCGTCGTGTAAAAGTAATT
GAAAGAGATTTGACAGAGCCTGATCGAATTGTTGGAGAACTCCTACAGCCGGGTGGTTACCTCAAATTGCAGGAGTTGGGATTGGAAGGTA
AAGTTCCTAAGTGATGTGATTTGTGTCAATTCTGGTTAATTTACTCTCTTGAAGAGAAAAAAAAGGAAGTTAGTCTTACATTTTTAGCTTCTCGT
CTGTCTCTTTTTCGGCATTTCTCATATTTGTATTTTGGTCTACTGCAGATTGTGTGGAGAAAATTGATGCTCAACGAGTGTTTGGGTATGCCCTT
TTCAAGGATGGAAAGAGTACACGTCTTTCTTATCCCCTGG
SQE_spl16_seq1-2
TTGGGGTTCGTTGCAGTTTTCTGTTTTTTCATTCAGAGAAATGGTGGCTGCAGCAAAAGTAAAATTGATGATTCAGCAACTAGCACCACTACA
ACTACGGTGTACAGTGGAAAGTCCCGATCAAAGGATGGAAACGACGACGTTGATATCATCATCGTCGGAGCCGGAGTTGCCGGTGCTGCTCT
TGCTCACACTCTTGGCAAGGTATTGTTTCTTTCTTTGTAATCCTTTTTTTTAAAGTTATTGTTCTACCTTGGTCCTTTAACTATTTTTATGTACATAT
GTGAGAAAACCGTAATTGTGTAGTGAGAAGTAATTAGACTGATTGATTTACGTATCTGAAGACATAGTTGATGTGGTTATTCATAAAAAACTT
GAACTTGATTGTTAATTTGATGAAACCAGAGAAATGAAAGATGCATTGATGATGAAAGCGTTTGGTGTGACTGCAATTTTTTTTATTGGAGTT
CTTTGTTGACAATTCTCTATGGAAAAAATTCTTTTTTGTGAGATTTATGATTATACATTTGGGACTACAGGAAGGGCGTCGTGTAAAAGTAATT
GAAAGAGATTTGACAGAGCCTGATCGAATTGTTGGAGAACTCCTACAGCCGGGTGGTTACCTCAAATTGCAGGAGTTGGGATTGGAAGGTA
AAGTTCCTAAGTGATGTGATTTGTGTCAATTCTGGTTAATTTACTCTCTTGAAGAGAAAAAAAAGGAAGTTAGTCTTACATTTTTAGCTTCTCGT
CTGTCTCTTTTTCGGCATTTCTCATATTTGTATTTTGGTCTACTGCAGATTGTGTGGAGAAAATTGATGCTCAACGAGTGTTTGGGTATGCCCTT
TTCAAGGATGGAAAGAGTACACGTCTTTCTTATCCCCTGG
SQE_spl81_seq2
TTGGGGTTCGTTGCAGTTTTCTGTTTTTTCATTCAGAGAAATGGTGGCTGCAGCAAAAGTAAAATTGATGATTCAGCAACTAGCACCACTACA
ACTACGATGTACAGTGGAAAGTCCCGATCAAAGGATGGAAACGACGACGTTGATATCATCATCGTCGGTGCCGGAGTTGCCGGTGCTGCTCT
TGCTCACACTCTTGGCAAGGTTTTATTTCTTTCTTTGTAATCCGTTTTTTTAAAGTTATTATTCTACTTTGGTCCTTCAACTATTTTTTATGTACATA
TGTGAGCAAACCGTAATTGTGTAGTGAGAAGTAATTAGATTGATTGATTTACGTATCTGAAGGCATAGTTGATGTGGTTATTCATAAAAAACT
TGAACTTGATTGTTAATTTGATGAATCAGAGAAATGAAAGATGCATTGATGATGAAAGCGTTTGGTGTGACTGCAATAATTTTATTGGAGTTC
TTTGTTGACAATTCTCTATAAATTTTTTTTATTTTTTTGTGAGATTTATGATTATACATTTGGGACTACAGGAAGGGCGTCGTGTAAAAGTAATT
GAAAGAGATTTGACAGAGCCTGATCGAATTGTTGGAGAACTCCTACAACCGGGTGGTTACCTCAAATTGCAGGAGTTGGGATTGGAAGGTA
AAGTTCCTAAGTGATGTGATTTGTGTCAATTCTGGTTAATTTACTCTCTTGAAGAGAAAAAAAAGGAAGTTAGTCTTACATTTTTTGCTTCTCGT
CTGTCTATTTTTTGGTATTTCTCATATTTGTATTTTGGTCTACTGCAGATTGTGTGGAGAAAATTGATGCTCAACGAGTGTTTGGGTATGCCCTT
TTCAAGGATGGAAAGAGTACACGTCTTTCTTATCCCCTGG
SQE_spl81_seq3
TTGGGGTTCGTTGCAGTTTTCTGTTTTTTCATTCAGAGAAATGGTGGCTGCAGCAAAAGTAAAATTGATGATTCAGCAACTAGCACCACTACA
ACTACGGTGTACAGTGGAAAGTCCGGATCAAAGGATGGAAACGACGACGTTGATATCATCATCGTTGGTGCCGGAGTTGCCGGTGCTGCTCT
TGCTCACACTCTTGGCAAGGTATTGTTTCTTTCTTTGTAATCCTTTTTTTTAAAGTTATTGTTCTACTTTGATCCTTTAACTATTTTTATGTACATAT
GTGAGCAAACCGTAATTGTGAAGTGAGAAGTAATTAGACTGATTGATTTACGTATCTGAAGACATAGTTGATGTGGTTATTCATAAAAGACTT
GAACTTGATTGTTAATTTGATGAAACCAGAAAAATGAAAGATGCATTGATGATGAAAGCGTTTGGTGTGACTGCAAATTTTTTTATTGGAGTT
CTTTGTTGACAATTCTCTATGAAAAAAAATTCTTTTTTATGAGATTTATGATTATACATTTGGGACTACAGGAAGGGCGTCGTGTAAAAGTAAT

TGAAAGAGATTTGACAGAGCCTGATCGAATTGTTGGAGAACTCCTACAACCGGGTGGTTACCTCAAATTGCAGGAGTTGGGATTGGAAGGTA
AAGTTCCTAAGTGATGTGATTTGTGTCAATTCTGGTTAATTTACTCTCTTGAAGAGAAAAAAAAGGAAGTTAGTCTTACACTTTTAGCTTCTCG
TCTGTCTCTTTTTTGGCATTTCTCATATTTGTATTTTGGTCTACTGCAGATTGTGTGGAGAAAATTGATGCTCAACGAGTGTTTGGGTATGCCCT
TTTCAAGGATGGTAAGAGTACACGTCTTTCTTATCCCCTGG
SQE_sto40_seq1
TTGGGGTTCGTTGCAGTTTTCTGTTTTTTCGTTCAGAGAAATGGTGGCCGCAGCAAAAGTAAAATTGATGACTCGGCAACTAGCACCACTACA
ACTACGGTGTACAGTGGAGAGTCTAGATCAAAGGATGGAAACGACGAAATTGATATCATCATCGTCGGTGCCGGTGTTGCCGGTGCTGCTCT
TGCTCACACTCTTGGCAAGGTATTGTTTCTTTCTTTGTAATCCTTTTTTTAAAAGTTATTATTCTACTTTGGTCCTTTAACTATTTTTATGTACATAT
GTGAGCAAACCGTAATTGTGTAGTGAGAAGTAATTAGACTGATTGATTTACGTATCTGAAGGCATACTTGATTGTTAATTTGATGAAATCAGA
GAAATGAAAGATGCATTGATGATGAAACCGTTTAGTGTGACTGCAAATAATTTTATTGGAGTTCTTTGTTGACAATTCTCTATAAAAATATTCT
TTTTTATGAGACTTATGATTATACATTTGGGACTACAGGAAGGGCGTCGTGTAAAAGTAATTGAAAGAGATTTGACAGAGCCTGATCGAATTG
TTGGAGAACTACTACAACCGGGTGGTTACCTCAAATTGCAGGAGTTGGGATTGGAAGGTAAAGTTCCTAAATGATGTGATTTGTGTCAATTCT
GGTTAATTTACTCTCTTGAAGAGAAAAAAAGGAAGTTAGTCTTACATTTTTAGCTTCTCGTCTGTCTCTTTTTCGGAATTTCTCATATTTGTATTT
TGGTCTACTGCAGATTGTGTGGAGAAAATTGATGCTCAACGAGTGTTTGGGTATGCCCTTTTCAAGGATGGAAAGAGTACACGTCTTTCTTAT
CCCCTGG
SQE_sto40_seq2
TTGGGGTTCGTTGCAGTTTTCTGTTTTTTCATTCAGAGAAATGGTGGCTGCAGCAAAAGTAAAATTGATGATTCAGCAACTAGCACCACTACA
ACTACGGTGTACAGTGGAAAGTCCCGATCAAAGGATGGAAACGACGACGTTGATATCATCATCGTTGGTGCCGGAGTTGCCGGTGCTGCTCT
TGCTCACACTCTTGGCAAGGTATTGTTTCTTTCTTTGTAATCCTTTTTTTTAAAGTTATTGTTCTACTTTGATCCTTTAACTATTTTTATGTACATAT
GTGAGCAAACCGTAATTGTGAAGTGAGAAGTAATTAGACTGATTGATTTACGTATCTGAAGACATAGTTGATGTGGTTATTCATAAAAGACTT
GAACTTGATTGTTAATTTGATGAAACCAGAGAAATGAAAGATGCATTGATGATGAAAGCGTTTGGTGTGACTGCAAATTTTTTTATTGGAGTT
CTTTGTTGACAATTCTCTATGAAAAAAAAATTCTTTTTTGTGAGATTTATGATTATACATTTGGGACTACAGGAAGGGCGTCGTGTAAAAGTAAT
TGAAAGAGATTTGACAGAGCCTGATCGAATTGTTGGAGAACTCCTACAACCGGGTGGTTACCTCAAATTGCAGGAGTTGGGATTGGAAGGTA
AAGTTCCTAAGTGATGTGATTTGTGTCAATTCTGGTTAATTTACTCTCTTGAAGAGAAAAAAAAGGAAGTTAGTCTTACACTTTTAGCTTCTCG
TCTGTCTCTTTTTTGGCATTTCTCATATTTGTATTTTGGTCTACTGCAGATTGTGTGGAGAAAATTGATGCTCAACGAGTGTTTGGGTATGCCCT
TTTCAAGGATGGAAAGAGTACACGTCTTTCTTATCCCCTGG
SQE_sto61_seq2
TTGGGGTTCGTTGCAGTTTTCTGTTTTTTCATTCAGAGAAATGGTGGCTGCAGCAAAAGTAAAATTGATGATTCAGCAACTAGCACCACTACA
ACTACGGTGTACAGTGGAAAGTCCCGATCAAAGGATGGAAACGACGACGTTGATATCATCATCGTTGGTGCCGGAGTTGCCGGTGCTGCTCT
TGCTCACACTCTTGGCAAGGTATTGTTTCTTTCTTTGTAATCCTTTTTTTTAAAGTTATTGTTCTACTTTGATCCTTTAACTATTTTTATGTACATAT
GTGAGCAAACCGTAATTGTGAAGTGAGAAGTAATTAGACTGATTGATTTACGTATCTGAAGACATAGTTGATGTGGTTATTCATAAAAGACTT
GAACTTGATTGTTAATTTGATGAAACCAGAGAAATGAAAGATGCATTGATGATGAAAGCGTTTGGTGTGACTGCAAATTTTTTTATTGGAGTT
CTTTGTTGACAATTCTCTATGAAAAAAAAATTCTTTTTTGTGAGATTTATGATTATACATTTGGGACTACAGGAAGGGCGTCGTGTAAAAGTAAT
TGAAAGAGATTTGACAGAGCCTGATCGAATTGTTGGAGAACTCCTACAACCGGGTGGTTACCTCAAATTGCAGGAGTTGGGATTGGAAGGTA
AAGTTCCTAAGTGATGTGATTTGTGTCAATTCTGGTTAATTTACTCTCTTGAAGAGAAAAAAAAGGAAGTTAGTCTTACACTTTTAGCTTCTCG
TCTGTCTCTTTTTTGGCATTTCTCATATTTGTATTTTGGTCTACTGCAGATTGTGTGGAGAAAATTGATGCTCAACGAGTGTTTGGGTATGCCCT
TTTCAAGGATGGAAAGAGTACACGTCTTTCTTATCCCCTGG
SQE_sto61_seq3
TTGGGGTTCGTTGCAGTTTTCTGTTTTTTCGTTCAGAGAAATGGTGGCCGCAGCAAAAGTAAAATTGATGACTCGGCAACTAGCACCACTACA
ACTACGGTGTACAGTGGAGAGTCTAGATCAAAGGATGGAAACGACGAAATTGATATCATCATCGTCGGTGCCGGTGTTGCCGGTGCTGCTCT
TGCTCACACTCTTGGCAAGGTATTGTTTCTTTCTTTGTAATCCTTTTTTTAAAAGTTATTATTCTACTTTGGTCCTTTAACTATTTTTATGTACATAT
GTGAGCAAGCCGTAATTGTGTAGTGAGAAGTAATTAGACTGATTGATTTACGTATCTGAAGGCATACTTGATTGTTAATTTGATGAAATCAGA
GAAATGAAAGATGCATTGATGATGAAACCGTTTAGTGTGACTGCAAATAATTTTATTGGAGTTCTTTGTTGACAATTCTCTATAAAAATATTCT
TTTTTATGAGACTTATGATTATACATTTGGGACTACAGGAAGGGCGTCGTGTAAAAGTAATTGAAAGAGATTTGACAGAGCCTGATCGAATTG
TTGGAGAACTACTACAACCGGGTGGTTACCTCAAATTGCAGGAGTTGGGATTGGAAGGTAAAGTTCCTAAATGATGTGATTTGTGTCAATTCT
GGTTAATTTACTCTCTTGAAGAGAAAAAAAGGAAGTTAGTCTTACATTTTTAGCTTCTCGTCTGTCTCTTTTTCGGAATTTCTCATATTTGTATTT
TGGTCTACTGCAGATTGTGTGGAGAAAATTGATGCTCAACGAGTGTTTGGGTATGCCCTTTTCAAGGATGGAAAGAGTACACGTCTTTCTTAT
CCCCTGG
SQE_chc80-1_seq1
TTGGGGTTCGTTGCAGTTTTCTGTTTTTTCATTCAGAGAAATGGTGGCTGCAGCAAAAGTAAAATTGATGACTCAGCAACTAGCACCACTACA
ACTACGGTGTACAGTGGAAAGTCCCGATCAAAGGATGGAAACGACGACGTTGATATCATCATCGTCGGTGCCGGAGTTGCCGGTGCTGCTCT
TGCTCACACTCTTGGCAAGGTATTGTTTCTTTCTTTGTAATCCTTTTTTTTAAAGTTATTGTTCTACTTTGGTCCTTTAACTATTTTTATGCACATAT
GTGAGCAAACTGTAATTGTGTAGTGAGAAGTAATTAGACTGATTGATTTACGTATCTGAAGACATAGTTGATGTGGTTATTCATAAAAAACTT
GAACTTGATTGTTAATTTGATGAAACCAGAGAAATGAAAGATGCATTGATGATGAAAGCGTTTGGTGTGACTGCAGATTTTTTTATTGGAGTT
CTTTGTTGACAATTCTCTATGAAAAAAATTCTTTTTTGTGAGATTTATGATTATACATTTGGGACTACAGGAAGGGCGTCGTGTAAAAGTAATT
GAAAGAGATTTGACAGAGCCCGATCGAATTGTTGGAGAACTCCTACAACCGGGTGGTTACCTCAAATTGCAGGAGTTGGGATTGGAAGGTA
AAGTTCCTAAGTGATGTGATTTGTGTCAATTCTGGTTAATTTACTCTCTTGAAGAGAAAAAAAAGGAAGTTAGTCTTACATTTTTAGCTTCTCGT

CTGTCTCTTTTTCGGCATTTCTCATGTTTGTATTTTGGTCTACTGCAGATTGTGTGGAGAAAATTGATGCTCAACGAGTGTTTGGGTATGCCCTT
TTCAAGGATGGAAAGAGTACACGTCTTTCTTATCCCCTGG
SQE_chc80-1_seq2
TTGGGGTTCGTTGCAGTTTTCTGTTTTTTCATTCAGAGAAATGGTGGCTGCAGCAAAAGTAAAATTGATGACTCAGCAACTAGCACCACTACA
ACTACGGTGTACAGTGGAAAGTCCCGATCAAAGGATGGAAATGACGACGTTGATATCATCATCGTCGGTGCCGGAGTTGCCGGTGCTGCTCT
TGCTCACACTCTTGGCAAGGTATTGTTTCTTTCTTTGTAATCCTTTTTTTTAAAGTTATTGTTCTACTTTGGTCCTTTAATTATTTTTATGTACATAT
GTGAGCAAACCGTAATTGTGTAGTGAGAAGTAATTAGACTGATTGATTTACGTATCTGAAGACAAAGTTGATGTGGTTATTCGTAAAAAACTT
GAACTTGATTGTTAATTTGATGAAACCAGAGAAATGAAAGATGCATTGATGATGAAAGCGTTTGGTGTGACTGCAAATTTTTTTATTGGAGTT
CTTTGTTGACAATTCTCTATAAAAAAAAATTATTTTTTTGTGAGATTTATGATTATACATTTGGGACTACAGGAAGGGCGTCGTGTAAAAGTAAT
TGAAAGAGATTTGACAGAGCCTGATCGAATTGTTGGAGAACTCCTACAACCGGGTGGTTACCTCAAATTGCAGGAGTTGGGATTGGAAGGTA
AAGTTCCTAAGTGATGTGATTTGTGTCAATTCTGGTTAATTTACTCTCTTGAAGAGAAAAAAAAGGAAGTTAGTCTTACATTTTTAGCTTCTCGT
CTGTCTCTTTTTCGGCATTTCTCATATTTGTATTTTGGTCTACTGCAGATTGTGTGGAGAAAATTGATGCTCAACGAGTGTTTGGGTATGCCCTT
TTCAAGGATGGAAAGAGTACACGTCTTTCTTATCCCCTGG
SQE_phuDH_seq1
TTGGGGTTCGTTGCAGTTTTCTGTTTTTTCATTCAGAGAAATGGTGGCTGCAGCAAAAGTAAAATTGATGACTCAGCAACTAGCACCACTACA
ACTACGGTGTACAGTGGAAAGTCCCGATCAAAGGATGGAAACGACGACGTTGATATCATCATCGTCGGTGCCGGAGTTGCCGGTGCTGCTCT
TGCTCACACTCTTGGCAAGGTATTGTTTCTTTCTTTGTAATCCGTTTTTTAAAAGTTATTATTCTACTTTGGTCCTTCAACTATTTTTATGTACATA
TGTGAGCAAACCGTAATTGTGTAGTGAGAAGTAATTAGATTGATTGATTTACGTATCTGAAGGCATAGTTGATGTGGTTATTCATAAAAAACT
TGAACTTGATTGTTAATTTGATGAAATCAGAGAAATGAAAGATGCATTGATGATGAAAGCGTTTGGTGTGACTGCAATTTTTTTTATTGGAGT
TCTTTGTTGACAATTCTCTATGAAAAAAAATTCTTTTTTGTGAGATTTATGATTATACATTTGGGACTACAGGAAGGGCGTCGTGTACAAGTAAT
TGAAAGAGATTTGACAGAGCCTGATCGAATTGTTGGAGAACTCCTACAACCGGGTGGTTACCTCAAATTGCAGGAGTTGGGATTGGAAGGTA
AAGTTCCTAAGTGATGTGATTTGTGTCAATTCTGGTTAATTTACTCTCTTGAAGAGAAAAAAAAGGAAGTTAGTCTTGCACTTTTAGCTTCTCG
TCTGTCTCTTTTTCGGCATTTCTCATATTTGTATTTTGGTCTACTGCAGATTGTGTGGAGAAAATTGATGCTCAACGAGTGTTTGGGTATGCCCT
TTTCAAGGATGGAAAGAGTACACGTCTTTCTTATCCCCTGG
SQE_phuDH_seq1-2
TTGGGGTTCGTTGCAGTTTTCTGTTTTTTCATTCAGAGAAATGGTGGCTGCAGCAAAAGTAAAATTGATGACTCAGCAACTAGCACCACTACA
ACTACGGTGTACAGTGGAAAGTCCCGATCAAAGGATGGAAACGACGACGTTGATATCATCATCGTCGGTGCCGGAGTTGCCGGTGCTGCTCT
TGCTCACACTCTTGGCAAGGTATTGTTTCTTTCTTTGTAATCCGTTTTTTAAAAGTTATTATTCTACTTTGGTCCTTCAACTATTTTTATGTACATA
TGTGAGCAAACCGTAATTGTGTAGTGAGAAGTAATTAGATTGATTGATTTACGTATCTGAAGGCATAGTTGATGTGGTTATTCATAAAAAACT
TGAACTTGATTGTTAATTTGATGAAATCAGAGAAATGAAAGATGCATTGATGATGAAAGCGTTTGGTGTGACTGCAATTTTTTTTATTGGAGT
TCTTTGTTGACAATTCTCTATGAAAAAAAATTCTTTTTTGTGAGATTTATGATTATACATTTGGGACTACAGGAAGGGCGTCGTGTACAAGTAAT
TGAAAGAGATTTGACAGAGCCTGATCGAATTGTTGGAGAACTCCTACAACCGGGTGGTTACCTCAAATTGCAGGAGTTGGGATTGGAAGGTA
AAGTTCCTAAGTGATGTGATTTGTGTCAATTCTGGTTAATTTACTCTCTTGAAGAGAAAAAAAAGGAAGTTAGTCTTGCACTTTTAGCTTCTCG
TCTGTCTCTTTTTCGGCATTTCTCATATTTGTATTTTGGTCTACTGCAGATTGTGTGGAGAAAATTGATGCTCAACGAGTGTTTGGGTATGCCCT
TTTCAAGGATGGAAAGAGTACACGTCTTTCTTATCCCCTGG
SQE_phuDM_seq1
TTGGGGTTCGTTGCAGTTTTCTGTTTTTTCATTCAGAGAAATGGTGGCTGCAGCAAAAGTAAAATTGATGACTCAGCAACTAGCACCACTACA
ACTACGGTGTACAGTGGAAAGTCCCGATCAAAGGATGGAAACGACGACGTTGATATCATCATCGTCGGTGCCGGAGTTGCCGGTGCTGCTCT
TGCTCACACTCTTGGCAAGGTATTGTTTCTTTCTTTGTAATCCGTTTTTTAAAAGTTATTATTCTACTTTGGTCCTTCAACTATTTTTATGTACATA
TGTGAGCAAACCGTAATTGTGTAGTGAGAAGTAATTAGATTGATTGATTTACGTATCTGAAGGCATAGTTGATGTGGTTATTCATAAAAAACT
TGAACTTGATTGTTAATTTGATGAAATCAGAGAAATGAAAGATGCATTGATGATGAAAGCGTTTGGTGTGACTGCAATTTTTTTTATTGGAGT
TCTTTGTTGACAATTCTCTATGAAAAAAAATTCTTTTTTGTGAGATTTATGATTATACATTTGGGACTACAGGAAGGGCGTCGTGTACAAGTAAT
TGAAAGAGATTTGACAGAGCCTGATCGAATTGTTGGAGAACTCCTACAACCGGGTGGTTACCTCAAATTGCAGGAGTTGGGATTGGAAGGTA
AAGTTCCTAAGTGATGTGATTTGTGTCAATTCTGGTTAATTTACTCTCTTGAAGAGAAAAAAAAGGAAGTTAGTCTTGCACTTTTAGCTTCTCG
TCTGTCTCTTTTTCGGCATTTCTCATATTTGTATTTTGGTCTACTGCAGATTGTGTGGAGAAAATTGATGCTCAACGAGTGTTTGGGTATGCCCT
TTTCAAGGATGGAAAGAGTACACGTCTTTCTTATCCCCTGG
SQE_phuDM_seq1-2
TTGGGGTTCGTTGCAGTTTTCTGTTTTTTCATTCAGAGAAATGGTGGCTGCAGCAAAAGTAAAATTGATGACTCAGCAACTAGCACCACTACA
ACTACGGTGTACAGTGGAAAGTCCCGATCAAAGGATGGAAACGACGACGTTGATATCATCATCGTCGGTGCCGGAGTTGCCGGTGCTGCTCT
TGCTCACACTCTTGGCAAGGTATTGTTTCTTTCTTTGTAATCCGTTTTTTAAAAGTTATTATTCTACTTTGGTCCTTCAACTATTTTTATGTACATA
TGTGAGCAAACCGTAATTGTGTAGTGAGAAGTAATTAGATTGATTGATTTACGTATCTGAAGGCATAGTTGATGTGGTTATTCATAAAAAACT
TGAACTTGATTGTTAATTTGATGAAATCAGAGAAATGAAAGATGCATTGATGATGAAAGCGTTTGGTGTGACTGCAATTTTTTTTATTGGAGT
TCTTTGTTGACAATTCTCTATGAAAAAAAATTCTTTTTTGTGAGATTTATGATTATACATTTGGGACTACAGGAAGGGCGTCGTGTACAAGTAAT
TGAAAGAGATTTGACAGAGCCTGATCGAATTGTTGGAGAACTCCTACAACCGGGTGGTTACCTCAAATTGCAGGAGTTGGGATTGGAAGGTA
AAGTTCCTAAGTGATGTGATTTGTGTCAATTCTGGTTAATTTACTCTCTTGAAGAGAAAAAAAAGGAAGTTAGTCTTGCACTTTTAGCTTCTCG
TCTGTCTCTTTTTCGGCATTTCTCATATTTGTATTTTGGTCTACTGCAGATTGTGTGGAGAAAATTGATGCTCAACGAGTGTTTGGGTATGCCCT
TTTCAAGGATGGAAAGAGTACACGTCTTTCTTATCCCCTGG

1.4 Solanidine galactosyltransferase (*SGT1*) sequences
28
SGT1_cmm7_seq1
CCTTGGACAGTAGATATTGCTGATGAGCTTCACATCCCTCGTATTTTGTACAATTTGTCTGCTTACATGTGCTACAGCATTATGCACAACCTTAA
GGTTTACAGACCTCACAAGCAGCCTAATCTAGACGAATCTCAAAGTTTCGTGGTTCCTGGTTTACCTGATGAGATAAAGTTCAAGTTATCCCAA
CTGACAGATGATCTGAGAAGGCCGGATGACCAAAAGACTGTTTTTGACGAATTGCTCGAACAAGTTGCAGATTCGGAGGAACGAAGCTATG
GCATTGTTCATGATACATTTTATGAGCTAGAACCTGCATATGTCGACTACTACCAGAAATTAAAGAAACCAAAATGCTGGCATTTTGGTCCGCT
CTCTCATTTTGCATCCAAAATCCGTAGTAAGGAACTAATTTCTGAGCATAACAACAATGAGATCGTTGTAGATTGGTTGAATGCACAGAAACCT
AAATCGGTTCTCTATGTATCTTTCGGAAGCATGGCTAGATTTCCTGAGAGCCAACTGAATGAAATAGCCGAAGCTCTGGATGCTTCAAATGTT
CCTTTCATTTTTGTATTGAGGCCTAATGAAGAAACGGCGTCGTGGTTGCCAGTTGGTAATTTAGAGGACAAGACTAAAAAGGGTTTGTACATC
AAAGGGTGGGTTCCACAGCTTACGATCATGGAACATTCAGCAACAGGCGGGTTCATGACACATTGTGGTACTAATTCGGTTCTGGAAGCCAT
CACTTTTGGCGTGCCAATGATAACATGGCCACTTTATGCTGATCAATTCTACAACGAGAAGGTAGTCGAGGTTAGGGGATTGGGA
SGT1_cmm7_seq1-2
CCTTGGACAGTAGATATTGCTGATGAGCTTCACATCCCTCGTATTTTGTACAATTTGTCTGCTTACATGTGCTACAGCATTATGCACAACCTTAA
GGTTTACAGACCTCACAAGCAGCCTAATCTAGACGAATCTCAAAGTTTCGTGGTTCCTGGTTTACCTGATGAGATAAAGTTCAAGTTATCCCAA
CTGACAGATGATCTGAGAAGGCCGGATGACCAAAAGACTGTTTTTGACGAATTGCTCGAACAAGTTGCAGATTCGGAGGAACGAAGCTATG
GCATTGTTCATGATACATTTTATGAGCTAGAACCTGCATATGTCGACTACTACCAGAAATTAAAGAAACCAAAATGCTGGCATTTTGGTCCGCT
CTCTCATTTTGCATCCAAAATCCGTAGTAAGGAACTAATTTCTGAGCATAACAACAATGAGATCGTTGTAGATTGGTTGAATGCACAGAAACCT
AAATCGGTTCTCTATGTATCTTTCGGAAGCATGGCTAGATTTCCTGAGAGCCAACTGAATGAAATAGCCGAAGCTCTGGATGCTTCAAATGTT
CCTTTCATTTTTGTATTGAGGCCTAATGAAGAAACGGCGTCGTGGTTGCCAGTTGGTAATTTAGAGGACAAGACTAAAAAGGGTTTGTACATC
AAAGGGTGGGTTCCACAGCTTACGATCATGGAACATTCAGCAACAGGCGGGTTCATGACACATTGTGGTACTAATTCGGTTCTGGAAGCCAT
CACTTTTGGCGTGCCAATGATAACATGGCCACTTTATGCTGATCAATTCTACAACGAGAAGGTAGTCGAGGTTAGGGGATTGGGA
SGT1_cmm26_seq1
CCTTGGACAGTAGATATTGCTGATGAGCTTCACATCCCTCGTATTTTGTACAATTTGTCTGCTTACATGTGCTACAGCATTATGCACAACCTTAA
GGTTTACAGACCTCACAAGCAGCCTAATCTAGACGAATCTCAAAGTTTCGTGGTTCCTGGTTTACCTGATGAGATAAAGTTCAAGTTATCCCAA
CTGACAGATGATCTGAGAAGGCCGGATGACCAAAAGACTGTTTTTGACGAATTGCTCGAACAAGTTGCAGATTCGGAGGAACGAAGCTATG
GCATTGTTCATGATACATTTTATGAGCTAGAACCTGCATATGTCGACTACTACCAGAAATTAAAGAAACCAAAATGCTGGCATTTTGGTCCGCT
CTCTCATTTTGCATCCAAAATCCGTAGTAAGGAACTAATTTCTGAGCATAACAACAATGAGATCGTTGTAGATTGGTTGAATGCACAGAAACCT
AAATCGGTTCTCTATGTATCTTTCGGAAGCATGGCTAGATTTCCTGAGAGCCAACTGAATGAAATAGCCGAAGCTCTGGATGCTTCAAATGTT
CCTTTCATTTTTGTATTGAGGCCTAATGAAGAAACGGCGTCGTGGTTGCCAGTTGGTAATTTAGAGGACAAGACTAAAAAGGGTTTGTACATC
AAAGGGTGGGTTCCACAGCTTACGATCATGGAACATTCAGCAACAGGCGGGTTCATGACACATTGTGGTACTAATTCGGTTCTGGAAGCCAT
CACTTTTGGCGTGCCAATGATAACATGGCCACTTTATGCTGATCAATTCTACAACGAGAAGGTAGTCGAGGTTAGGGGATTGGGA
SGT1_cmm26_seq1-2
CCTTGGACAGTAGATATTGCTGATGAGCTTCACATCCCTCGTATTTTGTACAATTTGTCTGCTTACATGTGCTACAGCATTATGCACAACCTTAA
GGTTTACAGACCTCACAAGCAGCCTAATCTAGACGAATCTCAAAGTTTCGTGGTTCCTGGTTTACCTGATGAGATAAAGTTCAAGTTATCCCAA
CTGACAGATGATCTGAGAAGGCCGGATGACCAAAAGACTGTTTTTGACGAATTGCTCGAACAAGTTGCAGATTCGGAGGAACGAAGCTATG
GCATTGTTCATGATACATTTTATGAGCTAGAACCTGCATATGTCGACTACTACCAGAAATTAAAGAAACCAAAATGCTGGCATTTTGGTCCGCT
CTCTCATTTTGCATCCAAAATCCGTAGTAAGGAACTAATTTCTGAGCATAACAACAATGAGATCGTTGTAGATTGGTTGAATGCACAGAAACCT
AAATCGGTTCTCTATGTATCTTTCGGAAGCATGGCTAGATTTCCTGAGAGCCAACTGAATGAAATAGCCGAAGCTCTGGATGCTTCAAATGTT
CCTTTCATTTTTGTATTGAGGCCTAATGAAGAAACGGCGTCGTGGTTGCCAGTTGGTAATTTAGAGGACAAGACTAAAAAGGGTTTGTACATC
AAAGGGTGGGTTCCACAGCTTACGATCATGGAACATTCAGCAACAGGCGGGTTCATGACACATTGTGGTACTAATTCGGTTCTGGAAGCCAT
CACTTTTGGCGTGCCAATGATAACATGGCCACTTTATGCTGATCAATTCTACAACGAGAAGGTAGTCGAGGTTAGGGGATTGGGA
SGT1_dms54_seq1
CCTTGGACAGTAGATATTGCTGATGAGCTGCACATCCCTCGTATTTTGTACAATTTGTCTGCTTACATGTGCTACAGCATTATGCACAACCTTAA
GGTTTACAGACCTCACAAGCAGCCTAATCTAGACGAATCTCAAAGTTTCGTGGTTCCTGGTTTACCTGATGAGATAAAGTTCAAGTTATCCCAA
CTGACAGATGATCTGAGAAAGCCGGATGACCAAAAGACTGGTTTTGACGAATTGCTCGAACAAGTTGGAGATTCGGAGGAGCGAAGCTATG
GCATTGTTCATGATACATTTTATGAGCTAGAACCTGCATATGTCGACTACTACCAGAAATTAAAGAAACCAAAATGTTGGCATTTTGGTCCGCT
CTCTCATTTTGCATCCAAAATCCGTAGTAAGGAACTAATTTCTGAGCATAACAACAATGAGATTGTTATAGATTGGTTGAATGCACAGAAACCT
AAATCGGTTCTCTATGTATCTTTCGGAAGCATGGCTAGATTTCCTGAGAGCCAACTGAATGAAATAGCCCAAGCTCTGGATGCTTCAAATGTTC
CTTTCATTTTTGTATTGAGGCCTAATGAAAAAACAACGTCGTGGTTGCCAGTTGGTAATTTAGAGGACAAGACTAAAAAGGGTTTGTACATCA
AAGGGTGGGTCCCACAGCTTACGATCATGGAACATTCAGCAACAGGCGGGTTCATGACTCATTGTGGTACTAATTCGGTTCTGGAAGCCATC
ACTTTTGGCGTGCCAATGATAACATGGCCACTTTATGCTGATCAATTCTACAACGAGAAGGTAGTCGAGGTTAGGGGATTGGGA
SGT1_dms54_seq2
CCTTGGACAGTAGATATTGCTGATGAGCTTCACATCCCTCGTATTTTGTACAATTTGTCTTCTTACATGTGCTACAGCATTAAGCACAACCTTAA
GGTTTACAGACCTCACAAGCAGCCTAATCTAGACGAATCTCAAAGTTTCGTGGTTCCTGGTTTACCTAATGAGATAAAGTTCAAGTTATCCCAA
CTGACAGATGATCTGAGAAAGCCGGATGACCAAAAGACTGTTTTTGACGAATTGCTCGAACAAGTTGGAGATTCGGAGGAACGAAGCTATG
GCATTGTTCATGATACATTTTATGAGCTAGAACCTGCATATGTCGACTATTACCAGAAATTAAAGAAACCAAAATGTTGGCATTTTGGTCCGCA
CTCTCATTTTGCATCCAAAATCCGTAGTAAGGAACTAATTTCTGAGCATAACAACAATGAGATTGTTATAGATTGGTTGAATGCACAGAAACCT

AAATCGGTTATCTATGTATCTTTCGGAAGCATGGCTAGATTTCCTGAGAGCCAACTGAATGAAATAGCCCAAGCTCTGGATGCTTCAAATGTT
CCTTTCATTTGTGTATTGAGGCCTAATGAAGAAACAATGTCGTGGTTGCCAGTTGGTAATTTAGAGGACAAGACTAAAAAGGGTTTGTACATC
AAAGGGTGGGTCCCACAACTTACGATCATGGAACATTCAGCAACAGGCGGGTTCATGACTCATTGTGGTACTAATTCGGTTCTAGAAGCCATC
ACTTTTGGCGTGCCAATGATAACATGGCCACTTTATGCTGATCAATTCTACAACGAGAAGGTAGTCGAGGTTAGGGGATTGGGA
SGT1_dms78_seq3
CCTTGGACAGTAGATATTGCTGATGAGCTTCACATCCCTCGTATTTTGTACAATTTGTCTGCTTACATGTGCTACAGCATTATGCACAACCTTAA
GGTTTACAGACCTCACAAGCAGCCTAATCTAGACGAATCTCAAAGTTTCGTGGTTCCTGGTTTACCTGATGAGATAAAGTTCAAGTTATCCCAA
CTGACAGATGATCTGAGAAAGCCGGATGACCAAAAGACTGGTTTTGACGAATTGCTCGAACAAGTTGGAGATTCGGAGGAGCGAAGCTATG
GCATTGTTCATGATACATTTTATGAGCTAGAACCTGCATATGTCGACTACTACCAGAAATTAAAGAAACCAAAATGTTGGCATTTTGGTCCGCT
CTCTCATTTTTCATCCAAAATTCGTAGTAAGGAACTAATTTCTGAGCATAACAACAATGAGATTGTTATAGATTGGTTGAATGCACAGAAACCT
AAATCGATTTTCTATGTATCTTTCGGAAGCATTGCTAGATATCCTGAGAGCCAACTGAATGAAATAGCCCAAGCTCTGGATGCTTCAAATGTTC
CTTTCATTTTTGTATTGAGGCCTAATGAAGAAACAACGTCGTGGTTGCCAGTTGGTAATTTAGAGGACAAGACTAAAAAGGGTTTGTACATCA
AAGGGTGGGTCCCACAACTTACGATCATGGAACATTCAGCAACAGGCGGGTTCATGACTCATTGTGGTACTAATTCGGTTCTGGAAGCCATC
ACTTTTGGCGTGCCAATGATAACATGGCCACTTTATGCTGATCAATTCTACAACGAGAAGGTAGTCGAGGTTAGGGGATTGGGA
SGT1_dms78_seq4
CCTTGGACAGTAGATATTGCTGATGAGCTTCACATCCCTCGTATTTTGTACAATTTGTCTGCTTACATGTGCTACAGCATTATGCACAACCTTAA
GGTTTACAGACCTCACAAGCAGCCTAATCTAGACGAATCTCAAAGTTTCGTGGTTCCTGGTTTACCTAATGAGATAAAGTTCAAGTTATCCCAA
CTGACAGATGATCTGAGAAAGCCGGATGACCAAAAGACTGTTTTTGACGAATTGCTCGAACAAGTTGGAGATTCGGAGGAACGAAGCTATG
GCATTGTTCATGATACATTTTATGAGCTAGAACCTGCATATGTCGACTATTACCAGAAATTAAAGAAACCAAAATGTTGGCATTTTGGTCCGCT
CTCTCATTTTGCATCCAAAATTCGTAGTAAGGAACTAATTTCTGAGCATAACAACAATGAGATTGTTATAGATTGGTTGAATGCACAGAAACCT
AAATCGGTTCTCTATGTATCTTTCGGAAGCATGGCTAGATTTCCTGAGAGCCAACTGAATGAAATAGCCCAAGCTCTGAATGCTTCAAATGTTC
CTTTCATTTTTGTATTGAGGCCTAATGAAGAAACAACGTCGTGGTTGCCAGTTGGTAATTTAGAGGACAAGACTAAAAAGGGTTTGTACATCA
AAGGGTGGGTCCCACAACTTACGATCATGGAACATTCCGCAACAGGCGGATTCATGACTCATTGTGGTACTAATTCGGTTCTGGAAGCCATCA
CTTTTGGCGTGCCAATGATAACATGGCCACTTTATGCTGATCAATTCTACAACGAGAAGGTAGTCGAGGTTAGGGGATTGGGA
SGT1_slp16_seq1
CCTTGGACAGTAGATATTGCTGATGAGCTTCACATCCCTCGTATTTTGTACAATTTGTCTGCTTACATGTGCTACAGCATTATGCACAACCTTAA
GGTTTACAGACCTCACAAGCAGCCTAATCTAGACGAATCTCAAAGTTTCGTGGTTCCTGGTTTACCTGATGAGATAAAGTTCAAGTTATCCCAA
CTGACAGATGATCTGAGAAAGCCGGATGACCAAAAGACTGTTTTTGACGAATTGCTCGAACAAGTTGGAGATTCGGAGGAACGAAGCTATG
GCATTGTTCATGATACATTTTATGAGCTAGAACCTGCATATGTTGACTACTACCAGAAATTAAAGAAACCAAAATGTTGGCATTTTGGTCCGCT
CTCTCATTTTGCATCCAAAATCCGTAGTAAGGAACTAATTTCTGAGCATAACAACAATGAGATTGTTATAGATTGGTTGAATGCACAGAAACCT
AAATCGGTTCTCTATGTATCTTTCGGAAGCATGGCTAGATTTCCTGAGAGCCAACTGAATGAAATAGCCCAAGCTCTAGATGCTTCAAATGTTC
CTTTCATTTTTGTATTGAGGCCTAATGAAGAAACGGCGTCGTGGTTACCAGTTGGTAATTTAGAGGACAAGACTAAAAAGGGTTTGTACATCA
AAGGGTGGGTCCCACAGCTTACGATCATGGAACATTCAGCAACAGGCGGGTTCATGACTCATTGTGGTACTAATTCGGTTCTGGAAGCCATC
ACTTTTGGCGTGCCAATGATAACATGGCCACTTTATGCTGATCAATTCTACAACGAGAAGGTAGTCGAGGTTAGGGGATTGGGA
SGT1_slp16_seq2
CCTTGGACAGTAGATATTGCTGATGAGCTTCACATCCCTCGTATTTTGTACAATTTGTCTGCTTACATGTGCTACAGCATTATGCACAACCTTAA
GGTTTACAGACCTCACAAGCAGCCTAATCTAGACGAATCTCAAAGTTTCGTGGTTCCTGGTTTACCTGATGAGATAAAGTTCAAGTTATCCCAA
CTGACAGATGATCTGAGAAAGCCGGATGACCAAAAGACTGTTTTTGACGAATTGCTCGAACAAGTTGGAGATTCGGAGGAACGAAGCTATG
GCATTGTTCATGATACATTTTATGAGCTAGAACCTGCCTATGTCGACTACTACCAGAAATTAAAGAAACCAAAATGTTGGCATTTTGGTCCGCT
CTCTCATTTTGCATCCAAAATCCGTAGTAAGGAACTAATTTCTGAGCATAACAACAATGAGATTGTTATAGATTGGTTGAATGCACAGAAACCT
AAATCGGTTCTCTATGTATCTTTCGGAAGCATGGCTAGATTTCCTGAGAGCCAACTGAATGAAATAGCCCAAGCTCTGGATGCTTCAAATGTTC
CTTTCATTTTTGTATTGAGGCCTAATGAAGAAACAGCGTCGTGGTTGCCAGTTGGTAATTTAGAGGACAAGTCTAAAAAGGGTTTGTACATCA
AAGGGTGGGTCCCACAGCTTACGATCATGGAACATTCAGCAACAGGCGGGTTCATGACTCATTGTGGTACTAATTCGGTTTTGGAAGCCATC
ACTTTTGGCGTGCCAATGATAACATGGCCACTTTATGCTGATCAATTCTACAACGAGAAGGTAGTCGAGGTTAGGGGATTGGGA
SGT1_slp81_seq2
CCTTGGACAGTAGATATTGCTGATGAGCTTCACATCCCTCGTATTTTGTACAATTTGTCTGCTTACATGTGCTACAGCATTATGCACAACCTTAA
GGTTTACAGACCTCACAAGCAGCCTAATCTAGACGAATCTCAAAGTTTCGTGGTTCCTGGTTTACCTGATGAGATAAAGTTCAAGTTATCCCAA
CTGACAGATGATCTGAGAAAGCCGGATGACCAAAAGACTGTTTTTGACGAATTGCTCGAACAAGTTGGAGATTCGGAGGAACGAAGCTATG
GCATTGTTCATGATACATTTTATGAGCTAGAACCTGCCTATGTCGACTACTACCAGAAATTAAAGAAACCAAAATGTTGGCATTTTGGTCCGCT
CTCTCATTTTGCATCCAAAATCCGTAGTAAGGAACTAATTTCTGAGCATAACAACAATGAGATTGTTATAGATTGGTTGAATGCACAGAAACCT
AAATCGGTTCTCTATGTATCTTTCGGAAGCATGGCTAGATTTCCTGAGAGCCAACTGAATGAAATAGCCCAAGCTCTGGATGCTTCAAATGTTC
CTTTCATTTTTGTATTGAGGCCTAATGAAGAAACAGCGTCGTGGTTGCCAGTTGGTAATTTAGAGGACAAGTCTAAAAAGGGTTTGTACATCA
AAGGGTGGGTCCCACAGCTTACGATCATGGAACATTCAGCAACAGGCGGGTTCATGACTCATTGTGGTACTAATTCGGTTTTGGAAGCCATC
ACTTTTGGCGTGCCAATGATAACATGGCCACTTTATGCTGATCAATTCTACAACGAGAAGGTAGTCGAGGTTAGGGGATTGGGA
SGT1_slp81_seq3
CCTTGGACAGTAGATATTGCTGATGAGCTTCACATCCCTCGTATTTTGTACAATTTGTCTGCTTACATGTGCTACAGCATTATGCACAACCTTAA
GGTTTACAGACCTCACAAGCAGCCTAATCTAGACGAATCTCAAAGTTTCGTGGTTCCTGGTTTACCTGATGAGATAAAGTTCAAGTTATCCCAA
CTGACAGATGATCTGAGAAAGCCGGATGACCAAAAGACTGTTTTTGACGAATTGCTCGAACAAATTGGAGATTCGGAGGAACGAAGCTATG

GCATTGTTCATGATACATTTTATGAGCTAGAACCTGCATATGTCGACTACTACCAGAAATTAAAGAAACCAAAATGTTGGCATTTTGGTCCGCT
CTCTCATTTTGCATCCAAAATCCGTAGTAAGGAACTAATTTCTGAGCATAACAACAATGAGATTGTTATAGATTGGTTGAATGCACAGAAACCT
AAATCGGTTCTCTATGTATCTTTCGGAAGCATGGCTAGATTTCCTGAGAACCAACTGAATGAAATAGCCCAAGCTCTGGATGCTTCAAATGTTC
CTTTCATTTTTGTATTGAGGCCTAATGAAGAAACAGCGTCGTGGTTGCCAGTTGGTAATTTAGAGGACAAGTCTAAAAAGGGTTTGTACATCA
AAGGGTGGGTCCCACAGCTTACGATCATGGAACATTCAGCAACAGGCGGGTTCATGACTCATTGTGGTACTAATTCGGTTCTGGAAGCCATC
ACTTTTGGCGTGCCAATGATAACATGGCCACTTTATGCTGATCAATTCTACAACGAGAAGGTAGTCGAGGTTAGGGGATTGGGA
SGT1_spg55_seq1
CCTTGGACAGTAGATATTGCTGATGAGCTTCACATCCCTCGTATTTTGTACAATTTGTCTGCTTACATGTGCTACAGTATTATGCACAACCTTAA
GCTTTACAGACCTCACAAGCAGCCCAATCTAGACGAATCTCAAAGTTTCGTGGTTCCTGGTTTACCTGATGAGATAAAGTTCAAGTTATCCCAA
CTGACAGAAGATTTGAGAAAGCCGGATGACCAAAAGACTGTTTTTGACGAATTGCTCGAACAAATTCGAGATGCGGAGGAACGAAGCTATG
GCATTGTTCATGATACATTTTATGAGCTAGAACCTGCATATGTCGACTACTACCAGAAATTAAAGAAACCAAAATGTTGGCATTTTGGTCCGCT
CTCTCATTTTGCATCCAAAATCCGTAGTAAGGAACTAATTTCTGAGCATAACAACAATGAGATTGTTGTAGATTGGTTGAATGCACAGAAACCT
AAATCGGTTCTCTATGTATCTTTCGGAAGCATGGCTAGATTTCCTGAGAGCCAACTGAATGAAATATCCCAAGCTCTGGATGCTTCAAATGTTC
CTTTCATTTTTGTATTGAGGCCTAATGAAGAAACGGCGTCGTGGTTGCCAGTTGGTAATTTAGAGGACAAGACTAAAAAGGGTTTGTACATCA
AAGGGTGGGTCCCACAGCTTACGATCATGGAACATGCAGCAACAGGCGGGTTCATGACTCATTGTGGTACTAATTCGGTTCTGGAAGCCATC
ACTTTTGGCGTGCCAATGATAACATGGCCACTTTATGCTGATCAATTCTACAACGAGAAGGTAGTCGAGGTTAGGGGATTGGGA
SGT1_spg55_seq2
CCTTGGACAGTAGATATTGCTGATGAGCTTCACATCCCTCGTATTTTGTACAATTTGTCTGCTTACATGTGCTACAGTATTATGCACAACCTTAA
GCTTTACAGACCTCACAAGCAGCCCAATCTAGACGAATCTCAAAGTTTCGTGGTTCCTGGTTTACCTGATGAGATAAAGTTCAAGTTATCCCAA
CTGACAGAAGATTTGAGAAAGCCGGATGACCAAAAGACTGTTTTTGACGAATTGCTCGAACAAATTCGAGATGCGGAGGAACGAAGCTATG
GCATTGTTCATGATACATTTTATGAGCTAGAACCTGCATATGTCGACTACTACCAGAAATTAAAGAAACCAAAATGTTGGCATTTTGGTCCGCT
CTCTCATTTTGCATCCAAAATCCGTAGTAAGGAACTAATTTCTGAGCATAACAACAATGAGATTGTTGTAGATTGGTTGAATGCACAGAAACCT
AAATCGGTTCTCTATGTATCTTTCGGAAGCATGGCTAGATTTCCTGAGAGCCAACTGAATGAAATATCCCAAGCTCTGGAGACTTCAAATGTTC
CTTTCATTTTTGTATTGAGGCCTAATGAAGAAACGGCGTCGTGGTTGCCAGTTGGTAATTTAGAGGACACGACTAAAAAGGGTTTGTACATCA
AAGGGTGGGTCCCACAGCTTACGATCATGGAACATGCAGCAACAGGCGGGTTCATGACTCATTGTGGTACTAATTCGGTTCTGGAAGCCATC
ACTTTTGGCGTGCCAATGATAACATGGCCACTTTATGCTGATCAATTCTACAACGAGAAGGTAGTCGAGGTTAGGGGATTGGGA
SGT1_spg74_seq3
CCTTGGACAGTAGATATTGCTGATGAGCTTCACATCCCTCGTATTTTGTACAATTTGTCTGCTTACATGTGCTACAGCATTATGCACAACCTTAA
GGTTTACAGACCTCACAAGCAGCCTAATCTAGACGAATCTCAAAATTTCGTGGTTCCTGGTTTACCTGATGAGATAAAGTTTAAGTTATCCCAA
CTGACAGATGATCTGAGAAAGCCGGATGATCAAAAGACTGTTTTTGACGAATTGCTCGAACAAATTGGAGATTCGGAGGAACGAAGCTATG
GCATTGTTCATGATACATTTTATGAGCTAGAACCTGCATATGTCGACTACTACCAAAAATTAAAGAAACCAAAATGTTGGCATTTTGGTCCGCT
CTCTCATTTTGCATCCAAAATCCGTAGTAAGGAACTAATTTCTGAGCATAACAACAATGAGATTGTTATAGATTGGTTGAATGCACAGAAACCT
AAATCGGTTCTCTATGTATCTTTCGGAAGCATGGCTAGATTTCCTGAGAGCCAACTGAATGAAATAGCCCAAGCTCTGGATGCTTCAAATGTTC
CTTTCATTTTTGTATTGAGGCCTAATGAAGAAACAACGTCGTGGTTGCCAGTTGGTAATTTAGAGGACAAGACTAAAAAGGGTTTGTACATCA
AAGGGTGGGTCCCACAGCTTACGATCATGGAACATTCAGCAACAGGCGGGTTCATGACTCATTGTGGTACTAATTCGGTTCTGGAAGCCATC
ACTTTTGGCGTGCCAATGATAACATGGCCACTTTATGCTGATCAATTCTACAACGAGAAGGTAGTCGAGGTTAGGGGATTGGGA
SGT1_spg74_seq4
CCTTGGACAGTAGATATTGCTGATGAGCTTCACATCCCTCGTATTTTGTACAATTTGTCTGCTTACATATGCTACAACATTATGCACAACCTTAA
GGTTTACAGACCTCACAAGCAGCCAAATCTAGACGAATCTCAAAGTTTCGTGGTTCCTGGTTTACCTGATGAGATAAAGTTCAAGTTATCCCA
ACTGACAGATGATCTGAGAAAGCCGGATGACCAAAAGACTGTTTTTGACGAATTGCTAGAACAAGTTGGAGATTCGGAGGAACGAAGCTAT
GGCATTGTTCATGATACATTTTATGAGCTAGAACCTGCATATGTCGACTACTACCAGAAATTAAAGAAACCAAAATGTTGGCATTTTGGTCCGC
TCTCTCATTTTGCATCCAAAATCCGTAGTAAGGAACTAATTTCTGAGCATAACAACAATGAGATTGTTATAGATTGGTTGAATGCACAAAAACC
TAAATCGGTTCTCTATGTATCTTTCGGAAGCATGGCTAGATTTCCTGAGAGCCAACTGAATGAAATAGACCAAGCTCTGGATGCTTCAAATGTT
CCTTTCATTTTTGTATTGAGGCCTAATGAAGAAACAACGTCGTGGTTGCCAGTTGGTAATTTAGAGGACAAGACTAAAAAGGGTTTGTACATC
AAAGGGTGGGTCCCACAGCTTACGATCATGGAACATTCAGCAACAGGCGGGTTCATGACTCATTGTGGTACTAATTCGGTTCTGGAAGCCAT
CACTTTTGGCGTGCCAATGATAACATGGCCACTTTATGCTGATCAATTCTACAACGAGAAGGTAGTCGAGGTTAGGGGATTGGGA
SGT1_sto40_seq1
CCTTGGACAGTAGATATTGCTGATGAGCTGCACATCCCTCGTATTTTGTACAATTTGTCTGCTTACATGTGCTACAGCATTATGCACAACCTTAA
GGTTTACAGACCTCACAAGCAGCCTAATCTAGACGAATCTCAAAGTTTCGTGGTTCCTGGTTTACCTGATGAGATAAAGTTCAAGTTATCCCAA
CTGACAGATGATCTGAGAAAGCCGGATGACCAAAAGACTGGTTTTGACGAATTGCTCGAACAAGTTGGAGATTCGGAGGAGCGAAGCTATG
GCATTGTTCATGATACATTTTATGAGCTAGAACCTGCATATGTCGACTACTACCAGAAATTAAAGAAACCAAAATGTTGGCATTTTGGTCCGCT
CTCTCATTTTGCATCCAAAATCCGTAGTAAGGAACTAATTTCTGAGCATAACAACAATGAGATTGTTATAGATTGGTTGAATGCACAGAAACCT
AAATCGGTTCTCTATGTATCTTTCGGAAGCATGGCTAGATTTCCTGAGAGCCAACTGAATGAAATAGCCCAAGCTCTGGATGCTTCAAATGTTC
CTTTCATTTTTGTATTGAGGCCTAATGAAAAAACAACGTCGTGGTTGCCAGTTGGTAATTTAGAGGACAAGACTAAAAAGGGTTTGTACATCA
AAGGGTGGGTCCCACAGCTTACGATCATGGAACATTCAGCAACAGGCGGGTTCATGACTCATTGTGGTACTAATTCGGTTCTGGAAGCCATC
ACTTTTGGCGTGCCAATGATAACATGGCCACTTTATGCTGATCAATTCTACAACGAGAAGGTAGTCGAGGTTAGGGGATTGGGA
SGT1_sto40_seq2

CCTTGGACAGTAGATATTGCTGAAGAGCTTCACATTCCTCGTATTTTGTACAATTTGTCTGCTTACATGTGCTACAGTATTATGCACAACCTTAA
GCTTTACAGACCTCACAAGCAGCCTAATCTAGACGAATCTCAGAGTTTCGTGGTTCCTGGTTTACCTGATGAGATAAAGTTCAAGTTATCCCAA
CTGACAGATGATCTGCGAAAGCCGGATGACCAAAAGACTGGTTTTGACGAATTACTCAAACAAGTTCGAGATTCGGAGGAACGAAGCTTTGG
CATTGTTCATGATACATTTTATGAGCTAGAACCTGCATACATCAACTACTATCAGAAATTAAAGAAACCAAAATGTTGGCATTTTGGTCCGCTC
TCTCATTTTGCATCCAAAATCCATAGTAAGGAACTAATTACTGAGCATAACAAC---
GACATCGTTGTAGATTGGTTGAATGCACAGAAACCTAAATCGGTTCTGTATGTATCTTTCGGAAGCATGGCTAGATTTCCTGAGAGCCAACTG
AATGAAATAGCGCATGCTCTGGATGCTTCAAATGTTCCTTTCATTTTTGTATTGAGGCCTAATGAAGAAACAGCGTCCTGGTTGCCAGTTGGTA
ATTTAGAGGACAAGACTAAAAAGGGTTTGTACATCAAAGGATGGGTCCCACAGCTCACGATCATGGAACATTCAGCAACAGGCGGGTTCATG
ACTCATTGTGGTACTAATTCGGTTCTGGAAGCCATCACTTTTGGCGTGCCAATGATAACATGGCCACTTTATGCTGATCAATTCTACAACGAGA
AGGTAGTCGAGGTTAGGGGATTGGGA
SGT1_sto61_seq1
CCTTGGACAGTAGATATTGCTGATGAGCTGCACATCCCTCGTATTTTGTACAATTTGTCTGCTTACATGTGCTACAGCATTATGCACAACCTTAA
GGTTTACAGACCTCACAAGCAGCCTAATCTAGACGAATCTCAAAGTTTCGTGGTTCCTGGTTTACCTGATGAGATAAAGTTCAAGTTATCCCAA
CTGACAGATGATCTGAGAAAGCCGGATGACCAAAAGACTGGTTTTGACGAATTGCTCGAACAAGTTGGAGATTCGGAGGAGCGAAGCTATG
GCATTGTTCATGATACATTTTATGAGCTAGAACCTGCATATGTCGACTACTACCAGAAATTAAAGAAACCAAAATGTTGGCATTTTGGTCCGCT
CTCTCATTTTGCATCCAAAATCCGTAGTAAGGAACTAATTTCTGAGCATAACAACAATGAGATTGTTATAGATTGGTTGAATGCACAGAAACCT
AAATCGGTTCTCTATGTATCTTTCGGAAGCATGGCTAGATTTCCTGAGAGCCAACTGAATGAAATAGCCCAAGCTCTGGATGCTTCAAATGTTC
CTTTCATTTTTGTATTGAGGCCTAATGAAAAAACAACGTCGTGGTTGCCAGTTGGTAATTTAGAGGACAAGACTAAAAAGGGTTTGTACATCA
AAGGGTGGGTCCCACAGCTTACGATCATGGAACATTCAGCAACAGGCGGGTTCATGACTCATTGTGGTACTAATTCGGTTCTGGAAGCCATC
ACTTTTGGCGTGCCAATGATAACATGGCCACTTTATGCTGATCAATTCTACAACGAGAAGGTAGTCGAGGTTAGGGGATTGGGA
SGT1_sto61_seq2
CCTTGGACAGTAGATATTGCTGAAGAGCTTCACATTCCTCGTATTTTGTACAATTTGTCTGCTTACATGTGCTACAGTATTATGCACAACCTTAA
GCTTTACAGACCTCACAAGCAGCCTAATCTAGACGAATCTCAGAGTTTCGTGGTTCCTGGTTTACCTGATGAGATAAAGTTCAAGTTATCCCAA
CTGACAGATGATCTGCGAAAGCCGGATGACCAAAAGACTGGTTTTGACGAATTACTCAAACAAGTTCGAGATTCGGAGGAACGAAGCTTTGG
CATTGTTCATGATACATTTTATGAGCTAGAACCTGCATACATCAACTACTATCAGAAATTAAAGAAACCAAAATGTTGGCATTTTGGTCCGCTC
TCTCATTTTGCATCCAAAATCCATAGTAAGGAACTAATTACTGAGCATAACAAC---
GACATCGTTGTAGATTGGTTGAATGCACAGAAACCTAAATCGGTTCTGTATGTATCTTTCGGAAGCATGGCTAGATTTCCTGAGAGCCAACTG
AATGAAATAGCGCATGCTCTGGATGCTTCAAATGTTCCTTTCATTTTTGTATTGAGGCCTAATGAAGAAACAGCGTCCTGGTTGCCAGTTGGTA
ATTTAGAGGACAAGACTAAAAAGGGTTTGTACATCAAAGGATGGGTCCCACAGCTCACGATCATGGAACATTCAGCAACAGGCGGGTTCATG
ACTCATTGTGGTACTAATTCGGTTCTGGAAGCCATCACTTTTGGCGTGCCAATGATAACATGGCCACTTTATGCTGATCAATTCTACAACGAGA
AGGTAGTCGAGGTTAGGGGATTGGGA
SGT1_chc80-1_seq1
CCTTGGACAGTAGATATTGCTGATGAGCTTCACATCCCTCGTATTTTGTACAATTTGTCTGCTTACATGTGCTACAGCATTATGCACAACCTTAA
GGTTTACAGACCTCACAAGCAGCCTAATCTAGACGAATCTCAAAGTTTCGTGGTTCCTGGTTTACCTGATGAGATAAAGTTCAAGCTATCCCAA
TTGACAGATGATCTGAGAAAGCCGGATGACCAAAAGACTGTTTTTGACGAATTGCTCGAACAAGTTAGAGATTCGGAGGAACGAAGCTATG
GCATTGTTCATGATACATTTTATGAGCTAGAACCTGCATATGTCGACTACTACCAGAAATTAAAGAAATCAAAATGTTGGCATTTTGGTCCGCT
CTCTCATTTTGCATCCAAAATCCGTAGTAAGGAACTAATTTCTGAGCATAACAACAATGAGATTGTTATAGATTGGTTGAATGCACAGAAACCT
AAATCGGTTCTCTATGTATCTTTCGGAAGCATGGCTAGATTTCCTGAGAGCCAACTGAATGAAATAGCCCAAGCTCTGGATGCTTCAAATGTTC
CTTTCATTTTTGTATTGAGGCCTAATGAAGAAACAATGTCGTGGTTGCCAGTTGGTAATTTAGAGGACAAGACTAAAAAGGGTTTGTACATCA
AAGGGTGGGTCCCACAGCTTACGATCATGGAACATTCAGCAACAGGCGGGTTCATGACTCATTGTGGTACTAATTCGGTTCTGGAAGCCATC
ACTTTTGGCGTGCCAATGATAACATGGCCACTTTATGCTGATCAATTCTACAACGAGAAGGTAGTCGAGGTTAGGGGATTGGGA
SGT1_chc80-1_seq2
CCTTGGACAGTAGATATTGCTGATGAGCTTCACATCCCTCGTATTTTGTACAATTTGTCTGCTTACATGTGCTACAGCATTATGCACAACCCTAA
CCATTACAGACCTCACAAGCAGCCTAATCTAGATGAATCTCAAAGTTTTGTGGTTCCTGGTTTACCTGATGAGATAAAGTTCAAGTTATCCCAA
CTGACAGATGATCTGAGAAATTCGGATGACCAAAAGACTGTTTTTGACGAATTGCTCGAACAAGTTGGAGATTCGGAGGAACGAAGCTATGG
CATTGTTCATGATACATTTTATGAGCTAGAACCTGCATATGTCGACTACTACAAGAAATTAAAGAAACCAAAATGTTGGCAGTTTGGTCCGCTC
TCTCATTTTGCATCCAAAATCCGTAGTAGGGAACTAATTTCTAAGGATAACAACAATGAGATCGTTGTAGATTGGTTGAATGCACAGAAACCT
AAATCGGTTCTCTATGTATCTTTCGGAAGCATGGCTAGATTTCCTGAGAGCCAACTGAATGAAATAGCCCAAGCTCTGGAAGCTTCAAATGTT
CCTTTCATTTTTGTATTGAGGCCTAATGAAGAAACGGCGTCGTGGTTGCCAGTTGGTAATTTAGAGGACAAGACTAAAAAGGGTTTGTACATC
AAATGGTGGGTCCCACAACTTACGATCATGGAACATTCAGCAACAGGCAGGTTCATGACTCATTGTGGTACTAATTCGGTTCTGGAAGCCATC
ACTTTTGGCGTGCCAATGATAACATGGCCACTTTATGTTGATCAATTCTACAATGAGAAGGTAGTCGAGGTTAGGGGATTGGGA
SGT1_phuDM_seq1
CCTTGGACAGTAGATATTGCTGATGAGCTTCACATCCCTCGTATTTTGTACAATTTGTCTGCTTACATGTGCTACAGCATTATGCACAACCTTAA
GCTTTACAGACCTCACAAGCAGCCTAATCTAGACGAATCTCAAAGTTTCGTGGTTCCTGGTTTACCTGATGAGATGAAGTTCAAGTTATCCCAA
CTGACAGATGATCTGAGAAAACCGGATGACCAAAAGACTGGTTTTGACGAATTGCTCGATAAAGTACGAGATTCGGAGGAACGAAGCTATG
GCATTGTTCATGATACATTTTATGAGCTAGAACCTGCATATGTCGACTACTACCAGAAATTAAAGAAACCAAAATGTTGGCATTTTGGTCCGCT
CTCTCATTTTGCATCCAAAATCCGTAGTAAGGAGCTAATTGCTGAGCATAACAACAATGAGATTGTTGTAGATTGGTTGAATGCACAGAAACC
TAAATCGGTTCTCTATGTATCTTTCGGAAGCATGGCTAGATTTCCTGAGAGCCAACTGAATGAAATATCCCAAGCTCTGGATGCTTCAAATGTT

CCTTTCATTTTTGTATTGAGGCCTAATGAAGAAACGGCGTCGTGGTTGCCAGTTGGTAATTTAGAGGACAAGACTAAAAAGGGTTTGTACATC
AAAGGGTGGGTCCCACAGCTTACGATCATGGAACATTCAGCAACAGGCGGGTTCATGACTCATTGTGGTACTAATTCGGTTCTGGAAGCCAT
CACTTTTGGCGTGCCAATGATAACATGGCCACTTTATGCTGATCAATTCTACAACGAGAAGGTAGTCGAGGTTAGGGGATTGGGA
SGT1_phuDM_seq1-2
CCTTGGACAGTAGATATTGCTGATGAGCTTCACATCCCTCGTATTTTGTACAATTTGTCTGCTTACATGTGCTACAGCATTATGCACAACCTTAA
GCTTTACAGACCTCACAAGCAGCCTAATCTAGACGAATCTCAAAGTTTCGTGGTTCCTGGTTTACCTGATGAGATGAAGTTCAAGTTATCCCAA
CTGACAGATGATCTGAGAAAACCGGATGACCAAAAGACTGGTTTTGACGAATTGCTCGATAAAGTACGAGATTCGGAGGAACGAAGCTATG
GCATTGTTCATGATACATTTTATGAGCTAGAACCTGCATATGTCGACTACTACCAGAAATTAAAGAAACCAAAATGTTGGCATTTTGGTCCGCT
CTCTCATTTTGCATCCAAAATCCGTAGTAAGGAGCTAATTGCTGAGCATAACAACAATGAGATTGTTGTAGATTGGTTGAATGCACAGAAACC
TAAATCGGTTCTCTATGTATCTTTCGGAAGCATGGCTAGATTTCCTGAGAGCCAACTGAATGAAATATCCCAAGCTCTGGATGCTTCAAATGTT
CCTTTCATTTTTGTATTGAGGCCTAATGAAGAAACGGCGTCGTGGTTGCCAGTTGGTAATTTAGAGGACAAGACTAAAAAGGGTTTGTACATC
AAAGGGTGGGTCCCACAGCTTACGATCATGGAACATTCAGCAACAGGCGGGTTCATGACTCATTGTGGTACTAATTCGGTTCTGGAAGCCAT
CACTTTTGGCGTGCCAATGATAACATGGCCACTTTATGCTGATCAATTCTACAACGAGAAGGTAGTCGAGGTTAGGGGATTGGGA
SGT1_phuDH_seq1
CCTTGGACAGTAGATATTGCTGATGAGCTTCACATCCCTCGTATTTTGTACAATTTGTCTGCTTACATGTGCTACAGCATTATGCACAACCTTAA
GCTTTACAGACCTCACAAGCAGCCTAATCTAGACGAATCTCAAAGTTTCGTGGTTCCTGGTTTACCTGATGAGATGAAGTTCAAGTTATCCCAA
CTGACAGATGATCTGAGAAAACCGGATGACCAAAAGACTGGTTTTGACGAATTGCTCGATAAAGTACGAGATTCGGAGGAACGAAGCTATG
GCATTGTTCATGATACATTTTATGAGCTAGAACCTGCATATGTCGACTACTACCAGAAATTAAAGAAACCAAAATGTTGGCATTTTGGTCCGCT
CTCTCATTTTGCATCCAAAATCCGTAGTAAGGAGCTAATTGCTGAGCATAACAACAATGAGATTGTTGTAGATTGGTTGAATGCACAGAAACC
TAAATCGGTTCTCTATGTATCTTTCGGAAGCATGGCTAGATTTCCTGAGAGCCAACTGAATGAAATATCCCAAGCTCTGGATGCTTCAAATGTT
CCTTTCATTTTTGTATTGAGGCCTAATGAAGAAACGGCGTCGTGGTTGCCAGTTGGTAATTTAGAGGACAAGACTAAAAAGGGTTTGTACATC
AAAGGGTGGGTCCCACAGCTTACGATCATGGAACATTCAGCAACAGGCGGGTTCATGACTCATTGTGGTACTAATTCGGTTCTGGAAGCCAT
CACTTTTGGCGTGCCAATGATAACATGGCCACTTTATGCTGATCAATTCTACAACGAGAAGGTAGTCGAGGTTAGGGGATTGGGA
SGT1_phuDH_seq2
CCTTGGACAGTAGATATTGCTGATGAGCTTCACATCCCTCGTATTTTGTACAATTTGTCTGCTTACATGTGCTACAGCATTATGCACAACCTTAA
GGTTTACAGACCTCACAAGCAGCCTAATCTAGACGAATCTCAAAGTTTCGTGGTTCCTGGTTTACCTGATGAGATAAAGTTCAAGTTATCCCAA
CTGACAGATGATCTGAGAAAGCCGGATGACCAAAAGACTGTTTTTGACGAATTGCTCGAACAAATTGGAGATTCGGAGGAACGAAGCTATG
GCATTGTTCATGATACATTTTATGAGCTAGAACCTGCATATGTCGACTACTACCAGAAATTAAAGAAACCAAAATGTTGGCATTTTGGTCCGCT
CTCTCATTTTGCATCCAAAATCCGTAGTAAGGAACTAATTTCTGAGCATAACAACAATGAGATTGTTATAGATTGGTTGAATGCACAGAAACCT
AAATCGGTTCTCTATGTATCTTTCGGAAGCATGGCTAGATTTCCTGAGAGCCAACTGAATGAAATAGCCCAAGCTCTGGATGCTTCAAATGTTC
CTTTCATTTTTGTATTGAGGCCTAATGAAGAAACGGCGTCGTGGTTGCCCGTTGGTAATTTAGAGGACAAGACTAAAAAGGGTTTGTACATCA
AAGGGTGGGTCCCACAGCTTACGATCATGGAACATTCAGCAACAGGCGGGTTCATGACTCATTGTGGTACTAATTCGGTTCTGGAAGCCATC
ACTTTTGGCGTGCCAATGATAACATGGCCACTTTATGCTGATCAATTCTACAACGAGAAGGTAGTCGAGGTTAGGGGATTGGGA
SGT1_tbr_seq1
CCTTGGACAGTAGATATTGCTGATGAGCTTCACATCCCTCGTATTTTGTACAATTTGTCTGCTTACATGTGCTACAGCATTATGCACAACCTTAA
GGTTTACAGACCTCACAAGCAGCCTAATCTAGACGAATCTCAAAGTTTCGTGGTTCCTGGTTTACCTGATGAGATAAAGTTCAAGTTATCCCAA
CTGACAGATGATCTGAGAAAGTCGGATGACCAAAAGACTGTTTTTGACGAATTGCTCGAACAAGTTGAAGATTCGGAGGAACGAAGCTATG
GCATTGTTCATGATACATTTTATGAGCTAGAACCTGCATATGTTGACTACTACCAGAAATTAAAGAAACCAAAATGTTGGCATTTTGGTCCGCT
CTCTCATTTTGCATCCAAAATCCGTAGTAAGGAACTAATTTCTGAGCATAACAACAATGAGATTGTTATAGATTGGTTGAATGCACAGAAACCT
AAATCGGTTCTCTATGTATCTTTCGGAAGCATGGCTAGATTTCCTGAGAGCCAACTGAATGAAATAGCCCAAGCTCTGGATGCTTCAAATGTTC
CTTTCATTTTTGTATTGAGGCCTAATGAAGAAACGGCGTCGTGGTTGCCAGTTGGTAATTTAGAGGACAAGACTAAAAAGGGTTTGTACATCA
AAGGGTGGGTCCCACAGCTTACGATCATGGAACATTCAGCAACAGGCGGGTTCATGACTCATTGTGGTACTAATTCGGTTCTGGAAGCCATC
ACTTTTGGCGTGCCAATGATAACATGGCCACTTTATGCTGATCAATTCTACAACGAGAAGGTAGTCGAGGTTAGGGGATTGGGA
SGT1_tbr_seq1-2
CCTTGGACAGTAGATATTGCTGATGAGCTTCACATCCCTCGTATTTTGTACAATTTGTCTGCTTACATGTGCTACAGCATTATGCACAACCTTAA
GGTTTACAGACCTCACAAGCAGCCTAATCTAGACGAATCTCAAAGTTTCGTGGTTCCTGGTTTACCTGATGAGATAAAGTTCAAGTTATCCCAA
CTGACAGATGATCTGAGAAAGTCGGATGACCAAAAGACTGTTTTTGACGAATTGCTCGAACAAGTTGAAGATTCGGAGGAACGAAGCTATG
GCATTGTTCATGATACATTTTATGAGCTAGAACCTGCATATGTTGACTACTACCAGAAATTAAAGAAACCAAAATGTTGGCATTTTGGTCCGCT
CTCTCATTTTGCATCCAAAATCCGTAGTAAGGAACTAATTTCTGAGCATAACAACAATGAGATTGTTATAGATTGGTTGAATGCACAGAAACCT
AAATCGGTTCTCTATGTATCTTTCGGAAGCATGGCTAGATTTCCTGAGAGCCAACTGAATGAAATAGCCCAAGCTCTGGATGCTTCAAATGTTC
CTTTCATTTTTGTATTGAGGCCTAATGAAGAAACGGCGTCGTGGTTGCCAGTTGGTAATTTAGAGGACAAGACTAAAAAGGGTTTGTACATCA
AAGGGTGGGTCCCACAGCTTACGATCATGGAACATTCAGCAACAGGCGGGTTCATGACTCATTGTGGTACTAATTCGGTTCTGGAAGCCATC
ACTTTTGGCGTGCCAATGATAACATGGCCACTTTATGCTGATCAATTCTACAACGAGAAGGTAGTCGAGGTTAGGGGATTGGGA

1.5 Solanidine glucosyltransferase (*SGT2*) sequences
27
SGT2_cmm7_seq1
CCTGCGGATGAGAGGAATGCTTTTGATGAATTGCTTGATCAAACCAGAGAATCTGAGGATCGAAGCTATGGTATCGTTCACGATACCTTTTAC
GAGCTAGAACCTGACTACGCTGAGTACTATCAGAAGATGAAGAAAACCAAATGTTGGCAAATTGGTCCCATTTCCTATTTTTCTTCCAAATTAT
CCCGAAGAAAAGAGCTGATTAGTTCTGCTGATGAAAGTATTTCATCTGTTGTGGAGTGGTTGAATAAACAAAAGCACAAATCGGTCCTCTACG
TCTCTTTCGGGAGCATAGTTACATTCCCAGAGGAGCAACTCGCTGAAATCGCAAAAGCTCTAGAAGCTTCTACCGTCCCTTTCATTTGGGCAGT
GAAGAAAGACCAATCAGCAAAAACCACGTGGTTACCGGAGAGTTTGTTCGATGAGAAAAAAGGTCTGATTATTAAAGGGTGGGCGCCGCAA
CTAACCATCTTAGATCATTCAGCAATAGGAGGATTCATGACACACTGTGGATGGAATTCGGTGCTTGAAGCTATCATCGCTGGGGTGCCGTTG
GTG
SGT2_cmm7_seq1-2
CCTGCGGATGAGAGGAATGCTTTTGATGAATTGCTTGATCAAACCAGAGAATCTGAGGATCGAAGCTATGGTATCGTTCACGATACCTTTTAC
GAGCTAGAACCTGACTACGCTGAGTACTATCAGAAGATGAAGAAAACCAAATGTTGGCAAATTGGTCCCATTTCCTATTTTTCTTCCAAATTAT
CCCGAAGAAAAGAGCTGATTAGTTCTGCTGATGAAAGTATTTCATCTGTTGTGGAGTGGTTGAATAAACAAAAGCACAAATCGGTCCTCTACG
TCTCTTTCGGGAGCATAGTTACATTCCCAGAGGAGCAACTCGCTGAAATCGCAAAAGCTCTAGAAGCTTCTACCGTCCCTTTCATTTGGGCAGT
GAAGAAAGACCAATCAGCAAAAACCACGTGGTTACCGGAGAGTTTGTTCGATGAGAAAAAAGGTCTGATTATTAAAGGGTGGGCGCCGCAA
CTAACCATCTTAGATCATTCAGCAATAGGAGGATTCATGACACACTGTGGATGGAATTCGGTGCTTGAAGCTATCATCGCTGGGGTGCCGTTG
GTG
SGT2_cmm26_seq1
CCTGCGGATGAGAGGAATGCTTTTGATGAATTGCTTGATCAAACCAGAGAATCTGAGGATCGAAGCTATGGTATCGTTCACGATACCTTTTAC
GAGCTAGAACCTGACTACGCTGAGTACTATCAGAAGATGAAGAAAACCAAATGTTGGCAAATTGGTCCCATTTCCTATTTTTCTTCCAAATTAT
CCCGAAGAAAAGAGCTGATTAGTTCTGCTGATGAAAGTATTTCATCTGTTGTGGAGTGGTTGAATAAACAAAAGCACAAATCGGTCCTCTACG
TCTCTTTCGGGAGCATAGTTACATTCCCAGAGGAGCAACTCGCTGAAATCGCAAAAGCTCTAGAAGCTTCTACCGTCCCTTTCATTTGGGCAGT
GAAGAAAGACCAATCAGCAAAAACCACGTGGTTACCGGAGAGTTTGTTCGATGAGAAAAAAGGTCTGATTATTAAAGGGTGGGCGCCGCAA
CTAACCATCTTAGATCATTCAGCAATAGGAGGATTCATGACACACTGTGGATGGAATTCGGTGCTTGAAGCTATCATCGCTGGGGTGCCGTTG
GTG
SGT2_cmm26_seq1-2
CCTGCGGATGAGAGGAATGCTTTTGATGAATTGCTTGATCAAACCAGAGAATCTGAGGATCGAAGCTATGGTATCGTTCACGATACCTTTTAC
GAGCTAGAACCTGACTACGCTGAGTACTATCAGAAGATGAAGAAAACCAAATGTTGGCAAATTGGTCCCATTTCCTATTTTTCTTCCAAATTAT
CCCGAAGAAAAGAGCTGATTAGTTCTGCTGATGAAAGTATTTCATCTGTTGTGGAGTGGTTGAATAAACAAAAGCACAAATCGGTCCTCTACG
TCTCTTTCGGGAGCATAGTTACATTCCCAGAGGAGCAACTCGCTGAAATCGCAAAAGCTCTAGAAGCTTCTACCGTCCCTTTCATTTGGGCAGT
GAAGAAAGACCAATCAGCAAAAACCACGTGGTTACCGGAGAGTTTGTTCGATGAGAAAAAAGGTCTGATTATTAAAGGGTGGGCGCCGCAA
CTAACCATCTTAGATCATTCAGCAATAGGAGGATTCATGACACACTGTGGATGGAATTCGGTGCTTGAAGCTATCATCGCTGGGGTGCCGTTG
GTG
SGT2_dms54_seq1
CCTGCGGATGAGAGGAATGCTTTTGATGAATTGCTCGATCGAACCAGAGAATCTGAGGATCTAAGCTACGGTATCGTTCATGATACTTTTTAC
GAGCTAGAACCTGCCTACGCTGACTACTATCAGAAGATGAAGAAAACCAAATGTTGGCAAATTGGTCCCATTTCCTATTTTTCTTCCAAATTAT
CCCCAAGAAAAGAACTGATTAATTCTTCTGATGAAAGTAACTCATCTGCCGTTGTTGTAGAGTGGTTGAATAAACATAAGCACAAATCGGTCC
TCTACGTCTCTTTTGGGAGCACAATTAGATTCCCAGAGGAGCAACTCGCTGAAATCGCAAAAGCTCTAGAAGCTTCTACCGTCCCTTTCATTTG
GGTAGTAAACAAAGACCAATTAGCAAAAACCACGTGGTTACCGGAGAGTTTGTTCGATGAGAAAAAAATGTCTGATTATTAAAGGGTGGGCAC
CGCAACTATCCATCTTAGATCATTCAGCAGTCGGAGGATTCATGACACACTGTGGTTGGAATTCAGTGCTTGAAGCCATCATCGCTGGGGTGC
CGTTGGTG
SGT2_dms54_seq1-2
CCTGCGGATGAGAGGAATGCTTTTGATGAATTGCTCGATCGAACCAGAGAATCTGAGGATCTAAGCTACGGTATCGTTCATGATACTTTTTAC
GAGCTAGAACCTGCCTACGCTGACTACTATCAGAAGATGAAGAAAACCAAATGTTGGCAAATTGGTCCCATTTCCTATTTTTCTTCCAAATTAT
CCCCAAGAAAAGAACTGATTAATTCTTCTGATGAAAGTAACTCATCTGCCGTTGTTGTAGAGTGGTTGAATAAACATAAGCACAAATCGGTCC
TCTACGTCTCTTTTGGGAGCACAATTAGATTCCCAGAGGAGCAACTCGCTGAAATCGCAAAAGCTCTAGAAGCTTCTACCGTCCCTTTCATTTG
GGTAGTAAACAAAGACCAATTAGCAAAAACCACGTGGTTACCGGAGAGTTTGTTCGATGAGAAAAAAATGTCTGATTATTAAAGGGTGGGCAC
CGCAACTATCCATCTTAGATCATTCAGCAGTCGGAGGATTCATGACACACTGTGGTTGGAATTCAGTGCTTGAAGCCATCATCGCTGGGGTGC
CGTTGGTG
SGT2_dms78_seq1
CCTGCGGATGAGAGGAATGCTTTTGATGAATTGCTCGATCGAACCAGAGAATCTGAGGATCTAAGCTACGGTATCGTTCATGATACTTTTTAC
GAGCTAGAACCTGCCTACGCTGACTACTATCAGAAGATGAAGAAAACCAAATGTTGGCAAATTGGTCCCATTTCCTATTTTTCTTCCAAATTAT
CCCCAAGAAAAGAACTGATTAATTCTTCTGATGAAAGTAACTCATCTGCCGTTGTTGTAGAGTGGTTGAATAAACATAAGCACAAATCGGTCC
TCTACGTCTCTTTTGGGAGCACAATTAGATTCCCAGAGGAGCAACTCGCTGAAATCGCAAAAGCTCTAGAAGCTTCTACCGTCCCTTTCATTTG
GGTAGTAAACAAAGACCAATTAGCAAAAACCACGTGGTTACCGGAGAGTTTGTTCGATGAGAAAAAAATGTCTGATTATTAAAGGGTGGGCAC
CGCAACTATCCATCTTAGATCATTCAGCAGTCGGAGGATTCATGACACACTGTGGTTGGAATTCAGTGCTTGAAGCCATCATCGCTGGGGTGC
CGTTGGTG

SGT2_dms78_seq1-2
CCTGCGGATGAGAGGAATGCTTTTGATGAATTGCTCGATCGAACCAGAGAATCTGAGGATCTAAGCTACGGTATCGTTCATGATACTTTTTAC
GAGCTAGAACCTGCCTACGCTGACTACTATCAGAAGATGAAGAAAACCAAATGTTGGCAAATTGGTCCCATTTCCTATTTTTCTTCCAAATTAT
CCCCAAGAAAAGAACTGATTAATTCTTCTGATGAAAGTAACTCATCTGCCGTTGTTGTAGAGTGGTTGAATAAACATAAGCACAAATCGGTCC
TCTACGTCTCTTTTGGGAGCACAATTAGATTCCCAGAGGAGCAACTCGCTGAAATCGCAAAAGCTCTAGAAGCTTCTACCGTCCCTTTCATTTG
GGTAGTAAACAAAGACCAATTAGCAAAAACCACGTGGTTACCGGAGAGTTTGTTCGATGAGAAAAAAATGTCTGATTATTAAAGGGTGGGCAC
CGCAACTATCCATCTTAGATCATTCAGCAGTCGGAGGATTCATGACACACTGTGGTTGGAATTCAGTGCTTGAAGCCATCATCGCTGGGGTGC
CGTTGGTG
SGT2_spg55_seq2
CCTGCGGATGAGAGGAATGCTTTTGATGAATTGCTCGATCGAACCAGAGAATCTGAGGATCAAAGCTACGGTATTGTTCATGATACTTTTTAC
GAGCTAGAACCTGCCTACGCTGACTACTATCAGAAGATGAAGAAAACCAAATGTTGGCAAATTGGTCCCATTTCCTATTTTTCTTCCAATTTAT
TCCGAAGAAAAGATCTGATTAATTCTTTTGATGAAAGTAACTCATCTGCCGCTGTTGTAGAGTGGTTAAATAAACAGAAGCACAAATCGGTCC
TCTACGTCTCTTTCGGGAGCACAGTTAAATTCCCAGAGGAGCAACTCGCTGAAATCGCAAAATCTCTAGAAGCTTCTACCGTCCCTTTCATTTG
GGTAGTGAAGAAGGACCAATCAGCAAAAACCACGTGGTTACCGGAGAGTTTGGTCGATGAGAAAAAAGGTCTGATTATTAATGGGTGGGCG
CCGCAACTAACCATCTTAGATCATTCAGCAGTAGGAGGATTCATGACACACTGTGGATGGAATTCGGTGCTTGAAGCTATCATCGCTGGGGT
GCCGTTGGTG
SGT2_spg74_seq3
CCTGCGGATGAGAGGAATGCTTTTGATGAATTGCTCGATCGAACCAGAGAATCTGAGGATCTAAGCTACGGTATCGTTCATGATACTTTTTAC
GAGCTAGAACCTGCCTACGCTGACTACTATCAGAAGATGAAGAAAACCAAATGTTGGCAAATTGGTCCCATTTCCTATTTTTCTTCCAAATTAT
CCCCAAGAAAAGAACTGATTAATTCTTCTGATGAAAGTAACTCATCTGCCGTTGTTGTAGAGTGGTTGAATAAACATAAGCACAAATCAGTCC
TCTACGTCTCTTTTGGGAGCACAATTAGATTCCCAGAGGAGCAACTCGCTGAAATCGCAAAAGCTCTAGAAGCTTCTACCGTCCCTTTCATTTG
GGTAGTGAAGGAGGACCAATCAGCAAAAACCACGTGGTTACCGGAGAGTTTATTCGATGAGAAAAAAGGTCTGATTATTAATGGGTGGGCG
CCGCAACTAACCATCTTAGATCATTCAGCAGTAGGAGGATTCATGACACACTGTGGATGGAATTCGGTGCTTGAAGCCATCATCGCTGGGGT
GCCGTTGGTG
SGT2_spg74_seq4
CCTGCGGATGAGAGGAATGCTTTTGATGAATTGCTCGATCGAACCAGAGAATCTGAGGATCTAAGCTACGGTATCGTTCATGATACTTTTTAC
GAGCTAGAACCTGCCTACGCTGACTACTATCAGAAGATGAAGAAAACCAAATGTTGGCAAATTGGTCCCATTTCCTATTTTTCTTCCAAATTAT
CCCCAAGAAAAGAACTGATTAATTCTTCTGATGAAAGTAACTCATCTGCCGTTGTTGTAGAGTGGTTGAATAAACATAAGCACAAATCAGTCC
TCTACGTCTCTTTTGGGAGCACAATTAGATTCCCAGAGGAGCAACTCGCTGAAATCGCAAAAGCTCTAGAAGCTTCTACCGTCCCTTTCATTTG
GGTAGTGAAGGAGGACCAATCAGCAAAAACCACGTGGTTACCGGAGAGTTTATTCGATGAGAAAAAAGGTCTGATTATTAATGGGTGGGCG
CCGCAACTAACCATCTTAGATCATTCAGCAGTCGGAGGATTCATGACACACTGTGGTTGGAATTCAGTGCTTGAAGCCATCATCGCTGGGGTG
CCGTTGGTG
SGT2_spl16_seq1
CCTGCGGATGAGAGGAATGCTTTTGATGAATTGCTCGATCGAACCAGAGAATCTGAGGATCTAAGCTACGGTATCGTTCATGATACTTTTTAC
GAGCTAGAACCTGACTACGCTGACTACTATCAGAAGATGAAGAAAACCAAATGTTGGCAAATTGGTCCCATTTCCTATTTTTCTTCCAAATTAT
CCCCAAGAAAAGAACTGATTAATTCTTCTGATGAAAGTAACTCATCTGCCGTTGTTGTAGAGTGGTTGAATAAACATAAGCACAAATCGGTCC
TCTACGTCTCTTTTGGGAGCACAATTAGATTCCCAGAGGAGCAACTCGCTGAAATCGCAAAAGCTCTAGAAGCTTCTACCGTCCCTTTCATTTG
GGTAGTAAACAAAGACCAATTAGCAAAAACCACGTGGTTACCGGAGAGTTTGTTCGATGAGAAAAAAATGTCTGATTATTAAAGGGTGGGCAC
CGCAACTAACCATCTTAGATCATTCAGCAGTCGGAGGATTCATGACACACTGTGGTTGGAATTCAGTGCTTGAAGCCATCATCGCTGGGGTGC
CGTTGGTG
SGT2_spl16_seq1-2
CCTGCGGATGAGAGGAATGCTTTTGATGAATTGCTCGATCGAACCAGAGAATCTGAGGATCTAAGCTACGGTATCGTTCATGATACTTTTTAC
GAGCTAGAACCTGACTACGCTGACTACTATCAGAAGATGAAGAAAACCAAATGTTGGCAAATTGGTCCCATTTCCTATTTTTCTTCCAAATTAT
CCCCAAGAAAAGAACTGATTAATTCTTCTGATGAAAGTAACTCATCTGCCGTTGTTGTAGAGTGGTTGAATAAACATAAGCACAAATCGGTCC
TCTACGTCTCTTTTGGGAGCACAATTAGATTCCCAGAGGAGCAACTCGCTGAAATCGCAAAAGCTCTAGAAGCTTCTACCGTCCCTTTCATTTG
GGTAGTAAACAAAGACCAATTAGCAAAAACCACGTGGTTACCGGAGAGTTTGTTCGATGAGAAAAAAATGTCTGATTATTAAAGGGTGGGCAC
CGCAACTAACCATCTTAGATCATTCAGCAGTCGGAGGATTCATGACACACTGTGGTTGGAATTCAGTGCTTGAAGCCATCATCGCTGGGGTGC
CGTTGGTG
SGT2_spl81_seq2
CCTGCGGATGAGAGGAATGCTTTTGATGAATTGCTCGATCGAACCAGAGAATCTGAGGATCTAAGCTACGGTATCGTTCATGATACTTTTTAC
GAGCTAGAACCTGCCTACGCTGACTACTATCAGAAGATGAAGAAAACCAAATGTTGGCAAATTGGTCCCATTTCCTATTTTTCTTCCAAATTAT
CCCCAAGAAAAGAACTGATTAATTCTTCTGATGAAAGTAACTCATCTGCCGTTGTTGTAGAGTGGTTGAATAAACATAAGCACAAATCGGTCC
TCTACGTCTCTTTTGGGAGCACAATTAGATTCCCAGAGGAGCAACTCGCTGAAATCGCAAAAGCTCTAGAAGCTTCTACCGTCCCTTTCATTTG
GGTAGTAAACAAAGACCAATTAGCAAAAACCACGTGGTTACCGGAGAGTTTGTTCGATGAGAAAAAAATGTCTGATTATTAAAGGGTGGGCAC
CGCAACTAACCATCTTAGATCATTCAGCAGTCGGAGGATTCATGACACACTGTGGTTGGAATTCAGTGCTTGAAGCCATCATCGCTGGGGTGC
CGTTGGTG
SGT2_spl81_seq3

CCTGCGGATGAGAGGAATGCTTTTGATGAATTGCTCGATCGAACCAGAGAATCTGAGGATCTAAGCTACGGTATCGTTCATGATACTTTTTAC
GAGCTAGAACCTGCCTACGCTGACTACTATCAGAAGATGAAGAAAACCAAATGTTGGCAAATTGGTCCCATTTCCTATTTTTCTTCCAAATTAT
CCCCAAGAAAAGAACTGATTAATTCTTCTGATGAAAGTAACTCATCTGCCGTTGTTGTAGAGTGGTTGAATAAACATAAGCACAAATCGGTCC
TCTACGTCTCTTTTGGGAGCACAATTAGATTCCCAGAGGAGCAACTCGCTGAAATCGCAAAAGCTCTAGAAGCTTCTACCGTCCCTTTCATTTG
GGTAGTAAACAAAGACCAATTAGCAAAAACCACGTGGTTACCGGAGAGTTTGTTCGATGAGAAAAAAATGTCTGATTATTAAAGGATGGGCAC
CGCAACTAACCATCTTAGATCATTCAGCAGTCGGAGGATTCATGACACACTGTGGTTGGAATTCAGTGCTTGAAGCCATCATCGCTGGGGTGC
CGTTGGTG
SGT2_sto40_seq1
CCTGCGGATGAGAGGAATGCTTTTGATGAATTGCTCGATCGAACCAGAGAATCTGAGGATCTAAGCTACGGTATCGTTCATGATACTTTTTAC
GAGCTAGAACCTGCCTACGCTGACTACTATCAGAAGATGAAGAAAACCAAATGTTGGCAAATTGGTCCCATTTCCTATTTTTCTTCCAAATTAT
CCCCAAGAAAAGAACTGATTAATTCTTCTGATGAAAGTAACTCATCTGCCGTTGTTGTAGAGTGGTTGAATAAACATAAGCACAAATCGGTCC
TCTACGTCTCTTTTGGGAGCACAATTAGATTCCCAGAGGAGCAACTCGCTGAAATCGCAAAAGCTCTAGAAGCTTCTACCGTCCCTTTCATTTG
GGTAGTAAACAAAGACCAATTAGCAAAAACCACGTGGTTACCGGAGAGTTTGTTCGATGAGAAAAAAATGTCTGATTATTAAAGGGTGGGCAC
CGCAACTATCCATCTTAGATCATTCAGCAGTCGGAGGATTCATGACACACTGTGGTTGGAATTCAGTGCTTGAAGCCATCATCGCTGGGGTGC
CGTTGGTG
SGT2_sto40_seq1-2
CCTGCGGATGAGAGGAATGCTTTTGATGAATTGCTCGATCGAACCAGAGAATCTGAGGATCTAAGCTACGGTATCGTTCATGATACTTTTTAC
GAGCTAGAACCTGCCTACGCTGACTACTATCAGAAGATGAAGAAAACCAAATGTTGGCAAATTGGTCCCATTTCCTATTTTTCTTCCAAATTAT
CCCCAAGAAAAGAACTGATTAATTCTTCTGATGAAAGTAACTCATCTGCCGTTGTTGTAGAGTGGTTGAATAAACATAAGCACAAATCGGTCC
TCTACGTCTCTTTTGGGAGCACAATTAGATTCCCAGAGGAGCAACTCGCTGAAATCGCAAAAGCTCTAGAAGCTTCTACCGTCCCTTTCATTTG
GGTAGTAAACAAAGACCAATTAGCAAAAACCACGTGGTTACCGGAGAGTTTGTTCGATGAGAAAAAAATGTCTGATTATTAAAGGGTGGGCAC
CGCAACTATCCATCTTAGATCATTCAGCAGTCGGAGGATTCATGACACACTGTGGTTGGAATTCAGTGCTTGAAGCCATCATCGCTGGGGTGC
CGTTGGTG
SGT2_sto61_seq2
CCTGCGGATGAGAGGAATGCTTTTGATGAATTGCTCGATCGAACCAGAGAATCTGAGGATCTAAGCTACGGTATCGTTCATGATACTTTTTAC
GAGCTAGAACCTGCCTACGCTGACTACTATCAGAAGATGAAGAAAACCAAATGTTGGCAAATTGGTCCCATTTCCTATTTTTCTTCCAAATTAT
CCCCAAGAAAAGAACTGATTAATTCTTCTGATGAAAGTAACTCATCTGCCGTTGTTGTAGAGTGGTTGAATAAACATAAGCACAAATCGGTCC
TCTACGTCTCTTTTGGGAGCACAATTAGATTCCCAGAGGAGCAACTCGCTGAAATCGCAAAAGCTCTAGAAGCTTCTACCGTCCCTTTCATTTG
GGTAGTAAACAAAGACCAATTAGCAAAAACCACGTGGTTACCGGAGAGTTTGTTCGATGAGAAAAAAATGTCTGATTATTAAAGGGTGGGCAC
CGCAACTATCAATCTTAGATCATTCAGCAGTCGGAGGATTCATGACACACTGTGGTTGGAATTCAGTGCTTGAAGCCATCATCGCTGGGGTGC
CGTTGGTG
SGT2_sto61_seq2-2
CCTGCGGATGAGAGGAATGCTTTTGATGAATTGCTCGATCGAACCAGAGAATCTGAGGATCTAAGCTACGGTATCGTTCATGATACTTTTTAC
GAGCTAGAACCTGCCTACGCTGACTACTATCAGAAGATGAAGAAAACCAAATGTTGGCAAATTGGTCCCATTTCCTATTTTTCTTCCAAATTAT
CCCCAAGAAAAGAACTGATTAATTCTTCTGATGAAAGTAACTCATCTGCCGTTGTTGTAGAGTGGTTGAATAAACATAAGCACAAATCGGTCC
TCTACGTCTCTTTTGGGAGCACAATTAGATTCCCAGAGGAGCAACTCGCTGAAATCGCAAAAGCTCTAGAAGCTTCTACCGTCCCTTTCATTTG
GGTAGTAAACAAAGACCAATTAGCAAAAACCACGTGGTTACCGGAGAGTTTGTTCGATGAGAAAAAAATGTCTGATTATTAAAGGGTGGGCAC
CGCAACTATCAATCTTAGATCATTCAGCAGTCGGAGGATTCATGACACACTGTGGTTGGAATTCAGTGCTTGAAGCCATCATCGCTGGGGTGC
CGTTGGTG
SGT2_chc80-1_seq1
CCTGCGGATTGGAGGAATGCTTTTGATGAATTGCTTGATCGAACCAGAGAATCTGAGGATCTAAGCTACGGTATCATTCATGATACTTTTTAC
GAGCTAGAACCTGCCTACGCTGAGTACTATCAGAAGATGAAGAAAACCAAATGTTGGCAAATTGGTCCCATTTCCTATTTTTCTTCCAAATTAT
TCCGAAGAAAAGAACTGATTAATTCTTCTGATGAAAGTAACTCATCTGCCGTTGTTGTAGAGTGGTTGAATAAACAGAAGCACAAATCGGTCC
TCTACGTCTCTTTTGGGAGCACAGTTAGATTCCCAGAGGAGCAACTCGCTGAAATCGCAAAAGCTCTAGAAGCTTCTACCGTCCCTTTCATTTG
GGCAGTGAACAAGGACCAATCAGCAAAAATCACGTGGTTACCGGAGAGTTTGTTCGATGAGAAAAAAATGTCTGATTATTAAAGGGTGGGCG
CCGCAACTAACCATCTTAGATCATTCAGCAGTCGGAGGATTCATGACACACTGTGGTTGGAATTCAGTGCTTGAAGCCATCATCGCTGGGGTG
CCGTTGGTG
SGT2_chc80-1_seq1-2
CCTGCGGATTGGAGGAATGCTTTTGATGAATTGCTTGATCGAACCAGAGAATCTGAGGATCTAAGCTACGGTATCATTCATGATACTTTTTAC
GAGCTAGAACCTGCCTACGCTGAGTACTATCAGAAGATGAAGAAAACCAAATGTTGGCAAATTGGTCCCATTTCCTATTTTTCTTCCAAATTAT
TCCGAAGAAAAGAACTGATTAATTCTTCTGATGAAAGTAACTCATCTGCCGTTGTTGTAGAGTGGTTGAATAAACAGAAGCACAAATCGGTCC
TCTACGTCTCTTTTGGGAGCACAGTTAGATTCCCAGAGGAGCAACTCGCTGAAATCGCAAAAGCTCTAGAAGCTTCTACCGTCCCTTTCATTTG
GGCAGTGAACAAGGACCAATCAGCAAAAATCACGTGGTTACCGGAGAGTTTGTTCGATGAGAAAAAAATGTCTGATTATTAAAGGGTGGGCG
CCGCAACTAACCATCTTAGATCATTCAGCAGTCGGAGGATTCATGACACACTGTGGTTGGAATTCAGTGCTTGAAGCCATCATCGCTGGGGTG
CCGTTGGTG
SGT2_phuDH_seq1
CCTGCGGATGAGAGGAATGCTTTTGATGAATTGCTCGATCGAACCAGAGAATCTGAGGATCTAAGCTACGGTATCGTTCATGATACTTTTTAC
GAGCTAGAACCTGCCTACGCTGACTACTATCAAAAGATGAAGAAAACCAAATGTTGGCAAATTGGTCCCATTTCCTATTTTTCTTCCAAATTAT

TCCGAAGAAAAGAACTGATTAATTCTTCTGATGAAAGTAACTCATCTGCCGTTGTTGTAGAGTGGTTGAATAAACACAAGCACAAATCGGTCC
TCTACGTCTCTTTTGGGAGCACAGTTAGATTCCCAGAGGAGCAACTCGCTGAAATCGCAAAAGCTCTAGAAGCTTCTACCGTCCCTTTCATTTG
GGCAGTGAACAAGGACCAATCCACGTGGTTACCGGAGAGTTTGTTCGATGAGAAAAAATGTCTGATTATTAAAGGGTGGGCACCGCAACTA
ACCATCTTAGATCATTCAGCAGTCGGAGGATTCATGACACACTGTGGTTGGAATTCAGTGCTTGAAGCCATCATCGCTGGGGTGCCGTTGGTG
SGT2_phuDH_seq1-2
CCTGCGGATGAGAGGAATGCTTTTGATGAATTGCTCGATCGAACCAGAGAATCTGAGGATCTAAGCTACGGTATCGTTCATGATACTTTTTAC
GAGCTAGAACCTGCCTACGCTGACTACTATCAAAAGATGAAGAAAACCAAATGTTGGCAAATTGGTCCCATTTCCTATTTTTCTTCCAAATTAT
TCCGAAGAAAAGAACTGATTAATTCTTCTGATGAAAGTAACTCATCTGCCGTTGTTGTAGAGTGGTTGAATAAACACAAGCACAAATCGGTCC
TCTACGTCTCTTTTGGGAGCACAGTTAGATTCCCAGAGGAGCAACTCGCTGAAATCGCAAAAGCTCTAGAAGCTTCTACCGTCCCTTTCATTTG
GGCAGTGAACAAGGACCAATCCACGTGGTTACCGGAGAGTTTGTTCGATGAGAAAAAATGTCTGATTATTAAAGGGTGGGCACCGCAACTA
ACCATCTTAGATCATTCAGCAGTCGGAGGATTCATGACACACTGTGGTTGGAATTCAGTGCTTGAAGCCATCATCGCTGGGGTGCCGTTGGTG
SGT2_phu_DM_all-1
CCTGCGGATGAGAGGAATGCTTTTGATGAATTGCTCGATCGAACCAGAGAATCTGAGGATCTAAGCTACGGTATCGTTCATGATACTTTTTAC
GAGCTAGAACCTGCCTACGCTGACTACTATCAAAAGATGAAGAAAACCAAATGTTGGCAAATTGGTCCCATTTCCTATTTTTCTTCCAAATTAT
TCCGAAGAAAAGAACTGATTAATTCTTCTGATGAAAGTAACTCATCTGCCGTTGTTGTAGAGTGGTTGAATAAACACAAGCACAAATCGGTCC
TCTACGTCTCTTTTGGGAGCACAGTTAGATTCCCAGAGGAGCAACTCGCTGAAATCGCAAAAGCTCTAGAAGCTTCTACCGTCCCTTTCATTTG
GGCAGTGAACAAGGACCAATCCACGTGGTTACCGGAGAGTTTGTTCGATGAGAAAAAATGTCTGATTATTAAAGGGTGGGCACCGCAACTA
ACCATCTTAGATCATTCAGCAGTCGGAGGATTCATGACACACTGTGGTTGGAATTCAGTGCTTGAAGCCATCATCGCTGGGGTGCCGTTGGTG
SGT2_phu_DM_all-1-2
CCTGCGGATGAGAGGAATGCTTTTGATGAATTGCTCGATCGAACCAGAGAATCTGAGGATCTAAGCTACGGTATCGTTCATGATACTTTTTAC
GAGCTAGAACCTGCCTACGCTGACTACTATCAAAAGATGAAGAAAACCAAATGTTGGCAAATTGGTCCCATTTCCTATTTTTCTTCCAAATTAT
TCCGAAGAAAAGAACTGATTAATTCTTCTGATGAAAGTAACTCATCTGCCGTTGTTGTAGAGTGGTTGAATAAACACAAGCACAAATCGGTCC
TCTACGTCTCTTTTGGGAGCACAGTTAGATTCCCAGAGGAGCAACTCGCTGAAATCGCAAAAGCTCTAGAAGCTTCTACCGTCCCTTTCATTTG
GGCAGTGAACAAGGACCAATCCACGTGGTTACCGGAGAGTTTGTTCGATGAGAAAAAATGTCTGATTATTAAAGGGTGGGCACCGCAACTA
ACCATCTTAGATCATTCAGCAGTCGGAGGATTCATGACACACTGTGGTTGGAATTCAGTGCTTGAAGCCATCATCGCTGGGGTGCCGTTGGTG
SGT2_tbr_seq1
CCTGCGGATGAGAGGAATGCTTTTGATGAATTGCTCGATCGAACCAGAGAATCTGAGGATCTAAGCTACGGAATCGTTCATGATACTTTTTAC
GAGCTAGAACCTGCCTACGCTGACTACTATCAGAAGATGAAGAAAACCAAATGTTGGCAAATTGGTCCCATTTCCTATTTTTCTTCCAAATTAT
CCCCAAGAAAAGAACTGATTAATTCTTCTGATGAAAGTAACTCATCTGCCGTTGTTGTAGAGTGGTTGAATAAACATAAGCACAAATCGGTCC
TCTACGTCTCTTTTGGGAGCACAATTAGATTCCCAGAGGAGCAACTCGCTGAAATCGCAAAAGCTCTAGAAGCTTCTACCGTCCCTTTCATTTG
GGTAGTAAACAAAGACCAATTAGCAAAAACCACGTGGTTACCGGAGAGTTTGTTCGATGAGAAAAAATGTCTGATTATTAAAGGGTGGGCAC
CGCAACTATCCATCTTAGATCATTCAGCAGTCGGAGGATTCATGACACACTGTGGTTGGAATTCAGTGCTTGAAGCCATCATCGCCGGGGTGC
CGTTGGTG
SGT2_tbr_seq2
CCTGCAGATGAGAGGAATGGTTTTGATGAATTGCTCGATCGAACCAGAGAATCTGAGGATCAAAGCTACGGTATCGTTCATGATACTTTTTAC
GAACTAGAACCTGCCTACGCTGACTACTATCAGAAGATGAAGAAAACCAAATGTTGGCAAATTGGTCCCATTTCCTATTTTTCTTCCAAATTAT
TCCGAAGAAAAGATCTGATTAATTCTTTTGATGAAAGTAACTCATCTGCCGCTGTTGTAGAGTGGTTGAATAAACAGAAGCACAAATCGGTCC
TCTACGTCTCTTTCGGGAGCACAGTTAAATTCCCAGAGGAGCAACTCGCTGAAATCGCAAAAGCTCTAGAAGCTTCTACCGTCCCTTTCATTTG
GGTAGTGAAGGAGGACCAATCAGCAAAAACCACCTGGTTACCGGAGAGTTTGTTCGATGAGAAAAAAGGTCTGATTATTAAAGGGTGGGCT
CCGCAACTAACCATCTTAGATCATTCAGCAGTAGGAGGATTCATGACACACTGTGGATGGAATTCGGTGCTTGAAGCTATCATCGCTGGGGT
GCCGTTGGTG

Table S1 Raw data from whole genome SNP genotyping with SolCAP 8303 Illumina Infinium potato SNP chip for 12 accessions

Available for download as an Excel file at http://www.g3journal.org/lookup/suppl/doi:10.1534/g3.113.007146/-/DC1.
