# Supplementary material for: Sequence Diversity in Coding Regions of Candidate Genes in the Glycoalkaloid Biosynthetic Pathway of Wild Potato Species
Source: G3 (Bethesda). 2013 Sep 1;3(9):1467–79. doi: 10.1534/g3.113.007146 (PMC3755908; doi:10.1534/g3.113.007146)
Supplement: Supporting Information [file supp_g3.113.007146_FileS1.pdf]

Supporting Data 1 Allelic sequences identified in six wild and one cultivated potato species for five candidate genes within the glycoalkaloid biosynthetic pathway

1.1 3-Hydroxy-3-methylglutaryl coenzyme A reductase 1 (*HMG1*) sequences
22
HMG1_cmm7_seq1
CGACCTGTTAAGCCTCTATACACATCTAAAGATGCTTCCGCCGGCGAACCTCTGAAACAACAAGAAGTTTCTTCTCCTAAAGCATCTGATGCGC
TTCCACTCCCATTGTACCTAACCAATGGGTTGTTTTTCACCATGTTTTTCTCTGTTATGTATTTTCTTCTCGTAAGGTGGCGTGAGAAGATCCGTA
ATTCTATTCCTCTTCATGTGGTTACCCTTTCTGAATTGTTAGCTATGGTGTCATTGATTGCTTCCGTTATATATCTTTTGGGTTTCTTTGGGATTG
GGTTTGTTCAGTCGTTTGTGTCCAGGTCGAATAGTGATTCATGGGATATTGAGGATGAGAATGCTGAGCAGCTTATTATTGAGGAAGATAGC
CGCCGTGGACCATGTGCTGCTGCCACTACTCTTGGCTGCGTTGTGCCTCCACCACCTGTTCGAAAAATTGCCCCAATGGTTCCACAGCAACCTG
CTAAGGTAGCTTTGTCCCAAACGGAGAAGCCTGCGCCAATAATTATGCCAGCATTATCGGAAGATGACGAGGAGATTATACAATCTGTTGTTC
AGGGTAAAACACCATCATATTCGTTGGAATCAAAGCTTGGTGATTGTATGAGAGCTGCTTCGATTCGAAAAGAGGCGTTACAGAGGATTACA
GGGAAGTCATTAGAAGGGCTCCCATTGGAGGGATTTGACTATGAGTCTATTCTTGGACAGTGCTGTGAGATGCCTGTAGGATATGTGCAAAT
ACCGGTGGGTATTGCTGGGCCTTTGTTGCTTGATGGGAGAGAGTACTCAGTGCCAATGGCAACTACAGAAGGATGTTTAGTGGCTAGCACCA
ACAGGGGTTGCAAGGCTATCTTTGTCTCTGGTGGC
HMG1_cmm7_seq1(2)
CGACCTGTTAAGCCTCTATACACATCTAAAGATGCTTCCGCCGGCGAACCTCTGAAACAACAAGAAGTTTCTTCTCCTAAAGCATCTGATGCGC
TTCCACTCCCATTGTACCTAACCAATGGGTTGTTTTTCACCATGTTTTTCTCTGTTATGTATTTTCTTCTCGTAAGGTGGCGTGAGAAGATCCGTA
ATTCTATTCCTCTTCATGTGGTTACCCTTTCTGAATTGTTAGCTATGGTGTCATTGATTGCTTCCGTTATATATCTTTTGGGTTTCTTTGGGATTG
GGTTTGTTCAGTCGTTTGTGTCCAGGTCGAATAGTGATTCATGGGATATTGAGGATGAGAATGCTGAGCAGCTTATTATTGAGGAAGATAGC
CGCCGTGGACCATGTGCTGCTGCCACTACTCTTGGCTGCGTTGTGCCTCCACCACCTGTTCGAAAAATTGCCCCAATGGTTCCACAGCAACCTG
CTAAGGTAGCTTTGTCCCAAACGGAGAAGCCTGCGCCAATAATTATGCCAGCATTATCGGAAGATGACGAGGAGATTATACAATCTGTTGTTC
AGGGTAAAACACCATCATATTCGTTGGAATCAAAGCTTGGTGATTGTATGAGAGCTGCTTCGATTCGAAAAGAGGCGTTACAGAGGATTACA
GGGAAGTCATTAGAAGGGCTCCCATTGGAGGGATTTGACTATGAGTCTATTCTTGGACAGTGCTGTGAGATGCCTGTAGGATATGTGCAAAT
ACCGGTGGGTATTGCTGGGCCTTTGTTGCTTGATGGGAGAGAGTACTCAGTGCCAATGGCAACTACAGAAGGATGTTTAGTGGCTAGCACCA
ACAGGGGTTGCAAGGCTATCTTTGTCTCTGGTGGC
HMG1_cmm26_seq2
CGACCTGTTAAGCCTCTATACACATCTAAAGATGCTTCCGCCGGCGAACCTCTGAAACAACAAGAAGTTTCTTCTCCTAAAGCATCTGATGCGC
TTCCACTCCCATTGTACCTAACCAATGGGTTGTTTTTCACCATGTTTTTCTCTGTTATGTATTTTCTTCTCGTAAGGTGGCGTGAGAAGATCCGTA
ATTCTATTCCTCTTCATGTGGTTACCCTTTCTGAATTGTTAGCTATGGTGTCATTGATTGCTTCCGTTATATATCTTTTGGGTTTCTTTGGGATTG
GGTTTGTTCAGTCGTTTGTGTCCAGGTCGAATAGTGATTCATGGGATATTGAGGATGAGAATGCTGAGCAGCTTATTATTGAGGAAGATAGC
CGCCGTGGACCATGTGCTGCTGCCACTACTCTTGGCTGCGTTGTGCCTCCACCACCTGTTCGAAAAATTGCCCCAATGGTTCCACAGCAACCTG
CTAAGGTAGCTTTGTCCCAAACGGAGAAGCCTGCGCCAATAATTATGCCAGCATTATCGGAAGATGACGAGGAGATTATACAATCTGTTGTTC
AGGGTAAAACACCATCATATTCGTTGGAATCAAAGCTTGGTGATTGTATGAGAGCTGCTTCGATTCGAAAAGAGGCGTTACAGAGGATTACA
GGGAAGTCATTGGAAGGGCTCCCATTGGAGGGATTTGACTATGAGTCTATTCTTGGACAGTGCTGTGAGATGCCTGTAGGATATGTGCAAAT
ACCGGTGGGTATTGCTGGGCCTTTGCTGCTTGATGGGAGAGAGTACTCAGTGCCAATGGCAACTACAGAAGGATGTTTAGTGGCTAGCACCA
ACAGGGGTTGCAAGGCTATCTTTGTCTCTGGTGGC
HMG1_cmm26_seq2(2)
CGACCTGTTAAGCCTCTATACACATCTAAAGATGCTTCCGCCGGCGAACCTCTGAAACAACAAGAAGTTTCTTCTCCTAAAGCATCTGATGCGC
TTCCACTCCCATTGTACCTAACCAATGGGTTGTTTTTCACCATGTTTTTCTCTGTTATGTATTTTCTTCTCGTAAGGTGGCGTGAGAAGATCCGTA
ATTCTATTCCTCTTCATGTGGTTACCCTTTCTGAATTGTTAGCTATGGTGTCATTGATTGCTTCCGTTATATATCTTTTGGGTTTCTTTGGGATTG
GGTTTGTTCAGTCGTTTGTGTCCAGGTCGAATAGTGATTCATGGGATATTGAGGATGAGAATGCTGAGCAGCTTATTATTGAGGAAGATAGC
CGCCGTGGACCATGTGCTGCTGCCACTACTCTTGGCTGCGTTGTGCCTCCACCACCTGTTCGAAAAATTGCCCCAATGGTTCCACAGCAACCTG
CTAAGGTAGCTTTGTCCCAAACGGAGAAGCCTGCGCCAATAATTATGCCAGCATTATCGGAAGATGACGAGGAGATTATACAATCTGTTGTTC
AGGGTAAAACACCATCATATTCGTTGGAATCAAAGCTTGGTGATTGTATGAGAGCTGCTTCGATTCGAAAAGAGGCGTTACAGAGGATTACA
GGGAAGTCATTGGAAGGGCTCCCATTGGAGGGATTTGACTATGAGTCTATTCTTGGACAGTGCTGTGAGATGCCTGTAGGATATGTGCAAAT
ACCGGTGGGTATTGCTGGGCCTTTGCTGCTTGATGGGAGAGAGTACTCAGTGCCAATGGCAACTACAGAAGGATGTTTAGTGGCTAGCACCA
ACAGGGGTTGCAAGGCTATCTTTGTCTCTGGTGGC
HMG1_dms54_seq1
CGACCTGTTAAGCCTCTATACACATCTAAAGATGCTTCCGCCGGCGAACCTCTGAAGCAACAAGAAGTTTCTTCTCCTAAAGCATCTGATGCGC
TTCCACTCCCATTGTACCTAACCAATGGGTTGTTTTTCACCATGTTTTTCTCTGTTATGTATTTTCTTCTCGTAAGGTGGCGTGAGAAGATCCGTA
ATTCTATTCCTCTTCATGTGGTTACCCTTTCTGAATTGTTAGCTATGGTGTCATTGATTGCTTCCGTTATATATCTTTTGGGTTTCTTTGGGATTG
GGTTTGTTCAGTCGTTTGTGTCCAGGTCGAATAGTGATTCATGGGATATTGAGGATGAGAATGCTGAGCAGCTTATTATTGAGGAAGATAGC
CGCCGTGGACCATGTGCTGCTGCCACTACTCTTGGCTGCGTTGTGCCTCCACCACCTGTTCGAAAAATTGCCCCAATGGTTCCACAGCAACCTG
CTAAGGTAGCTTTGTCCCAAACGGAGAAGCCTGCGCCAATAATTATGCCAGCATTATCGGAAGATGACGAGGAGATTATACAATCTGTTGTTC
AGGGTAAAACACCATCATATTCGTTGGAATCAAAGCTTGGTGATTGTATGAGAGCTGCTTCGATTCGAAAAGAGGCGTTACAGAGGATTACA
GGGAAGTCATTGGAAGGGCTCCCATTGGAGGGATTTGACTATGAGTCTATTCTTGGACAGTGCTGTGAGATGCCTGTAGGATATGTGCAAAT

ACCGGTGGGTATTGCTGGGCCTTTGTTGCTTGATGGGAGAGAGTACTCAGTGCCAATGGCAACTACAGAAGGATGTTTAGTGGCTAGCACCA
ACAGGGGTTGCAAGGCTATCTTTGTCTCTGGTGGC
HMG1_dms54_seq2
CGACCTGTTAAGCCTCTATACACATCTAAAGATGCTTCCGCCGGCGAACCTCTGAAACAACAAGAAGTTTCTTCTCCTAAAGCATCTGATGCGC
TTCCACTCCCATTGTACCTAACCAATGGGCTGTTTTTCACCATGTTTTTCTCTGTTATGTATTTTCTTCTCGTAAGGTGGCGTGAGAAGATCCGT
AATTCTATTCCTCTTCATGTGGTTACCCTTTCTGAATTGTTAGCTATGGTGTCATTGATTGCTTCCGTTATATATCTTTTGGGTTTCTTTGGGATT
GGGTTTGTTCAGTCGTTTGTGTCCAGGTCGAATAGTGATTCATGGGATATTGAGGATGAGAATGCTGAGCAGCTTATTATTGAGGAAGATAG
CCGCCGTGGACCATGTGCTGCTGCCACTACTCTTGGCTGCGTTGTGCCTCCACCACCTGTTCGAAAAATTGCCCCAATGGTTCCACTGCAACCT
GCTAAGGTAGCTTTGTCCCAAACGGAGAAGCCTGCGCCAATAATTATGCCAGCATTATCGGAAGATGACGAGGAGATTATACAATCTGTTGT
TCAGGGTAAAACACCATCATATTCGTTGGAATCAAAGCTTGGTGATTGTATGAGAGCTGCTTCGATTCGAAAAGAGGCGTTACAGAGGATTA
CAGGGAAGTCATTGGAAGGGCTCCCATTGGAGGGATTTGACTATGAGTCTATTCTTGGACAGTGCTGTGAGATGCCTGTAGGATATGTGCAA
ATACCGGTGGGTATTGCTGGGCCTTTGTTGCTTGATGGGAGAGAGTACTCAGTGCCAATGGCAACTACAGAAGGATGTTTAGTGGCTAGCAC
CAACAGGGGTTGCAAGGCTATCTTTGTCTCTGGTGGC
HMG1_dms78_seq1
CGACCTGTTAAGCCTCTATACACATCTAAAGATGCTTCCGCCGGCGAACCTCTGAAGCAACAAGAAGTTTCTTCTCCTAAAGCATCTGATGCGC
TTCCACTCCCATTGTACCTAACCAATGGGTTGTTTTTCACCATGTTTTTCTCTGTTATGTATTTTCTTCTCGTAAGGTGGCGTGAGAAGATCCGTA
ATTCTATTCCTCTTCATGTGGTTACCCTTTCTGAATTGTTAGCTATGGTGTCATTGATTGCTTCCGTTATATATCTTTTGGGTTTCTTTGGGATTG
GGTTTGTTCAGTCGTTTGTGTCCAGGTCGAATAGTGATTCATGGGATATTGAGGATGAGAATGCTGAGCAGCTTATTATTGAGGAAGATAGC
CGCCGTGGACCATGTGCTGCTGCCACTACTCTTGGCTGCGTTGTGCCTCCACCACCTGTTCGAAAAATTGCCCCAATGGTTCCACAGCAACCTG
CTAAGGTAGCTTTGTCCCAAACGGAGAAGCCTGCGCCAATAATTATGCCAGCATTATCGGAAGATGACGAGGAGATTATACAATCTGTTGTTC
AGGGTAAAACACCATCATATTCGTTGGAATCAAAGCTTGGTGATTGTATGAGAGCTGCTTCGATTCGAAAAGAGGCGTTACAGAGGATTACA
GGGAAGTCATTGGAAGGGCTCCCATTGGAGGGATTTGACTATGAGTCTATTCTTGGACAGTGCTGTGAGATGCCTGTAGGATATGTGCAAAT
ACCGGTGGGTATTGCTGGGCCTTTGTTGCTTGATGGGAGAGAGTACTCAGTGCCAATGGCAACTACAGAAGGATGTTTAGTGGCTAGCACCA
ACAGGGGTTGCAAGGCTATCTTTGTCTCTGGTGGC
HMG1_dms78_seq2
CGACCTGTTAAGCCTCTATACACATCTAAAGATGCTTCCGCCGGCGAACCTCTGAAACAACAAGAAGTTTCTTCTCCTAAAGCATCTGATGCGC
TTCCACTCCCATTGTACCTAACCAATGGGCTGTTTTTCACCATGTTTTTCTCTGTTATGTATTTTCTTCTCGTAAGGTGGCGTGAGAAGATCCGT
AATTCTATTCCTCTTCATGTGGTTACCCTTTCTGAATTGTTAGCTATGGTGTCATTGATTGCTTCCGTTATATATCTTTTGGGTTTCTTTGGGATT
GGGTTTGTTCAGTCGTTTGTGTCCAGGTCGAATAGTGATTCATGGGATATTGAGGATGAGAATGCTGAGCAGCTTATTATTGAGGAAGATAG
CCGCCGTGGACCATGTGCTGCTGCCACTACTCTTGGCTGCGTTGTGCCTCCACCACCTGTTCGAAAAATTGCCCCAATGGTTCCACTGCAACCT
GCTAAGGTAGCTTTGTCCCAAACGGAGAAGCCTGCGCCAATAATTATGCCAGCATTATCGGAAGATGACGAGGAGATTATACAATCTGTTGT
TCAGGGTAAAACACCATCATATTCGTTGGAATCAAAGCTTGGTGATTGTATGAGAGCTGCTTCGATTCGAAAAGAGGCGTTACAGAGGATTA
CAGGGAAGTCATTGGAAGGGCTCCCATTGGAGGGATTTGACTATGAGTCTATTCTTGGACAGTGCTGTGAGATGCCTGTAGGATATGTGCAA
ATACCGGTGGGTATTGCTGGGCCTTTGTTGCTTGATGGGAGAGAGTACTCAGTGCCAATGGCAACTACAGAAGGATGTTTAGTGGCTAGCAC
CAACAGGGGTTGCAAGGCTATCTTTGTCTCTGGTGGC
HMG1_spg55_seq1
CGACCTGTTAAGCCTCTATACACATCTAAAGATGCTTCCGCCGGCGAACCTCTGAAACAACAAGAAGTTTCTTCTCCTAAAGCATCTGATGCGC
TTCCACTCCCATTGTACCTAACCAATGGGTTGTTTTTCACCATGTTTTTCTCTGTTATGTATTTTCTTCTCGTAAGGTGGCGTGAGAAGATCCGTA
ATTCTATTCCTCTTCATGTGGTTACCCTTTCTGAATTGTTAGCTATGGTGTCATTGATTGCTTCCGTTATATATCTTTTGGGTTTCTTTGGGATTG
GGTTTGTTCAGTCGTTTGTGTCCAGGTCGAATAGTGATTCATGGGATATTGAGGATGAGAATGCTGAGCAGCTTATTATTGAGGAAGATAGC
CGCCGTGGACCATGTGCTGCTGCCACTACTCTTGGCTGCGTTGTGCCTCCACCACCTGTTCGAAAAATTGCCCCAATGGTTCCACAGCAACCTG
CTAAGGTAGCTTTGTCCCAAACGGAGAAGCCTGCGCCAATAATTATGCCAGCATTATCGGAAGATGACGAGGAGATTATACAATCTGTTGTTC
AGGGTAAAACACCATCATATTCGTTGGAATCAAAGCTTGGTGATTGTATGAGAGCTGCTTCGATTCGAAAAGAGGCGTTACAGAGGATTACA
GGGAAGTCATTGGAAGGGCTCCCATTGGAGGGATTTGACTATGAGTCTATTCTTGGACAGTGCTGTGAGATGCCTGTAGGATATGTGCAAAT
ACCGGTGGGTATTGCTGGGCCTTTGTTGCTTGATGGGAGAGAGTACTCAGTGCCAATGGCAACTACAGAAGGATGTTTAGTGGCTAGCACCA
ACAGGGGTTGCAAGGCTATCTTTGTCTCTGGTGGC
HMG1_spg55_seq2
CGACCTGTTAAGCCTCTATACACATCTAAAGATGCTTTCGCCGGCGAACCTCTGAAACAAGAAGTTTTTTCTCCTAAAGCATCTGATGCGCTTC
CACTCCCATTGTACCTAACCACTGGGTTGTTTTTCACCATGTTTTTCTCTGTTATGTATTTTCTTCTCGTAAGGTGGCGTGAGAAGATCCGTAATT
CTATTCCTCTTCATGTGGTTACCCTTTCTGAATTGTTAGCTATGGTGTCATTGATTGCTTCCGTTATATATCTTTTGGGTTTCTTTGGGATTGGGT
TTGTTCAGTCATTTGTGTCCAGGTCGAATAGTGATTCATGGGATATTGAGGATGAGAATGCTGAGCAGCTTATTATTGAGGAAGATAGCCGTC
GCGGACCATGTGCTGCTGCCACTACTCTTGGCTGCGTTGTGCCTCCACCACCTGTTCGAAAAATTGCCCCAATGGTTCCACAGCAACCTGCTAA
GGTAGCTTTGTCCCAAATGGAGAAGCCTGCGCCAATAATTATGCCCGCATTATCGGAAGATGACGAGGAGATTATACAATCTGTTGTTCAGG
GTAAAACACCATCATATTCGTTGGAATCAAAGCTTGGTGATTGTATGAGAGCTGCTTCGATTCAAAAAGAGGCGTTACAGAGGATTACAGGG
AAGTCATTGGAAGGGCTCCCATTGGAGGGATTTGACTATGAGTCTATTCTTGGACAGTGCTGTGAGATGCCTGTAGGATATGTGCAAATACC
GGTGGGTATTGTTGGGCCTTTGTTGCTTGATGGGAGAGAGTACTCAGTGCCAATGGCAACTACAGAAGGATGCTTAGTGGCTAGCACCAACA
GGGGTTGCAAGGCTATCTTTGTCTCTGGTGGC
HMG1_spg74_seq1

CGACCTGTTAAGCCTCTATACACATCTAAAGATGCTTCCGCCGGCGAACCTCTGAAACAACAAGAAGTTTCTTCTCCTAAAGCATCTGATGCGC
TTCCACTCCCATTGTACCTAACCAATGGGTTGTTTTTCACCATGTTTTTCTCTGTTATGTATTTTCTTCTCGTAAGGTGGCGTGAGAAGATCCGTA
ATTCTATTCCTCTTCATGTGGTTACCCTTTCTGAATTGTTAGCTATGGTGTCATTGATTGCTTCCGTTATATATCTTTTGGGTTTCTTTGGGATTG
GGTTTGTTCAGTCGTTTGTGTCCAGGTCGAATAGTGATTCATGGGATATTGAGGATGAGAATGCTGAGCAGCTTATTATTGAGGAAGATAGC
CGCCGTGGACCATGTGCTGCTGCCACTACTCTTGGCTGCGTTGTGCCTCCACCACCTGTTCGAAAAATTGCCCCAATGGTTCCACAGCAACCTG
CTAAGGTAGCTTTGTCCCAAACGGAGAAGCCTGCGCCAATAATTATGCCAGCATTATCGGAAGATGACGAGGAGATTATACAATCTGTTGTTC
AGGGTAAAACACCATCATATTCGTTGGAATCAAAGCTTGGTGATTGTATGAGAGCTGCTTCGATTCGAAAAGAGGCGTTACAGAGGATTACA
GGGAAGTCATTGGAAGGGCTCCCATTGGAGGGATTTGACTATGAGTCTATTCTTGGACAGTGCTGTGAGATGCCTGTAGGATATGTGCAAAT
ACCGGTGGGTATTGCTGGGCCTTTGTTGCTTGATGGGAGAGAGTACTCAGTGCCAATGGCAACTACAGAAGGATGTTTAGTGGCTAGCACCA
ACAGGGGTTGCAAGGCTATCTTTGTCTCTGGTGGC

HMG1_spg74_seq2

CGACCTGTTAAGCCTCTATACACATCTAAAGATGCTTTCGCCGGCGAACCTCTGAAACAAGAAGTTTTTTCTCCTAAAGCATCTGATGCGCTTC
CACTCCCATTGTACCTAACCACTGGGTTGTTTTTCACCATGTTTTTCTCTGTTATGTATTTTCTTCTCGTAAGGTGGCGTGAGAAGATCCGTAATT
CTATTCCTCTTCATGTGGTTACCCTTTCTGAATTGTTAGCTATGGTGTCATTGATTGCTTCCGTTATATATCTTTTGGGTTTCTTTGGGATTGGGT
TTGTTCAGTCATTTGTGTCCAGGTCGAATAGTGATTCATGGGATATTGAGGATGAGAATGCTGAGCAGCTTATTATTGAGGAAGATAGCCGTC
GCGGACCATGTGCTGCTGCCACTACTCTTGGCTGCGTTGTGCCTCCACCACCTGTTCGAAAAATTGCCCCAATGGTTCCACAGCAACCTGCTAA
GGTAGCTTTGTCCCAAATGGAGAAGCCTGCGCCAATAATTATGCCCGCATTATCGGAAGATGACGAGGAGATTATACAATCTGTTGTTCAGG
GTAAAACACCATCATATTCGTTGGAATCAAAGCTTGGTGATTGTATGAGAGCTGCTTCGATTCAAAAAGAGGCGTTACAGAGGATTACAGGG
AAGTCATTGGAAGGGCTCCCATTGGAGGGATTTGACTATGAGTCTATTCTTGGACAGTGCTGTGAGATGCCTGTAGGATATGTGCAAATACC
GGTGGGTATTGTTGGGCCTTTGTTGCTTGATGGGAGAGAGTACTCAGTGCCAATGGCAACTACAGAAGGATGCTTAGTGGCTAGCACCAACA
GGGGTTGCAAGGCTATCTTTGTCTCTGGTGGC

HMG1_spl16_seq2

CGACCTGTTAAGCCTCTATACACATCTAAAGATGCTTCCGCCGGCGAACCTCTGAAACAACAAGAAGTTTCTTCTCCTAAAGCATCTGATGCGC
TTCCACTCCCATTGTACCTAACCAATGGGTTGTTTTTCACCATGTTTTTCTCTGTTATGTATTTTCTTCTCGTAAGGTGGCGTGAGAAGATCCGTA
ATTCTATTCCTCTTCATGTGGTTACCCTTTCTGAATTGTTAGCTATGGTGTCATTGATTGCTTCCGTTATATATCTTTTGGGTTTCTTTGGGATTG
GGTTTGTTCAGTCGTTTGTGTCCAGGTCGAATAGTGATTCATGGGATATTGAGGATGAGAATGCTGAGCAGCTTATTATTGAGGAAGATAGC
CGCCGTGGACCATGTGCTGCTGCCACTACTCTTGGCTGCGTTGTGCCTCCACCACCTGTTCGAAAAATTGCCCCAATGGTTCCACAGCAACCTG
CTAAGGTAGCTTTGTCCCAAACGGAGAAGCCTGCGCCAATAATTATGCCAGCATTATCGGAAGATGACGAGGAGATTATACAATCTGTTGTTC
AGGGTAAAACACCATCATATTCGTTGGAATCAAAGCTTGGTGATTGTATGAGAGCTGCTTCGATTCGAAAAGAAGCGTTACAGAGGATTACA
GGGAAGTCATTGGAAGGGCTCCCATTGGAGGGATTTGACTATGAGTCTATTCTTGGACAGTGCTGTGAGATGCCTGTAGGATATGTGCAAAT
ACCGGTGGGTATTGCTGGGCCTTTGTTGCTTGATGGGAGAGAGTACTCAGTGCCAATGGCAACTACAGAAGGATGTTTAGTGGCTAGCACCA
ACAGGGGTTGCAAGGCTATCTTTGTCTCTGGTGGC

HMG1_spl81_seq3

CGACCTGTTAAGCCTCTATACACATCTAAAGATGCTTCCGCCGGCGAACCTCTGAAACAACAAGAAGTTTCTTCTCCTAAAGCATCTGATGCGC
TTCCACTCCCATTGTACCTAACCAATGGGCTGTTTTTCACCATGTTTTTCTCTGTTATGTATTTTCTTCTCGTAAGGTGGCGTGAGAAGATCCGT
AATTCTATTCCTCTTCATGTGGTTACCCTTTCTGAATTGTTAGCTATGGTGTCATTGATTGCTTCCGTTATATATCTTTTGGGTTTCTTTGGGATT
GGGTTTGTTCAGTCGTTTGTGTCCAGGTCGAATAGTGATTCATGGGATATTGAGGATGAGAATGCTGAGCAGCTTATTATTGAGGAAGATAG
CCGCCGTGGACCATGTGCTGCTGCCACTACTCTTGGCTGCGTTGTGCCTCCACCACCTGTTCGAAAAATTGCCCCAATGGTTCCACAGCAACCT
GCTAAGGTAGCTTTGTCCCAAACGGAGAAGCCTGCGCCAATAATTATGCCAGCATTATCGGAAGATGACGAGGAGATTATACAATCTGTTGT
TCAGGGTAAAACACCATCATATTCGTTGGAATCAAAGCTTGGTGATTGTATGAGAGCTGCTTCGATTCGAAAAGAGGCGTTACAGAGGATTA
CAGGGAAGTCATTGGAAGGGCTCCCATTGGAGGGATTTGACTATGAGTCTATTCTTGGACAGTGCTGTGAGATGCCTGTAGGATATGTGCAA
ATACCGGTGGGTATTGCTGGGCCTTTGTTGCTTGATGGGAGAGAGTACTCAGTGCCAATGGCAACTACAGAAGGATGTTTAGTGGCTAGCAC
CAACAGGGGTTGCAAGGCTATCTTTGTCTCTGGTGGC

HMG1_sto40_seq1

CGACCTGTTAAGCCTCTATACACATCTAAAGATGCTTCCGCCGGCGAACCTCTGAAACAACAAGAAGTTTCTTCTCCTAAAGCATCTGATGCGC
TTCCACTCCCATTGTACCTAACCAATGGGTTGTTTTTCACCATGTTTTTCTCTGTTATGTATTTTCTTCTCGTAAGGTGGCGTGAGAAGATCCGTA
ATTCTATTCCTCTTCATGTGGTTACCCTTTCTGAATTGTTAGCTATGGTGTCATTGATTGCTTCCGTTATATATCTTTTGGGTTTCTTTGGGATTG
GGTTTGTTCAGTCGTTTGTGTCCAGGTCGAATAGTGATTCATGGGATATTGAGGATGAGAATGCTGAGCAGCTTATTATTGAGGAAGATAGC
CGTCGTGGACCATGTGCTGCTGCCACTACTCTTGGCTGCGTTGTGCCTCCACCACCTGTTCGAAAAATTGCCCCAATGGTTCCACAGCAACCTG
CTAAGGTAGCTTTGTCCCAAACGGAGAAACCTGCGCCAATAATTATGCCAGCATTATCGGAAGATGACGAGGAGATTATACAATCTGTTGTTC
AGGGTAAAACACCATCATATTCGTTGGAATCAAAGCTTGGTGATTGTATGAGAGCTGCTTCGATTCGAAAAGAGGCGTTACAGAGGATTACA
GGGAAGTCATTGGAAGGGCTCCCATTGGAGGGATTTGACTATGAGTCTATTCTTGGACAGTGTTGTGAGATGCCTGTAGGATATGTGCAAAT
ACCGGTGGGTATTGCTGGGCCTTTGTTGCTTGATGGGAGAGAGTACTCAGTGCCAATGGCAACTACAGAAGGATGTTTAGTGGCTAGCACCA
ACAGGGGTTGCAAGGCTATCTTTGTCTCTGGTGGC

HMG1_sto40_seq2

CGACCTGTTAAGCCTCTATACACATCTAAAGATGCTTCCGCCGGCGAACCTCTGAAACAACAAGAAGTTTCTTCTCCTAAAGCATCTGATGCGC
TTCCACTCCCATTGTACCTAACCAATGGGTTGTTTTTCACCATGTTTTTCTCTGTTATGTATTTTCTTCTCGTAAGGTGGCGTGAGAAGATCCGTA
ATTCTATTCCTCTTCATGTGGTTACCCTTTCTGAATTGTTAGCTATGGTGTCATTGATTGCTTCCGTTATATATCTTTTGGGTTTCTTTGGGATTG

GGTTTGTTCAGTCGTTTGTGTCCAGGTCGAATAGTGATTCATGGGATATTGAGGATGAGAATGCTGAGCAGCTTATTATTGAGGAAGATAGC
CGCCGTGGACCATGTGCTGCTGCCACTACTCTTGGCTGCGTTGTGCCTCCACCACCTGTTCGAAAAATTGCCCCAATGGTTCCACAGCAACCTG
CTAAGGTAGCTTTGTCCCAAACGGAGAAGCCTGCGCCAATAATTATGCCAGCATTATCGGAAGATGACGAGGAGATTATACAATCTGTTGTTC
AGGGTAAAACACCATCATATTCGTTGGAATCAAAGCTTGGTGATTGTATGAGAGCTGCTTCGATTCGAAAAGAAGCGTTACAGAGGATTACA
GGGAAGTCATTGGAAGGGCTCCCATTGGAGGGATTTGACTATGAGTCTATTCTTGGACAGTGCTGTGAGATGCCTGTAGGATATGTGCAAAT
ACCGGTGGGTATTGCTGGGCCTTTGTTGCTTGATGGGAGAGAGTACTCAGTGCCAATGGCAACTACAGAAGGATGTTTAGTGGCTAGCACCA
ACAGGGGTTGCAAGGCTATCTTTGTCTCTGGTGGC
HMG1_sto61_seq1
CGACCTGTTAAGCCTCTATACACATCTAAAGATGCTTCCGCCGGCGAACCTCTGAAACAACAAGAAGTTTCTTCTCCTAAAGCATCTGATGCGC
TTCCACTCCCATTGTACCTAACCAATGGGTTGTTTTTCACCATGTTTTTCTCTGTTATGTATTTTCTTCTCGTAAGGTGGCGTGAGAAGATCCGTA
ATTCTATTCCTCTTCATGTGGTTACCCTTTCTGAATTGTTAGCTATGGTGTCATTGATTGCTTCCGTTATATATCTTTTGGGTTTCTTTGGGATTG
GGTTTGTTCAGTCGTTTGTGTCCAGGTCGAATAGTGATTCATGGGATATTGAGGATGAGAATGCTGAGCAGCTTATTATTGAGGAAGATAGC
CGTCGTGGACCATGTGCTGCTGCCACTACTCTTGGCTGCGTTGTGCCTCCACCACCTGTTCGAAAAATTGCCCCAATGGTTCCACAGCAACCTG
CTAAGGTAGCTTTGTCCCAAACGGAGAAACCTGCGCCAATAATTATGCCAGCATTATCGGAAGATGACGAGGAGATTATACAATCTGTTGTTC
AGGGTAAAACACCATCATATTCGTTGGAATCAAAGCTTGGTGATTGTATGAGAGCTGCTTCGATTCGAAAAGAGGCGTTACAGAGGATTACA
GGGAAGTCATTGGAAGGGCTCCCATTGGAGGGATTTGACTATGAGTCTATTCTTGGACAGTGTTGTGAGATGCCTGTAGGATATGTGCAAAT
ACCGGTGGGTATTGCTGGGCCTTTGTTGCTTGATGGGAGAGAGTACTCAGTGCCAATGGCAACTACAGAAGGATGTTTAGTGGCTAGCACCA
ACAGGGGTTGCAAGGCTATCTTTGTCTCTGGTGGC
HMG1_sto61_seq2
CGACCTGTTAAGCCTCTATACACATCTAAAGATGCTTCCGCCGGCGAACCTCTGAAACAACAAGAAGTTTCTTCTCCTAAAGCATCTGATGCGC
TTCCACTCCCATTGTACCTAACCAATGGGTTGTTTTTCACCATGTTTTTCTCTGTTATGTATTTTCTTCTCGTAAGGTGGCGTGAGAAGATCCGTA
ATTCTATTCCTCTTCATGTGGTTACCCTTTCTGAATTGTTAGCTATGGTGTCATTGATTGCTTCCGTTATATATCTTTTGGGTTTCTTTGGGATTG
GGTTTGTTCAGTCGTTTGTGTCCAGGTCGAATAGTGATTCATGGGATATTGAGGATGAGAATGCTGAGCAGCTTATTATTGAGGAAGATAGC
CGCCGTGGACCATGTGCTGCTGCCACTACTCTTGGCTGCGTTGTGCCTCCACCACCTGTTCGAAAAATTGCCCCAATGGTTCCACAGCAACCTG
CTAAGGTAGCTTTGTCCCAAACGGAGAAGCCTGCGCCAATAATTATGCCAGCATTATCGGAAGATGACGAGGAGATTATACAATCTGTTGTTC
AGGGTAAAACACCATCATATTCGTTGGAATCAAAGCTTGGTGATTGTATGAGAGCTGCTTCGATTCGAAAAGAAGCGTTACAGAGGATTACA
GGGAAGTCATTGGAAGGGCTCCCATTGGAGGGATTTGACTATGAGTCTATTCTTGGACAGTGCTGTGAGATGCCTGTAGGATATGTGCAAAT
ACCGGTGGGTATTGCTGGGCCTTTGTTGCTTGATGGGAGAGAGTACTCAGTGCCAATGGCAACTACAGAAGGATGTTTAGTGGCTAGCACCA
ACAGGGGTTGCAAGGCTATCTTTGTCTCTGGTGGC
HMG1_tbr_seq1
CGACCTGTTAAGCCTCTATACACATCTAAAGATGCTTCCGCCGGCGAACCTCTGAAACAACAAGAAGTTTCTTCTCCTAAAGCATCTGATGCGC
TTCCACTCCCATTGTACCTAACCAATGGGTTGTTTTTCACCATGTTTTTCTCTGTTATGTATTTTCTTCTCGTAAGGTGGCGTGAGAAGATCCGTA
ATTCTATTCCTCTTCATGTGGTTACCCTTTCTGAATTGTTAGCTATGGTGTCATTGATTGCTTCCGTTATATATCTTTTGGGTTTCTTTGGGATTG
GGTTTGTTCAGTCGTTTGTGTCCAGGTCGAATAGTGATTCATGGGATATTGAGGATGAGAATGCTGAGCAGCTTATTATTGAGGAAGATAGC
CGCCGTGGACCATGTGCTGCTGCCACTACTCTTGGCTGCGTTGTGCCTCCACCACCTGTTCGAAAAATTGCCCCAATGGTTCCACAGCAACCTG
CTAAGGTAGCTTTGTCCCAAACGGAGAAGCCTTCGCCAATAATTATGCCAGCATTATCGGAAGATGACGAGGAGATTATACAATCTGTTGTTC
AGGGTAAAACACCATCATATTCGTTGGAATCAAAGCTTGGTGATTGTATGAGAGCTGCTTCGATTCGAAAAGAGGCGTTACAGAGGATTACA
GGGAAGTCATTGGAAGGGCTCCCATTGGAGGGATTTGACTATTCGTCTATTCTTGGACAGTGCTGTGAGATGCCTGTAGGATATGTGCAAAT
ACCGGTGGGTATTGCTGGGCCTTTGTTGCTTGATGGGAGAGAGTACTCAGTGCCAATGGCAACTACAGAAGGATGTTTAGTGGCTAGCACCA
ACAGGGGTTGCAAGGCTATCTTTGTCTCTGGTGGC
HMG1_tbr_seq1(2)
CGACCTGTTAAGCCTCTATACACATCTAAAGATGCTTCCGCCGGCGAACCTCTGAAACAACAAGAAGTTTCTTCTCCTAAAGCATCTGATGCGC
TTCCACTCCCATTGTACCTAACCAATGGGTTGTTTTTCACCATGTTTTTCTCTGTTATGTATTTTCTTCTCGTAAGGTGGCGTGAGAAGATCCGTA
ATTCTATTCCTCTTCATGTGGTTACCCTTTCTGAATTGTTAGCTATGGTGTCATTGATTGCTTCCGTTATATATCTTTTGGGTTTCTTTGGGATTG
GGTTTGTTCAGTCGTTTGTGTCCAGGTCGAATAGTGATTCATGGGATATTGAGGATGAGAATGCTGAGCAGCTTATTATTGAGGAAGATAGC
CGCCGTGGACCATGTGCTGCTGCCACTACTCTTGGCTGCGTTGTGCCTCCACCACCTGTTCGAAAAATTGCCCCAATGGTTCCACAGCAACCTG
CTAAGGTAGCTTTGTCCCAAACGGAGAAGCCTTCGCCAATAATTATGCCAGCATTATCGGAAGATGACGAGGAGATTATACAATCTGTTGTTC
AGGGTAAAACACCATCATATTCGTTGGAATCAAAGCTTGGTGATTGTATGAGAGCTGCTTCGATTCGAAAAGAGGCGTTACAGAGGATTACA
GGGAAGTCATTGGAAGGGCTCCCATTGGAGGGATTTGACTATTCGTCTATTCTTGGACAGTGCTGTGAGATGCCTGTAGGATATGTGCAAAT
ACCGGTGGGTATTGCTGGGCCTTTGTTGCTTGATGGGAGAGAGTACTCAGTGCCAATGGCAACTACAGAAGGATGTTTAGTGGCTAGCACCA
ACAGGGGTTGCAAGGCTATCTTTGTCTCTGGTGGC
HMG1_DM_seq3
CGACCTGTTAAGCCTCTATACACATCTAAAGATGCTTCCGCCGGCGAACCTCTGAAACAAGAAGTTTCTTCTCCTAAAGCATCTGATGCGCTTC
CACTCCCATTGTACCTAACCACTGGGTTGTTTTTCACCATGTTTTTCTCTGTTATGTATTTTCTTCTCGTAAGGTGGCGTGAGAAGATCCGTAATT
CTATTCCTCTTCATGTGGTTACCCTTTCTGAATTGTTAGCTATGGTGTCATTGATTGCTTCCGTTATATATCTTTTGGGTTTCTTTGGGATTGGGT
TTGTTCAGTCGTTTGTGTCCAGGTCGAATAGTGATTCATGGGATATTGAGGATGAGAATGCTGAGCAGCTTATTATTGAGGAAGATAGCCGC
CGTGGACCATGTGCTGCTGCCACTACTCTTGGCTGCGTTGTGCCTCCACCACCTGTTCGAAAAATTGCCCCAATGGTTCCACAGCAACCTGCTA
AGGTAGCTTTGTCCCAAACGGAGAAGCCTTCGCCAATAATTATGCCAGCATTATCGGAAGATGACGAGGAGATTATACAATCTGTTGTTCAG

GGTAAAACACCATCATATTCGTTGGAATCAAAGCTTGGTGATTGTATGAGAGCTGCTTCGATTCGAAAAGAGGCGTTACAGAGGATTACAGG
GAAGTCATTGGAAGGGCTCCCATTGGAGGGATTTGACTATGAGTCTATTCTTGGACAGTGCTGTGAGATGCCTGTAGGATATGTGCAAATAC
CGGTGGGTATTGCTGGGCCTTTGTTGCTTGATGGGAGAGAGTACTCAGTGCCAATGGCAACTACAGAAGGATGTTTAGTGGCTAGCACCAAC
AGGGGTTGCAAGGCTATCTTTGTCTCTGGTGGC

HMG1_DM_seq3(2)

CGACCTGTTAAGCCTCTATACACATCTAAAGATGCTTCCGCCGGCGAACCTCTGAAACAAGAAGTTTCTTCTCCTAAAGCATCTGATGCGCTTC
CACTCCCATTGTACCTAACCACTGGGTTGTTTTTCACCATGTTTTTCTCTGTTATGTATTTTCTTCTCGTAAGGTGGCGTGAGAAGATCCGTAATT
CTATTCCTCTTCATGTGGTTACCCTTTCTGAATTGTTAGCTATGGTGTCATTGATTGCTTCCGTTATATATCTTTTGGGTTTCTTTGGGATTGGGT
TTGTTCAGTCGTTTGTGTCCAGGTCGAATAGTGATTCATGGGATATTGAGGATGAGAATGCTGAGCAGCTTATTATTGAGGAAGATAGCCGC
CGTGGACCATGTGCTGCTGCCACTACTCTTGGCTGCGTTGTGCCTCCACCACCTGTTCGAAAAATTGCCCCAATGGTTCCACAGCAACCTGCTA
AGGTAGCTTTGTCCCAAACGGAGAAGCCTTCGCCAATAATTATGCCAGCATTATCGGAAGATGACGAGGAGATTATACAATCTGTTGTTCAG
GGTAAAACACCATCATATTCGTTGGAATCAAAGCTTGGTGATTGTATGAGAGCTGCTTCGATTCGAAAAGAGGCGTTACAGAGGATTACAGG
GAAGTCATTGGAAGGGCTCCCATTGGAGGGATTTGACTATGAGTCTATTCTTGGACAGTGCTGTGAGATGCCTGTAGGATATGTGCAAATAC
CGGTGGGTATTGCTGGGCCTTTGTTGCTTGATGGGAGAGAGTACTCAGTGCCAATGGCAACTACAGAAGGATGTTTAGTGGCTAGCACCAAC
AGGGGTTGCAAGGCTATCTTTGTCTCTGGTGGC

1.2 3-Hydroxy-3-methylglutaryl coenzyme A reductase 2 (*HMG2*) sequences with intron
28
HMG2_cmm-7_seq1
TGGTGTCCAAAGGTGTACAAAATGTTCTTGATTACCTTCAGAATGAATATCCTGACATGGATGTCATCGGCATATCTGGTATTCCACCTACCCT
TTAGTTTTTCCTTAACTTCTCTGCTATGCATGGTGATCTGGTTCTGCTTTTTGTTCCCCCTAAAATAGAGATGTTTGTCTAATTAAAGTTATCAAT
AACTGTGACAGGGAACTTTTGCTCGGACAAGAAGCCATCAGCAGTTAATTGGATCGAGGGGAGAGGAAAGTCTGTAGTTTGTGAGGCAATT
ATTACAGAAGAGGTGGTGAAGAAAGTTCTAAAAACTGAGGTTGCTGCTCTTGTGGAGCTGAACATGCTTAAAAATCTTACTGGCTCTGCCAT
GGCTGGTGCCCTTGGTGGTTTCAATGCTCATGCCAGCAATATCGTCTCAGCTGTGTTTATTGCCACTGGTCAGGATCCAGCTCAGAACATAGA
GAGCTCGCACTGCATCACTATGATGGAGGCTGTAAATGATGGCAAGGACCTCCATATTTCTGT
HMG2_cmm-7_seq1-2
TGGTGTCCAAAGGTGTACAAAATGTTCTTGATTACCTTCAGAATGAATATCCTGACATGGATGTCATCGGCATATCTGGTATTCCACCTACCCT
TTAGTTTTTCCTTAACTTCTCTGCTATGCATGGTGATCTGGTTCTGCTTTTTGTTCCCCCTAAAATAGAGATGTTTGTCTAATTAAAGTTATCAAT
AACTGTGACAGGGAACTTTTGCTCGGACAAGAAGCCATCAGCAGTTAATTGGATCGAGGGGAGAGGAAAGTCTGTAGTTTGTGAGGCAATT
ATTACAGAAGAGGTGGTGAAGAAAGTTCTAAAAACTGAGGTTGCTGCTCTTGTGGAGCTGAACATGCTTAAAAATCTTACTGGCTCTGCCAT
GGCTGGTGCCCTTGGTGGTTTCAATGCTCATGCCAGCAATATCGTCTCAGCTGTGTTTATTGCCACTGGTCAGGATCCAGCTCAGAACATAGA
GAGCTCGCACTGCATCACTATGATGGAGGCTGTAAATGATGGCAAGGACCTCCATATTTCTGT
HMG2_cmm26_seq1
TGGTGTCCAAAGGTGTACAAAATGTTCTTGATTACCTTCAGAATGAATATCCTGACATGGATGTCATCGGCATATCTGGTATTCCACCTACCCT
TTAGTTTTTCCTTAACTTCTCTGCTATGCATGGTGATCTGGTTCTGCTTTTTGTTCCCCCTAAAATAGAGATGTTTGTCTAATTAAAGTTATCAAT
AACTGTGACAGGGAACTTTTGCTCGGACAAGAAGCCATCAGCAGTTAATTGGATCGAGGGGAGAGGAAAGTCTGTAGTTTGTGAGGCAATT
ATTACAGAAGAGGTGGTGAAGAAAGTTCTAAAAACTGAGGTTGCTGCTCTTGTGGAGCTGAACATGCTTAAAAATCTTACTGGCTCTGCCAT
GGCTGGTGCCCTTGGTGGTTTCAATGCTCATGCCAGCAATATCGTCTCAGCTGTGTTTATTGCCACTGGTCAGGATCCAGCTCAGAACATAGA
GAGCTCGCACTGCATCACTATGATGGAGGCTGTAAATGATGGCAAGGACCTCCATATTTCTGT
HMG2_cmm26_seq1-2
TGGTGTCCAAAGGTGTACAAAATGTTCTTGATTACCTTCAGAATGAATATCCTGACATGGATGTCATCGGCATATCTGGTATTCCACCTACCCT
TTAGTTTTTCCTTAACTTCTCTGCTATGCATGGTGATCTGGTTCTGCTTTTTGTTCCCCCTAAAATAGAGATGTTTGTCTAATTAAAGTTATCAAT
AACTGTGACAGGGAACTTTTGCTCGGACAAGAAGCCATCAGCAGTTAATTGGATCGAGGGGAGAGGAAAGTCTGTAGTTTGTGAGGCAATT
ATTACAGAAGAGGTGGTGAAGAAAGTTCTAAAAACTGAGGTTGCTGCTCTTGTGGAGCTGAACATGCTTAAAAATCTTACTGGCTCTGCCAT
GGCTGGTGCCCTTGGTGGTTTCAATGCTCATGCCAGCAATATCGTCTCAGCTGTGTTTATTGCCACTGGTCAGGATCCAGCTCAGAACATAGA
GAGCTCGCACTGCATCACTATGATGGAGGCTGTAAATGATGGCAAGGACCTCCATATTTCTGT
HMG2_dms54_seqR
TGGTGTCCAAAGGTGTACAAAATGTTCTTGATTACCTTCAGAATGAATATCCCGACATGGATGTCATCGGCATATCTGGTATTCCATCTACCTT
TATAGTAATTTGTTTCAAATAGGAGTAGCAAAATTTTTCAAAAAAATAATTTTCATAACGAAAAATATGGTGATCTGGTTCTGCTTTTTGTTCCC
CCTAAAATAGAGATGTTTGTCTAATTAAAGTTATCAATAACTGTGACAGGGAACTTTTGCTCGGACAAGAAGCCATCAGCAGTTAATTGGATC
GAGGGGAGAGGAAAGTCTGTAGTTTGTGAGGCAATTATCACAGAAGAGGTGGTGAAGAAAGTTCTGAAAACTGAAGTTGCTGCTCTTGTGG
AGCTGAACATGCTTAAAAATCTTACTGGCTCTGCCATGGCTGGTGCTCTTGGTGGTTTCAATGCCCATGCCAGCAATATCGTCTCTGCTGTGTT
TATAGCCACTGGTCAGGATCCAGCCCAGAACATAGAGAGCTCGCACTGCATCACTATGATGGAGGCTGTAAATGATGGCAAGGACCTCCATA
TTTCTGT
HMG2_dms54_seq2
TGGTGTCCAAAGGTGTACAAAATGTTCTTGATTACCTTCAGAATGAATATCCTGACATGGATGTCATCGGCATATCTGGTATTCCACCTACCCT
TTAGTTTTTCCTTAACTTCTCTGCTATGCATGGTGATCTGGTTCTGCTTTAATTTTTGTTCCCCCTAAAATAGAGATGTTTGTCTAATTAAAGTTA
TCAATAACTGTGACAGGGAACTTTTGCTCGGACAAGAAGCCATCAGCAGTTAATTGGATCGAGGGGAGAGGAAAGTCTGTAGTTTGTGAGG
CAATTATCACAGAAGAGGTGGTGAAGAAAGTTCTGAAAACTGAAGTTGCTGCTCTTGTGGAGCTGAACATGCTTAAAAATCTTACTGGCTCTG
CCATGGCTGGTGCTCTTGGTGGTTTCAATGCCCATGCCAGCAATATCGTCTCAGCTGTGTTTATAGCCACTGGTCAGGATCCAGCTCAGAACAT
AGAGAGCTCGCACTGCATCACTATGATGGAGGCTGTAAATGATGGCAAGGACCTCCATATTTCTGT
HMG2_dms78_seqR
TGGTGTCCAAAGGTGTACAAAATGTTCTTGATTACCTTCAGAATGAATATCCCGACATGGATGTCATCGGCATATCTGGTATTCCATCTACCTT
TATAGTAATTTGTTTCAAATAGGAGTAGCAAAATTTTTCAAAAAAATAATTTTCATAACGAAAAATATGGTGATCTGGTTCTGCTTTTTGTTCCC
CCTAAAATAGAGATGTTTGTCTAATTAAAGTTATCAATAACTGTGACAGGGAACTTTTGCTCGGACAAGAAGCCATCAGCAGTTAATTGGATC
GAGGGGAGAGGAAAGTCTGTAGTTTGTGAGGCAATTATCACAGAAGAGGTGGTGAAGAAAGTTCTGAAAACTGAAGTTGCTGCTCTTGTGG
AGCTGAACATGCTTAAAAATCTTACTGGCTCTGCCATGGCTGGTGCTCTTGGTGGTTTCAATGCCCATGCCAGCAATATCGTCTCTGCTGTGTT
TATAGCCACTGGTCAGGATCCAGCCCAGAACATAGAGAGCTCGCACTGCATCACTATGATGGAGGCTGTAAATGATGGCAAGGACCTCCATA
TTTCTGT
HMG2_dms78_seq2
TGGTGTCCAAAGGTGTACAAAATGTTCTTGATTACCTTCAGAATGAATATCCTGACATGGATGTCATCGGCATATCTGGTATTCCACCTACCCT
TTAGTTTTTCCTTAACTTCTCTGCTATGCATGGTGATCTGGTTCTGCTTTAATTTTTGTTCCCCCTAAAATAGAGATGTTTGTCTAATTAAAGTTA
TCAATAACTGTGACAGGGAACTTTTGCTCGGACAAGAAGCCATCAGCAGTTAATTGGATCGAGGGGAGAGGAAAGTCTGTAGTTTGTGAGG
CAATTATCACAGAAGAGGTGGTGAAGAAAGTTCTGAAAACTGAAGTTGCTGCTCTTGTGGAGCTGAACATGCTTAAAAATCTTACTGGCTCTG

CCATGGCTGGTGCTCTTGGTGGTTTCAATGCCCATGCCAGCAATATCGTCTCAGCTGTGTTTATAGCCACTGGTCAGGATCCAGCTCAGAACAT
AGAGAGCTCGCACTGCATCACTATGATGGAGGCTGTAAATGATGGCAAGGACCTCCATATTTCTGT
HMG2_spg55_seq1
TGGTGTCCAAAGGTGTACAAAATGTTCTTGATTACCTTCAGAATGAATATCCCGACATGGATGTCATCGGCATATCTGGTATTCCATCTACTAC
CAATAATTTTCATAACGAAAAACATGGTGATCTGTTTGTCTAATTAAAGTTATCTATAACTGTGACAGGGAACTTTTGCTCGGACAAGAAGCCA
GCAGCAGTTAATTGGATTGAGGGGAGAGGAAAGTCTGTAGTTTGTGAGGCAATCATCACAGAAGAGGTGGTGAAGAAAGTTTTGAAAACTG
AGGTTGCTGCTCTTGTGGAGCTGAACATGCTTAAAAATCTTACTGGCTCTGCCATGGCTGGTGCCCTTGGTGGTTTCAATGCCCACGCCAGCA
ATATCGTCTCAGCTGTGTTTATAGCCACTGGTCAGGATCCAGCTCAGAACATAGAGAGCTCGCACTGCATCACTATGATGGAGGCTGTAAATG
ATGGCAAGGACCTCCATATTTCTGT
HMG2_spg55_seq1-2
TGGTGTCCAAAGGTGTACAAAATGTTCTTGATTACCTTCAGAATGAATATCCCGACATGGATGTCATCGGCATATCTGGTATTCCATCTACTAC
CAATAATTTTCATAACGAAAAACATGGTGATCTGTTTGTCTAATTAAAGTTATCTATAACTGTGACAGGGAACTTTTGCTCGGACAAGAAGCCA
GCAGCAGTTAATTGGATTGAGGGGAGAGGAAAGTCTGTAGTTTGTGAGGCAATCATCACAGAAGAGGTGGTGAAGAAAGTTTTGAAAACTG
AGGTTGCTGCTCTTGTGGAGCTGAACATGCTTAAAAATCTTACTGGCTCTGCCATGGCTGGTGCCCTTGGTGGTTTCAATGCCCACGCCAGCA
ATATCGTCTCAGCTGTGTTTATAGCCACTGGTCAGGATCCAGCTCAGAACATAGAGAGCTCGCACTGCATCACTATGATGGAGGCTGTAAATG
ATGGCAAGGACCTCCATATTTCTGT
HMG2_spg74_seqR
TGGTGTCCAAAGGTGTACAAAATGTTCTTGATTACCTTCAGAATGAATATCCCGACATGGATGTCATCGGCATATCTGGTATTCCATCTACCTT
TATAGTAATTTGTTTCAAATAGGAGTAGCAAAATTTTTCAAAAAAATAATTTTCATAACGAAAAATATGGTGATCTGGTTCTGCTTTTTGTTCCC
CCTAAAATAGAGATGTTTGTCTAATTAAAGTTATCAATAACTGTGACAGGGAACTTTTGCTCGGACAAGAAGCCATCAGCAGTTAATTGGATC
GAGGGGAGAGGAAAGTCTGTAGTTTGTGAGGCAATTATCACAGAAGAGGTGGTGAAGAAAGTTCTGAAAACTGAAGTTGCTGCTCTTGTGG
AGCTGAACATGCTTAAAAATCTTACTGGCTCTGCCATGGCTGGTGCTCTTGGTGGTTTCAATGCCCATGCCAGCAATATCGTCTCTGCTGTGTT
TATAGCCACTGGTCAGGATCCAGCCCAGAACATAGAGAGCTCGCACTGCATCACTATGATGGAGGCTGTAAATGATGGCAAGGACCTCCATA
TTTCTGT
HMG2_spg74_seq2
TGGTGTCCAAAGGTGTACAAAATGTTCTTGATTACCTTCAGAATGAATATCCCGACATGGATGTCATCGGCATATCTGGTATTCCATCTACTAG
CAAAAATTTTCATAACGAAAAACATGGTGATCTGTTTGTCTAATTAAAGTTATCTATAACTGTGACAGGGAACTTTTGCTCGGACAAGAAGCC
AGCAGCAGTTAATTGGATTGAGGGGAGAGGAAAGTCTGTAGTTTGTGAGGCAATCATCACAGAAGAGGTGGTGAAGAAAGTTTTGAAAACT
GAGGTTGCTGCTCTTGTGGAGCTGAACATGCTTAAAAATCTTACTGGCTCTGCCATGGCTGGTGCCCTTGGTGGTTTCAATGCCCACGCCAGC
AATATCGTCTCAGCTGTGTTTATAGCCACTGGTCAGGATCCAGCTCAGAACATAGAGAGCTCGCACTGCATCACTATGATGGAGGCTGTAAAT
GATGGCAAGGACCTCCATATTTCTGT
HMG2_spl16_seq1
TGGTGTCCAAAGGTGTACAAAATGTTCTTGATTACCTTCAGAATGAATATCCCGACATGGATGTCATCGGCATATCTGGTATATCATCTACCTT
TATAGTAATTTGTTTCAAATAGGAGTACCAAAAATAATTTTCATAACGAAAAATATGGTGATCTGGTTCTGCTTTAATTTTTGTTCCCCCTAAAA
TAGAGATGTTTGTCTAATTAAAGTTATCAATAACTGTGACAGGGAACTTTTGCTCGGACAAGAAGCCATCAGCAGTTAATTGGATCGAGGGG
AGAGGAAAGTCTGTAGTTTGTGAGGCAATTATCACAGAAGAGGTGGTGAAGAAAGTTCTGAAAACTGAAGTTGCTGCTCTTGTGGAGCTGA
ACATGCTTAAAAATCTTACTGGCTCTGCCATGGCTGGTGCTCTTGGTGGTTTCAATGCCCATGCCAGCAATATCGTCTCAGCTGTGTTTATAGC
CACTGGTCAGGATCCAGCTCAGAACATAGAGAGCTCGCACTGCATCACTATGATGGAGGCTGTAAATGATGGCAAGGACCTCCATATTTCTG
T
HMG2_spl16_seq1-2
TGGTGTCCAAAGGTGTACAAAATGTTCTTGATTACCTTCAGAATGAATATCCCGACATGGATGTCATCGGCATATCTGGTATATCATCTACCTT
TATAGTAATTTGTTTCAAATAGGAGTACCAAAAATAATTTTCATAACGAAAAATATGGTGATCTGGTTCTGCTTTAATTTTTGTTCCCCCTAAAA
TAGAGATGTTTGTCTAATTAAAGTTATCAATAACTGTGACAGGGAACTTTTGCTCGGACAAGAAGCCATCAGCAGTTAATTGGATCGAGGGG
AGAGGAAAGTCTGTAGTTTGTGAGGCAATTATCACAGAAGAGGTGGTGAAGAAAGTTCTGAAAACTGAAGTTGCTGCTCTTGTGGAGCTGA
ACATGCTTAAAAATCTTACTGGCTCTGCCATGGCTGGTGCTCTTGGTGGTTTCAATGCCCATGCCAGCAATATCGTCTCAGCTGTGTTTATAGC
CACTGGTCAGGATCCAGCTCAGAACATAGAGAGCTCGCACTGCATCACTATGATGGAGGCTGTAAATGATGGCAAGGACCTCCATATTTCTG
T
HMG2_spl81_seq2
TGGTGTCCAAAGGTGTACAAAATGTTCTTGATTACCTTCAGAATGAATATCCCGACATGGATGTCATCGGCATATCTGGTATTCCATCTACCTT
TATAGTAATTTGTTTCAAATAGGAGTACCAAAATTTTCAAAAAAATAATTTTCCTAACGAAAAATATGGTGATCTGGTTCTGCTTTTTGTTTCCC
CTAAAATAGAGATGTTTGTCTAATTAAAGTTATCAATAACTGTGACAGGGAACTTTTGCTCGGACAAGAAGCCATCAGCAGTTAATTGGATCG
AGGGGAGAGGAAAGTCTGTAGTTTGTGAGGCAATTATCACAGAAGAGGTGGTGAAGAAAGTTCTGAAAACTGAAGTTGCTGCTCTTGTGGA
GCTGAACATGCTTAAAAATCTTACTGGCTCTGCCATGGCTGGTGCTCTTGGTGGTTTCAATGCCCATGCCAGCAATATCGTCTCTGCTGTGTTT
ATAGCCACTGGTCAGGATCCAGCCCAGAACATAGAGAGCTCGCACTGCATCACTATGATGGAGGCTGTAAATGATGGCAAGGACCTCCATAT
TTCTGT
HMG2_spl81_seq2-2
TGGTGTCCAAAGGTGTACAAAATGTTCTTGATTACCTTCAGAATGAATATCCCGACATGGATGTCATCGGCATATCTGGTATTCCATCTACCTT
TATAGTAATTTGTTTCAAATAGGAGTACCAAAATTTTCAAAAAAATAATTTTCCTAACGAAAAATATGGTGATCTGGTTCTGCTTTTTGTTTCCC

CTAAAATAGAGATGTTTGTCTAATTAAAGTTATCAATAACTGTGACAGGGAACTTTTGCTCGGACAAGAAGCCATCAGCAGTTAATTGGATCG
AGGGGAGAGGAAAGTCTGTAGTTTGTGAGGCAATTATCACAGAAGAGGTGGTGAAGAAAGTTCTGAAAACTGAAGTTGCTGCTCTTGTGGA
GCTGAACATGCTTAAAAATCTTACTGGCTCTGCCATGGCTGGTGCTCTTGGTGGTTTCAATGCCCATGCCAGCAATATCGTCTCTGCTGTGTTT
ATAGCCACTGGTCAGGATCCAGCCCAGAACATAGAGAGCTCGCACTGCATCACTATGATGGAGGCTGTAAATGATGGCAAGGACCTCCATAT
TTCTGT
HMG2_sto40_seqR
TGGTGTCCAAAGGTGTACAAAATGTTCTTGATTACCTTCAGAATGAATATCCCGACATGGATGTCATCGGCATATCTGGTATTCCATCTACCTT
TATAGTAATTTGTTTCAAATAGGAGTAGCAAAATTTTTCAAAAAAATAATTTTCATAACGAAAAATATGGTGATCTGGTTCTGCTTTTTGTTCCC
CCTAAAATAGAGATGTTTGTCTAATTAAAGTTATCAATAACTGTGACAGGGAACTTTTGCTCGGACAAGAAGCCATCAGCAGTTAATTGGATC
GAGGGGAGAGGAAAGTCTGTAGTTTGTGAGGCAATTATCACAGAAGAGGTGGTGAAGAAAGTTCTGAAAACTGAAGTTGCTGCTCTTGTGG
AGCTGAACATGCTTAAAAATCTTACTGGCTCTGCCATGGCTGGTGCTCTTGGTGGTTTCAATGCCCATGCCAGCAATATCGTCTCTGCTGTGTT
TATAGCCACTGGTCAGGATCCAGCCCAGAACATAGAGAGCTCGCACTGCATCACTATGATGGAGGCTGTAAATGATGGCAAGGACCTCCATA
TTTCTGT
HMG2_sto40_seqR-2
TGGTGTCCAAAGGTGTACAAAATGTTCTTGATTACCTTCAGAATGAATATCCCGACATGGATGTCATCGGCATATCTGGTATTCCATCTACCTT
TATAGTAATTTGTTTCAAATAGGAGTAGCAAAATTTTTCAAAAAAATAATTTTCATAACGAAAAATATGGTGATCTGGTTCTGCTTTTTGTTCCC
CCTAAAATAGAGATGTTTGTCTAATTAAAGTTATCAATAACTGTGACAGGGAACTTTTGCTCGGACAAGAAGCCATCAGCAGTTAATTGGATC
GAGGGGAGAGGAAAGTCTGTAGTTTGTGAGGCAATTATCACAGAAGAGGTGGTGAAGAAAGTTCTGAAAACTGAAGTTGCTGCTCTTGTGG
AGCTGAACATGCTTAAAAATCTTACTGGCTCTGCCATGGCTGGTGCTCTTGGTGGTTTCAATGCCCATGCCAGCAATATCGTCTCTGCTGTGTT
TATAGCCACTGGTCAGGATCCAGCCCAGAACATAGAGAGCTCGCACTGCATCACTATGATGGAGGCTGTAAATGATGGCAAGGACCTCCATA
TTTCTGT
HMG2_sto61_seqR
TGGTGTCCAAAGGTGTACAAAATGTTCTTGATTACCTTCAGAATGAATATCCCGACATGGATGTCATCGGCATATCTGGTATTCCATCTACCTT
TATAGTAATTTGTTTCAAATAGGAGTAGCAAAATTTTTCAAAAAAATAATTTTCATAACGAAAAATATGGTGATCTGGTTCTGCTTTTTGTTCCC
CCTAAAATAGAGATGTTTGTCTAATTAAAGTTATCAATAACTGTGACAGGGAACTTTTGCTCGGACAAGAAGCCATCAGCAGTTAATTGGATC
GAGGGGAGAGGAAAGTCTGTAGTTTGTGAGGCAATTATCACAGAAGAGGTGGTGAAGAAAGTTCTGAAAACTGAAGTTGCTGCTCTTGTGG
AGCTGAACATGCTTAAAAATCTTACTGGCTCTGCCATGGCTGGTGCTCTTGGTGGTTTCAATGCCCATGCCAGCAATATCGTCTCTGCTGTGTT
TATAGCCACTGGTCAGGATCCAGCCCAGAACATAGAGAGCTCGCACTGCATCACTATGATGGAGGCTGTAAATGATGGCAAGGACCTCCATA
TTTCTGT
HMG2_sto61_seq2
TGGTGTCCAAAGGTGTACAAAATGTTCTTGATTACCTTCAGAATGAATATCCCGACATGGATGTCATCGGCATATCTGGTATTCCATCTACCTT
TATAGTAATTTGTTTCAAATAGGAGTACCAAAATTTTTCAAAAAAATAATTTTCATAACGAAAAATATGGTGATCTGGTTCTGCTTTTTGTTCCC
CCTAAAATAGAGATGTTTGTCTAATTAAAGTTATCAATAACTGTGACAGGGAACTTTTGCTCGGACAAGAAGCCAGCAGCAGTTAATTGGATC
GAGGGGAGAGGAAAGTCTGTAGTTTGTGAGGCAACTATCACAGAAGATGTGGTGAAGAAAGTTCTGAAAACTGAAGTTGCTGCTCTTGTGG
AGCTGAACATGCTTAAAAATCTTACTGGCTCTGCCATGGCTGGTGCCCTTGGTGGTTTCAATGCCCACGCCAGCAATATCGTCTCAGCTTTGTT
TATAGCCACTGGTCAGGATCCAGCTCAGAACATAGAGAGCTCGCACTGCATCACTATGATGGAGGCTGTAAATGATGGCAAGGACCTCCATA
TTTCTGT
HMG2_chc80-1_seq1
TGGTGTCCAAAGGTGTACAAAATGTTCTTGATTACCTTCAGAATGAATATCCCGACATGGATGTCATCGGCATATCTGGTATTCCATCTACTAG
CAAAAATTTTCATAACGAAAAACATGGTGATCTGTTTGTCTAATTAAAGTTATCTATAACTGTGACAGGGAACTTTTGCTCGGACAAGAAGCC
AGCAGCAGTTAATTGGATTGAGGGGAAAGGAAAGTCTGTAGTTTGTGAGGCAATCATCACAGAAGAGGTGGTGAAGAAAGTTTTGAAAACT
GAGGTTTCTGCTCTTGTGGAGCTGAACATGCTTAAAAATCTTACTGGCTCTGCCATGGCTGGTGCCCTTGGTGGTTTCAATGCCCACGCCAGC
AATATCGTCTCAGCTGTGTTTATAGCCACTGGTCAGGATCCAGCTCAGAACATAGAGAGCTCGCACTGCATCACTATGATGGAGGCTGTAAAT
GATGGCAAGGACCTCCATATTTCTGT
HMG2_chc80-1_seq2
TGGTGTCCAAAGGTGTACAAAATGTTCTTGATTACCTTCAGAATGAATATCCCGACATGGATGTCATCGGCATATCTGGTATTCCATCTACTAG
CAAAAATTTTCATAACGAAAAACATGGTGATCTGTTTGTCTAATTAAAGTTATCTATAACTGTGACAGGGAACTTTTGCTCGGACAAGAAGCC
AGCAGCAGTTAATTGGATTGAGGGGAGAGGAAAGTCTGTAGTTTGTGAGGCAATCATCACAGAAGAGGTGGTGAAGAAAGTTTTGAAAACT
GAGGTTTCTGCTCTTGTGGAGCTGAACATGCTTAAAAATCTTACTGGCTCTGCCATGGCTGGTGCCCTTGGTGGTTTCAATGCCCACGCCAGC
AATATCGTCTCAGCTGTGTTTATAGCCACTGGTCAGGATCCAGCTCAGAACATAGAGAGCTCGCACTGCATCACTATGATGGAGGCTGTAAAT
GATGGCAAGGACCTCCATATTTCTGT
HMG2_phuDH_seq1
TGGTGTCCAAAGGTGTACAAAATGTTCTTGATTACCTTCAGAATGAATATCCCGACATGGATGTCATCGGCATATCTGGTATTCCATCTACTAG
CAAAAATTTTCATAACGAAAAACATGGTGATCTGTTTGTCTAATTAAAGTTATCTATAACTGTGACAGGGAACTTTTGCTCGGACAAGAAGCC
AGCAGCAGTTAATTGGATTGAGGGGAGAGGAAAGTCTGTAGTTTGTGAGGCAATCATCACAGAAGAGGTGGTGAAGAAAGTTTTGAAAACT
GAGGTTGCTGCTCTTGTGGAGCTGAACATGCTTAAAAATCTTACTGGCTCTGCCATGGCTGGTGCCCTTGGTGGTTTCAATGCCCACGCCAGC
AATATCGTCTCAGCTGTGTTTATAGCCACTGGTCAGGATCCAGCTCAGAACATAGAGAGCTCGCACTGCATCACTATGATGGAGGCTGTAAAT
GATGGCAAGGACCTCCATATTTCTGT

HMG2_phuDH_seq2
TGGTGTCCAAAGGTGTACAAAATGTTCTTGATTACCTTCAGAATGAATATCCTGACATGGATGTCATCGGCATATCTGGTATTCCACCTACCCT
TTAGTTTTTCCTTAACTTCTCTGCTATGCATGGTGATCTGGTTCTGCTTTAATTTTTGTTCCCCCTAAAATAGAGATGTTTGTCTAATTAAAGTTA
TCAATAACTGTGACAGGGAACTTTTGCTCGGACAAGAAGCCATCAGCAGTTAATTGGATCGAGGGGAGAGGAAAGTCTGTAGTTTGTGAGG
CAATTATCACAGAAGAGGTGGTGAAGAAAGTTCTGAAAACTGAAGTTGCTTCTCTTGTGGAGCTGAACATGCTTAAAAATCTTACTGGCTCTG
CCATGGCTGGTGCTCTTGGTGGTTTCAATGCCCATGCCAGCAATATCGTCTCAGCTGTGTTTATAGCCACTGGTCAGGATCCAGCTCAGAACAT
AGAGAGCTCGCACTGCATCACTATGATGGAGGCTGTAAATGATGGCAAGGACCTCCATATTTCTGT
HMG2_phuDM_seq2
TGGTGTCCAAAGGTGTACAAAATGTTCTTGATTACCTTCAGAATGAATATCCTGACATGGATGTCATCGGCATATCTGGTATTCCACCTACCCT
TTAGTTTTTCCTTAACTTCTCTGCTATGCATGGTGATCTGGTTCTGCTTTAATTTTTGTTCCCCCTAAAATAGAGATGTTTGTCTAATTAAAGTTA
TCAATAACTGTGACAGGGAACTTTTGCTCGGACAAGAAGCCATCAGCAGTTAATTGGATCGAGGGGAGAGGAAAGTCTGTAGTTTGTGAGG
CAATTATCACAGAAGAGGTGGTGAAGAAAGTTCTGAAAACTGAAGTTGCTTCTCTTGTGGAGCTGAACATGCTTAAAAATCTTACTGGCTCTG
CCATGGCTGGTGCTCTTGGTGGTTTCAATGCCCATGCCAGCAATATCGTCTCAGCTGTGTTTATAGCCACTGGTCAGGATCCAGCTCAGAACAT
AGAGAGCTCGCACTGCATCACTATGATGGAGGCTGTAAATGATGGCAAGGACCTCCATATTTCTGT
HMG2_phuDM_seq2-2
TGGTGTCCAAAGGTGTACAAAATGTTCTTGATTACCTTCAGAATGAATATCCTGACATGGATGTCATCGGCATATCTGGTATTCCACCTACCCT
TTAGTTTTTCCTTAACTTCTCTGCTATGCATGGTGATCTGGTTCTGCTTTAATTTTTGTTCCCCCTAAAATAGAGATGTTTGTCTAATTAAAGTTA
TCAATAACTGTGACAGGGAACTTTTGCTCGGACAAGAAGCCATCAGCAGTTAATTGGATCGAGGGGAGAGGAAAGTCTGTAGTTTGTGAGG
CAATTATCACAGAAGAGGTGGTGAAGAAAGTTCTGAAAACTGAAGTTGCTTCTCTTGTGGAGCTGAACATGCTTAAAAATCTTACTGGCTCTG
CCATGGCTGGTGCTCTTGGTGGTTTCAATGCCCATGCCAGCAATATCGTCTCAGCTGTGTTTATAGCCACTGGTCAGGATCCAGCTCAGAACAT
AGAGAGCTCGCACTGCATCACTATGATGGAGGCTGTAAATGATGGCAAGGACCTCCATATTTCTGT
HMG2_tbr_seq1
TGGTGTCCAAAGGTGTACAAAATGTTCTTGATTACCTTCAGAATGAATATCCCGACATGGATGTCATCGGCATATCTGGGAACTTTTGCTCGG
ACAAGAAGCCAGCAGCAGTTAATTGGATCGAGGGGAGAGGAAAGTCTGTAGTTTGTGAGGCAATTATCACAGAAGAGGTGGTGAAGAAAG
TTCTGAAAACTGAAGTTGCTGCTCTTGTGGAGCTGAACATGCTTAAAAATCTTACTGGCTCTGCCATGGCTGGTGCCCTTGGTGGTTTCAATGC
CCATGCCAGCAATATCGTCTCAGCTGTGTTTATAGCCACTGGTCAGGATCCAGCTCAGAACATAGAGAGCTCGCACTGCATCACTATGATGGA
GGCTGTAAATGATGGCAAGGACCTCCATATTTCTGT
HMG2_tbr_seq1-2
TGGTGTCCAAAGGTGTACAAAATGTTCTTGATTACCTTCAGAATGAATATCCCGACATGGATGTCATCGGCATATCTGGGAACTTTTGCTCGG
ACAAGAAGCCAGCAGCAGTTAATTGGATCGAGGGGAGAGGAAAGTCTGTAGTTTGTGAGGCAATTATCACAGAAGAGGTGGTGAAGAAAG
TTCTGAAAACTGAAGTTGCTGCTCTTGTGGAGCTGAACATGCTTAAAAATCTTACTGGCTCTGCCATGGCTGGTGCCCTTGGTGGTTTCAATGC
CCATGCCAGCAATATCGTCTCAGCTGTGTTTATAGCCACTGGTCAGGATCCAGCTCAGAACATAGAGAGCTCGCACTGCATCACTATGATGGA
GGCTGTAAATGATGGCAAGGACCTCCATATTTCTGT

1.3 2,3-squalene epoxidase (SQE) sequences with introns

26

SQE_cmm7_seq1

TTGGGGTTCGTTGCAGTTTTCTGTTTTTTCATTCAGAAAAATGGTGGCCGCAGCAAAAGTAAAATTGATGATTCGGCAACTAGCACCACTACA
ACTACCGTGTACAGTGGAGAGTCCAGAACAAAGGATGGAAACGACGACGTTGATATCATCATCGTCGGTGCCGGAGTTGCCGGTGCTGCTCT
TGCTCACACTCTTGGCAAGGTATTGTTTCTTTCTTTGTAATCCGTTTTTTAAAAGTTACTATTCTACTTTGGTCCTTCAACTATTTTTTATGTACAT
ATGTGAGCAAACCGTAATTGTGTAGTGAGAAGTAATTAGATTGATTGATTTACGTATCTGAAGGCATAGTTGAGTGGTTATTCATAAAAAACT
TGAACTTGATTGTTAATTTGATGAAATCAGAGAAATGAAAGATGCAATGATGATGAAAGCGTTTGGTGTGACTGCAAATTTTTTTGTTGGAGT
TCTTTGTTGACAATTCTCTATGAAAAAAAAATTCTTTTTTGTGAGATTTATGATTATACATTTGGGACTACAGGAAGGGCGTCGTGTAAAAGTAA
TTGAAAGAGATTTGACAGAGCCTGATCGAATTGTTGGAGAACTCCTACAACCGGGTGGTTACCTCAAATTGCAGGAGTTGGGATTGGAAGGT
AAAGTTCCTAAGTGATGTGATTTGTGTCAATTCTGATTAATTTACTCTCTTGAAGAGAAAAAAAAGGAAGTTAGTCTTACACTTTTAGCTTCTC
GTTTGTCTCTTTTTCGGCATTTCTCATATTTGTATTTTGGTCTACTGCAGATTGTGTGGAGAAAATTGATGCTCAACGAGTGTTTGGGTATGCCC
TTTTCAAGGATGGAAAGAGTACACGTCTTTCTTATCCCCTGG

>SQE_cmm7_seq2

TTGGGGTTCGTTGCAGTTTTCTGTTTTTTCATTCAGAAAAATGGTGGCCGCAGCAAAAGTAAAATTGATGATTCGGCAACTAGCACCACTACA
ACTACCGTGTACAGTGGAGAGTCCAGAACAAAGGATGGAAACGACGACGTTGATATCATCATCGTCGGTGCCGGAGTTGCCGGTGCTGCTCT
TGCTCACACTCTTGGCAAGGTATTGTTTCTTTCTTTGTAATCCGTTTTTTAAAAGTTACTATTCTACTTTGGTCCTTCAACTATTTTTTATGTACAT
ATGTGAGCAAACCGTAATTGTGTAGTGAGAAGTAATTAGATTGATTGATTTACGTATCTGAAGGCATAGTTGAGTGGTTATTCATAAAAAACT
TGAACTTGATTGTTAATTTGATGAAATCAGAGAAATGAAAGATGCATTGATGATGAAAGCGTTTGGTGTGACTGCAATTTTTTTTATTGGAGT
TCTTTGTTGACAATTCGCTATGAAAAAAAATTCTTTTTCGTGAGATTTATGATTATACATTTGGGACTACAGGAAGGGCGTCGTGTAAAAGTAAT
TGAAAGAGATTTGACAGAGCCTGATCGAATTGTTGGAGAACTCCTACAACCGGGTGGTTACCTCAAATTGCAGGAGTTGGGATTGGAAGGTA
AAGTTCCTAAGTGATGTGATTTGTGTCAATTCTGATTAATTTACTCTCTTGAAGAGCAAAAAAAGGAAGTTAGTCTTACACTTTTAGCTTCTCGT
TTGTCTCTTTTTCGGCATTTCTCATATTTGTATTTTGGTCTACTGCAGATTGTGTGGAGAAAATTGATGCTCAACGAGTGTTTGGGTATGCCCTT
TTCAAGGATGGAAAGAGTACACGTCTTTCTTATCCCCTGG

SQE_cmm26_seq2

TTGGGGTTCGTTGCAGTTTTCTGTTTTTTCATTCAGAAAAATGGTGGCCGCAGCAAAAGTAAAATTGATGATTCGGCAACTAGCACCACTACA
ACTACCGTGTACAGTGGAGAGTCCAGAACAAAGGATGGAAACGACGACGTTGATATCATCATCGTCGGTGCCGGAGTTGCCGGTGCTGCTCT
TGCTCACACTCTTGGCAAGGTATTGTTTCTTTCTTTGTAATCCGTTTTTTAAAAGTTACTATTCTACTTTGGTCCTTCAACTATTTTTTATGTACAT
ATGTGAGCAAACCGTAATTGTGTAGTGAGAAGTAATTAGATTGATTGATTTACGTATCTGAAGGCATAGTTGAGTGGTTATTCATAAAAAACT
TGAACTTGATTGTTAATTTGATGAAATCAGAGAAATGAAAGATGCATTGATGATGAAAGCGTTTGGTGTGACTGCAATTTTTTTTATTGGAGT
TCTTTGTTGACAATTCGCTATGAAAAAAAATTCTTTTTCGTGAGATTTATGATTATACATTTGGGACTACAGGAAGGGCGTCGTGTAAAAGTAAT
TGAAAGAGATTTGACAGAGCCTGATCGAATTGTTGGAGAACTCCTACAACCGGGTGGTTACCTCAAATTGCAGGAGTTGGGATTGGAAGGTA
AAGTTCCTAAGTGATGTGATTTGTGTCAATTCTGATTAATTTACTCTCTTGAAGAGCAAAAAAAGGAAGTTAGTCTTACACTTTTAGCTTCTCGT
TTGTCTCTTTTTCGGCATTTCTCATATTTGTATTTTGGTCTACTGCAGATTGTGTGGAGAAAATTGATGCTCAACGAGTGTTTGGGTATGCCCTT
TTCAAGGATGGAAAGAGTACACGTCTTTCTTATCCCCTGG

SQE_cmm26_seq3

TTGGGGTTCGTTGCAGTTTTCTGTTTTTTCATTCAGAAAAATGGTGGCCGCAGCAAAAGTAAAATTGATGACTCGGCAACTAGCACCACTACA
ACTACCGTGTACAGTGGAGAGTCCAGAACAAAGGATGGAAACGACGACGTTGATATCATCATCGTCGGTGCCGGAGTTGCCGGTGCTGCTCT
TGCTCACACTCTTGGCAAGGTATTGTTTCTTTCTTTGTAATCCGTTTTTTAAAAGTTACTATTCTACTTTGGTCCTTCAACTATTTTTTATGTACAT
ATGTGAGCAAACCGTAATTGTGTAGTGAGAAGTAATTAGATTGATTGATTTACGTATCTGAAGGCATAGTTGAGTGGTTATTCATAAAAAACT
TGAACTTGATTGTTAATTTGATGAAATCAGAGAAATGAAAGATGCATTGATGATGAAAGCGTTTGGTGTGACTGCAAATTTTTTTATTGGAGT
TCTTTGTTGACAATTCGCTATGAAAAAAAATTCTTTTTCGTGAGATTTAAGATTATACATTTGGGACTACAGGAAGGGCGTCGTGTAAAAGTAAT
TGAAAGAGATTTGACAGAGCCTGATCGAATTGTTGGAGAACTCCTACAACCGGGTGGTTACCTCAAATTGCAGGAGTTGGGATTGGAAGGTA
AAGTTCCTAAGTGATGTGATTTGTGTCAATTCTGATTAATTTACTCTCTTGAAGAGAAAAAAAGGGAAGTTAGTCTTACACTTTTAGCTTCTCG
TTTGTCTCTTTTTCGGCATTTCTCATATTTGTATTTTGGTCTACTGCAGATTGTGTGGAGAAAATTGATGCTCAACGAGTGTTTGGGTATGCCCT
TTTCAAGGATGGAAAGAGTACACGTCTTTCTTATCCCCTGG

SQE_dms54_seq1

TTGGGGTTCGTTGCAGTTTTCTGTTTTTTCATTCAGAGAAATGGTGGCTGCAGCAAAAGTAAAATTGATGACTCAGCAACTAGCACCACTACA
ACTACGGTGTACAGTGGAAAGTCCCGATCAAAGGATGGAAACGACGACGTTGATATCATCATCGTTGGTGCCGGAGTTGCCGGTGCTGCTCT
TGCTCACACTCTTGGCAAGGTATTGTTTCTTTCTTTGTAATCCTTTTTTTTAAAGTTATTGTTCTACTTTGATCCTTTAACTATTTTTATGTACATAT
GTGAGCAAACCGTAATTGTGAAGTGAGAAGTAATTAGACTGATTGATTTACGTATCTGAAGACATAGTTGATGTGGTTATTCATAAAAGACTT
GAACTTGATTGTTAATTTGATGAAACCAGAGAAATGAAAGATGCATTGATGATGAAAGCATTTGGTGTGACTGCAAATTTTTTTATTGGAGTT
CTTTGTTGACAATTCTCTATGAAAAAAAAATTCTTTTTTGTGAGATTTATGATTATACATTTGGGACTACAGGAAGGGCGTCGTGTAAAAGTAAT
TGAAAGAGATTTGACAGAGCCTGATCGAATTGTTGGAGAACTCCTACAACCGGGTGGTTACCTCAAATTGCAGGAGTTGGGATTGGAAGGTA
AAGTTCCTAAGTGATGTGATTTGTGTCAATTCTGGTTAATTTACTCTCTTGAAGAGAAAAAAAAGGAAGTTAGTCTTACACTTTTAGCTTCTCG
TCTGTCTCTTTTTTGGCATTTCTCATATTTGTATTTTGGTCTACTGCAGATTGTGTGGAGAAAATTGATGCTCAACGAGTGTTTGGGTATGCCCT
TTTCAAGGATGGAAAGAGTACACGTCTTTCTTATCCCCTGG

SQE_dms54_seq2

TTGGGGTTCGTTGCAGTTTTCTGTTTTTTCATTCAGAGAAATGGTGGCTGCAGCAAAAGTAAAATTGATGACTCAGCAACTAGCACCACTACA
ACTACGGTGTACAGTGGAAAGTCCCGATCAAAGGATGGAAACGACGACGTTGATATCATCATCGTCGGTGCCGGAGTTGCCGGTGCTGCTCT
TGCTCACACTCTTGGCAAGGTATTGTTTCTTTCTTTGTAATCCGTTTTTTTAAAGTTATATTCTACTTGGTCCTTCAACTATTTTTTATGTACATAT
GTGAGCAAACCGTAATTGTGTAGTGAGAAGTAATTAGATTGATTGATTTACGTATCTGAAGGCATAGTTGATGTGGTTATTCATAAAAAACTT
GAACTTGATTGTTAATTTGATGAAATCAGAGAAATGAAAGATCCATTGATGATGAAAGCGTTTGGTGTGACTGCAAATAATTTTATTGGAGTT
CTTTGTTGACAATTCTCTATAAAAAAAATTATTTTTTTGTGAGATTTATGATTATACATTTGGGACTACAGGAAGGGCGTCGTGTAAAAGTAAT
TGAAAGAGATTTGACAGAGCCTGATCGAATTGTTGGAGAACTCCTACAACCGGGTGGTTACCTCAAATTGCAGGAGTTGGGATTGGAAGGTA
AAGTTCCTAAGTGATGTGATTTGTGTCAATTCTGGTTAATTTACTCTCTTGAAGAGAAAAAAAAGGAAGTTAGTCTTACGCTTTTAGCTTCTCG
TCTGTCTCTTTTTCGGCATTTCTCATATTTGTATTTTGGTCTACTGCAGATTGTGTGGAGAAAATTGATGCTCAACGAGTGTTTGGGTATGCCCT
TTTCAAGGATGGAAAGAGTACACGTCTTTCTTATCCCCTGG
SQE_dms78_seq1
TTGGGGTTCGTTGCAGTTTTCTGTTTTTTCATTCAGAGAAATGGTGGCTGCAGCAAAAGTAAAATTGATGACTCAGCAACTAGCACCACTACA
ACTACGGTGTACAGTGGAAAGTCCCGATCAAAGGATGGAAACGACGACGTTGATATCATCATCGTTGGTGCCGGAGTTGCCGGTGCTGCTCT
TGCTCACACTCTTGGCAAGGTATTGTTTCTTTCTTTGTAATCCTTTTTTTTAAAGTTATTGTTCTACTTTGATCCTTTAACTATTTTTATGTACATAT
GTGAGCAAACCGTAATTGTGAAGTGAGAAGTAATTAGACTGATTGATTTACGTATCTGAAGACATAGTTGATGTGGTTATTCATAAAAGACTT
GAACTTGATTGTTAATTTGATGAAACCAGAGAAATGAAAGATGCATTGATGATGAAAGCATTTGGTGTGACTGCAAATTTTTTTATTGGAGTT
CTTTGTTGACAATTCTCTATGAAAAAAAAATTCTTTTTTGTGAGATTTATGATTATACATTTGGGACTACAGGAAGGGCGTCGTGTAAAAGTAAT
TGAAAGAGATTTGACAGAGCCTGATCGAATTGTTGGAGAACTCCTACAACCGGGTGGTTACCTCAAATTGCAGGAGTTGGGATTGGAAGGTA
AAGTTCCTAAGTGATGTGATTTGTGTCAATTCTGGTTAATTTACTCTCTTGAAGAGAAAAAAAAGGAAGTTAGTCTTACACTTTTAGCTTCTCG
TCTGTCTCTTTTTTGGCATTTCTCATATTTGTATTTTGGTCTACTGCAGATTGTGTGGAGAAAATTGATGCTCAACGAGTGTTTGGGTATGCCCT
TTTCAAGGATGGAAAGAGTACACGTCTTTCTTATCCCCTGG
SQE_dms78_seq2
TTGGGGTTCGTTGCAGTTTTCTGTTTTTTCATTCAGAGAAATGGTGGCTGCAGCAAAAGTAAAATTGATGACTCAGCAACTAGCACCACTACA
ACTACGGTGTACAGTGGAAAGTCCCGATCAAAGGATGGAAACGACGACGTTGATATCATCATCGTCGGTGCCGGAGTTGCCGGTGCTGCTCT
TGCTCACACTCTTGGCAAGGTATTGTTTCTTTCTTTGTAATCCGTTTTTTTAAAGTTATATTCTACTTGGTCCTTCAACTATTTTTTATGTACATAT
GTGAGCAAACCGTAATTGTGTAGTGAGAAGTAATTAGATTGATTGATTTACGTATCTGAAGGCATAGTTGATGTGGTTATTCATAAAAAACTT
GAACTTGATTGTTAATTTGATGAAATCAGAGAAATGAAAGATCCATTGATGATGAAAGCGTTTGGTGTGACTGCAAATAATTTTATTGGAGTT
CTTTGTTGACAATTCTCTATAAAAAAAATTATTTTTTTGTGAGATTTATGATTATACATTTGGGACTACAGGAAGGGCGTCGTGTAAAAGTAAT
TGAAAGAGATTTGACAGAGCCTGATCGAATTGTTGGAGAACTCCTACAACCGGGTGGTTACCTCAAATTGCAGGAGTTGGGATTGGAAGGTA
AAGTTCCTAAGTGATGTGATTTGTGTCAATTCTGGTTAATTTACTCTCTTGAAGAGAAAAAAAAGGAAGTTAGTCTTACGCTTTTAGCTTCTCG
TCTGTCTCTTTTTCGGCATTTCTCATATTTGTATTTTGGTCTACTGCAGATTGTGTGGAGAAAATTGATGCTCAACGAGTGTTTGGGTATGCCCT
TTTCAAGGATGGAAAGAGTACACGTCTTTCTTATCCCCTGG
SQE_spg55_seq1
TTGGGGTTCGTTGCAGTTTTCTGTTTTTTCATTCAGAGAAATGGTGGCTGCAGCAAAAGTAAAATTGATGATTCAGCAACTAGCACCACTACA
ACTACGGTGTACAGTGGAAAGTCCCGATCAAAGGATGGAAACGACGACGTTGATATCATCATCGTCGGTGCCGGAGTTGCCGGTGCTGCTCT
TGCTCACACTCTTGGCAAGGTATTGTTTCTTTCTTTGTAATCCTTTTTTTTAAAGTTATTGTTCTACTTTGGTCCTTTAACTATTTTTATGTACATAT
GTGAGCAAACCGTAATTGTGTAGTGAGAAGTAATTAGACTGATTGATTTACGTATCTGAAGACATAGTTGATGTGGTTATTCATAAAAAACTT
GAACTTGATTGTTAATTTGATGAATCAGAGAAATGAAAGATGCATTGATGATGAAAGCGTTTGGTGTGACTGCAATAGTTTTATTGGAGTTCT
TTGTTGACAATTCTCTATAAAAAAAATTATTTTTTTGTGAGATTTATGATTATACATTTGGGACTACAGGAAGGGCGTCGTGTAAAAGTAATTG
AAAGAGATTTGACAGAGCCTGATCGAATTGTTGGAGAACTCCTACAACCGGGTGGTTACCTCAAATTGCAGGAGTTGGGATTGGAAGGTAAA
GTTCCTAAATGATGTGATTTGTGTCAATTCTGGTTAATTTACTGTCTTGAAGAGAAAAAAAGGAAAGTCTTACATTTTTAGCTTCTCGTCTGTCT
CTTTTTCGGCATTTCTCATATTTGTATTTTGGTCTACTGCAGATTGTGTGGAGAAAATTGATGCTCAACGAGTGTTTGGGTATGCCCTTTTCAAG
GATGGAAAGAGTACACGTCTTTCTTATCCCCTGG
SQE_spg55_seq1-2
TTGGGGTTCGTTGCAGTTTTCTGTTTTTTCATTCAGAGAAATGGTGGCTGCAGCAAAAGTAAAATTGATGATTCAGCAACTAGCACCACTACA
ACTACGGTGTACAGTGGAAAGTCCCGATCAAAGGATGGAAACGACGACGTTGATATCATCATCGTCGGTGCCGGAGTTGCCGGTGCTGCTCT
TGCTCACACTCTTGGCAAGGTATTGTTTCTTTCTTTGTAATCCTTTTTTTTAAAGTTATTGTTCTACTTTGGTCCTTTAACTATTTTTATGTACATAT
GTGAGCAAACCGTAATTGTGTAGTGAGAAGTAATTAGACTGATTGATTTACGTATCTGAAGACATAGTTGATGTGGTTATTCATAAAAAACTT
GAACTTGATTGTTAATTTGATGAATCAGAGAAATGAAAGATGCATTGATGATGAAAGCGTTTGGTGTGACTGCAATAGTTTTATTGGAGTTCT
TTGTTGACAATTCTCTATAAAAAAAATTATTTTTTTGTGAGATTTATGATTATACATTTGGGACTACAGGAAGGGCGTCGTGTAAAAGTAATTG
AAAGAGATTTGACAGAGCCTGATCGAATTGTTGGAGAACTCCTACAACCGGGTGGTTACCTCAAATTGCAGGAGTTGGGATTGGAAGGTAAA
GTTCCTAAATGATGTGATTTGTGTCAATTCTGGTTAATTTACTGTCTTGAAGAGAAAAAAAGGAAAGTCTTACATTTTTAGCTTCTCGTCTGTCT
CTTTTTCGGCATTTCTCATATTTGTATTTTGGTCTACTGCAGATTGTGTGGAGAAAATTGATGCTCAACGAGTGTTTGGGTATGCCCTTTTCAAG
GATGGAAAGAGTACACGTCTTTCTTATCCCCTGG
SQE_spg74_seq2
TTGGGGTTCGTTGCAGTTTTCTGTTTTTTCATTCAGAGAAATGGTGGCTGCAGCAAAAGTAAAATTGATGATTCAGCAACTAGCACCACTACA
ACTACGGTGTACAGTGGAAAGTCCCGATCAAAGGATGGAAACGACGACGTTGATATCATCATCGTCGGTGCCGGAGTTGCCGGTGCTGCTCT
TGCTCACACTCTTGGCAAGGTATTGTTTCTTTCTTTGTAATCCTTTTTTTTAAAGTTATTGTTCTACTTTGGTCCTTTAACTATTTTTATGTACATAT

GTGAGCAAACCGTAATTGTGTAGTGAGAAGTAATTAGACTGATTGATTTACGTATCTGAAGACATAGTTGATGTGGTTATTCATAAAAAACTT
GAACTTGATTGTTAATTTGATGAAACCAGAGAAATGAAAGATGCATTGATGATGAAAGCTTTTGGTGTGACTGCAATTTTTTTTATTGGAGTTC
TTTGTTGACAATTCTCTATGAAAAAAAATCTTTTTTGTGAGATTTATGATTATACATTTGGGACTACAGGAAGGGCGTCGTGTAAAAGTAATTG
AAAGAGATTTGACAGAGCCTGATCGAATTGTTGGAGAACTCCTACAACCGGGTGGTTACCTCAAATTGCAGGAGTTGGGATTGGAAGGTAAA
GTTCCTAAGTGATGTGATTTGTGTCAATTCTGGTTAATTTACTCTCTTGAAGAGAAAAAAAAGGAAGTTAGTCTTACATTTTTAGCTTCTCGTCT
GTCTCTTTTTCGGCATTTCTCATATTTGTATTTTGGTCTACTGCAGATTGTGTGGAGAAAATTGATGCTCAACGAGTGTTTGGGTATGCCCTTTT
CAAGGATGGAAAGAGTACACGTCTTTCTTATCCCCTGG
SQE_spg74_seq2-2
TTGGGGTTCGTTGCAGTTTTCTGTTTTTTCATTCAGAGAAATGGTGGCTGCAGCAAAAGTAAAATTGATGATTCAGCAACTAGCACCACTACA
ACTACGGTGTACAGTGGAAAGTCCCGATCAAAGGATGGAAACGACGACGTTGATATCATCATCGTCGGTGCCGGAGTTGCCGGTGCTGCTCT
TGCTCACACTCTTGGCAAGGTATTGTTTCTTTCTTTGTAATCCTTTTTTTTAAAGTTATTGTTCTACTTTGGTCCTTTAACTATTTTTATGTACATAT
GTGAGCAAACCGTAATTGTGTAGTGAGAAGTAATTAGACTGATTGATTTACGTATCTGAAGACATAGTTGATGTGGTTATTCATAAAAAACTT
GAACTTGATTGTTAATTTGATGAAACCAGAGAAATGAAAGATGCATTGATGATGAAAGCTTTTGGTGTGACTGCAATTTTTTTTATTGGAGTTC
TTTGTTGACAATTCTCTATGAAAAAAAATCTTTTTTGTGAGATTTATGATTATACATTTGGGACTACAGGAAGGGCGTCGTGTAAAAGTAATTG
AAAGAGATTTGACAGAGCCTGATCGAATTGTTGGAGAACTCCTACAACCGGGTGGTTACCTCAAATTGCAGGAGTTGGGATTGGAAGGTAAA
GTTCCTAAGTGATGTGATTTGTGTCAATTCTGGTTAATTTACTCTCTTGAAGAGAAAAAAAAGGAAGTTAGTCTTACATTTTTAGCTTCTCGTCT
GTCTCTTTTTCGGCATTTCTCATATTTGTATTTTGGTCTACTGCAGATTGTGTGGAGAAAATTGATGCTCAACGAGTGTTTGGGTATGCCCTTTT
CAAGGATGGAAAGAGTACACGTCTTTCTTATCCCCTGG
SQE_spl16_seq1
TTGGGGTTCGTTGCAGTTTTCTGTTTTTTCATTCAGAGAAATGGTGGCTGCAGCAAAAGTAAAATTGATGATTCAGCAACTAGCACCACTACA
ACTACGGTGTACAGTGGAAAGTCCCGATCAAAGGATGGAAACGACGACGTTGATATCATCATCGTCGGAGCCGGAGTTGCCGGTGCTGCTCT
TGCTCACACTCTTGGCAAGGTATTGTTTCTTTCTTTGTAATCCTTTTTTTTAAAGTTATTGTTCTACCTTGGTCCTTTAACTATTTTTATGTACATAT
GTGAGAAAACCGTAATTGTGTAGTGAGAAGTAATTAGACTGATTGATTTACGTATCTGAAGACATAGTTGATGTGGTTATTCATAAAAAACTT
GAACTTGATTGTTAATTTGATGAAACCAGAGAAATGAAAGATGCATTGATGATGAAAGCGTTTGGTGTGACTGCAATTTTTTTTATTGGAGTT
CTTTGTTGACAATTCTCTATGGAAAAAATTCTTTTTTGTGAGATTTATGATTATACATTTGGGACTACAGGAAGGGCGTCGTGTAAAAGTAATT
GAAAGAGATTTGACAGAGCCTGATCGAATTGTTGGAGAACTCCTACAGCCGGGTGGTTACCTCAAATTGCAGGAGTTGGGATTGGAAGGTA
AAGTTCCTAAGTGATGTGATTTGTGTCAATTCTGGTTAATTTACTCTCTTGAAGAGAAAAAAAAGGAAGTTAGTCTTACATTTTTAGCTTCTCGT
CTGTCTCTTTTTCGGCATTTCTCATATTTGTATTTTGGTCTACTGCAGATTGTGTGGAGAAAATTGATGCTCAACGAGTGTTTGGGTATGCCCTT
TTCAAGGATGGAAAGAGTACACGTCTTTCTTATCCCCTGG
SQE_spl16_seq1-2
TTGGGGTTCGTTGCAGTTTTCTGTTTTTTCATTCAGAGAAATGGTGGCTGCAGCAAAAGTAAAATTGATGATTCAGCAACTAGCACCACTACA
ACTACGGTGTACAGTGGAAAGTCCCGATCAAAGGATGGAAACGACGACGTTGATATCATCATCGTCGGAGCCGGAGTTGCCGGTGCTGCTCT
TGCTCACACTCTTGGCAAGGTATTGTTTCTTTCTTTGTAATCCTTTTTTTTAAAGTTATTGTTCTACCTTGGTCCTTTAACTATTTTTATGTACATAT
GTGAGAAAACCGTAATTGTGTAGTGAGAAGTAATTAGACTGATTGATTTACGTATCTGAAGACATAGTTGATGTGGTTATTCATAAAAAACTT
GAACTTGATTGTTAATTTGATGAAACCAGAGAAATGAAAGATGCATTGATGATGAAAGCGTTTGGTGTGACTGCAATTTTTTTTATTGGAGTT
CTTTGTTGACAATTCTCTATGGAAAAAATTCTTTTTTGTGAGATTTATGATTATACATTTGGGACTACAGGAAGGGCGTCGTGTAAAAGTAATT
GAAAGAGATTTGACAGAGCCTGATCGAATTGTTGGAGAACTCCTACAGCCGGGTGGTTACCTCAAATTGCAGGAGTTGGGATTGGAAGGTA
AAGTTCCTAAGTGATGTGATTTGTGTCAATTCTGGTTAATTTACTCTCTTGAAGAGAAAAAAAAGGAAGTTAGTCTTACATTTTTAGCTTCTCGT
CTGTCTCTTTTTCGGCATTTCTCATATTTGTATTTTGGTCTACTGCAGATTGTGTGGAGAAAATTGATGCTCAACGAGTGTTTGGGTATGCCCTT
TTCAAGGATGGAAAGAGTACACGTCTTTCTTATCCCCTGG
SQE_spl81_seq2
TTGGGGTTCGTTGCAGTTTTCTGTTTTTTCATTCAGAGAAATGGTGGCTGCAGCAAAAGTAAAATTGATGATTCAGCAACTAGCACCACTACA
ACTACGATGTACAGTGGAAAGTCCCGATCAAAGGATGGAAACGACGACGTTGATATCATCATCGTCGGTGCCGGAGTTGCCGGTGCTGCTCT
TGCTCACACTCTTGGCAAGGTTTTATTTCTTTCTTTGTAATCCGTTTTTTTAAAGTTATTATTCTACTTTGGTCCTTCAACTATTTTTTATGTACATA
TGTGAGCAAACCGTAATTGTGTAGTGAGAAGTAATTAGATTGATTGATTTACGTATCTGAAGGCATAGTTGATGTGGTTATTCATAAAAAACT
TGAACTTGATTGTTAATTTGATGAATCAGAGAAATGAAAGATGCATTGATGATGAAAGCGTTTGGTGTGACTGCAATAATTTTATTGGAGTTC
TTTGTTGACAATTCTCTATAAATTTTTTTTATTTTTTTGTGAGATTTATGATTATACATTTGGGACTACAGGAAGGGCGTCGTGTAAAAGTAATT
GAAAGAGATTTGACAGAGCCTGATCGAATTGTTGGAGAACTCCTACAACCGGGTGGTTACCTCAAATTGCAGGAGTTGGGATTGGAAGGTA
AAGTTCCTAAGTGATGTGATTTGTGTCAATTCTGGTTAATTTACTCTCTTGAAGAGAAAAAAAAGGAAGTTAGTCTTACATTTTTTGCTTCTCGT
CTGTCTATTTTTTGGTATTTCTCATATTTGTATTTTGGTCTACTGCAGATTGTGTGGAGAAAATTGATGCTCAACGAGTGTTTGGGTATGCCCTT
TTCAAGGATGGAAAGAGTACACGTCTTTCTTATCCCCTGG
SQE_spl81_seq3
TTGGGGTTCGTTGCAGTTTTCTGTTTTTTCATTCAGAGAAATGGTGGCTGCAGCAAAAGTAAAATTGATGATTCAGCAACTAGCACCACTACA
ACTACGGTGTACAGTGGAAAGTCCGGATCAAAGGATGGAAACGACGACGTTGATATCATCATCGTTGGTGCCGGAGTTGCCGGTGCTGCTCT
TGCTCACACTCTTGGCAAGGTATTGTTTCTTTCTTTGTAATCCTTTTTTTTAAAGTTATTGTTCTACTTTGATCCTTTAACTATTTTTATGTACATAT
GTGAGCAAACCGTAATTGTGAAGTGAGAAGTAATTAGACTGATTGATTTACGTATCTGAAGACATAGTTGATGTGGTTATTCATAAAAGACTT
GAACTTGATTGTTAATTTGATGAAACCAGAAAAATGAAAGATGCATTGATGATGAAAGCGTTTGGTGTGACTGCAAATTTTTTTATTGGAGTT
CTTTGTTGACAATTCTCTATGAAAAAAAATTCTTTTTTATGAGATTTATGATTATACATTTGGGACTACAGGAAGGGCGTCGTGTAAAAGTAAT

TGAAAGAGATTTGACAGAGCCTGATCGAATTGTTGGAGAACTCCTACAACCGGGTGGTTACCTCAAATTGCAGGAGTTGGGATTGGAAGGTA
AAGTTCCTAAGTGATGTGATTTGTGTCAATTCTGGTTAATTTACTCTCTTGAAGAGAAAAAAAAGGAAGTTAGTCTTACACTTTTAGCTTCTCG
TCTGTCTCTTTTTTGGCATTTCTCATATTTGTATTTTGGTCTACTGCAGATTGTGTGGAGAAAATTGATGCTCAACGAGTGTTTGGGTATGCCCT
TTTCAAGGATGGTAAGAGTACACGTCTTTCTTATCCCCTGG
SQE_sto40_seq1
TTGGGGTTCGTTGCAGTTTTCTGTTTTTTCGTTCAGAGAAATGGTGGCCGCAGCAAAAGTAAAATTGATGACTCGGCAACTAGCACCACTACA
ACTACGGTGTACAGTGGAGAGTCTAGATCAAAGGATGGAAACGACGAAATTGATATCATCATCGTCGGTGCCGGTGTTGCCGGTGCTGCTCT
TGCTCACACTCTTGGCAAGGTATTGTTTCTTTCTTTGTAATCCTTTTTTTAAAAGTTATTATTCTACTTTGGTCCTTTAACTATTTTTATGTACATAT
GTGAGCAAACCGTAATTGTGTAGTGAGAAGTAATTAGACTGATTGATTTACGTATCTGAAGGCATACTTGATTGTTAATTTGATGAAATCAGA
GAAATGAAAGATGCATTGATGATGAAACCGTTTAGTGTGACTGCAAATAATTTTATTGGAGTTCTTTGTTGACAATTCTCTATAAAAATATTCT
TTTTTATGAGACTTATGATTATACATTTGGGACTACAGGAAGGGCGTCGTGTAAAAGTAATTGAAAGAGATTTGACAGAGCCTGATCGAATTG
TTGGAGAACTACTACAACCGGGTGGTTACCTCAAATTGCAGGAGTTGGGATTGGAAGGTAAAGTTCCTAAATGATGTGATTTGTGTCAATTCT
GGTTAATTTACTCTCTTGAAGAGAAAAAAAGGAAGTTAGTCTTACATTTTTAGCTTCTCGTCTGTCTCTTTTTCGGAATTTCTCATATTTGTATTT
TGGTCTACTGCAGATTGTGTGGAGAAAATTGATGCTCAACGAGTGTTTGGGTATGCCCTTTTCAAGGATGGAAAGAGTACACGTCTTTCTTAT
CCCCTGG
SQE_sto40_seq2
TTGGGGTTCGTTGCAGTTTTCTGTTTTTTCATTCAGAGAAATGGTGGCTGCAGCAAAAGTAAAATTGATGATTCAGCAACTAGCACCACTACA
ACTACGGTGTACAGTGGAAAGTCCCGATCAAAGGATGGAAACGACGACGTTGATATCATCATCGTTGGTGCCGGAGTTGCCGGTGCTGCTCT
TGCTCACACTCTTGGCAAGGTATTGTTTCTTTCTTTGTAATCCTTTTTTTTAAAGTTATTGTTCTACTTTGATCCTTTAACTATTTTTATGTACATAT
GTGAGCAAACCGTAATTGTGAAGTGAGAAGTAATTAGACTGATTGATTTACGTATCTGAAGACATAGTTGATGTGGTTATTCATAAAAGACTT
GAACTTGATTGTTAATTTGATGAAACCAGAGAAATGAAAGATGCATTGATGATGAAAGCGTTTGGTGTGACTGCAAATTTTTTTATTGGAGTT
CTTTGTTGACAATTCTCTATGAAAAAAAAATTCTTTTTTGTGAGATTTATGATTATACATTTGGGACTACAGGAAGGGCGTCGTGTAAAAGTAAT
TGAAAGAGATTTGACAGAGCCTGATCGAATTGTTGGAGAACTCCTACAACCGGGTGGTTACCTCAAATTGCAGGAGTTGGGATTGGAAGGTA
AAGTTCCTAAGTGATGTGATTTGTGTCAATTCTGGTTAATTTACTCTCTTGAAGAGAAAAAAAAGGAAGTTAGTCTTACACTTTTAGCTTCTCG
TCTGTCTCTTTTTTGGCATTTCTCATATTTGTATTTTGGTCTACTGCAGATTGTGTGGAGAAAATTGATGCTCAACGAGTGTTTGGGTATGCCCT
TTTCAAGGATGGAAAGAGTACACGTCTTTCTTATCCCCTGG
SQE_sto61_seq2
TTGGGGTTCGTTGCAGTTTTCTGTTTTTTCATTCAGAGAAATGGTGGCTGCAGCAAAAGTAAAATTGATGATTCAGCAACTAGCACCACTACA
ACTACGGTGTACAGTGGAAAGTCCCGATCAAAGGATGGAAACGACGACGTTGATATCATCATCGTTGGTGCCGGAGTTGCCGGTGCTGCTCT
TGCTCACACTCTTGGCAAGGTATTGTTTCTTTCTTTGTAATCCTTTTTTTTAAAGTTATTGTTCTACTTTGATCCTTTAACTATTTTTATGTACATAT
GTGAGCAAACCGTAATTGTGAAGTGAGAAGTAATTAGACTGATTGATTTACGTATCTGAAGACATAGTTGATGTGGTTATTCATAAAAGACTT
GAACTTGATTGTTAATTTGATGAAACCAGAGAAATGAAAGATGCATTGATGATGAAAGCGTTTGGTGTGACTGCAAATTTTTTTATTGGAGTT
CTTTGTTGACAATTCTCTATGAAAAAAAAATTCTTTTTTGTGAGATTTATGATTATACATTTGGGACTACAGGAAGGGCGTCGTGTAAAAGTAAT
TGAAAGAGATTTGACAGAGCCTGATCGAATTGTTGGAGAACTCCTACAACCGGGTGGTTACCTCAAATTGCAGGAGTTGGGATTGGAAGGTA
AAGTTCCTAAGTGATGTGATTTGTGTCAATTCTGGTTAATTTACTCTCTTGAAGAGAAAAAAAAGGAAGTTAGTCTTACACTTTTAGCTTCTCG
TCTGTCTCTTTTTTGGCATTTCTCATATTTGTATTTTGGTCTACTGCAGATTGTGTGGAGAAAATTGATGCTCAACGAGTGTTTGGGTATGCCCT
TTTCAAGGATGGAAAGAGTACACGTCTTTCTTATCCCCTGG
SQE_sto61_seq3
TTGGGGTTCGTTGCAGTTTTCTGTTTTTTCGTTCAGAGAAATGGTGGCCGCAGCAAAAGTAAAATTGATGACTCGGCAACTAGCACCACTACA
ACTACGGTGTACAGTGGAGAGTCTAGATCAAAGGATGGAAACGACGAAATTGATATCATCATCGTCGGTGCCGGTGTTGCCGGTGCTGCTCT
TGCTCACACTCTTGGCAAGGTATTGTTTCTTTCTTTGTAATCCTTTTTTTAAAAGTTATTATTCTACTTTGGTCCTTTAACTATTTTTATGTACATAT
GTGAGCAAGCCGTAATTGTGTAGTGAGAAGTAATTAGACTGATTGATTTACGTATCTGAAGGCATACTTGATTGTTAATTTGATGAAATCAGA
GAAATGAAAGATGCATTGATGATGAAACCGTTTAGTGTGACTGCAAATAATTTTATTGGAGTTCTTTGTTGACAATTCTCTATAAAAATATTCT
TTTTTATGAGACTTATGATTATACATTTGGGACTACAGGAAGGGCGTCGTGTAAAAGTAATTGAAAGAGATTTGACAGAGCCTGATCGAATTG
TTGGAGAACTACTACAACCGGGTGGTTACCTCAAATTGCAGGAGTTGGGATTGGAAGGTAAAGTTCCTAAATGATGTGATTTGTGTCAATTCT
GGTTAATTTACTCTCTTGAAGAGAAAAAAAGGAAGTTAGTCTTACATTTTTAGCTTCTCGTCTGTCTCTTTTTCGGAATTTCTCATATTTGTATTT
TGGTCTACTGCAGATTGTGTGGAGAAAATTGATGCTCAACGAGTGTTTGGGTATGCCCTTTTCAAGGATGGAAAGAGTACACGTCTTTCTTAT
CCCCTGG
SQE_chc80-1_seq1
TTGGGGTTCGTTGCAGTTTTCTGTTTTTTCATTCAGAGAAATGGTGGCTGCAGCAAAAGTAAAATTGATGACTCAGCAACTAGCACCACTACA
ACTACGGTGTACAGTGGAAAGTCCCGATCAAAGGATGGAAACGACGACGTTGATATCATCATCGTCGGTGCCGGAGTTGCCGGTGCTGCTCT
TGCTCACACTCTTGGCAAGGTATTGTTTCTTTCTTTGTAATCCTTTTTTTTAAAGTTATTGTTCTACTTTGGTCCTTTAACTATTTTTATGCACATAT
GTGAGCAAACTGTAATTGTGTAGTGAGAAGTAATTAGACTGATTGATTTACGTATCTGAAGACATAGTTGATGTGGTTATTCATAAAAAACTT
GAACTTGATTGTTAATTTGATGAAACCAGAGAAATGAAAGATGCATTGATGATGAAAGCGTTTGGTGTGACTGCAGATTTTTTTATTGGAGTT
CTTTGTTGACAATTCTCTATGAAAAAAAATTCTTTTTTGTGAGATTTATGATTATACATTTGGGACTACAGGAAGGGCGTCGTGTAAAAGTAATT
GAAAGAGATTTGACAGAGCCCGATCGAATTGTTGGAGAACTCCTACAACCGGGTGGTTACCTCAAATTGCAGGAGTTGGGATTGGAAGGTA
AAGTTCCTAAGTGATGTGATTTGTGTCAATTCTGGTTAATTTACTCTCTTGAAGAGAAAAAAAAGGAAGTTAGTCTTACATTTTTAGCTTCTCGT

CTGTCTCTTTTTCGGCATTTCTCATGTTTGTATTTTGGTCTACTGCAGATTGTGTGGAGAAAATTGATGCTCAACGAGTGTTTGGGTATGCCCTT
TTCAAGGATGGAAAGAGTACACGTCTTTCTTATCCCCTGG
SQE_chc80-1_seq2
TTGGGGTTCGTTGCAGTTTTCTGTTTTTTCATTCAGAGAAATGGTGGCTGCAGCAAAAGTAAAATTGATGACTCAGCAACTAGCACCACTACA
ACTACGGTGTACAGTGGAAAGTCCCGATCAAAGGATGGAAATGACGACGTTGATATCATCATCGTCGGTGCCGGAGTTGCCGGTGCTGCTCT
TGCTCACACTCTTGGCAAGGTATTGTTTCTTTCTTTGTAATCCTTTTTTTTAAAGTTATTGTTCTACTTTGGTCCTTTAATTATTTTTATGTACATAT
GTGAGCAAACCGTAATTGTGTAGTGAGAAGTAATTAGACTGATTGATTTACGTATCTGAAGACAAAGTTGATGTGGTTATTCGTAAAAAACTT
GAACTTGATTGTTAATTTGATGAAACCAGAGAAATGAAAGATGCATTGATGATGAAAGCGTTTGGTGTGACTGCAAATTTTTTTATTGGAGTT
CTTTGTTGACAATTCTCTATAAAAAAAAATTATTTTTTTGTGAGATTTATGATTATACATTTGGGACTACAGGAAGGGCGTCGTGTAAAAGTAAT
TGAAAGAGATTTGACAGAGCCTGATCGAATTGTTGGAGAACTCCTACAACCGGGTGGTTACCTCAAATTGCAGGAGTTGGGATTGGAAGGTA
AAGTTCCTAAGTGATGTGATTTGTGTCAATTCTGGTTAATTTACTCTCTTGAAGAGAAAAAAAAGGAAGTTAGTCTTACATTTTTAGCTTCTCGT
CTGTCTCTTTTTCGGCATTTCTCATATTTGTATTTTGGTCTACTGCAGATTGTGTGGAGAAAATTGATGCTCAACGAGTGTTTGGGTATGCCCTT
TTCAAGGATGGAAAGAGTACACGTCTTTCTTATCCCCTGG
SQE_phuDH_seq1
TTGGGGTTCGTTGCAGTTTTCTGTTTTTTCATTCAGAGAAATGGTGGCTGCAGCAAAAGTAAAATTGATGACTCAGCAACTAGCACCACTACA
ACTACGGTGTACAGTGGAAAGTCCCGATCAAAGGATGGAAACGACGACGTTGATATCATCATCGTCGGTGCCGGAGTTGCCGGTGCTGCTCT
TGCTCACACTCTTGGCAAGGTATTGTTTCTTTCTTTGTAATCCGTTTTTTAAAAGTTATTATTCTACTTTGGTCCTTCAACTATTTTTATGTACATA
TGTGAGCAAACCGTAATTGTGTAGTGAGAAGTAATTAGATTGATTGATTTACGTATCTGAAGGCATAGTTGATGTGGTTATTCATAAAAAACT
TGAACTTGATTGTTAATTTGATGAAATCAGAGAAATGAAAGATGCATTGATGATGAAAGCGTTTGGTGTGACTGCAATTTTTTTTATTGGAGT
TCTTTGTTGACAATTCTCTATGAAAAAAAATTCTTTTTTGTGAGATTTATGATTATACATTTGGGACTACAGGAAGGGCGTCGTGTACAAGTAAT
TGAAAGAGATTTGACAGAGCCTGATCGAATTGTTGGAGAACTCCTACAACCGGGTGGTTACCTCAAATTGCAGGAGTTGGGATTGGAAGGTA
AAGTTCCTAAGTGATGTGATTTGTGTCAATTCTGGTTAATTTACTCTCTTGAAGAGAAAAAAAAGGAAGTTAGTCTTGCACTTTTAGCTTCTCG
TCTGTCTCTTTTTCGGCATTTCTCATATTTGTATTTTGGTCTACTGCAGATTGTGTGGAGAAAATTGATGCTCAACGAGTGTTTGGGTATGCCCT
TTTCAAGGATGGAAAGAGTACACGTCTTTCTTATCCCCTGG
SQE_phuDH_seq1-2
TTGGGGTTCGTTGCAGTTTTCTGTTTTTTCATTCAGAGAAATGGTGGCTGCAGCAAAAGTAAAATTGATGACTCAGCAACTAGCACCACTACA
ACTACGGTGTACAGTGGAAAGTCCCGATCAAAGGATGGAAACGACGACGTTGATATCATCATCGTCGGTGCCGGAGTTGCCGGTGCTGCTCT
TGCTCACACTCTTGGCAAGGTATTGTTTCTTTCTTTGTAATCCGTTTTTTAAAAGTTATTATTCTACTTTGGTCCTTCAACTATTTTTATGTACATA
TGTGAGCAAACCGTAATTGTGTAGTGAGAAGTAATTAGATTGATTGATTTACGTATCTGAAGGCATAGTTGATGTGGTTATTCATAAAAAACT
TGAACTTGATTGTTAATTTGATGAAATCAGAGAAATGAAAGATGCATTGATGATGAAAGCGTTTGGTGTGACTGCAATTTTTTTTATTGGAGT
TCTTTGTTGACAATTCTCTATGAAAAAAAATTCTTTTTTGTGAGATTTATGATTATACATTTGGGACTACAGGAAGGGCGTCGTGTACAAGTAAT
TGAAAGAGATTTGACAGAGCCTGATCGAATTGTTGGAGAACTCCTACAACCGGGTGGTTACCTCAAATTGCAGGAGTTGGGATTGGAAGGTA
AAGTTCCTAAGTGATGTGATTTGTGTCAATTCTGGTTAATTTACTCTCTTGAAGAGAAAAAAAAGGAAGTTAGTCTTGCACTTTTAGCTTCTCG
TCTGTCTCTTTTTCGGCATTTCTCATATTTGTATTTTGGTCTACTGCAGATTGTGTGGAGAAAATTGATGCTCAACGAGTGTTTGGGTATGCCCT
TTTCAAGGATGGAAAGAGTACACGTCTTTCTTATCCCCTGG
SQE_phuDM_seq1
TTGGGGTTCGTTGCAGTTTTCTGTTTTTTCATTCAGAGAAATGGTGGCTGCAGCAAAAGTAAAATTGATGACTCAGCAACTAGCACCACTACA
ACTACGGTGTACAGTGGAAAGTCCCGATCAAAGGATGGAAACGACGACGTTGATATCATCATCGTCGGTGCCGGAGTTGCCGGTGCTGCTCT
TGCTCACACTCTTGGCAAGGTATTGTTTCTTTCTTTGTAATCCGTTTTTTAAAAGTTATTATTCTACTTTGGTCCTTCAACTATTTTTATGTACATA
TGTGAGCAAACCGTAATTGTGTAGTGAGAAGTAATTAGATTGATTGATTTACGTATCTGAAGGCATAGTTGATGTGGTTATTCATAAAAAACT
TGAACTTGATTGTTAATTTGATGAAATCAGAGAAATGAAAGATGCATTGATGATGAAAGCGTTTGGTGTGACTGCAATTTTTTTTATTGGAGT
TCTTTGTTGACAATTCTCTATGAAAAAAAATTCTTTTTTGTGAGATTTATGATTATACATTTGGGACTACAGGAAGGGCGTCGTGTACAAGTAAT
TGAAAGAGATTTGACAGAGCCTGATCGAATTGTTGGAGAACTCCTACAACCGGGTGGTTACCTCAAATTGCAGGAGTTGGGATTGGAAGGTA
AAGTTCCTAAGTGATGTGATTTGTGTCAATTCTGGTTAATTTACTCTCTTGAAGAGAAAAAAAAGGAAGTTAGTCTTGCACTTTTAGCTTCTCG
TCTGTCTCTTTTTCGGCATTTCTCATATTTGTATTTTGGTCTACTGCAGATTGTGTGGAGAAAATTGATGCTCAACGAGTGTTTGGGTATGCCCT
TTTCAAGGATGGAAAGAGTACACGTCTTTCTTATCCCCTGG
SQE_phuDM_seq1-2
TTGGGGTTCGTTGCAGTTTTCTGTTTTTTCATTCAGAGAAATGGTGGCTGCAGCAAAAGTAAAATTGATGACTCAGCAACTAGCACCACTACA
ACTACGGTGTACAGTGGAAAGTCCCGATCAAAGGATGGAAACGACGACGTTGATATCATCATCGTCGGTGCCGGAGTTGCCGGTGCTGCTCT
TGCTCACACTCTTGGCAAGGTATTGTTTCTTTCTTTGTAATCCGTTTTTTAAAAGTTATTATTCTACTTTGGTCCTTCAACTATTTTTATGTACATA
TGTGAGCAAACCGTAATTGTGTAGTGAGAAGTAATTAGATTGATTGATTTACGTATCTGAAGGCATAGTTGATGTGGTTATTCATAAAAAACT
TGAACTTGATTGTTAATTTGATGAAATCAGAGAAATGAAAGATGCATTGATGATGAAAGCGTTTGGTGTGACTGCAATTTTTTTTATTGGAGT
TCTTTGTTGACAATTCTCTATGAAAAAAAATTCTTTTTTGTGAGATTTATGATTATACATTTGGGACTACAGGAAGGGCGTCGTGTACAAGTAAT
TGAAAGAGATTTGACAGAGCCTGATCGAATTGTTGGAGAACTCCTACAACCGGGTGGTTACCTCAAATTGCAGGAGTTGGGATTGGAAGGTA
AAGTTCCTAAGTGATGTGATTTGTGTCAATTCTGGTTAATTTACTCTCTTGAAGAGAAAAAAAAGGAAGTTAGTCTTGCACTTTTAGCTTCTCG
TCTGTCTCTTTTTCGGCATTTCTCATATTTGTATTTTGGTCTACTGCAGATTGTGTGGAGAAAATTGATGCTCAACGAGTGTTTGGGTATGCCCT
TTTCAAGGATGGAAAGAGTACACGTCTTTCTTATCCCCTGG

1.4 Solanidine galactosyltransferase (*SGT1*) sequences

28

SGT1_cmm7_seq1

CCTTGGACAGTAGATATTGCTGATGAGCTTCACATCCCTCGTATTTTGTACAATTTGTCTGCTTACATGTGCTACAGCATTATGCACAACCTTAA
GGTTTACAGACCTCACAAGCAGCCTAATCTAGACGAATCTCAAAGTTTCGTGGTTCCTGGTTTACCTGATGAGATAAAGTTCAAGTTATCCCAA
CTGACAGATGATCTGAGAAGGCCGGATGACCAAAAGACTGTTTTTGACGAATTGCTCGAACAAGTTGCAGATTCGGAGGAACGAAGCTATG
GCATTGTTCATGATACATTTTATGAGCTAGAACCTGCATATGTCGACTACTACCAGAAATTAAAGAAACCAAAATGCTGGCATTTTGGTCCGCT
CTCTCATTTTGCATCCAAAATCCGTAGTAAGGAACTAATTTCTGAGCATAACAACAATGAGATCGTTGTAGATTGGTTGAATGCACAGAAACCT
AAATCGGTTCTCTATGTATCTTTCGGAAGCATGGCTAGATTTCCTGAGAGCCAACTGAATGAAATAGCCGAAGCTCTGGATGCTTCAAATGTT
CCTTTCATTTTTGTATTGAGGCCTAATGAAGAAACGGCGTCGTGGTTGCCAGTTGGTAATTTAGAGGACAAGACTAAAAAGGGTTTGTACATC
AAAGGGTGGGTTCCACAGCTTACGATCATGGAACATTCAGCAACAGGCGGGTTCATGACACATTGTGGTACTAATTCGGTTCTGGAAGCCAT
CACTTTTGGCGTGCCAATGATAACATGGCCACTTTATGCTGATCAATTCTACAACGAGAAGGTAGTCGAGGTTAGGGGATTGGGA

SGT1_cmm7_seq1-2

CCTTGGACAGTAGATATTGCTGATGAGCTTCACATCCCTCGTATTTTGTACAATTTGTCTGCTTACATGTGCTACAGCATTATGCACAACCTTAA
GGTTTACAGACCTCACAAGCAGCCTAATCTAGACGAATCTCAAAGTTTCGTGGTTCCTGGTTTACCTGATGAGATAAAGTTCAAGTTATCCCAA
CTGACAGATGATCTGAGAAGGCCGGATGACCAAAAGACTGTTTTTGACGAATTGCTCGAACAAGTTGCAGATTCGGAGGAACGAAGCTATG
GCATTGTTCATGATACATTTTATGAGCTAGAACCTGCATATGTCGACTACTACCAGAAATTAAAGAAACCAAAATGCTGGCATTTTGGTCCGCT
CTCTCATTTTGCATCCAAAATCCGTAGTAAGGAACTAATTTCTGAGCATAACAACAATGAGATCGTTGTAGATTGGTTGAATGCACAGAAACCT
AAATCGGTTCTCTATGTATCTTTCGGAAGCATGGCTAGATTTCCTGAGAGCCAACTGAATGAAATAGCCGAAGCTCTGGATGCTTCAAATGTT
CCTTTCATTTTTGTATTGAGGCCTAATGAAGAAACGGCGTCGTGGTTGCCAGTTGGTAATTTAGAGGACAAGACTAAAAAGGGTTTGTACATC
AAAGGGTGGGTTCCACAGCTTACGATCATGGAACATTCAGCAACAGGCGGGTTCATGACACATTGTGGTACTAATTCGGTTCTGGAAGCCAT
CACTTTTGGCGTGCCAATGATAACATGGCCACTTTATGCTGATCAATTCTACAACGAGAAGGTAGTCGAGGTTAGGGGATTGGGA

SGT1_cmm26_seq1

CCTTGGACAGTAGATATTGCTGATGAGCTTCACATCCCTCGTATTTTGTACAATTTGTCTGCTTACATGTGCTACAGCATTATGCACAACCTTAA
GGTTTACAGACCTCACAAGCAGCCTAATCTAGACGAATCTCAAAGTTTCGTGGTTCCTGGTTTACCTGATGAGATAAAGTTCAAGTTATCCCAA
CTGACAGATGATCTGAGAAGGCCGGATGACCAAAAGACTGTTTTTGACGAATTGCTCGAACAAGTTGCAGATTCGGAGGAACGAAGCTATG
GCATTGTTCATGATACATTTTATGAGCTAGAACCTGCATATGTCGACTACTACCAGAAATTAAAGAAACCAAAATGCTGGCATTTTGGTCCGCT
CTCTCATTTTGCATCCAAAATCCGTAGTAAGGAACTAATTTCTGAGCATAACAACAATGAGATCGTTGTAGATTGGTTGAATGCACAGAAACCT
AAATCGGTTCTCTATGTATCTTTCGGAAGCATGGCTAGATTTCCTGAGAGCCAACTGAATGAAATAGCCGAAGCTCTGGATGCTTCAAATGTT
CCTTTCATTTTTGTATTGAGGCCTAATGAAGAAACGGCGTCGTGGTTGCCAGTTGGTAATTTAGAGGACAAGACTAAAAAGGGTTTGTACATC
AAAGGGTGGGTTCCACAGCTTACGATCATGGAACATTCAGCAACAGGCGGGTTCATGACACATTGTGGTACTAATTCGGTTCTGGAAGCCAT
CACTTTTGGCGTGCCAATGATAACATGGCCACTTTATGCTGATCAATTCTACAACGAGAAGGTAGTCGAGGTTAGGGGATTGGGA

SGT1_cmm26_seq1-2

CCTTGGACAGTAGATATTGCTGATGAGCTTCACATCCCTCGTATTTTGTACAATTTGTCTGCTTACATGTGCTACAGCATTATGCACAACCTTAA
GGTTTACAGACCTCACAAGCAGCCTAATCTAGACGAATCTCAAAGTTTCGTGGTTCCTGGTTTACCTGATGAGATAAAGTTCAAGTTATCCCAA
CTGACAGATGATCTGAGAAGGCCGGATGACCAAAAGACTGTTTTTGACGAATTGCTCGAACAAGTTGCAGATTCGGAGGAACGAAGCTATG
GCATTGTTCATGATACATTTTATGAGCTAGAACCTGCATATGTCGACTACTACCAGAAATTAAAGAAACCAAAATGCTGGCATTTTGGTCCGCT
CTCTCATTTTGCATCCAAAATCCGTAGTAAGGAACTAATTTCTGAGCATAACAACAATGAGATCGTTGTAGATTGGTTGAATGCACAGAAACCT
AAATCGGTTCTCTATGTATCTTTCGGAAGCATGGCTAGATTTCCTGAGAGCCAACTGAATGAAATAGCCGAAGCTCTGGATGCTTCAAATGTT
CCTTTCATTTTTGTATTGAGGCCTAATGAAGAAACGGCGTCGTGGTTGCCAGTTGGTAATTTAGAGGACAAGACTAAAAAGGGTTTGTACATC
AAAGGGTGGGTTCCACAGCTTACGATCATGGAACATTCAGCAACAGGCGGGTTCATGACACATTGTGGTACTAATTCGGTTCTGGAAGCCAT
CACTTTTGGCGTGCCAATGATAACATGGCCACTTTATGCTGATCAATTCTACAACGAGAAGGTAGTCGAGGTTAGGGGATTGGGA

SGT1_dms54_seq1

CCTTGGACAGTAGATATTGCTGATGAGCTGCACATCCCTCGTATTTTGTACAATTTGTCTGCTTACATGTGCTACAGCATTATGCACAACCTTAA
GGTTTACAGACCTCACAAGCAGCCTAATCTAGACGAATCTCAAAGTTTCGTGGTTCCTGGTTTACCTGATGAGATAAAGTTCAAGTTATCCCAA
CTGACAGATGATCTGAGAAAGCCGGATGACCAAAAGACTGGTTTTGACGAATTGCTCGAACAAGTTGGAGATTCGGAGGAGCGAAGCTATG
GCATTGTTCATGATACATTTTATGAGCTAGAACCTGCATATGTCGACTACTACCAGAAATTAAAGAAACCAAAATGTTGGCATTTTGGTCCGCT
CTCTCATTTTGCATCCAAAATCCGTAGTAAGGAACTAATTTCTGAGCATAACAACAATGAGATTGTTATAGATTGGTTGAATGCACAGAAACCT
AAATCGGTTCTCTATGTATCTTTCGGAAGCATGGCTAGATTTCCTGAGAGCCAACTGAATGAAATAGCCCAAGCTCTGGATGCTTCAAATGTTC
CTTTCATTTTTGTATTGAGGCCTAATGAAAAAACAACGTCGTGGTTGCCAGTTGGTAATTTAGAGGACAAGACTAAAAAGGGTTTGTACATCA
AAGGGTGGGTCCCACAGCTTACGATCATGGAACATTCAGCAACAGGCGGGTTCATGACTCATTGTGGTACTAATTCGGTTCTGGAAGCCATC
ACTTTTGGCGTGCCAATGATAACATGGCCACTTTATGCTGATCAATTCTACAACGAGAAGGTAGTCGAGGTTAGGGGATTGGGA

SGT1_dms54_seq2

CCTTGGACAGTAGATATTGCTGATGAGCTTCACATCCCTCGTATTTTGTACAATTTGTCTTCTTACATGTGCTACAGCATTAAGCACAACCTTAA
GGTTTACAGACCTCACAAGCAGCCTAATCTAGACGAATCTCAAAGTTTCGTGGTTCCTGGTTTACCTAATGAGATAAAGTTCAAGTTATCCCAA
CTGACAGATGATCTGAGAAAGCCGGATGACCAAAAGACTGTTTTTGACGAATTGCTCGAACAAGTTGGAGATTCGGAGGAACGAAGCTATG
GCATTGTTCATGATACATTTTATGAGCTAGAACCTGCATATGTCGACTATTACCAGAAATTAAAGAAACCAAAATGTTGGCATTTTGGTCCGCA
CTCTCATTTTGCATCCAAAATCCGTAGTAAGGAACTAATTTCTGAGCATAACAACAATGAGATTGTTATAGATTGGTTGAATGCACAGAAACCT

AAATCGGTTATCTATGTATCTTTCGGAAGCATGGCTAGATTTCCTGAGAGCCAACTGAATGAAATAGCCCAAGCTCTGGATGCTTCAAATGTT
CCTTTCATTTGTGTATTGAGGCCTAATGAAGAAACAATGTCGTGGTTGCCAGTTGGTAATTTAGAGGACAAGACTAAAAAGGGTTTGTACATC
AAAGGGTGGGTCCCACAACTTACGATCATGGAACATTCAGCAACAGGCGGGTTCATGACTCATTGTGGTACTAATTCGGTTCTAGAAGCCATC
ACTTTTGGCGTGCCAATGATAACATGGCCACTTTATGCTGATCAATTCTACAACGAGAAGGTAGTCGAGGTTAGGGGATTGGGA
SGT1_dms78_seq3
CCTTGGACAGTAGATATTGCTGATGAGCTTCACATCCCTCGTATTTTGTACAATTTGTCTGCTTACATGTGCTACAGCATTATGCACAACCTTAA
GGTTTACAGACCTCACAAGCAGCCTAATCTAGACGAATCTCAAAGTTTCGTGGTTCCTGGTTTACCTGATGAGATAAAGTTCAAGTTATCCCAA
CTGACAGATGATCTGAGAAAGCCGGATGACCAAAAGACTGGTTTTGACGAATTGCTCGAACAAGTTGGAGATTCGGAGGAGCGAAGCTATG
GCATTGTTCATGATACATTTTATGAGCTAGAACCTGCATATGTCGACTACTACCAGAAATTAAAGAAACCAAAATGTTGGCATTTTGGTCCGCT
CTCTCATTTTTCATCCAAAATTCGTAGTAAGGAACTAATTTCTGAGCATAACAACAATGAGATTGTTATAGATTGGTTGAATGCACAGAAACCT
AAATCGATTTTCTATGTATCTTTCGGAAGCATTGCTAGATATCCTGAGAGCCAACTGAATGAAATAGCCCAAGCTCTGGATGCTTCAAATGTTC
CTTTCATTTTTGTATTGAGGCCTAATGAAGAAACAACGTCGTGGTTGCCAGTTGGTAATTTAGAGGACAAGACTAAAAAGGGTTTGTACATCA
AAGGGTGGGTCCCACAACTTACGATCATGGAACATTCAGCAACAGGCGGGTTCATGACTCATTGTGGTACTAATTCGGTTCTGGAAGCCATC
ACTTTTGGCGTGCCAATGATAACATGGCCACTTTATGCTGATCAATTCTACAACGAGAAGGTAGTCGAGGTTAGGGGATTGGGA
SGT1_dms78_seq4
CCTTGGACAGTAGATATTGCTGATGAGCTTCACATCCCTCGTATTTTGTACAATTTGTCTGCTTACATGTGCTACAGCATTATGCACAACCTTAA
GGTTTACAGACCTCACAAGCAGCCTAATCTAGACGAATCTCAAAGTTTCGTGGTTCCTGGTTTACCTAATGAGATAAAGTTCAAGTTATCCCAA
CTGACAGATGATCTGAGAAAGCCGGATGACCAAAAGACTGTTTTTGACGAATTGCTCGAACAAGTTGGAGATTCGGAGGAACGAAGCTATG
GCATTGTTCATGATACATTTTATGAGCTAGAACCTGCATATGTCGACTATTACCAGAAATTAAAGAAACCAAAATGTTGGCATTTTGGTCCGCT
CTCTCATTTTGCATCCAAAATTCGTAGTAAGGAACTAATTTCTGAGCATAACAACAATGAGATTGTTATAGATTGGTTGAATGCACAGAAACCT
AAATCGGTTCTCTATGTATCTTTCGGAAGCATGGCTAGATTTCCTGAGAGCCAACTGAATGAAATAGCCCAAGCTCTGAATGCTTCAAATGTTC
CTTTCATTTTTGTATTGAGGCCTAATGAAGAAACAACGTCGTGGTTGCCAGTTGGTAATTTAGAGGACAAGACTAAAAAGGGTTTGTACATCA
AAGGGTGGGTCCCACAACTTACGATCATGGAACATTCCGCAACAGGCGGATTCATGACTCATTGTGGTACTAATTCGGTTCTGGAAGCCATCA
CTTTTGGCGTGCCAATGATAACATGGCCACTTTATGCTGATCAATTCTACAACGAGAAGGTAGTCGAGGTTAGGGGATTGGGA
SGT1_slp16_seq1
CCTTGGACAGTAGATATTGCTGATGAGCTTCACATCCCTCGTATTTTGTACAATTTGTCTGCTTACATGTGCTACAGCATTATGCACAACCTTAA
GGTTTACAGACCTCACAAGCAGCCTAATCTAGACGAATCTCAAAGTTTCGTGGTTCCTGGTTTACCTGATGAGATAAAGTTCAAGTTATCCCAA
CTGACAGATGATCTGAGAAAGCCGGATGACCAAAAGACTGTTTTTGACGAATTGCTCGAACAAGTTGGAGATTCGGAGGAACGAAGCTATG
GCATTGTTCATGATACATTTTATGAGCTAGAACCTGCATATGTTGACTACTACCAGAAATTAAAGAAACCAAAATGTTGGCATTTTGGTCCGCT
CTCTCATTTTGCATCCAAAATCCGTAGTAAGGAACTAATTTCTGAGCATAACAACAATGAGATTGTTATAGATTGGTTGAATGCACAGAAACCT
AAATCGGTTCTCTATGTATCTTTCGGAAGCATGGCTAGATTTCCTGAGAGCCAACTGAATGAAATAGCCCAAGCTCTAGATGCTTCAAATGTTC
CTTTCATTTTTGTATTGAGGCCTAATGAAGAAACGGCGTCGTGGTTACCAGTTGGTAATTTAGAGGACAAGACTAAAAAGGGTTTGTACATCA
AAGGGTGGGTCCCACAGCTTACGATCATGGAACATTCAGCAACAGGCGGGTTCATGACTCATTGTGGTACTAATTCGGTTCTGGAAGCCATC
ACTTTTGGCGTGCCAATGATAACATGGCCACTTTATGCTGATCAATTCTACAACGAGAAGGTAGTCGAGGTTAGGGGATTGGGA
SGT1_slp16_seq2
CCTTGGACAGTAGATATTGCTGATGAGCTTCACATCCCTCGTATTTTGTACAATTTGTCTGCTTACATGTGCTACAGCATTATGCACAACCTTAA
GGTTTACAGACCTCACAAGCAGCCTAATCTAGACGAATCTCAAAGTTTCGTGGTTCCTGGTTTACCTGATGAGATAAAGTTCAAGTTATCCCAA
CTGACAGATGATCTGAGAAAGCCGGATGACCAAAAGACTGTTTTTGACGAATTGCTCGAACAAGTTGGAGATTCGGAGGAACGAAGCTATG
GCATTGTTCATGATACATTTTATGAGCTAGAACCTGCCTATGTCGACTACTACCAGAAATTAAAGAAACCAAAATGTTGGCATTTTGGTCCGCT
CTCTCATTTTGCATCCAAAATCCGTAGTAAGGAACTAATTTCTGAGCATAACAACAATGAGATTGTTATAGATTGGTTGAATGCACAGAAACCT
AAATCGGTTCTCTATGTATCTTTCGGAAGCATGGCTAGATTTCCTGAGAGCCAACTGAATGAAATAGCCCAAGCTCTGGATGCTTCAAATGTTC
CTTTCATTTTTGTATTGAGGCCTAATGAAGAAACAGCGTCGTGGTTGCCAGTTGGTAATTTAGAGGACAAGTCTAAAAAGGGTTTGTACATCA
AAGGGTGGGTCCCACAGCTTACGATCATGGAACATTCAGCAACAGGCGGGTTCATGACTCATTGTGGTACTAATTCGGTTTTGGAAGCCATC
ACTTTTGGCGTGCCAATGATAACATGGCCACTTTATGCTGATCAATTCTACAACGAGAAGGTAGTCGAGGTTAGGGGATTGGGA
SGT1_slp81_seq2
CCTTGGACAGTAGATATTGCTGATGAGCTTCACATCCCTCGTATTTTGTACAATTTGTCTGCTTACATGTGCTACAGCATTATGCACAACCTTAA
GGTTTACAGACCTCACAAGCAGCCTAATCTAGACGAATCTCAAAGTTTCGTGGTTCCTGGTTTACCTGATGAGATAAAGTTCAAGTTATCCCAA
CTGACAGATGATCTGAGAAAGCCGGATGACCAAAAGACTGTTTTTGACGAATTGCTCGAACAAGTTGGAGATTCGGAGGAACGAAGCTATG
GCATTGTTCATGATACATTTTATGAGCTAGAACCTGCCTATGTCGACTACTACCAGAAATTAAAGAAACCAAAATGTTGGCATTTTGGTCCGCT
CTCTCATTTTGCATCCAAAATCCGTAGTAAGGAACTAATTTCTGAGCATAACAACAATGAGATTGTTATAGATTGGTTGAATGCACAGAAACCT
AAATCGGTTCTCTATGTATCTTTCGGAAGCATGGCTAGATTTCCTGAGAGCCAACTGAATGAAATAGCCCAAGCTCTGGATGCTTCAAATGTTC
CTTTCATTTTTGTATTGAGGCCTAATGAAGAAACAGCGTCGTGGTTGCCAGTTGGTAATTTAGAGGACAAGTCTAAAAAGGGTTTGTACATCA
AAGGGTGGGTCCCACAGCTTACGATCATGGAACATTCAGCAACAGGCGGGTTCATGACTCATTGTGGTACTAATTCGGTTTTGGAAGCCATC
ACTTTTGGCGTGCCAATGATAACATGGCCACTTTATGCTGATCAATTCTACAACGAGAAGGTAGTCGAGGTTAGGGGATTGGGA
SGT1_slp81_seq3
CCTTGGACAGTAGATATTGCTGATGAGCTTCACATCCCTCGTATTTTGTACAATTTGTCTGCTTACATGTGCTACAGCATTATGCACAACCTTAA
GGTTTACAGACCTCACAAGCAGCCTAATCTAGACGAATCTCAAAGTTTCGTGGTTCCTGGTTTACCTGATGAGATAAAGTTCAAGTTATCCCAA
CTGACAGATGATCTGAGAAAGCCGGATGACCAAAAGACTGTTTTTGACGAATTGCTCGAACAAATTGGAGATTCGGAGGAACGAAGCTATG

GCATTGTTCATGATACATTTTATGAGCTAGAACCTGCATATGTCGACTACTACCAGAAATTAAAGAAACCAAAATGTTGGCATTTTGGTCCGCT
CTCTCATTTTGCATCCAAAATCCGTAGTAAGGAACTAATTTCTGAGCATAACAACAATGAGATTGTTATAGATTGGTTGAATGCACAGAAACCT
AAATCGGTTCTCTATGTATCTTTCGGAAGCATGGCTAGATTTCCTGAGAACCAACTGAATGAAATAGCCCAAGCTCTGGATGCTTCAAATGTTC
CTTTCATTTTTGTATTGAGGCCTAATGAAGAAACAGCGTCGTGGTTGCCAGTTGGTAATTTAGAGGACAAGTCTAAAAAGGGTTTGTACATCA
AAGGGTGGGTCCCACAGCTTACGATCATGGAACATTCAGCAACAGGCGGGTTCATGACTCATTGTGGTACTAATTCGGTTCTGGAAGCCATC
ACTTTTGGCGTGCCAATGATAACATGGCCACTTTATGCTGATCAATTCTACAACGAGAAGGTAGTCGAGGTTAGGGGATTGGGA
SGT1_spg55_seq1
CCTTGGACAGTAGATATTGCTGATGAGCTTCACATCCCTCGTATTTTGTACAATTTGTCTGCTTACATGTGCTACAGTATTATGCACAACCTTAA
GCTTTACAGACCTCACAAGCAGCCCAATCTAGACGAATCTCAAAGTTTCGTGGTTCCTGGTTTACCTGATGAGATAAAGTTCAAGTTATCCCAA
CTGACAGAAGATTTGAGAAAGCCGGATGACCAAAAGACTGTTTTTGACGAATTGCTCGAACAAATTCGAGATGCGGAGGAACGAAGCTATG
GCATTGTTCATGATACATTTTATGAGCTAGAACCTGCATATGTCGACTACTACCAGAAATTAAAGAAACCAAAATGTTGGCATTTTGGTCCGCT
CTCTCATTTTGCATCCAAAATCCGTAGTAAGGAACTAATTTCTGAGCATAACAACAATGAGATTGTTGTAGATTGGTTGAATGCACAGAAACCT
AAATCGGTTCTCTATGTATCTTTCGGAAGCATGGCTAGATTTCCTGAGAGCCAACTGAATGAAATATCCCAAGCTCTGGATGCTTCAAATGTTC
CTTTCATTTTTGTATTGAGGCCTAATGAAGAAACGGCGTCGTGGTTGCCAGTTGGTAATTTAGAGGACAAGACTAAAAAGGGTTTGTACATCA
AAGGGTGGGTCCCACAGCTTACGATCATGGAACATGCAGCAACAGGCGGGTTCATGACTCATTGTGGTACTAATTCGGTTCTGGAAGCCATC
ACTTTTGGCGTGCCAATGATAACATGGCCACTTTATGCTGATCAATTCTACAACGAGAAGGTAGTCGAGGTTAGGGGATTGGGA
SGT1_spg55_seq2
CCTTGGACAGTAGATATTGCTGATGAGCTTCACATCCCTCGTATTTTGTACAATTTGTCTGCTTACATGTGCTACAGTATTATGCACAACCTTAA
GCTTTACAGACCTCACAAGCAGCCCAATCTAGACGAATCTCAAAGTTTCGTGGTTCCTGGTTTACCTGATGAGATAAAGTTCAAGTTATCCCAA
CTGACAGAAGATTTGAGAAAGCCGGATGACCAAAAGACTGTTTTTGACGAATTGCTCGAACAAATTCGAGATGCGGAGGAACGAAGCTATG
GCATTGTTCATGATACATTTTATGAGCTAGAACCTGCATATGTCGACTACTACCAGAAATTAAAGAAACCAAAATGTTGGCATTTTGGTCCGCT
CTCTCATTTTGCATCCAAAATCCGTAGTAAGGAACTAATTTCTGAGCATAACAACAATGAGATTGTTGTAGATTGGTTGAATGCACAGAAACCT
AAATCGGTTCTCTATGTATCTTTCGGAAGCATGGCTAGATTTCCTGAGAGCCAACTGAATGAAATATCCCAAGCTCTGGAGACTTCAAATGTTC
CTTTCATTTTTGTATTGAGGCCTAATGAAGAAACGGCGTCGTGGTTGCCAGTTGGTAATTTAGAGGACACGACTAAAAAGGGTTTGTACATCA
AAGGGTGGGTCCCACAGCTTACGATCATGGAACATGCAGCAACAGGCGGGTTCATGACTCATTGTGGTACTAATTCGGTTCTGGAAGCCATC
ACTTTTGGCGTGCCAATGATAACATGGCCACTTTATGCTGATCAATTCTACAACGAGAAGGTAGTCGAGGTTAGGGGATTGGGA
SGT1_spg74_seq3
CCTTGGACAGTAGATATTGCTGATGAGCTTCACATCCCTCGTATTTTGTACAATTTGTCTGCTTACATGTGCTACAGCATTATGCACAACCTTAA
GGTTTACAGACCTCACAAGCAGCCTAATCTAGACGAATCTCAAAATTTCGTGGTTCCTGGTTTACCTGATGAGATAAAGTTTAAGTTATCCCAA
CTGACAGATGATCTGAGAAAGCCGGATGATCAAAAGACTGTTTTTGACGAATTGCTCGAACAAATTGGAGATTCGGAGGAACGAAGCTATG
GCATTGTTCATGATACATTTTATGAGCTAGAACCTGCATATGTCGACTACTACCAAAAATTAAAGAAACCAAAATGTTGGCATTTTGGTCCGCT
CTCTCATTTTGCATCCAAAATCCGTAGTAAGGAACTAATTTCTGAGCATAACAACAATGAGATTGTTATAGATTGGTTGAATGCACAGAAACCT
AAATCGGTTCTCTATGTATCTTTCGGAAGCATGGCTAGATTTCCTGAGAGCCAACTGAATGAAATAGCCCAAGCTCTGGATGCTTCAAATGTTC
CTTTCATTTTTGTATTGAGGCCTAATGAAGAAACAACGTCGTGGTTGCCAGTTGGTAATTTAGAGGACAAGACTAAAAAGGGTTTGTACATCA
AAGGGTGGGTCCCACAGCTTACGATCATGGAACATTCAGCAACAGGCGGGTTCATGACTCATTGTGGTACTAATTCGGTTCTGGAAGCCATC
ACTTTTGGCGTGCCAATGATAACATGGCCACTTTATGCTGATCAATTCTACAACGAGAAGGTAGTCGAGGTTAGGGGATTGGGA
SGT1_spg74_seq4
CCTTGGACAGTAGATATTGCTGATGAGCTTCACATCCCTCGTATTTTGTACAATTTGTCTGCTTACATATGCTACAACATTATGCACAACCTTAA
GGTTTACAGACCTCACAAGCAGCCAAATCTAGACGAATCTCAAAGTTTCGTGGTTCCTGGTTTACCTGATGAGATAAAGTTCAAGTTATCCCA
ACTGACAGATGATCTGAGAAAGCCGGATGACCAAAAGACTGTTTTTGACGAATTGCTAGAACAAGTTGGAGATTCGGAGGAACGAAGCTAT
GGCATTGTTCATGATACATTTTATGAGCTAGAACCTGCATATGTCGACTACTACCAGAAATTAAAGAAACCAAAATGTTGGCATTTTGGTCCGC
TCTCTCATTTTGCATCCAAAATCCGTAGTAAGGAACTAATTTCTGAGCATAACAACAATGAGATTGTTATAGATTGGTTGAATGCACAAAAACC
TAAATCGGTTCTCTATGTATCTTTCGGAAGCATGGCTAGATTTCCTGAGAGCCAACTGAATGAAATAGACCAAGCTCTGGATGCTTCAAATGTT
CCTTTCATTTTTGTATTGAGGCCTAATGAAGAAACAACGTCGTGGTTGCCAGTTGGTAATTTAGAGGACAAGACTAAAAAGGGTTTGTACATC
AAAGGGTGGGTCCCACAGCTTACGATCATGGAACATTCAGCAACAGGCGGGTTCATGACTCATTGTGGTACTAATTCGGTTCTGGAAGCCAT
CACTTTTGGCGTGCCAATGATAACATGGCCACTTTATGCTGATCAATTCTACAACGAGAAGGTAGTCGAGGTTAGGGGATTGGGA
SGT1_sto40_seq1
CCTTGGACAGTAGATATTGCTGATGAGCTGCACATCCCTCGTATTTTGTACAATTTGTCTGCTTACATGTGCTACAGCATTATGCACAACCTTAA
GGTTTACAGACCTCACAAGCAGCCTAATCTAGACGAATCTCAAAGTTTCGTGGTTCCTGGTTTACCTGATGAGATAAAGTTCAAGTTATCCCAA
CTGACAGATGATCTGAGAAAGCCGGATGACCAAAAGACTGGTTTTGACGAATTGCTCGAACAAGTTGGAGATTCGGAGGAGCGAAGCTATG
GCATTGTTCATGATACATTTTATGAGCTAGAACCTGCATATGTCGACTACTACCAGAAATTAAAGAAACCAAAATGTTGGCATTTTGGTCCGCT
CTCTCATTTTGCATCCAAAATCCGTAGTAAGGAACTAATTTCTGAGCATAACAACAATGAGATTGTTATAGATTGGTTGAATGCACAGAAACCT
AAATCGGTTCTCTATGTATCTTTCGGAAGCATGGCTAGATTTCCTGAGAGCCAACTGAATGAAATAGCCCAAGCTCTGGATGCTTCAAATGTTC
CTTTCATTTTTGTATTGAGGCCTAATGAAAAAACAACGTCGTGGTTGCCAGTTGGTAATTTAGAGGACAAGACTAAAAAGGGTTTGTACATCA
AAGGGTGGGTCCCACAGCTTACGATCATGGAACATTCAGCAACAGGCGGGTTCATGACTCATTGTGGTACTAATTCGGTTCTGGAAGCCATC
ACTTTTGGCGTGCCAATGATAACATGGCCACTTTATGCTGATCAATTCTACAACGAGAAGGTAGTCGAGGTTAGGGGATTGGGA
SGT1_sto40_seq2

CCTTGGACAGTAGATATTGCTGAAGAGCTTCACATTCCTCGTATTTTGTACAATTTGTCTGCTTACATGTGCTACAGTATTATGCACAACCTTAA
GCTTTACAGACCTCACAAGCAGCCTAATCTAGACGAATCTCAGAGTTTCGTGGTTCCTGGTTTACCTGATGAGATAAAGTTCAAGTTATCCCAA
CTGACAGATGATCTGCGAAAGCCGGATGACCAAAAGACTGGTTTTGACGAATTACTCAAACAAGTTCGAGATTCGGAGGAACGAAGCTTTGG
CATTGTTCATGATACATTTTATGAGCTAGAACCTGCATACATCAACTACTATCAGAAATTAAAGAAACCAAAATGTTGGCATTTTGGTCCGCTC
TCTCATTTTGCATCCAAAATCCATAGTAAGGAACTAATTACTGAGCATAACAAC---
GACATCGTTGTAGATTGGTTGAATGCACAGAAACCTAAATCGGTTCTGTATGTATCTTTCGGAAGCATGGCTAGATTTCCTGAGAGCCAACTG
AATGAAATAGCGCATGCTCTGGATGCTTCAAATGTTCCTTTCATTTTTGTATTGAGGCCTAATGAAGAAACAGCGTCCTGGTTGCCAGTTGGTA
ATTTAGAGGACAAGACTAAAAAGGGTTTGTACATCAAAGGATGGGTCCCACAGCTCACGATCATGGAACATTCAGCAACAGGCGGGTTCATG
ACTCATTGTGGTACTAATTCGGTTCTGGAAGCCATCACTTTTGGCGTGCCAATGATAACATGGCCACTTTATGCTGATCAATTCTACAACGAGA
AGGTAGTCGAGGTTAGGGGATTGGGA

SGT1_sto61_seq1

CCTTGGACAGTAGATATTGCTGATGAGCTGCACATCCCTCGTATTTTGTACAATTTGTCTGCTTACATGTGCTACAGCATTATGCACAACCTTAA
GGTTTACAGACCTCACAAGCAGCCTAATCTAGACGAATCTCAAAGTTTCGTGGTTCCTGGTTTACCTGATGAGATAAAGTTCAAGTTATCCCAA
CTGACAGATGATCTGAGAAAGCCGGATGACCAAAAGACTGGTTTTGACGAATTGCTCGAACAAGTTGGAGATTCGGAGGAGCGAAGCTATG
GCATTGTTCATGATACATTTTATGAGCTAGAACCTGCATATGTCGACTACTACCAGAAATTAAAGAAACCAAAATGTTGGCATTTTGGTCCGCT
CTCTCATTTTGCATCCAAAATCCGTAGTAAGGAACTAATTTCTGAGCATAACAACAATGAGATTGTTATAGATTGGTTGAATGCACAGAAACCT
AAATCGGTTCTCTATGTATCTTTCGGAAGCATGGCTAGATTTCCTGAGAGCCAACTGAATGAAATAGCCCAAGCTCTGGATGCTTCAAATGTTC
CTTTCATTTTTGTATTGAGGCCTAATGAAAAAACAACGTCGTGGTTGCCAGTTGGTAATTTAGAGGACAAGACTAAAAAGGGTTTGTACATCA
AAGGGTGGGTCCCACAGCTTACGATCATGGAACATTCAGCAACAGGCGGGTTCATGACTCATTGTGGTACTAATTCGGTTCTGGAAGCCATC
ACTTTTGGCGTGCCAATGATAACATGGCCACTTTATGCTGATCAATTCTACAACGAGAAGGTAGTCGAGGTTAGGGGATTGGGA

SGT1_sto61_seq2

CCTTGGACAGTAGATATTGCTGAAGAGCTTCACATTCCTCGTATTTTGTACAATTTGTCTGCTTACATGTGCTACAGTATTATGCACAACCTTAA
GCTTTACAGACCTCACAAGCAGCCTAATCTAGACGAATCTCAGAGTTTCGTGGTTCCTGGTTTACCTGATGAGATAAAGTTCAAGTTATCCCAA
CTGACAGATGATCTGCGAAAGCCGGATGACCAAAAGACTGGTTTTGACGAATTACTCAAACAAGTTCGAGATTCGGAGGAACGAAGCTTTGG
CATTGTTCATGATACATTTTATGAGCTAGAACCTGCATACATCAACTACTATCAGAAATTAAAGAAACCAAAATGTTGGCATTTTGGTCCGCTC
TCTCATTTTGCATCCAAAATCCATAGTAAGGAACTAATTACTGAGCATAACAAC---
GACATCGTTGTAGATTGGTTGAATGCACAGAAACCTAAATCGGTTCTGTATGTATCTTTCGGAAGCATGGCTAGATTTCCTGAGAGCCAACTG
AATGAAATAGCGCATGCTCTGGATGCTTCAAATGTTCCTTTCATTTTTGTATTGAGGCCTAATGAAGAAACAGCGTCCTGGTTGCCAGTTGGTA
ATTTAGAGGACAAGACTAAAAAGGGTTTGTACATCAAAGGATGGGTCCCACAGCTCACGATCATGGAACATTCAGCAACAGGCGGGTTCATG
ACTCATTGTGGTACTAATTCGGTTCTGGAAGCCATCACTTTTGGCGTGCCAATGATAACATGGCCACTTTATGCTGATCAATTCTACAACGAGA
AGGTAGTCGAGGTTAGGGGATTGGGA

SGT1_chc80-1_seq1

CCTTGGACAGTAGATATTGCTGATGAGCTTCACATCCCTCGTATTTTGTACAATTTGTCTGCTTACATGTGCTACAGCATTATGCACAACCTTAA
GGTTTACAGACCTCACAAGCAGCCTAATCTAGACGAATCTCAAAGTTTCGTGGTTCCTGGTTTACCTGATGAGATAAAGTTCAAGCTATCCCAA
TTGACAGATGATCTGAGAAAGCCGGATGACCAAAAGACTGTTTTTGACGAATTGCTCGAACAAGTTAGAGATTCGGAGGAACGAAGCTATG
GCATTGTTCATGATACATTTTATGAGCTAGAACCTGCATATGTCGACTACTACCAGAAATTAAAGAAATCAAAATGTTGGCATTTTGGTCCGCT
CTCTCATTTTGCATCCAAAATCCGTAGTAAGGAACTAATTTCTGAGCATAACAACAATGAGATTGTTATAGATTGGTTGAATGCACAGAAACCT
AAATCGGTTCTCTATGTATCTTTCGGAAGCATGGCTAGATTTCCTGAGAGCCAACTGAATGAAATAGCCCAAGCTCTGGATGCTTCAAATGTTC
CTTTCATTTTTGTATTGAGGCCTAATGAAGAAACAATGTCGTGGTTGCCAGTTGGTAATTTAGAGGACAAGACTAAAAAGGGTTTGTACATCA
AAGGGTGGGTCCCACAGCTTACGATCATGGAACATTCAGCAACAGGCGGGTTCATGACTCATTGTGGTACTAATTCGGTTCTGGAAGCCATC
ACTTTTGGCGTGCCAATGATAACATGGCCACTTTATGCTGATCAATTCTACAACGAGAAGGTAGTCGAGGTTAGGGGATTGGGA

SGT1_chc80-1_seq2

CCTTGGACAGTAGATATTGCTGATGAGCTTCACATCCCTCGTATTTTGTACAATTTGTCTGCTTACATGTGCTACAGCATTATGCACAACCCTAA
CCATTACAGACCTCACAAGCAGCCTAATCTAGATGAATCTCAAAGTTTTGTGGTTCCTGGTTTACCTGATGAGATAAAGTTCAAGTTATCCCAA
CTGACAGATGATCTGAGAAATTCGGATGACCAAAAGACTGTTTTTGACGAATTGCTCGAACAAGTTGGAGATTCGGAGGAACGAAGCTATGG
CATTGTTCATGATACATTTTATGAGCTAGAACCTGCATATGTCGACTACTACAAGAAATTAAAGAAACCAAAATGTTGGCAGTTTGGTCCGCTC
TCTCATTTTGCATCCAAAATCCGTAGTAGGGAACTAATTTCTAAGGATAACAACAATGAGATCGTTGTAGATTGGTTGAATGCACAGAAACCT
AAATCGGTTCTCTATGTATCTTTCGGAAGCATGGCTAGATTTCCTGAGAGCCAACTGAATGAAATAGCCCAAGCTCTGGAAGCTTCAAATGTT
CCTTTCATTTTTGTATTGAGGCCTAATGAAGAAACGGCGTCGTGGTTGCCAGTTGGTAATTTAGAGGACAAGACTAAAAAGGGTTTGTACATC
AAATGGTGGGTCCCACAACTTACGATCATGGAACATTCAGCAACAGGCAGGTTCATGACTCATTGTGGTACTAATTCGGTTCTGGAAGCCATC
ACTTTTGGCGTGCCAATGATAACATGGCCACTTTATGTTGATCAATTCTACAATGAGAAGGTAGTCGAGGTTAGGGGATTGGGA

SGT1_phuDM_seq1

CCTTGGACAGTAGATATTGCTGATGAGCTTCACATCCCTCGTATTTTGTACAATTTGTCTGCTTACATGTGCTACAGCATTATGCACAACCTTAA
GCTTTACAGACCTCACAAGCAGCCTAATCTAGACGAATCTCAAAGTTTCGTGGTTCCTGGTTTACCTGATGAGATGAAGTTCAAGTTATCCCAA
CTGACAGATGATCTGAGAAAAACCGGATGACCAAAAGACTGGTTTTGACGAATTGCTCGATAAAGTACGAGATTCGGAGGAACGAAGCTATG
GCATTGTTCATGATACATTTTATGAGCTAGAACCTGCATATGTCGACTACTACCAGAAATTAAAGAAACCAAAATGTTGGCATTTTGGTCCGCT
CTCTCATTTTGCATCCAAAATCCGTAGTAAGGAGCTAATTGCTGAGCATAACAACAATGAGATTGTTGTAGATTGGTTGAATGCACAGAAACC
TAAATCGGTTCTCTATGTATCTTTCGGAAGCATGGCTAGATTTCCTGAGAGCCAACTGAATGAAATATCCCAAGCTCTGGATGCTTCAAATGTT

CCTTTCATTTTTGTATTGAGGCCTAATGAAGAAACGGCGTCGTGGTTGCCAGTTGGTAATTTAGAGGACAAGACTAAAAAGGGTTTGTACATC
AAAGGGTGGGTCCCACAGCTTACGATCATGGAACATTCAGCAACAGGCGGGTTCATGACTCATTGTGGTACTAATTCGGTTCTGGAAGCCAT
CACTTTTGGCGTGCCAATGATAACATGGCCACTTTATGCTGATCAATTCTACAACGAGAAGGTAGTCGAGGTTAGGGGATTGGGA
SGT1_phuDM_seq1-2
CCTTGGACAGTAGATATTGCTGATGAGCTTCACATCCCTCGTATTTTGTACAATTTGTCTGCTTACATGTGCTACAGCATTATGCACAACCTTAA
GCTTTACAGACCTCACAAGCAGCCTAATCTAGACGAATCTCAAAGTTTCGTGGTTCCTGGTTTACCTGATGAGATGAAGTTCAAGTTATCCCAA
CTGACAGATGATCTGAGAAAACCGGATGACCAAAAGACTGGTTTTGACGAATTGCTCGATAAAGTACGAGATTCGGAGGAACGAAGCTATG
GCATTGTTCATGATACATTTTATGAGCTAGAACCTGCATATGTCGACTACTACCAGAAATTAAAGAAACCAAAATGTTGGCATTTTGGTCCGCT
CTCTCATTTTGCATCCAAAATCCGTAGTAAGGAGCTAATTGCTGAGCATAACAACAATGAGATTGTTGTAGATTGGTTGAATGCACAGAAACC
TAAATCGGTTCTCTATGTATCTTTCGGAAGCATGGCTAGATTTCCTGAGAGCCAACTGAATGAAATATCCCAAGCTCTGGATGCTTCAAATGTT
CCTTTCATTTTTGTATTGAGGCCTAATGAAGAAACGGCGTCGTGGTTGCCAGTTGGTAATTTAGAGGACAAGACTAAAAAGGGTTTGTACATC
AAAGGGTGGGTCCCACAGCTTACGATCATGGAACATTCAGCAACAGGCGGGTTCATGACTCATTGTGGTACTAATTCGGTTCTGGAAGCCAT
CACTTTTGGCGTGCCAATGATAACATGGCCACTTTATGCTGATCAATTCTACAACGAGAAGGTAGTCGAGGTTAGGGGATTGGGA
SGT1_phuDH_seq1
CCTTGGACAGTAGATATTGCTGATGAGCTTCACATCCCTCGTATTTTGTACAATTTGTCTGCTTACATGTGCTACAGCATTATGCACAACCTTAA
GCTTTACAGACCTCACAAGCAGCCTAATCTAGACGAATCTCAAAGTTTCGTGGTTCCTGGTTTACCTGATGAGATGAAGTTCAAGTTATCCCAA
CTGACAGATGATCTGAGAAAACCGGATGACCAAAAGACTGGTTTTGACGAATTGCTCGATAAAGTACGAGATTCGGAGGAACGAAGCTATG
GCATTGTTCATGATACATTTTATGAGCTAGAACCTGCATATGTCGACTACTACCAGAAATTAAAGAAACCAAAATGTTGGCATTTTGGTCCGCT
CTCTCATTTTGCATCCAAAATCCGTAGTAAGGAGCTAATTGCTGAGCATAACAACAATGAGATTGTTGTAGATTGGTTGAATGCACAGAAACC
TAAATCGGTTCTCTATGTATCTTTCGGAAGCATGGCTAGATTTCCTGAGAGCCAACTGAATGAAATATCCCAAGCTCTGGATGCTTCAAATGTT
CCTTTCATTTTTGTATTGAGGCCTAATGAAGAAACGGCGTCGTGGTTGCCAGTTGGTAATTTAGAGGACAAGACTAAAAAGGGTTTGTACATC
AAAGGGTGGGTCCCACAGCTTACGATCATGGAACATTCAGCAACAGGCGGGTTCATGACTCATTGTGGTACTAATTCGGTTCTGGAAGCCAT
CACTTTTGGCGTGCCAATGATAACATGGCCACTTTATGCTGATCAATTCTACAACGAGAAGGTAGTCGAGGTTAGGGGATTGGGA
SGT1_phuDH_seq2
CCTTGGACAGTAGATATTGCTGATGAGCTTCACATCCCTCGTATTTTGTACAATTTGTCTGCTTACATGTGCTACAGCATTATGCACAACCTTAA
GGTTTACAGACCTCACAAGCAGCCTAATCTAGACGAATCTCAAAGTTTCGTGGTTCCTGGTTTACCTGATGAGATAAAGTTCAAGTTATCCCAA
CTGACAGATGATCTGAGAAAGCCGGATGACCAAAAGACTGTTTTTGACGAATTGCTCGAACAAATTGGAGATTCGGAGGAACGAAGCTATG
GCATTGTTCATGATACATTTTATGAGCTAGAACCTGCATATGTCGACTACTACCAGAAATTAAAGAAACCAAAATGTTGGCATTTTGGTCCGCT
CTCTCATTTTGCATCCAAAATCCGTAGTAAGGAACTAATTTCTGAGCATAACAACAATGAGATTGTTATAGATTGGTTGAATGCACAGAAACCT
AAATCGGTTCTCTATGTATCTTTCGGAAGCATGGCTAGATTTCCTGAGAGCCAACTGAATGAAATAGCCCAAGCTCTGGATGCTTCAAATGTTC
CTTTCATTTTTGTATTGAGGCCTAATGAAGAAACGGCGTCGTGGTTGCCCGTTGGTAATTTAGAGGACAAGACTAAAAAGGGTTTGTACATCA
AAGGGTGGGTCCCACAGCTTACGATCATGGAACATTCAGCAACAGGCGGGTTCATGACTCATTGTGGTACTAATTCGGTTCTGGAAGCCATC
ACTTTTGGCGTGCCAATGATAACATGGCCACTTTATGCTGATCAATTCTACAACGAGAAGGTAGTCGAGGTTAGGGGATTGGGA
SGT1_tbr_seq1
CCTTGGACAGTAGATATTGCTGATGAGCTTCACATCCCTCGTATTTTGTACAATTTGTCTGCTTACATGTGCTACAGCATTATGCACAACCTTAA
GGTTTACAGACCTCACAAGCAGCCTAATCTAGACGAATCTCAAAGTTTCGTGGTTCCTGGTTTACCTGATGAGATAAAGTTCAAGTTATCCCAA
CTGACAGATGATCTGAGAAAGTCGGATGACCAAAAGACTGTTTTTGACGAATTGCTCGAACAAGTTGAAGATTCGGAGGAACGAAGCTATG
GCATTGTTCATGATACATTTTATGAGCTAGAACCTGCATATGTTGACTACTACCAGAAATTAAAGAAACCAAAATGTTGGCATTTTGGTCCGCT
CTCTCATTTTGCATCCAAAATCCGTAGTAAGGAACTAATTTCTGAGCATAACAACAATGAGATTGTTATAGATTGGTTGAATGCACAGAAACCT
AAATCGGTTCTCTATGTATCTTTCGGAAGCATGGCTAGATTTCCTGAGAGCCAACTGAATGAAATAGCCCAAGCTCTGGATGCTTCAAATGTTC
CTTTCATTTTTGTATTGAGGCCTAATGAAGAAACGGCGTCGTGGTTGCCAGTTGGTAATTTAGAGGACAAGACTAAAAAGGGTTTGTACATCA
AAGGGTGGGTCCCACAGCTTACGATCATGGAACATTCAGCAACAGGCGGGTTCATGACTCATTGTGGTACTAATTCGGTTCTGGAAGCCATC
ACTTTTGGCGTGCCAATGATAACATGGCCACTTTATGCTGATCAATTCTACAACGAGAAGGTAGTCGAGGTTAGGGGATTGGGA
SGT1_tbr_seq1-2
CCTTGGACAGTAGATATTGCTGATGAGCTTCACATCCCTCGTATTTTGTACAATTTGTCTGCTTACATGTGCTACAGCATTATGCACAACCTTAA
GGTTTACAGACCTCACAAGCAGCCTAATCTAGACGAATCTCAAAGTTTCGTGGTTCCTGGTTTACCTGATGAGATAAAGTTCAAGTTATCCCAA
CTGACAGATGATCTGAGAAAGTCGGATGACCAAAAGACTGTTTTTGACGAATTGCTCGAACAAGTTGAAGATTCGGAGGAACGAAGCTATG
GCATTGTTCATGATACATTTTATGAGCTAGAACCTGCATATGTTGACTACTACCAGAAATTAAAGAAACCAAAATGTTGGCATTTTGGTCCGCT
CTCTCATTTTGCATCCAAAATCCGTAGTAAGGAACTAATTTCTGAGCATAACAACAATGAGATTGTTATAGATTGGTTGAATGCACAGAAACCT
AAATCGGTTCTCTATGTATCTTTCGGAAGCATGGCTAGATTTCCTGAGAGCCAACTGAATGAAATAGCCCAAGCTCTGGATGCTTCAAATGTTC
CTTTCATTTTTGTATTGAGGCCTAATGAAGAAACGGCGTCGTGGTTGCCAGTTGGTAATTTAGAGGACAAGACTAAAAAGGGTTTGTACATCA
AAGGGTGGGTCCCACAGCTTACGATCATGGAACATTCAGCAACAGGCGGGTTCATGACTCATTGTGGTACTAATTCGGTTCTGGAAGCCATC
ACTTTTGGCGTGCCAATGATAACATGGCCACTTTATGCTGATCAATTCTACAACGAGAAGGTAGTCGAGGTTAGGGGATTGGGA

1.5 Solanidine glucosyltransferase (*SGT2*) sequences

27

SGT2_cmm7_seq1

CCTGCGGATGAGAGGAATGCTTTTGATGAATTGCTTGATCAAACCAGAGAATCTGAGGATCGAAGCTATGGTATCGTTCACGATACCTTTTAC
GAGCTAGAACCTGACTACGCTGAGTACTATCAGAAGATGAAGAAAACCAAATGTTGGCAAATTGGTCCCATTTCCTATTTTTCTTCCAAATTAT
CCCGAAGAAAAGAGCTGATTAGTTCTGCTGATGAAAGTATTTCATCTGTTGTGGAGTGGTTGAATAAACAAAAGCACAAATCGGTCCTCTACG
TCTCTTTCGGGAGCATAGTTACATTCCCAGAGGAGCAACTCGCTGAAATCGCAAAAGCTCTAGAAGCTTCTACCGTCCCTTTCATTTGGGCAGT
GAAGAAAGACCAATCAGCAAAAACCACGTGGTTACCGGAGAGTTTGTTCGATGAGAAAAAAGGTCTGATTATTAAAGGGTGGGCGCCGCAA
CTAACCATCTTAGATCATTCAGCAATAGGAGGATTCATGACACACTGTGGATGGAATTCGGTGCTTGAAGCTATCATCGCTGGGGTGCCGTTG
GTG

SGT2_cmm7_seq1-2

CCTGCGGATGAGAGGAATGCTTTTGATGAATTGCTTGATCAAACCAGAGAATCTGAGGATCGAAGCTATGGTATCGTTCACGATACCTTTTAC
GAGCTAGAACCTGACTACGCTGAGTACTATCAGAAGATGAAGAAAACCAAATGTTGGCAAATTGGTCCCATTTCCTATTTTTCTTCCAAATTAT
CCCGAAGAAAAGAGCTGATTAGTTCTGCTGATGAAAGTATTTCATCTGTTGTGGAGTGGTTGAATAAACAAAAGCACAAATCGGTCCTCTACG
TCTCTTTCGGGAGCATAGTTACATTCCCAGAGGAGCAACTCGCTGAAATCGCAAAAGCTCTAGAAGCTTCTACCGTCCCTTTCATTTGGGCAGT
GAAGAAAGACCAATCAGCAAAAACCACGTGGTTACCGGAGAGTTTGTTCGATGAGAAAAAAGGTCTGATTATTAAAGGGTGGGCGCCGCAA
CTAACCATCTTAGATCATTCAGCAATAGGAGGATTCATGACACACTGTGGATGGAATTCGGTGCTTGAAGCTATCATCGCTGGGGTGCCGTTG
GTG

SGT2_cmm26_seq1

CCTGCGGATGAGAGGAATGCTTTTGATGAATTGCTTGATCAAACCAGAGAATCTGAGGATCGAAGCTATGGTATCGTTCACGATACCTTTTAC
GAGCTAGAACCTGACTACGCTGAGTACTATCAGAAGATGAAGAAAACCAAATGTTGGCAAATTGGTCCCATTTCCTATTTTTCTTCCAAATTAT
CCCGAAGAAAAGAGCTGATTAGTTCTGCTGATGAAAGTATTTCATCTGTTGTGGAGTGGTTGAATAAACAAAAGCACAAATCGGTCCTCTACG
TCTCTTTCGGGAGCATAGTTACATTCCCAGAGGAGCAACTCGCTGAAATCGCAAAAGCTCTAGAAGCTTCTACCGTCCCTTTCATTTGGGCAGT
GAAGAAAGACCAATCAGCAAAAACCACGTGGTTACCGGAGAGTTTGTTCGATGAGAAAAAAGGTCTGATTATTAAAGGGTGGGCGCCGCAA
CTAACCATCTTAGATCATTCAGCAATAGGAGGATTCATGACACACTGTGGATGGAATTCGGTGCTTGAAGCTATCATCGCTGGGGTGCCGTTG
GTG

SGT2_cmm26_seq1-2

CCTGCGGATGAGAGGAATGCTTTTGATGAATTGCTTGATCAAACCAGAGAATCTGAGGATCGAAGCTATGGTATCGTTCACGATACCTTTTAC
GAGCTAGAACCTGACTACGCTGAGTACTATCAGAAGATGAAGAAAACCAAATGTTGGCAAATTGGTCCCATTTCCTATTTTTCTTCCAAATTAT
CCCGAAGAAAAGAGCTGATTAGTTCTGCTGATGAAAGTATTTCATCTGTTGTGGAGTGGTTGAATAAACAAAAGCACAAATCGGTCCTCTACG
TCTCTTTCGGGAGCATAGTTACATTCCCAGAGGAGCAACTCGCTGAAATCGCAAAAGCTCTAGAAGCTTCTACCGTCCCTTTCATTTGGGCAGT
GAAGAAAGACCAATCAGCAAAAACCACGTGGTTACCGGAGAGTTTGTTCGATGAGAAAAAAGGTCTGATTATTAAAGGGTGGGCGCCGCAA
CTAACCATCTTAGATCATTCAGCAATAGGAGGATTCATGACACACTGTGGATGGAATTCGGTGCTTGAAGCTATCATCGCTGGGGTGCCGTTG
GTG

SGT2_dms54_seq1

CCTGCGGATGAGAGGAATGCTTTTGATGAATTGCTCGATCGAACCAGAGAATCTGAGGATCTAAGCTACGGTATCGTTCATGATACTTTTTAC
GAGCTAGAACCTGCCTACGCTGACTACTATCAGAAGATGAAGAAAACCAAATGTTGGCAAATTGGTCCCATTTCCTATTTTTCTTCCAAATTAT
CCCCAAGAAAAGAACTGATTAATTCTTCTGATGAAAGTAACTCATCTGCCGTTGTTGTAGAGTGGTTGAATAAACATAAGCACAAATCGGTCC
TCTACGTCTCTTTTGGGAGCACAATTAGATTCCCAGAGGAGCAACTCGCTGAAATCGCAAAAGCTCTAGAAGCTTCTACCGTCCCTTTCATTTG
GGTAGTAAACAAAGACCAATTAGCAAAAACCACGTGGTTACCGGAGAGTTTGTTCGATGAGAAAAAAATGTCTGATTATTAAAGGGTGGGCAC
CGCAACTATCCATCTTAGATCATTCAGCAGTCGGAGGATTCATGACACACTGTGGTTGGAATTCAGTGCTTGAAGCCATCATCGCTGGGGTGC
CGTTGGTG

SGT2_dms54_seq1-2

CCTGCGGATGAGAGGAATGCTTTTGATGAATTGCTCGATCGAACCAGAGAATCTGAGGATCTAAGCTACGGTATCGTTCATGATACTTTTTAC
GAGCTAGAACCTGCCTACGCTGACTACTATCAGAAGATGAAGAAAACCAAATGTTGGCAAATTGGTCCCATTTCCTATTTTTCTTCCAAATTAT
CCCCAAGAAAAGAACTGATTAATTCTTCTGATGAAAGTAACTCATCTGCCGTTGTTGTAGAGTGGTTGAATAAACATAAGCACAAATCGGTCC
TCTACGTCTCTTTTGGGAGCACAATTAGATTCCCAGAGGAGCAACTCGCTGAAATCGCAAAAGCTCTAGAAGCTTCTACCGTCCCTTTCATTTG
GGTAGTAAACAAAGACCAATTAGCAAAAACCACGTGGTTACCGGAGAGTTTGTTCGATGAGAAAAAAATGTCTGATTATTAAAGGGTGGGCAC
CGCAACTATCCATCTTAGATCATTCAGCAGTCGGAGGATTCATGACACACTGTGGTTGGAATTCAGTGCTTGAAGCCATCATCGCTGGGGTGC
CGTTGGTG

SGT2_dms78_seq1

CCTGCGGATGAGAGGAATGCTTTTGATGAATTGCTCGATCGAACCAGAGAATCTGAGGATCTAAGCTACGGTATCGTTCATGATACTTTTTAC
GAGCTAGAACCTGCCTACGCTGACTACTATCAGAAGATGAAGAAAACCAAATGTTGGCAAATTGGTCCCATTTCCTATTTTTCTTCCAAATTAT
CCCCAAGAAAAGAACTGATTAATTCTTCTGATGAAAGTAACTCATCTGCCGTTGTTGTAGAGTGGTTGAATAAACATAAGCACAAATCGGTCC
TCTACGTCTCTTTTGGGAGCACAATTAGATTCCCAGAGGAGCAACTCGCTGAAATCGCAAAAGCTCTAGAAGCTTCTACCGTCCCTTTCATTTG
GGTAGTAAACAAAGACCAATTAGCAAAAACCACGTGGTTACCGGAGAGTTTGTTCGATGAGAAAAAAATGTCTGATTATTAAAGGGTGGGCAC
CGCAACTATCCATCTTAGATCATTCAGCAGTCGGAGGATTCATGACACACTGTGGTTGGAATTCAGTGCTTGAAGCCATCATCGCTGGGGTGC
CGTTGGTG

SGT2_dms78_seq1-2
CCTGCGGATGAGAGGAATGCTTTTGATGAATTGCTCGATCGAACCAGAGAATCTGAGGATCTAAGCTACGGTATCGTTCATGATACTTTTTAC
GAGCTAGAACCTGCCTACGCTGACTACTATCAGAAGATGAAGAAAACCAAATGTTGGCAAATTGGTCCCATTTCCTATTTTTCTTCCAAATTAT
CCCCAAGAAAAGAACTGATTAATTCTTCTGATGAAAGTAACTCATCTGCCGTTGTTGTAGAGTGGTTGAATAAACATAAGCACAAATCGGTCC
TCTACGTCTCTTTTGGGAGCACAATTAGATTCCCAGAGGAGCAACTCGCTGAAATCGCAAAAGCTCTAGAAGCTTCTACCGTCCCTTTCATTTG
GGTAGTAAACAAAGACCAATTAGCAAAAACCACGTGGTTACCGGAGAGTTTGTTCGATGAGAAAAAAATGTCTGATTATTAAAGGGTGGGCAC
CGCAACTATCCATCTTAGATCATTCAGCAGTCGGAGGATTCATGACACACTGTGGTTGGAATTCAGTGCTTGAAGCCATCATCGCTGGGGTGC
CGTTGGTG
SGT2_spg55_seq2
CCTGCGGATGAGAGGAATGCTTTTGATGAATTGCTCGATCGAACCAGAGAATCTGAGGATCAAAGCTACGGTATTGTTCATGATACTTTTTAC
GAGCTAGAACCTGCCTACGCTGACTACTATCAGAAGATGAAGAAAACCAAATGTTGGCAAATTGGTCCCATTTCCTATTTTTCTTCCAATTTAT
TCCGAAGAAAAGATCTGATTAATTCTTTTGATGAAAGTAACTCATCTGCCGCTGTTGTAGAGTGGTTAAATAAACAGAAGCACAAATCGGTCC
TCTACGTCTCTTTCGGGAGCACAGTTAAATTCCCAGAGGAGCAACTCGCTGAAATCGCAAAATCTCTAGAAGCTTCTACCGTCCCTTTCATTTG
GGTAGTGAAGAAGGACCAATCAGCAAAAACCACGTGGTTACCGGAGAGTTTGGTCGATGAGAAAAAAGGTCTGATTATTAATGGGTGGGCG
CCGCAACTAACCATCTTAGATCATTCAGCAGTAGGAGGATTCATGACACACTGTGGATGGAATTCGGTGCTTGAAGCTATCATCGCTGGGGT
GCCGTTGGTG
SGT2_spg74_seq3
CCTGCGGATGAGAGGAATGCTTTTGATGAATTGCTCGATCGAACCAGAGAATCTGAGGATCTAAGCTACGGTATCGTTCATGATACTTTTTAC
GAGCTAGAACCTGCCTACGCTGACTACTATCAGAAGATGAAGAAAACCAAATGTTGGCAAATTGGTCCCATTTCCTATTTTTCTTCCAAATTAT
CCCCAAGAAAAGAACTGATTAATTCTTCTGATGAAAGTAACTCATCTGCCGTTGTTGTAGAGTGGTTGAATAAACATAAGCACAAATCAGTCC
TCTACGTCTCTTTTGGGAGCACAATTAGATTCCCAGAGGAGCAACTCGCTGAAATCGCAAAAGCTCTAGAAGCTTCTACCGTCCCTTTCATTTG
GGTAGTGAAGGAGGACCAATCAGCAAAAACCACGTGGTTACCGGAGAGTTTATTCGATGAGAAAAAAGGTCTGATTATTAATGGGTGGGCG
CCGCAACTAACCATCTTAGATCATTCAGCAGTAGGAGGATTCATGACACACTGTGGATGGAATTCGGTGCTTGAAGCCATCATCGCTGGGGT
GCCGTTGGTG
SGT2_spg74_seq4
CCTGCGGATGAGAGGAATGCTTTTGATGAATTGCTCGATCGAACCAGAGAATCTGAGGATCTAAGCTACGGTATCGTTCATGATACTTTTTAC
GAGCTAGAACCTGCCTACGCTGACTACTATCAGAAGATGAAGAAAACCAAATGTTGGCAAATTGGTCCCATTTCCTATTTTTCTTCCAAATTAT
CCCCAAGAAAAGAACTGATTAATTCTTCTGATGAAAGTAACTCATCTGCCGTTGTTGTAGAGTGGTTGAATAAACATAAGCACAAATCAGTCC
TCTACGTCTCTTTTGGGAGCACAATTAGATTCCCAGAGGAGCAACTCGCTGAAATCGCAAAAGCTCTAGAAGCTTCTACCGTCCCTTTCATTTG
GGTAGTGAAGGAGGACCAATCAGCAAAAACCACGTGGTTACCGGAGAGTTTATTCGATGAGAAAAAAGGTCTGATTATTAATGGGTGGGCG
CCGCAACTAACCATCTTAGATCATTCAGCAGTCGGAGGATTCATGACACACTGTGGTTGGAATTCAGTGCTTGAAGCCATCATCGCTGGGGTG
CCGTTGGTG
SGT2_spl16_seq1
CCTGCGGATGAGAGGAATGCTTTTGATGAATTGCTCGATCGAACCAGAGAATCTGAGGATCTAAGCTACGGTATCGTTCATGATACTTTTTAC
GAGCTAGAACCTGACTACGCTGACTACTATCAGAAGATGAAGAAAACCAAATGTTGGCAAATTGGTCCCATTTCCTATTTTTCTTCCAAATTAT
CCCCAAGAAAAGAACTGATTAATTCTTCTGATGAAAGTAACTCATCTGCCGTTGTTGTAGAGTGGTTGAATAAACATAAGCACAAATCGGTCC
TCTACGTCTCTTTTGGGAGCACAATTAGATTCCCAGAGGAGCAACTCGCTGAAATCGCAAAAGCTCTAGAAGCTTCTACCGTCCCTTTCATTTG
GGTAGTAAACAAAGACCAATTAGCAAAAACCACGTGGTTACCGGAGAGTTTGTTCGATGAGAAAAAAATGTCTGATTATTAAAGGGTGGGCAC
CGCAACTAACCATCTTAGATCATTCAGCAGTCGGAGGATTCATGACACACTGTGGTTGGAATTCAGTGCTTGAAGCCATCATCGCTGGGGTGC
CGTTGGTG
SGT2_spl16_seq1-2
CCTGCGGATGAGAGGAATGCTTTTGATGAATTGCTCGATCGAACCAGAGAATCTGAGGATCTAAGCTACGGTATCGTTCATGATACTTTTTAC
GAGCTAGAACCTGACTACGCTGACTACTATCAGAAGATGAAGAAAACCAAATGTTGGCAAATTGGTCCCATTTCCTATTTTTCTTCCAAATTAT
CCCCAAGAAAAGAACTGATTAATTCTTCTGATGAAAGTAACTCATCTGCCGTTGTTGTAGAGTGGTTGAATAAACATAAGCACAAATCGGTCC
TCTACGTCTCTTTTGGGAGCACAATTAGATTCCCAGAGGAGCAACTCGCTGAAATCGCAAAAGCTCTAGAAGCTTCTACCGTCCCTTTCATTTG
GGTAGTAAACAAAGACCAATTAGCAAAAACCACGTGGTTACCGGAGAGTTTGTTCGATGAGAAAAAAATGTCTGATTATTAAAGGGTGGGCAC
CGCAACTAACCATCTTAGATCATTCAGCAGTCGGAGGATTCATGACACACTGTGGTTGGAATTCAGTGCTTGAAGCCATCATCGCTGGGGTGC
CGTTGGTG
SGT2_spl81_seq2
CCTGCGGATGAGAGGAATGCTTTTGATGAATTGCTCGATCGAACCAGAGAATCTGAGGATCTAAGCTACGGTATCGTTCATGATACTTTTTAC
GAGCTAGAACCTGCCTACGCTGACTACTATCAGAAGATGAAGAAAACCAAATGTTGGCAAATTGGTCCCATTTCCTATTTTTCTTCCAAATTAT
CCCCAAGAAAAGAACTGATTAATTCTTCTGATGAAAGTAACTCATCTGCCGTTGTTGTAGAGTGGTTGAATAAACATAAGCACAAATCGGTCC
TCTACGTCTCTTTTGGGAGCACAATTAGATTCCCAGAGGAGCAACTCGCTGAAATCGCAAAAGCTCTAGAAGCTTCTACCGTCCCTTTCATTTG
GGTAGTAAACAAAGACCAATTAGCAAAAACCACGTGGTTACCGGAGAGTTTGTTCGATGAGAAAAAAATGTCTGATTATTAAAGGGTGGGCAC
CGCAACTAACCATCTTAGATCATTCAGCAGTCGGAGGATTCATGACACACTGTGGTTGGAATTCAGTGCTTGAAGCCATCATCGCTGGGGTGC
CGTTGGTG
SGT2_spl81_seq3

CCTGCGGATGAGAGGAATGCTTTTGATGAATTGCTCGATCGAACCAGAGAATCTGAGGATCTAAGCTACGGTATCGTTCATGATACTTTTTAC
GAGCTAGAACCTGCCTACGCTGACTACTATCAGAAGATGAAGAAAACCAAATGTTGGCAAATTGGTCCCATTTCCTATTTTTCTTCCAAATTAT
CCCCAAGAAAAGAACTGATTAATTCTTCTGATGAAAGTAACTCATCTGCCGTTGTTGTAGAGTGGTTGAATAAACATAAGCACAAATCGGTCC
TCTACGTCTCTTTTGGGAGCACAATTAGATTCCCAGAGGAGCAACTCGCTGAAATCGCAAAAGCTCTAGAAGCTTCTACCGTCCCTTTCATTTG
GGTAGTAAACAAAGACCAATTAGCAAAAACCACGTGGTTACCGGAGAGTTTGTTCGATGAGAAAAAAATGTCTGATTATTAAAGGATGGGCAC
CGCAACTAACCATCTTAGATCATTCAGCAGTCGGAGGATTCATGACACACTGTGGTTGGAATTCAGTGCTTGAAGCCATCATCGCTGGGGTGC
CGTTGGTG
SGT2_sto40_seq1
CCTGCGGATGAGAGGAATGCTTTTGATGAATTGCTCGATCGAACCAGAGAATCTGAGGATCTAAGCTACGGTATCGTTCATGATACTTTTTAC
GAGCTAGAACCTGCCTACGCTGACTACTATCAGAAGATGAAGAAAACCAAATGTTGGCAAATTGGTCCCATTTCCTATTTTTCTTCCAAATTAT
CCCCAAGAAAAGAACTGATTAATTCTTCTGATGAAAGTAACTCATCTGCCGTTGTTGTAGAGTGGTTGAATAAACATAAGCACAAATCGGTCC
TCTACGTCTCTTTTGGGAGCACAATTAGATTCCCAGAGGAGCAACTCGCTGAAATCGCAAAAGCTCTAGAAGCTTCTACCGTCCCTTTCATTTG
GGTAGTAAACAAAGACCAATTAGCAAAAACCACGTGGTTACCGGAGAGTTTGTTCGATGAGAAAAAAATGTCTGATTATTAAAGGGTGGGCAC
CGCAACTATCCATCTTAGATCATTCAGCAGTCGGAGGATTCATGACACACTGTGGTTGGAATTCAGTGCTTGAAGCCATCATCGCTGGGGTGC
CGTTGGTG
SGT2_sto40_seq1-2
CCTGCGGATGAGAGGAATGCTTTTGATGAATTGCTCGATCGAACCAGAGAATCTGAGGATCTAAGCTACGGTATCGTTCATGATACTTTTTAC
GAGCTAGAACCTGCCTACGCTGACTACTATCAGAAGATGAAGAAAACCAAATGTTGGCAAATTGGTCCCATTTCCTATTTTTCTTCCAAATTAT
CCCCAAGAAAAGAACTGATTAATTCTTCTGATGAAAGTAACTCATCTGCCGTTGTTGTAGAGTGGTTGAATAAACATAAGCACAAATCGGTCC
TCTACGTCTCTTTTGGGAGCACAATTAGATTCCCAGAGGAGCAACTCGCTGAAATCGCAAAAGCTCTAGAAGCTTCTACCGTCCCTTTCATTTG
GGTAGTAAACAAAGACCAATTAGCAAAAACCACGTGGTTACCGGAGAGTTTGTTCGATGAGAAAAAAATGTCTGATTATTAAAGGGTGGGCAC
CGCAACTATCCATCTTAGATCATTCAGCAGTCGGAGGATTCATGACACACTGTGGTTGGAATTCAGTGCTTGAAGCCATCATCGCTGGGGTGC
CGTTGGTG
SGT2_sto61_seq2
CCTGCGGATGAGAGGAATGCTTTTGATGAATTGCTCGATCGAACCAGAGAATCTGAGGATCTAAGCTACGGTATCGTTCATGATACTTTTTAC
GAGCTAGAACCTGCCTACGCTGACTACTATCAGAAGATGAAGAAAACCAAATGTTGGCAAATTGGTCCCATTTCCTATTTTTCTTCCAAATTAT
CCCCAAGAAAAGAACTGATTAATTCTTCTGATGAAAGTAACTCATCTGCCGTTGTTGTAGAGTGGTTGAATAAACATAAGCACAAATCGGTCC
TCTACGTCTCTTTTGGGAGCACAATTAGATTCCCAGAGGAGCAACTCGCTGAAATCGCAAAAGCTCTAGAAGCTTCTACCGTCCCTTTCATTTG
GGTAGTAAACAAAGACCAATTAGCAAAAACCACGTGGTTACCGGAGAGTTTGTTCGATGAGAAAAAAATGTCTGATTATTAAAGGGTGGGCAC
CGCAACTATCAATCTTAGATCATTCAGCAGTCGGAGGATTCATGACACACTGTGGTTGGAATTCAGTGCTTGAAGCCATCATCGCTGGGGTGC
CGTTGGTG
SGT2_sto61_seq2-2
CCTGCGGATGAGAGGAATGCTTTTGATGAATTGCTCGATCGAACCAGAGAATCTGAGGATCTAAGCTACGGTATCGTTCATGATACTTTTTAC
GAGCTAGAACCTGCCTACGCTGACTACTATCAGAAGATGAAGAAAACCAAATGTTGGCAAATTGGTCCCATTTCCTATTTTTCTTCCAAATTAT
CCCCAAGAAAAGAACTGATTAATTCTTCTGATGAAAGTAACTCATCTGCCGTTGTTGTAGAGTGGTTGAATAAACATAAGCACAAATCGGTCC
TCTACGTCTCTTTTGGGAGCACAATTAGATTCCCAGAGGAGCAACTCGCTGAAATCGCAAAAGCTCTAGAAGCTTCTACCGTCCCTTTCATTTG
GGTAGTAAACAAAGACCAATTAGCAAAAACCACGTGGTTACCGGAGAGTTTGTTCGATGAGAAAAAAATGTCTGATTATTAAAGGGTGGGCAC
CGCAACTATCAATCTTAGATCATTCAGCAGTCGGAGGATTCATGACACACTGTGGTTGGAATTCAGTGCTTGAAGCCATCATCGCTGGGGTGC
CGTTGGTG
SGT2_chc80-1_seq1
CCTGCGGATTGGAGGAATGCTTTTGATGAATTGCTTGATCGAACCAGAGAATCTGAGGATCTAAGCTACGGTATCATTCATGATACTTTTTAC
GAGCTAGAACCTGCCTACGCTGAGTACTATCAGAAGATGAAGAAAACCAAATGTTGGCAAATTGGTCCCATTTCCTATTTTTCTTCCAAATTAT
TCCGAAGAAAAGAACTGATTAATTCTTCTGATGAAAGTAACTCATCTGCCGTTGTTGTAGAGTGGTTGAATAAACAGAAGCACAAATCGGTCC
TCTACGTCTCTTTTGGGAGCACAGTTAGATTCCCAGAGGAGCAACTCGCTGAAATCGCAAAAGCTCTAGAAGCTTCTACCGTCCCTTTCATTTG
GGCAGTGAACAAGGACCAATCAGCAAAAATCACGTGGTTACCGGAGAGTTTGTTCGATGAGAAAAAAATGTCTGATTATTAAAGGGTGGGCG
CCGCAACTAACCATCTTAGATCATTCAGCAGTCGGAGGATTCATGACACACTGTGGTTGGAATTCAGTGCTTGAAGCCATCATCGCTGGGGTG
CCGTTGGTG
SGT2_chc80-1_seq1-2
CCTGCGGATTGGAGGAATGCTTTTGATGAATTGCTTGATCGAACCAGAGAATCTGAGGATCTAAGCTACGGTATCATTCATGATACTTTTTAC
GAGCTAGAACCTGCCTACGCTGAGTACTATCAGAAGATGAAGAAAACCAAATGTTGGCAAATTGGTCCCATTTCCTATTTTTCTTCCAAATTAT
TCCGAAGAAAAGAACTGATTAATTCTTCTGATGAAAGTAACTCATCTGCCGTTGTTGTAGAGTGGTTGAATAAACAGAAGCACAAATCGGTCC
TCTACGTCTCTTTTGGGAGCACAGTTAGATTCCCAGAGGAGCAACTCGCTGAAATCGCAAAAGCTCTAGAAGCTTCTACCGTCCCTTTCATTTG
GGCAGTGAACAAGGACCAATCAGCAAAAATCACGTGGTTACCGGAGAGTTTGTTCGATGAGAAAAAAATGTCTGATTATTAAAGGGTGGGCG
CCGCAACTAACCATCTTAGATCATTCAGCAGTCGGAGGATTCATGACACACTGTGGTTGGAATTCAGTGCTTGAAGCCATCATCGCTGGGGTG
CCGTTGGTG
SGT2_phuDH_seq1
CCTGCGGATGAGAGGAATGCTTTTGATGAATTGCTCGATCGAACCAGAGAATCTGAGGATCTAAGCTACGGTATCGTTCATGATACTTTTTAC
GAGCTAGAACCTGCCTACGCTGACTACTATCAAAAGATGAAGAAAACCAAATGTTGGCAAATTGGTCCCATTTCCTATTTTTCTTCCAAATTAT

TCCGAAGAAAAGAACTGATTAATTCTTCTGATGAAAGTAACTCATCTGCCGTTGTTGTAGAGTGGTTGAATAAACACAAGCACAAATCGGTCC
TCTACGTCTCTTTTGGGAGCACAGTTAGATTCCCAGAGGAGCAACTCGCTGAAATCGCAAAAGCTCTAGAAGCTTCTACCGTCCCTTTCATTTG
GGCAGTGAACAAGGACCAATCCACGTGGTTACCGGAGAGTTTGTTCGATGAGAAAAAATGTCTGATTATTAAAGGGTGGGCACCGCAACTA
ACCATCTTAGATCATTCAGCAGTCGGAGGATTCATGACACACTGTGGTTGGAATTCAGTGCTTGAAGCCATCATCGCTGGGGTGCCGTTGGTG
SGT2_phuDH_seq1-2
CCTGCGGATGAGAGGAATGCTTTTGATGAATTGCTCGATCGAACCAGAGAATCTGAGGATCTAAGCTACGGTATCGTTCATGATACTTTTTAC
GAGCTAGAACCTGCCTACGCTGACTACTATCAAAAGATGAAGAAAACCAAATGTTGGCAAATTGGTCCCATTTCCTATTTTTCTTCCAAATTAT
TCCGAAGAAAAGAACTGATTAATTCTTCTGATGAAAGTAACTCATCTGCCGTTGTTGTAGAGTGGTTGAATAAACACAAGCACAAATCGGTCC
TCTACGTCTCTTTTGGGAGCACAGTTAGATTCCCAGAGGAGCAACTCGCTGAAATCGCAAAAGCTCTAGAAGCTTCTACCGTCCCTTTCATTTG
GGCAGTGAACAAGGACCAATCCACGTGGTTACCGGAGAGTTTGTTCGATGAGAAAAAATGTCTGATTATTAAAGGGTGGGCACCGCAACTA
ACCATCTTAGATCATTCAGCAGTCGGAGGATTCATGACACACTGTGGTTGGAATTCAGTGCTTGAAGCCATCATCGCTGGGGTGCCGTTGGTG
SGT2_phu_DM_all-1
CCTGCGGATGAGAGGAATGCTTTTGATGAATTGCTCGATCGAACCAGAGAATCTGAGGATCTAAGCTACGGTATCGTTCATGATACTTTTTAC
GAGCTAGAACCTGCCTACGCTGACTACTATCAAAAGATGAAGAAAACCAAATGTTGGCAAATTGGTCCCATTTCCTATTTTTCTTCCAAATTAT
TCCGAAGAAAAGAACTGATTAATTCTTCTGATGAAAGTAACTCATCTGCCGTTGTTGTAGAGTGGTTGAATAAACACAAGCACAAATCGGTCC
TCTACGTCTCTTTTGGGAGCACAGTTAGATTCCCAGAGGAGCAACTCGCTGAAATCGCAAAAGCTCTAGAAGCTTCTACCGTCCCTTTCATTTG
GGCAGTGAACAAGGACCAATCCACGTGGTTACCGGAGAGTTTGTTCGATGAGAAAAAATGTCTGATTATTAAAGGGTGGGCACCGCAACTA
ACCATCTTAGATCATTCAGCAGTCGGAGGATTCATGACACACTGTGGTTGGAATTCAGTGCTTGAAGCCATCATCGCTGGGGTGCCGTTGGTG
SGT2_phu_DM_all-1-2
CCTGCGGATGAGAGGAATGCTTTTGATGAATTGCTCGATCGAACCAGAGAATCTGAGGATCTAAGCTACGGTATCGTTCATGATACTTTTTAC
GAGCTAGAACCTGCCTACGCTGACTACTATCAAAAGATGAAGAAAACCAAATGTTGGCAAATTGGTCCCATTTCCTATTTTTCTTCCAAATTAT
TCCGAAGAAAAGAACTGATTAATTCTTCTGATGAAAGTAACTCATCTGCCGTTGTTGTAGAGTGGTTGAATAAACACAAGCACAAATCGGTCC
TCTACGTCTCTTTTGGGAGCACAGTTAGATTCCCAGAGGAGCAACTCGCTGAAATCGCAAAAGCTCTAGAAGCTTCTACCGTCCCTTTCATTTG
GGCAGTGAACAAGGACCAATCCACGTGGTTACCGGAGAGTTTGTTCGATGAGAAAAAATGTCTGATTATTAAAGGGTGGGCACCGCAACTA
ACCATCTTAGATCATTCAGCAGTCGGAGGATTCATGACACACTGTGGTTGGAATTCAGTGCTTGAAGCCATCATCGCTGGGGTGCCGTTGGTG
SGT2_tbr_seq1
CCTGCGGATGAGAGGAATGCTTTTGATGAATTGCTCGATCGAACCAGAGAATCTGAGGATCTAAGCTACGGAATCGTTCATGATACTTTTTAC
GAGCTAGAACCTGCCTACGCTGACTACTATCAGAAGATGAAGAAAACCAAATGTTGGCAAATTGGTCCCATTTCCTATTTTTCTTCCAAATTAT
CCCCAAGAAAAGAACTGATTAATTCTTCTGATGAAAGTAACTCATCTGCCGTTGTTGTAGAGTGGTTGAATAAACATAAGCACAAATCGGTCC
TCTACGTCTCTTTTGGGAGCACAATTAGATTCCCAGAGGAGCAACTCGCTGAAATCGCAAAAGCTCTAGAAGCTTCTACCGTCCCTTTCATTTG
GGTAGTAAACAAAGACCAATTAGCAAAAACCACGTGGTTACCGGAGAGTTTGTTCGATGAGAAAAAATGTCTGATTATTAAAGGGTGGGCAC
CGCAACTATCCATCTTAGATCATTCAGCAGTCGGAGGATTCATGACACACTGTGGTTGGAATTCAGTGCTTGAAGCCATCATCGCCGGGGTGC
CGTTGGTG
SGT2_tbr_seq2
CCTGCAGATGAGAGGAATGGTTTTGATGAATTGCTCGATCGAACCAGAGAATCTGAGGATCAAAGCTACGGTATCGTTCATGATACTTTTTAC
GAACTAGAACCTGCCTACGCTGACTACTATCAGAAGATGAAGAAAACCAAATGTTGGCAAATTGGTCCCATTTCCTATTTTTCTTCCAAATTAT
TCCGAAGAAAAGATCTGATTAATTCTTTTGATGAAAGTAACTCATCTGCCGCTGTTGTAGAGTGGTTGAATAAACAGAAGCACAAATCGGTCC
TCTACGTCTCTTTCGGGAGCACAGTTAAATTCCCAGAGGAGCAACTCGCTGAAATCGCAAAAGCTCTAGAAGCTTCTACCGTCCCTTTCATTTG
GGTAGTGAAGGAGGACCAATCAGCAAAAACCACCTGGTTACCGGAGAGTTTGTTCGATGAGAAAAAAGGTCTGATTATTAAAGGGTGGGCT
CCGCAACTAACCATCTTAGATCATTCAGCAGTAGGAGGATTCATGACACACTGTGGATGGAATTCGGTGCTTGAAGCTATCATCGCTGGGGT
GCCGTTGGTG
